# Genomic Distortion of Jawed Vertebrate Phylogeny

**DOI:** 10.64898/2026.06.28.735080

**Published:** 2026-06-29

**Authors:** Chase D. Brownstein, Liandong Yang, Alex Dornburg, Thomas J. Near

**Affiliations:** 1 Department of Ecology and Evolutionary Biology, Yale University, New Haven CT, USA; 2 Institute of Hydrobiology, Chinese Academy of Sciences, Beijing, China; 3 Department of Bioinformatics and Genomics, University of North Carolina Charlotte, Charlotte, NC, USA; 4 Peabody Museum, Yale University, CT, USA

## Abstract

Reconstructing patterns of evolution requires understanding the interrelationships of species, yet evolutionary relationships that defy resolution and calibration in time are commonplace across the Tree of Life. Here, we investigate the dynamics of temporal and topological uncertainty by generating a phylogeny of jawed vertebrates using 1105 exonic loci sampled for 540 species spanning all major orders and most families of gnathostomes. Across loci and DNA sequence sites, we observe rapid reductions in statistical support for the monophyly of jawed vertebrate clades that originated around the Cretaceous-Paleogene mass extinction. Phylogenetic signal was scrambled to different degrees during rapid successive divergences in multiple unrelated jawed vertebrate lineages that radiated in this interval, including birds, snakes, placental mammals, and acanthomorph fishes. In addition to showing that particular events have modified phylogenetic signal across the same loci in distantly related vertebrate clades, we also demonstrate how rates of genomic evolution affect our ability to infer the timescale of vertebrate evolution. By testing how the inclusion of lineages of ray-finned fishes with very fast and slow rates of molecular evolution changes inferences of the vertebrate evolutionary timescale, we show that the deepest divergences in ray-finned fishes may be impossible to accurately infer using sequence data and calibrations from a limited fossil record. These results hint at the macroevolutionary realities underlying topological and divergence time uncertainty across evolutionary trees.

## Introduction.

Phylogenetics has emerged as a foundational framework that unifies the biological sciences (Garland et al. 2005; Cavender-Bares et al. 2009; Dunn et al. 2014; Smith et al. 2020). The widespread application of genomic sequencing to non-model organisms has enabled unprecedented reconstruction of evolutionary relationships across the Tree of Life, including illuminating the origins of animals (Dunn et al. 2008, 2014; Simakov et al. 2022; Schultz et al. 2023) and vertebrates (Delsuc et al. 2006, 2018; Smith et al. 2013; Zhu et al. 2021; Marlétaz et al. 2024; Yu et al. 2024), and the phylogenetic relationships of the major clades of flowering plants (Zhao et al. 2021a; Zuntini et al. 2024), arthropods (Giribet and Edgecombe 2019; Su et al. 2024), mammals (Foley et al. 2023), birds (Stiller et al. 2024), and fishes (Thompson et al. 2021; Parey et al. 2023). Despite the increasing use of genome-scale data to tackle phylogenetic problems, considerable uncertainty persists surrounding the relationships of several species-rich lineages (Song et al. 2012; Jarvis et al. 2014; Prum et al. 2015; Esselstyn et al. 2017; Feng et al. 2017; Hughes et al. 2018; Friedman et al. 2019; Giribet and Edgecombe 2019; Kimball et al. 2019; Dornburg and Near 2021; Kuhl et al. 2021; Ghezelayagh et al. 2022; Mongiardino Koch et al. 2022, 2023; Álvarez-Carretero et al. 2022; Budd and Mann 2023; Foley et al. 2023; Glass et al. 2023; Su et al. 2024). In many lineages, such as neoavian birds or placental mammals, significant improvement in phylogenetic resolution is only accomplishable using tens or hundreds of thousands of loci, which necessitates the use of analyses targeting lineage-specific conserved regions (Foley et al. 2023; Stiller et al. 2024). Different types of genomic markers may result in the inference of alternative phylogenetic relationships within lineages, reflecting the varied nature of genomic ancestry and the different histories of different genes and syntenic blocks (Alda et al. 2021; Arcila et al. 2021; Foley et al. 2023; Mongiardino Koch et al. 2023; Stiller et al. 2024; Brownstein and Near 2026). For inferences at wider taxonomic scales, the assembly of such large genomic datasets is difficult owing to the ancient divergence of sampled species.

Regions of phylogenetic uncertainty frequently correspond to macroevolutionary events, especially mass extinctions, when trees are calibrated in time (Jarvis et al. 2014; Prum et al. 2015; Feng et al. 2017; Foley et al. 2023; Stiller et al. 2024; Somogyi et al. 2026). Although temporal patterns of phylogenetic discordance have been investigated within specific clades, including birds, mammals, and ray-finned fishes (Song et al. 2012; Jarvis et al. 2014; Prum et al. 2015; Esselstyn et al. 2017; Reddy et al. 2017; Ghezelayagh et al. 2022; Foley et al. 2023; Berv et al. 2024; Stiller et al. 2024), broader comparative analyses across the Tree of Life remain limited (Budd and Mann 2023). Consequently, the relative magnitude of phylogenetic uncertainty across distantly related clades and their correspondence to shared macroevolutionary phenomena remain poorly understood (O’Leary et al. 2013; Crouch et al. 2019). Moreover, lineage-specific biological attributes may influence both phylogenetic resolution and time-calibration of phylogeny (Sanderson 2002; Bromham and Woolfit 2004; Drummond et al. 2006; Lepage et al. 2007; Dornburg et al. 2012; Berv and Field 2018; Budd and Mann 2023; Berv et al. 2024; Duchêne et al. 2025). As phylogenetics enters an era of increased genome coverage for organismal diversity, data are now available to infer interactions among the structure of phylogenies and patterns of life history, ecology, and physiology in deep time (Berv and Field 2018; Berv et al. 2024).

Jawed vertebrates (*Gnathostomata*) comprise over 70,000 species present in nearly every ecosystem on the planet and are perhaps the best-studied of all animals. However, phylogenetic relationships among several jawed vertebrate clades remain contentious, a factor that for many lineages is often attributed to rapid diversification following major extinctions and biotic turnovers (Meredith et al. 2011; Song et al. 2012; Jarvis et al. 2014; Chen et al. 2015; Prum et al. 2015; Esselstyn et al. 2017; Irisarri et al. 2017; Hughes et al. 2018; Hime et al. 2020; Meyer et al. 2021; Álvarez-Carretero et al. 2022; Foley et al. 2023; Parey et al. 2023; Berv et al. 2024; Schartl et al. 2024; Stiller et al. 2024). No study has attempted to compare levels of phylogenetic uncertainty across a comprehensive sample of gnathostomes to assess global patterns of phylogenomic discordance through time in the three major gnathostome divisions, *Chondrichthyes* (cartilaginous fishes), *Actinopterygii* (ray-finned fishes), and *Sarcopterygii* (lobe-finned fishes), nor have previous analyses compared levels of phylogenetic discordance in classic vertebrate radiations such as birds, placental mammals, and acanthomorph fishes using the same genomic sequence data.

Here, we infer a phylogeny of 540 jawed vertebrates from 117 of 133 order-level (Foley et al. 2023; Near and Thacker 2024; Stiller et al. 2024) clades using a conserved set of 1,105 exonic loci (Hughes et al. 2018, 2021) to test whether directly comparable levels of phylogenetic uncertainty exist across these classic post-extinction adaptive radiations. Originally designed to circumvent the paralogy introduced by the teleost-specific whole genome duplication and interrogate ray-finned fish relationships (Hughes et al. 2018), these exons allow us to examine common patterns of phylogenetic discordance in a genomic marker set conserved across jawed vertebrates. Jawed vertebrate phylogeny, when calibrated in time, reveals tree-wide signatures of phylogenetic uncertainty and lineage origination in distantly related lineages of jawed vertebrates that correspond to major extinctions and biotic revolutions, especially the K-Pg event. We also show that biological variation in rates of protein-coding gene evolution make inferring the ages of some of the most species-rich clades of jawed vertebrates untenable. This suggests that the ages of some branches on the tree of life may not be accurately constrained past the minimum ages provided by unambiguous extinct representatives from the fossil record. These dual patterns of uncertainty in phylogenetic space and geological time have sculpted the shape of vertebrate phylogeny and prescribed the limits of what we can learn about jawed vertebrate evolutionary history from genomic data.

## Methods.

### (a) Sequence Dataset Assembly.

Constructing a comprehensive jawed vertebrate phylogeny requires accounting for hidden paralogs in ray-finned fishes (several lineages underwent independent whole-genome duplication episodes) when creating alignments. As such, we used the set of 1,105 exon loci published by Hughes et al. (Hughes et al. 2018, 2021). as a basis for producing a genome-wide marker sequence dataset to investigate jawed vertebrate phylogenetic relationships. Although we recognize that tens of thousands of markers may be necessary to resolve the relationships of clades in rapid radiations such as neoavian birds (Stiller et al. 2024), our interests were in checking for the presence of similar patterns of phylogenetic discordance across a broad sample of jawed vertebrates. We pooled data from Hughes et al. (Hughes et al. 2018, 2021) with sequences extracted using available whole genome assemblies on NCBI for non-actinopterygian vertebrates, including 13 chondrichthyans, the coelacanth *Latimeria chalumnae*, two lungfishes, 11 amphibians, 69 mammals, 39 lepidosaurs, 22 turtles, four crocodylians, and 60 birds, as well as 20 additional teleosts and holosteans for a total of 542 tips representing 540 species (see Brownstein et al. (Brownstein et al. 2023b, 2024a) for the inclusion of all seven living species of gars). The dataset represents a more than five-fold increase in sampling relative to previous attempts to reconstruct jawed vertebrate phylogeny, which sampled between 58 and 100 taxa and did not sample all of the deepest divergences in ray-finned fishes (Chen et al. 2015; Irisarri et al. 2017; Hime et al. 2020). Exon sequences were located in available genomes using HMMER 3.1 (Wheeler and Eddy 2013), extracted using the custom python scripts from Hughes et al. (Hughes et al. 2018; Brownstein et al. 2024a), and finally aligned using MAFFT 7.3 (Katoh and Standley 2013). Together, the exon sequence dataset included 729935 bp with a mean length of 661 bp per exon. We also used *phyluce* 1.7.3 (Faircloth 2016) to produce a 75% complete taxon-sequence matrix in addition to the original 1,105 exons; this subsampling step retained 662 (60% of the total) exon loci. We also used *CIAlign* (118) to identify and remove chimaeric alignments. Table S1 includes information on all sequences extracted from genomes available on the NCBI repository GenBank. For the 1,105 exon dataset, the total and average number of parsimony informative, singleton, and invariant sites were 53824 and 1922.29, 9364 and 302, and 56066 and 1808.6, respectively. For the 662 exon dataset, the total and average number of parsimony informative, singleton, and invariant sites were 26844 and 2237, 4099 and 341.58, and 23312 and 1943.66, respectively.

### (b) Maximum Likelihood Phylogenetic Analyses and Nodal Support.

We inferred phylogenies using the original and 75% complete datasets using both concatenation and multispecies coalescent approaches. First, we used IQ-TREE 2 (Nguyen et al. 2015; Minh et al. 2020b) to infer maximum likelihood phylogenies from the concatenated exon sequence datasets. In each case, we used ModelFinder (Kalyaanamoorthy et al. 2017) to select best-fit models of sequence evolution and calculated ultrafast bootstraps over 1000 replicates. For multispecies coalescent inference, we inferred gene trees for exons in IQ-TREE2 using ModelFinder (Kalyaanamoorthy et al. 2017) to find best-fit models, and then used ASTRAL-III (Zhang et al. 2018) to infer a multispecies coalescent tree from the individual gene trees. In addition to ultrafast bootstrap supports, we further quantified statistical support for individual branches along the inferred phylogenies by inferring gene and site concordance factors for the phylogenies inferred from the original and 75% complete exon matrices. Gene and site concordance factors measure the number of decisive gene trees and sites, respectively, that are congruent with an input species tree topology (in this case, the concatenated trees were used as input trees) (Minh et al. 2020a). As such, concordance factors can provide information on patterns of support across two different levels of information in the genome: genes and sites. We conducted all maximum likelihood phylogenetic analyses and concordance factor calculations on the Yale High Performance Computing Cluster McCleary. We examined associations between different levels of support and compared gene and site concordance factors, multispecies coalescent support values, and ultrafast bootstrap values for clades in the inferred phylogenies using heatmaps and scatterplots built using custom scripts in the R package *ggplot2* (Valero-Mora 2010).

### (c) Anomaly Zone Detection.

We further interrogated statistical support for branches along the inferred phylogenies by inferring the position of anomaly zones along the ASTRAL species trees. Anomaly zones (AZs) are regions of species trees where an alternative topology is supported more strongly in a handful of gene trees than the consensus topology is in the species tree (Degnan and Rosenberg 2006, 2009). The presence of anomaly zones in regions of a tree is usually taken to indicate rapid successive divergences or high effective population sizes (Degnan and Rosenberg 2006, 2009; Liu and Edwards 2009; Linkem et al. 2016; Chafin et al. 2021; Brownstein et al. 2024b). We used custom python scripts(Linkem et al. 2016; Chafin et al. 2021) to check how many anomalous bipartitions included each branch in the input ASTRAL-III species trees generated using the original and 75% complete exon matrices. Branches that were present in more than 10 anomalous bipartitions were considered to be in anomaly zones.

### (d) Time Calibration of Jawed Vertebrate Phylogeny.

In order to produce a time-calibrated phylogeny of jawed vertebrates, we conducted Bayesian node-dating analysis in BEAST 2.6.7 (Bouckaert et al. 2014, 2019) on three sets containing the 150 largest exon sequences from the 75% complete dataset for 375 representative species. For time-calibration, we fixed the phylogeny by pruning the concatenated maximum likelihood tree generated from the 75% complete matrix. We placed minimum bounds on nodes in the tree using 52 fossil calibrations. Because fossil calibrations can extensively change our understanding of the timescale of evolution, even fossils that have been allied with crown groups in phylogenetic analyses should be rigorously evaluated (Parham et al. 2012; Ksepka et al. 2015; Whiteside et al. 2022; Brownstein et al. 2023a). As such, we vetted the phylogenetic placement of included fossil calibrations through a comprehensive literature review and phylogenetic analysis of morphological character matrices when character states uniting taxa used as fossil calibrations with the relevant crown clades in jawed vertebrates were not provided (Brownstein et al. 2022, 2023a; Brownstein 2023b; Brownstein and Near 2024). This approach allowed us to supply rigorous justification of fossil calibration placement based on the observation of phylogenetically optimized character states. For each calibration, we also searched the literature for penecontemporaneous occurrences of equivalent calibrations, such that in many cases our fossil calibrations are based on multiple coeval extinct taxa or would only need slight modification in cases where the phylogenetic position of the principal fossil calibration is revised. To this extent, we reran the time-calibration analyses with a modified set of fossil calibrations to test for whether the exclusion of a handful of somewhat controversial mammal fossils affected the ages that we inferred (Table S2). A full list of fossil calibration justifications, including lists of optimized character states that unite extinct species used as fossil calibrations with crown clades in jawed vertebrates, is included in the Supplementary Material, following best practice recommendations (Parham et al. 2012).

For the root node, we set the origin prior to 443.0 Ma, the earliest Silurian, which is the earliest age for potential jawed vertebrate material from the fossil record (The distribution of the vertebrates in the Late Ordovician and Early Silurian palaeobasins of the Siberian Platform 1995). We set the bounds on the root prior to 439.0 Ma, the age of the oldest known unambiguous crown-group jawed vertebrate †*Fanjingshania renovata* (Pan-*Chondrichthyes*)(Andreev et al. 2022) and 509.0 Ma, the lower bound of the Cambrian Series 2. This is the maximum age of the Burgess Shale, which contains several fossil chordates but no crown vertebrates (Morris and Caron 2012, 2014; Mussini et al. 2024). For each fossil calibration, we inputted bounds on the prior distribution of specified clades such that 97.5% of the distribution fell before the age of the fossil used. Although maximum age bounds have been proposed for some nodes calibrated in this phylogeny, we chose to sculpt prior distributions in this manner to account for uncertainty regarding early fossil occurrences following previous studies (Prum et al. 2015; Ghezelayagh et al. 2022; Brownstein et al. 2024b, 2025), which should in any case postdate the origination of crown clades. We placed the fossil calibrations on the tree using monophyletic MRCA priors (Table S3).

We used a General Time Reversible model of sequence evolution, a Relaxed Lognormal Clock Model, and the BEAST2 implementation of the Fossilized Birth-Death (FBD) Model. We set the diversification rate to 0.025, which was found by rounding from the approximation found using the equation in Magallón and Sanderson (Magallón and Sanderson 2001) using the input values of 439.0 Ma for the clade age and 75,681 (the current sum of jawed vertebrate species as of February 2025 taken from Eschemeyer’s Catalogue of Fishes, the Reptile Database, The Clements Checklist of Birds of the World, the ASM Mammal Diversity Database, and AmphibiaWeb) for the species count. Finally, we ran each exon set three times independently over 4.0 × 10^8^ generations with a 1.0 × 10^8^ pre-burnin, checked the log files for convergence of the posteriors and ESS values >200 using Tracer v 1.7 (Rambaut et al. 2018), combined the top 5% of trees from the combined set of nine runs (=3.6 billion generations) using LogCombiner v.2.6.6 (Bouckaert et al. 2019), and summarized the posterior trees in a single tree with common ancestor heights annotated onto the input concatenated maximum likelihood topology. For each exon set, we also produced trees with median node heights annotated onto the maximum likelihood topology to check the similarity of divergence times estimated from different exon sets.

### (e) Analyses of Support Through Time.

We matched gene and site concordance values, ultrafast bootstrap support values, and branch lengths in substitutions per site from the 75% concatenated maximum likelihood phylogeny to the corresponding branches in the time-calibrated phylogeny generated from pooling posterior tree sets from the nine independent node-dating runs to assess for patterns of change in phylogenetic support over deep time. Similarly, we assessed how the length of branches in millions of years changed through time to see whether certain intervals of jawed vertebrate evolutionary history have featured a higher number of short branching events and replicated this analysis across trees generated from each of the three exon sets. We used custom scripts in R to plot all these values through time (Valero-Mora 2010). Next, we subsampled nodes to only those with median ages within 10 million years of the K-Pg boundary (76.02 to 56.02 Ma) and explored variance in node support across ecological categories, clades, and evolutionary radiations. We determined whether nodes in this subsample belonged to classic post-extinction radiations, such as birds (Hackett et al. 2008; Jarvis et al. 2014; Brusatte et al. 2015; Prum et al. 2015; Kuhl et al. 2021; Berv et al. 2024; Stiller et al. 2024), acanthomorphs (Friedman 2010; Near et al. 2013; Friedman et al. 2019; Ghezelayagh et al. 2022), some otophysan fish orders (*Cypriniformes*, *Siluriformes*)(Kappas et al. 2016; Bagley et al. 2018; Roxo et al. 2019; Day et al. 2023), placental mammal orders (Meredith et al. 2011; dos Reis et al. 2012, 2014; Phillips and Fruciano 2018; Upham et al. 2021; Álvarez-Carretero et al. 2022; Foley et al. 2023), and snakes (Burbrink et al. 2020; Klein et al. 2021), and compared concordance factors for these nodes to those that appeared in the same time period but are not associated with classic adaptive radiations. For ecological characterization, we based our scorings on presumed ancestral ecology. For example, the nodes representing the MRCA of penguins (*Spheniscidae*) with other waterbirds were coded as ‘flighted’ since penguins are secondarily flightless (Cole et al. 2022).

### (f) In silico *Divergence Time Estimation Experiments.*

To explore how lineage-specific rates of molecular evolution might affect our inferences of the timescale of jawed vertebrate evolution, we conducted *in silico* taxon and fossil calibration inclusion and exclusion experiments on a subset of 45 species of non-tetrapod sarcopterygians and actinopterygians sampling all major lineages in lungfishes, coelacanths, and ray-finned fishes (*Polypteridae*, *Acipenseriformes*, *Holostei*, *Oseanacephala, Otocephala*, and *Euteleostei*). We selected this region of the tree for *in silico* divergence time testing because of the known heterogeneity in rates of substitution per site among the first four divergences in ray-finned fishes (Takezaki 2018; Brownstein et al. 2024a) and the long ghost lineages for these divergences implied by the ages inferred by previous molecular phylogenies (Near et al. 2012; Betancur-R et al. 2013; Giles et al. 2017; Hughes et al. 2018), which suggest that the earliest known fossil actinopterygians, actinopterians, neopterygians, and teleosts postdate the origins of these clades by up to 100 million years (Hurley et al. 2006; Giles et al. 2017; Friedman 2022). For all tests on the subset of 45 taxa, we ran Bayesian node-dating analysis in BEAST2 on two of the three 50 exon sets over 1.0 × 10^8^ generations with a 1.0 × 10^8^ pre-burnin, checked the log files for convergence of the posteriors and ESS values >200 using Tracer v 1.7 (Rambaut et al. 2018), combined the top 5% of trees from the combined set of nine runs (=3.6 billion generations) using LogCombiner v.2.6.6 (Bouckaert et al. 2019), and summarized the posterior trees in a single tree with median node heights annotated onto the input concatenated maximum likelihood topology. We analyzed the following permutations to the 45 taxon dataset:

1. The full 45 taxon dataset, with all relevant fossil calibrations included, to provide a base of comparison.
2. A reduced 40 taxon dataset, excluding holosteans (gars and bowfins) and acipenseriforms (sturgeons and paddlefishes), to test whether the exclusion of living fossil ray-finned fish lineages with slow rates of nucleotide substitution modifies the inferred ages of crown *Actinopterygii* and crown *Teleostei*.
3. The full the 45 taxon dataset, but with fossil calibrations providing hard constraints on the ages of the lepisosteid and acipenseriform crown groups removed. Because these fossil calibrations, †*Atractosteus falipoui* (Grande 2010) and †*Protopsephurus liui* (Grande et al. 2002) have been reliably placed within the gar and acipenseriform crown groups in numerous phylogenetic analyses of morphological characters (Grande et al. 2002; Grande 2010; Hilton et al. 2011, 2023; Brownstein 2022; Brownstein and Lyson 2022; Brownstein et al. 2023b), and because many similarly old or slightly geologically younger crown gars and acipenseriforms are known from complete skeletons (Grande and Bemis 1991; Grande 2010; Hilton et al. 2011, 2023; Brito et al. 2017; Sato et al. 2018; Brownstein and Lyson 2022; Brownstein et al. 2023b; Murray et al. 2023), appreciably younger ages for the gar and acipenseriform crown groups found when these fossil calibrations are excluded would conflict directly with a well-established fossil record and provide support for the hypothesis that slow and fast rates of nucleotide substitution confound inference of the ages of the deepest divergences in ray-finned fishes without minimum age constraints from fossils.

We then recorded the median and 95% highest posterior density bounds for divergence times and branch-wise rates of substitution across different analyses and plotted them together (Table S4). We compared these rate and divergence time values to those of the time-calibrated phylogeny built using all three exon sets and the 375 species set. Finally, we used the application FigTree (http://tree.bio.ed.ac.uk/software/figtree/) to visualize median rates of nucleotide substitution across the 375 taxon phylogenies made using each and all of the three exon sets and the 40 and 45 taxon phylogenies built using two of the three exon datasets.

## Results.

### (a) Concordance and discordance across jawed vertebrate phylogeny and in time.

We assembled a dataset of 1,105 orthologous exon loci (Hughes et al. 2018, 2021; Brownstein et al. 2024a) for 542 jawed vertebrates representing all major divisions (e.g., *Chondrichthyes, Polypteridae, Acipenseriformes, Holostei, Oseanacephala, Clupeocephala, Lepidosauria, Amphibia, Mammalia, Squamata, Testudines, Crocodylia,* and *Aves*) and 88% of orders, inferred phylogenies using multispecies coalescent (Zhang et al. 2018) and concatenated maximum likelihood (Minh et al. 2020b) approaches, and inferred a time-calibrated evolutionary tree under a Uncorrelated Relaxed Log-Normal clock and General Time Reversible model in a Bayesian framework (Bouckaert et al. 2014, 2019) using 52 fossil calibrations rigorously vetted using optimized character states from phylogenetic analyses of morphological characters (Supplementary Information).

**Figure 1.**
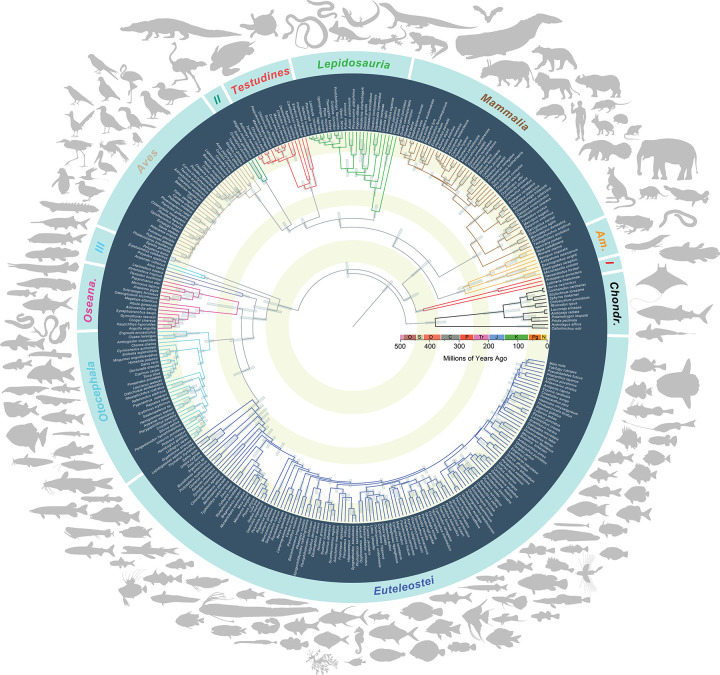
Time-Calibrated Phylogeny of Jawed Vertebrates. Radially projected Bayesian node-calibrated phylogeny of 375 representative species of jawed vertebrates based on the topology inferred for 1105 concatenated exon loci sampled for 542 species under a maximum likelihood criterion in IQ-TREE2. The phylogeny was time-calibrated in BEAST 2.6.7 using 52 vetted fossil calibrations justified in the Supplementary Information. Bars at nodes indicate 95% highest posterior density (HPD) intervals for divergence times. Major vertebrate clades are noted around the phylogeny, and abbreviations are as follows, counterclockwise: *Chond*., *Chondrichthyes*; *I*, *Latimeria-Dipnoi* clade; *Am*., *Amphibia*; *II*, *Crocodylia*; *III*, non-teleost actinopterygians, including *Polypteridae*, *Acipenseriformes*, and *Holostei*; *Oseana*., *Oseanacephala*.(Near and Thacker 2024) Borders between tan and white concentric regions denote the timing of the ‘big five’ mass extinctions: Ordovician-Silurian, End-Devonian, Permo-Triassic, End-Triassic, and Cretaceous-Paleogene. Silhouettes are public domain from phylopic.org or by CDB.

**Figure 2.**
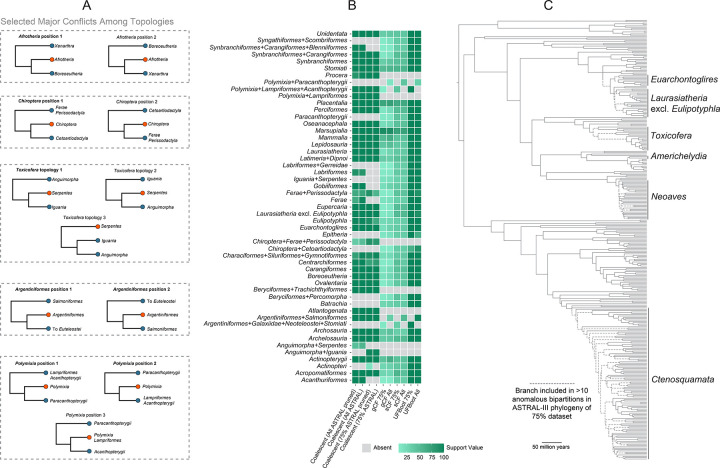
Discordance Across Jawed Vertebrate Phylogeny. (A) Selected major conflicts among trees built in this and previous(Song et al. 2012; dos Reis et al. 2014; Esselstyn et al. 2017; Hughes et al. 2018; Burbrink et al. 2020; Singhal et al. 2021; Ghezelayagh et al. 2022; Foley et al. 2023; Title et al. 2024) phylogenies built using genome-wide sequence data. (B) Heatmap of support values across different metrics for selected clades. gCF, gene concordance factor; sCF, site concordance factor; UFBoot, ultrafast bootstrap. (C) Time-calibrated phylogeny shown in Figure 1 annotated with the corresponding inferred locations of anomaly zones on the ASTRAL-III species tree, indicated by dashed lines.

Our phylogeny (Figure 1; Figure 2) compares favorably to previous large-scale phylogenomic analyses of jawed vertebrate clades in resolving several relationships that conflict with traditional morphology-based phylogenies, including the resolution of a clade containing odd-toed ungulates, carnivoran mammals, and pangolins (Song et al. 2012; Esselstyn et al. 2017; Álvarez-Carretero et al. 2022; Foley et al. 2023), the placement of turtles as the living sister to archosaurs (birds and crocodylians)(Cao et al. 2000; Chiari et al. 2012; Crawford et al. 2012; Wang et al. 2013; Chen et al. 2015; Hime et al. 2020), the placement of geckos as the earliest diverging clade of squamates (Pyron et al. 2013; Zheng and Wiens 2016; Streicher and Wiens 2017; Burbrink et al. 2020; Simões and Pyron 2021; Singhal et al. 2021; Title et al. 2024), the resolution of a clade containing snakes, iguanian, and anguimorph lizards (Pyron et al. 2013; Zheng and Wiens 2016; Streicher and Wiens 2017; Burbrink et al. 2020; Simões and Pyron 2021; Singhal et al. 2021; Title et al. 2024), and the resolution of the first four diverging clades of ray-finned fishes (Grande 2010; Near et al. 2012; Betancur-R et al. 2013; Hughes et al. 2018, 2021; Dornburg and Near 2021; Thompson et al. 2021; Near and Thacker 2024): bichirs and Reedfish (*Polypteridae*), sturgeons and paddlefishes (*Acipenseriformes*), gars and bowfins (*Holostei*), and all other ray-finned fishes (*Teleostei*) (Figure 1, Figure 2).

In our analyses, osteoglossomorph (mooneyes, mormyrid elephantfishes, and arowanas) and elopomorph (eels and tarpons) fishes are found to comprise a lineage, *Oseanacephala* (Near and Thacker 2024), that is the sister to all other teleosts. This clade was only recently supported using data on genome structure evolution (Parey et al. 2023) and some (though not all) (Hughes et al. 2018, 2021) whole-genome-based phylogenies (Bian et al. 2016; Hao et al. 2020; Takezaki 2021). We also resolve a clade, *Stomiati* (Betancur-R et al. 2013; Hughes et al. 2018; Near and Thacker 2024), containing the deep-sea dragonfishes and relatives (*Stomiiformes*) and the smelts (*Osmeriformes*), reject the monophyly of the traditional *Characiformes* (containing citharinoid fishes)(Chakrabarty et al. 2017; Melo et al. 2022), and resolve the major lineages containing the over 18,900 species in percomorph fishes. In order of divergence, these are cusk eels and brotulas (*Ophidiiformes*), toadfishes (*Batrachoididae*), gobies and cardinalfishes (*Gobiiformes*), a clade containing seahorses and relatives (*Syngnathiformes*) and tunas and relatives (*Scombriformes*), and all other percomorphs, which form two major lineages (Ghezelayagh et al. 2022; Near and Thacker 2024). *Eupercaria* contains five major groups (Figure 1, Figure 2): darters, true perches, groupers, icefishes, sculpins, rockfishes, sea lions, snailfishes, sticklebacks, searobins, and eelpouts form the *Perciformes*, the sister lineage of all other *Eupercaria*. The other eupercarian clades are wrasses, parrotfishes, and stargazers (*Labriformes*), wreckfishes and deep-sea cardinalfishes (*Acropomatiformes*), black basses, sunfishes, flagtails, and relatives (*Centrarchiformes*), and a bizarre lineage containing anglerfishes, pufferfishes and *Mola mola*, butterflyfishes, seabasses, surgeonfishes, drums, and a host of other marine and especially reef-associated clades (*Acanthuriformes*). Sister to *Eupercaria* is an unnamed clade consisting of four orders: jacks, swordfishes, flatfishes, and relatives (*Carangiformes*), swamp eels and betta fishes (*Synbranchiformes*), the flyingfishes, livebearers, halfbeaks, ricefishes, New World silversides, and relatives (*Atheriniformes*), and the ‘*Blenniiformes*,’ a grade comprising species-rich families like cichlids, damselfishes, surfperches, and blennies (Figure 1; Figure 2) (Ghezelayagh et al. 2022; Near and Thacker 2024).

Oddly, we find strong support across different analyses for a clade containing coelacanths and lungfishes to the exclusion of tetrapods (Figure 1, Figure 2), which contrasts with many (Chen et al. 2015; Irisarri and Meyer 2016; Irisarri et al. 2017; Bi et al. 2021; Meyer et al. 2021; Wang et al. 2021; Schartl et al. 2024), but not all (Shan and Gras 2011), phylogenies built using large DNA sequence datasets. Many previous studies evaluating the relationships of coelacanths, lungfishes, and tetrapods did not sample the major early-diverging ray-finned fish clades (Irisarri and Meyer 2016; Meyer et al. 2021; Wang et al. 2021; Schartl et al. 2024). Our inclusion of representatives of the major chondrichthyan and actinopterygian clades means that we fully sample the initial divergences among living jawed vertebrates, which might partially account for this result. High-resolution stratigraphic columns and an appreciably complete fossil record from China show that the stem lineages of coelacanths, lungfishes, and tetrapods originated in rapid succession in the Early Devonian (Zhao et al. 2021b; Cui et al. 2022), which could lead to a lack of clarity about the relationships of these lineages (Degnan and Rosenberg 2006, 2009; Irisarri and Meyer 2016; Reddy et al. 2017; Berv et al. 2024). As such, this uncertainty might conceivably be due to the absence of phylogenetic signal retained in the initial rapid radiation of lobe-finned fishes, in addition to the inability for even over 1000 exon loci to provide clarity surrounding the relationships at the base of *Sarcopterygii*.

More importantly, our results demonstrate that the relationships of some of the most species-rich vertebrate clades, including neoavian birds (Hackett et al. 2008; Jarvis et al. 2014; Prum et al. 2015; Reddy et al. 2017; Kimball et al. 2019; Berv et al. 2024; Stiller et al. 2024), iguanians, anguimorphs, and snakes (Pyron et al. 2013; Zheng and Wiens 2016; Streicher and Wiens 2017; Burbrink et al. 2020; Singhal et al. 2021; Title et al. 2024), several interordinal relationships of placental mammals (Meredith et al. 2011; Song et al. 2012; Foley et al. 2016, 2023; Tarver et al. 2016; Esselstyn et al. 2017; Irisarri et al. 2017; Álvarez-Carretero et al. 2022), and euteleost and acanthomorph fishes (Near et al. 2012, 2013; Betancur-R et al. 2013; Hughes et al. 2018, 2021; Ghezelayagh et al. 2022) are all inferred with similar levels of uncertainty using the same genomic dataset. We investigated the factors hindering resolution of these clades' relationships through multiple approaches (Figure 2A-C; Figures S1-S4), including comparing jawed vertebrate phylogenies using both multispecies coalescent and concatenation methods, evaluating gene and site concordance factors (Minh et al. 2020a; Kück et al. 2022), and identifying and inferring the location of anomaly zones (Degnan and Rosenberg 2006, 2009). Concordance factors measure how many gene trees and sequence sites align with relationships in a species tree, while anomaly zones represent regions where a small number of gene trees strongly support alternative relationships compared to the consensus species tree. We find that phylogenetic discordance underlying the unresolved relationships of these species-rich clades (Figure 2A) is primarily due to a low number of gene histories that support a given topology (Figures S1-S4); many contentious relationships among acanthomorph and placental mammal clades, for example, are supported by only a handful of gene trees or none at all. Indeed, reducing the number of sampled exons to account for matrix incompleteness results in many branches in neoavian birds, placental mammals, and acanthomorph fishes collapsing (Figure 2B, Supplementary Information) despite receiving strong support in concatenated maximum likelihood phylogenies (Figure 1, Figure 2B) and in analyses using genome-wide data (Esselstyn et al. 2017; Hughes et al. 2018, 2021; Ghezelayagh et al. 2022; Foley et al. 2023). This supports the hypothesis that lineage-specific phylogenies that can incorporate a higher number of orthologs are necessary to resolve the relationships of clades such as neoavian birds (Stiller et al. 2024) that are entirely contained in anomaly zones in our jawed vertebrate phylogeny (Figure 2C).

**Figure 3.**
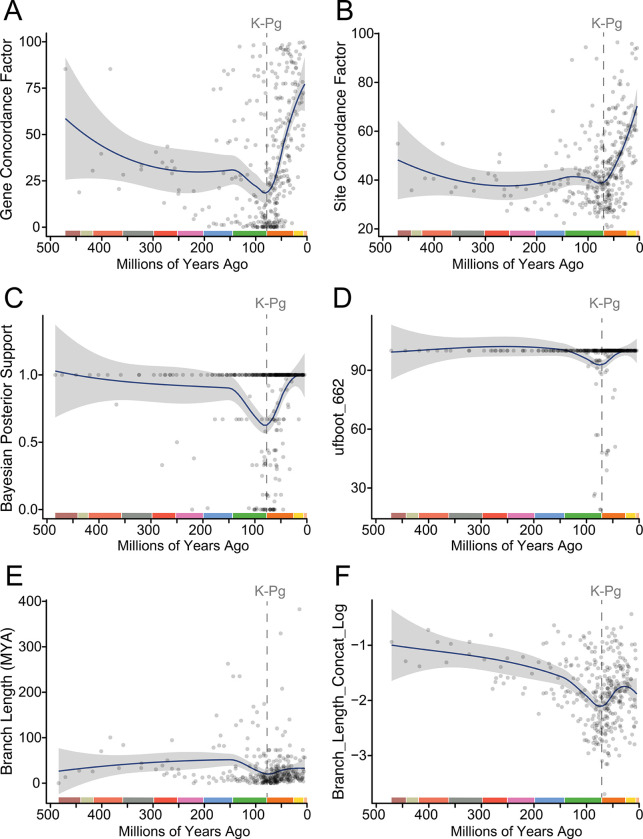
Signatures of Mass Extinction on Phylogenetic Resolution. Graphs show trendlines and 95% confidence intervals for several measures of nodal support (A-D) and branch length (E-F) through time. (A-C, F) are based on the concatenated maximum likelihood phylogeny built form the 75% complete matrix. (A) Gene concordance factors through time, (B) site concordance factors through time, (C) ultrafast bootstraps through time, (D) Bayesian posterior support through time, (E) branch length in millions of years through time, (F) branch length in substitutions per site through time. Dashed line indicates the Cretaceous-Paleogene Mass Extinction.

When placed in a temporal context, these patterns of concordance and discordance across jawed vertebrate phylogeny are associated with major macroevolutionary events, especially the Cretaceous-Paleogene mass extinction 66.02 million years ago (Gradstein et al. 2021)(Figure 3; Figures S6-S9). Gene and site concordance factors, bootstrap and Bayesian posterior supports, and branch lengths all sharply decrease at the Cretaceous-Paleogene boundary before rebounding in the Cenozoic (Figure 3). These shifts correspond to an increase in the rapidity of successive divergences among many jawed vertebrate clades, including neoavian birds, several orders of placental mammals, and pelagic scombriform and carangiform fishes, around the Cretaceous-Paleogene boundary (Figure 1, Figure 3B; Figures S6-S9; Table S2).

**Figure 4.**
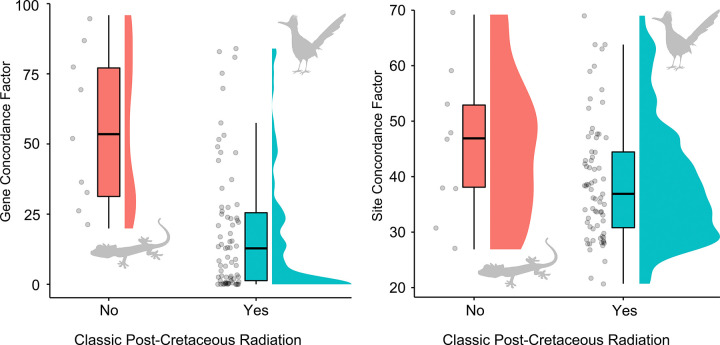
Classic Post-Cretaceous Evolutionary Radiations Show Increased Phylogenetic Discordance. Boxplot shows that, for nodes with ages falling within 10 million years of the K-Pg boundary (76.02 to 56.02 Ma), both (A) gene and (B) site concordance factors are on average lower in classic post-Cretaceous adaptive radiations, including neoavian birds, acanthomorph and placental mammal order-level clades, and snakes.

Although previous studies have found associations between phylogenetic discordance and rapid diversification following the Cretaceous-Paleogene boundary (Near et al. 2013; Jarvis et al. 2014; Prum et al. 2015; Alfaro et al. 2018; Friedman et al. 2019; Burbrink et al. 2020; Kuhl et al. 2021; Ghezelayagh et al. 2022; Foley et al. 2023; Glass et al. 2023; Berv et al. 2024; Stiller et al. 2024), these lineage-specific analyses do not allow for direct comparisons of phylogenomic discordance across jawed vertebrates as shown here. In addition to the decreases in branch lengths and phylogenetic support across genes and sites found across jawed vertebrate diversity at the K-Pg boundary (Figure 3), we also find that classic post-Cretaceous adaptive radiations recognized in the literature, including neoavian birds (Jarvis et al. 2014; Brusatte et al. 2015; Prum et al. 2015; Field et al. 2019; Kuhl et al. 2021; Berv et al. 2024; Stiller et al. 2024), various clades of acanthomorph fishes (Friedman 2010; Near et al. 2013; Alfaro et al. 2018; Friedman et al. 2019; Ghezelayagh et al. 2022; Brownstein et al. 2024b; Miller et al. 2024), and the interordinal diversifications of placental mammals (Meredith et al. 2011; Álvarez-Carretero et al. 2022; Foley et al. 2023), show moderately lower gene and site concordance factors than equivalent-age clades not associated with post-Cretaceous radiation (Figure 4). We also find a drop in phylogenetic support around the K-Pg extinction is concentrated in flighted, marine, freshwater-marine (euryhaline), and terrestrial clades; freshwater clades that diverge within 10 million years of the K-Pg are supported by comparatively high gene and site concordance factors (Figure 2, Figure 4). This result is consistent with the hypothesis that freshwater faunas are thought to have been the least affected by the K-Pg extinction (Sheehan and Fastovsky 1992; Robertson et al. 2013; Brownstein and Lyson 2022).

Our analysis reveals that mass extinctions are associated with sudden decreases in phylogenetic support across both genes and individual nucleotide sites throughout jawed vertebrate phylogeny (Figure 3). Because only a handful of lineages remain after a mass extinction and usually are represented by only small population sizes, rapid successive divergences into ecological opportunity created by mass extinctions in concert with the effects of bottleneck events might produce unclear evolutionary histories as genes and genomes experienced uneven recombination (Mirarab et al. 2024) fail to fix at the pace of rapid lineage divergence (Degnan and Rosenberg 2006; Suh et al. 2015; Reddy et al. 2017; Mirarab et al. 2024). Our analyses support the process of mass extinction and faunal recovery as a fundamental driver of discordance across the jawed vertebrate tree, which contains many of the classic examples of post-extinction evolutionary radiation (Jablonski 2001, 2005; Stroud and Losos 2016). By inducing sudden bottlenecks and opening up opportunities for lineage diversification as ecosystems reassemble, mass extinctions can scramble the genomic record of evolutionary history across diverse vertebrate lineages within a haze of rapid lineage origination, introgression, and repressed recombination.

### (b) Timing the jawed vertebrate radiation.

Improved sampling of gnathostome clades allows us to chart jawed vertebrate diversification through time in detail. For example, our analyses strongly support a Mesozoic origin and diversification of placental mammal orders, followed by diversification within orders after the Cretaceous-Paleogene mass extinction (Springer et al. 2003; Meredith et al. 2011; dos Reis et al. 2012, 2014; Phillips and Fruciano 2018; Upham et al. 2021; Álvarez-Carretero et al. 2022; Carlisle et al. 2023; Foley et al. 2023), rather than diversification of both in the Cenozoic (Figure 1, Figure S3, 6)(Alroy 1999; Archibald and Deutschman 2001; Wible et al. 2007). The conservative placement of several of our node calibrations, including for *Carnivora* and *Arctoidea*, should skew posterior ages younger if crown clades originate in the Paleocene. Furthermore, the estimated ages of placental mammal orders are robust to the use of different fossil calibration schemes that alternately exclude contentious and recently debated fossils for the setting of prior age distributions (Table S2).Thus, we remain confident in the inferred Cretaceous origin of placental order-level clades. Similarly, our results support the inference that three to five avian clades survived the Cretaceous-Paleogene extinction (Jarvis et al. 2014; Prum et al. 2015; Berv and Field 2018; Berv et al. 2024; Stiller et al. 2024) and soundly reject a Mesozoic diversification of crown birds (Figure 1, Figure S3, 6)(Jetz et al. 2012; Wu et al. 2024).

Our time-calibrated phylogenies using different sets of fossil calibrations and exonic loci also consistently estimate rapid Paleogene diversifications of several major ray-finned fish clades, including eels (*Anguilliformes*), minnows, carps, loaches, and suckers (*Cypriniformes*), catfishes (*Siluriformes*), opahs and oarfishes (*Lampriformes*)(Brownstein and Near 2024), tunas and mackerels (*Scombriformes*),(Friedman et al. 2019) jacks, swordfishes, and flatfishes (*Carangiformes*)(Ribeiro et al. 2018; Glass et al. 2023), anglerfishes (*Lophioidei*)(Brownstein et al. 2024b; Miller et al. 2024), and the orders of *Eupercaria* (Figure 1; Figures S4–6). Several deeply-divergent vertebrate clades also appear to have originated after mass extinctions. For example, the major reptile crown clades (*Lepidosauria* and *Archosauria*) and the amphibian crown group appear close to the Permo-Triassic mass extinction, which is consistent with the late Permian origins and rapid Triassic diversification of crown reptiles and amphibians implied by the fossil record (Marjanović and Laurin 2007; Anderson et al. 2008; Brusatte et al. 2010; Nesbitt 2011; Jones et al. 2013; Bernardi et al. 2015; Simões et al. 2018, 2022; Ezcurra et al. 2020; Sander et al. 2021; Simões and Pyron 2021; Kear et al. 2023; Kligman et al. 2023). Thus, the timescale of evolution for many jawed vertebrate clades matches expectations from the hypothesis that mass extinctions were key events in shaping living vertebrate diversity.

### (c) Some jawed vertebrate clade ages are highly unreliable.

Our analyses reveal that molecular age estimates for certain clades remain unreliable even when hundreds of conserved loci are analyzed, particularly in groups with sparse fossil records. To investigate the patterns and underlying causes of age estimation inaccuracies across jawed vertebrate phylogeny, we conducted computational experiments examining how taxonomic sampling and fossil calibration selection influence the estimated ages of major sarcopterygian and actinopterygian crown clades (Figure 4). Divergence times for basal actinopterygian lineages estimated using molecular data consistently precede the oldest unambiguous crown-group fossils by 100 to 150 million years in time (Giles et al. 2017; Friedman 2022). This discrepancy has largely been attributed to a poor fossil record, and several putative members of the ray-finned fish crown have been described from the Paleozoic (Hurley et al. 2006; Argyriou et al. 2018, n.d.). However, this disagreement between the fossil record and molecular phylogenies could also plausibly stem from extreme heterogeneity in genomic evolutionary rates across the most deeply divergent vertebrate lineages. The earliest-diverging actinopterygian and sarcopterygian clades (lungfishes, coelacanths, gars, bowfins, sturgeons, and polypterids) are often considered to be 'living fossils,' due to their low species diversity, ancient (>250 million year) common ancestry with other vertebrates, and the observation that living species show few morphological differences from extinct relatives that lived tens of millions of years in the past (Darwin 1859; Stanley 1975; Schultze and Wiley 1984; Grande and Bemis 1991, 1998; Grande 2010; Hilton et al. 2011; Amemiya et al. 2013; Casane and Laurenti 2013; Braasch et al. 2016; Clarke et al. 2016; Brito et al. 2017; Giles et al. 2017; Lidgard and Love 2018; Cavin et al. 2021; Meyer et al. 2021; Brownstein et al. 2022, 2024a; Cui et al. 2022; Hilton and Grande 2023).

**Figure 5.**
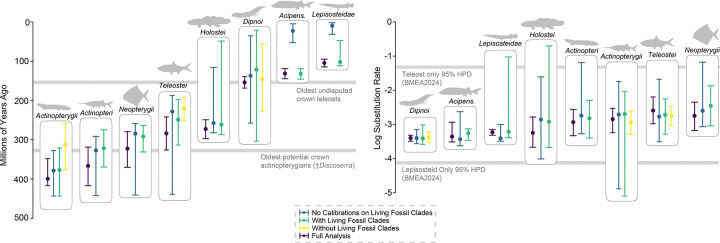
Variation in Genomic Evolution Renders Some Divergence Times Unknowable. Bowtie plot shows the median (dots) and 95% highest posterior density intervals (bars) for selected early-diverging vertebrate clades inferred across the taxon and fossil calibration inclusion experiments and the main tree. Silhouettes are public domain from phylopic.org or by CDB.

Several early diverging ray-finned fish clades, including gars and sturgeons, exhibit the slowest rates of nucleotide substitution among vertebrates (Braasch et al. 2016; Takezaki 2018; Du et al. 2020; Brownstein et al. 2024a), a phenomenon that may be linked to their capacity for successful hybridization between lineages separated by over 100 million years of common ancestry (Havelka et al. 2011; Bohn et al. 2017; Káldy et al. 2020; Taylor et al. 2020; Brownstein et al. 2024b). The juxtaposition of these exceptionally slow evolutionary rates with the accelerated rates characteristic of teleost fishes (Takezaki 2018; Brownstein et al. 2024a) can confound molecular clock models, leading to systematic errors in divergence time estimation (Dornburg et al. 2012). We investigated the impact of extreme molecular rate variation on divergence time estimation by inferring time-calibrated phylogenies that alternatively included and excluded species from the ‘living fossil’ clades *Lepisosteidae* (gars), *Amia* (bowfins), and *Acipenseriformes* (sturgeons and paddlefishes), and which included these species but without fossil calibrations placed on the nodes corresponding to their crown clades. As is expected if rate accommodation induces spurious inference of the ages of crown ray-finned fishes and the oldest actinopterygian divergences, we found that excluding living fossils resulted in crown ages for *Actinopterygii* and *Teleostei* that, on average, are 20 to 70 million years younger than when living fossil clades of ray-finned fishes are included (Figure 5). We did not observe similar shifts in estimated divergence times for deeply-divergent sarcopterygian clades (e.g., lungfishes; (Figure 5), suggesting that errors related to rate accommodation are largely restricted to ray-finned fishes. This observation is further supported by our analysis of nucleotide substitution rates across time-calibrated phylogenies from both subsampled and complete datasets (Figure 4B; Figures S10–11).

Previous research using both this exon dataset (Brownstein et al. 2024a) and other sequence data (Braasch et al. 2016; Takezaki 2018; Du et al. 2020) has demonstrated that gars and sturgeons exhibit slow molecular evolutionary rates relative to other vertebrates across their genomes. The mechanisms accounting for these slow rates of molecular evolution remain unclear, and may involve the suppression of transposable element activity (Braasch et al. 2016) or enhanced DNA repair capacity (Brownstein et al. 2024a). These slow rates represent biological characteristics rather than analytical artifacts (Braasch et al. 2016; Takezaki 2018; Du et al. 2020; Brownstein et al. 2024a), and so the inclusion of more sequence data from these and other ray-finned fishes would not necessarily remedy the problem.

When rates of substitution and divergence times are jointly estimated across ray-finned fishes, we found that the inferred rates for these typically slow-evolving clades converge more closely with the higher teleost rates, and vice versa (Figure 5). As rate variance is accommodated during inference of the time tree, the successive divergences among the four major actinopterygian clades (*Teleostei, Holostei, Acipenseriformes,* and *Polypteridae*) are pushed backward in time (Figure 5; Figures S10-S11).

Further evidence that the extreme disparity in molecular evolutionary rates among the major clades of ray-finned fishes contributes to erroneous divergence time estimation comes from analyses where holosteans and acipenseriforms are included without corresponding fossil calibrations. Crown gars are known from complete skeletons starting in the Late Cretaceous, ~97.2 Ma, and many members of crown *Lepisosteidae* have been described from complete body fossils from the latest Mesozoic and early Cenozoic (Grande 2010; Brito et al. 2017; Brownstein 2022; Brownstein and Lyson 2022; Brownstein et al. 2023b). Similarly, crown group acipenseriforms and acipenserids are known from partial and complete skeletons from throughout the Cretaceous and Cenozoic (Grande and Bemis 1991; Hilton et al. 2011; Sato et al. 2018; Murray et al. 2020; Hilton et al. 2023; Murray et al. 2023; Brownstein 2023a; Hilton and Grande 2023). Yet, when fossil calibrations are not used for these clades, the median ages of crown gars and acipenseriforms shift far towards the Recent, between 9 and 23 million years ago (Figure 5). These age estimates directly contradict the fossil records of these clades and show that even relaxed clock models fail to accommodate the exceptionally slow rates of these living fossil lineages. The estimated ages of *Actinopterygii*, *Teleostei*, and the other major ray-finned fish divergences found in analyses in which living fossil clades are included without corresponding fossil calibrations are also similar to those estimated when the living fossil crown clades are calibrated using fossils, suggesting that the disparity between the exceptionally fast teleost rate and slow holostean and acipenseriform molecular evolutionary rates drives the overestimation of ray-finned fish divergences as the relaxed clock model attempts to accommodate these rate differences by pushing the estimated teleost crown age and successive ray-finned fish divergences farther back in time (Figure 5).

## Discussion.

Here, we use a time-calibrated phylogeny of jawed vertebrates to investigate how a phylogeny of a species-rich lineage can be distorted by macroevolutionary events and intrinsic aspects of organismal molecular biology. Our analyses tie rapid diversifications of both terrestrial and marine gnathostome lineages to macroevolutionary events, such as the Cretaceous Terrestrial Revolution (Lloyd et al. 2008; Meredith et al. 2011; Weaver et al. 2024) and the Cretaceous-Paleogene Mass Extinction (Hackett et al. 2008; Jarvis et al. 2014; Prum et al. 2015; Feng et al. 2017; Alfaro et al. 2018; Burbrink et al. 2020; Kuhl et al. 2021; Ghezelayagh et al. 2022; Foley et al. 2023; Berv et al. 2024; Stiller et al. 2024). Phylogenetic signal within genes and sequence sites plunges at the K-Pg boundary (Figure 3), supporting the hypothesis that extinctions leave a lasting mark on evolutionary history by subjecting lineages to both major bottlenecks and sudden ecological opportunity as ecosystems reassemble (Gavrilets and Losos 2009; Prum et al. 2015; Stroud and Losos 2016; Reddy et al. 2017; Berv et al. 2024). The process of mass extinction and subsequent rapid radiation has sculpted the diversity of living jawed vertebrates across their phylogenetic and ecological diversity and resulted in some of the most famous evolutionary radiations, including birds, placental mammals, snakes, and pelagic predatory ray-finned fishes such as tunas, mackerels, jacks, and swordfishes (Figure 1). The intense diversification following the Cretaceous-Paleogene extinction manifests in our data as a distinct reduction in successive branching times among jawed vertebrates, centered at approximately 66 million years ago (Figure 3). Additionally, we observe anomaly zones – regions characterized by substantial conflict between gene trees and species trees – throughout the backbone phylogenies of these post-Cretaceous radiations (Figure 2C; Figure 3). Paradoxically, as much as genomes have provided consilience around jawed vertebrate relationships, these data also reveal the confounding effects of macroevolutionary events on inferring phylogenies.

Just as macroevolutionary events confound the resolution of jawed vertebrate phylogeny, the organismal biology of some jawed vertebrate clades hinders our ability to reconstruct their deep-time evolutionary history. Through experimental taxon and calibration exclusion experiments, we show that the inclusion of living representatives of *Acipenseriformes* and *Holostei*, which are two of the three major non-teleost lineages of ray-finned fishes and possess the slowest nucleotide substitution rates of all vertebrates (Braasch et al. 2016; Takezaki 2018; Du et al. 2020; Brownstein et al. 2024a), pushes the ages of *Teleostei* and *Actinopterygii* back in time by 29 to 65 million years (Figure 4). This temporal displacement helps explain a notable discrepancy in the fossil record. Although time-calibrated phylogenies built using molecular data suggest a Devonian-Carboniferous origin for the ray-finned fish crown group (Near et al. 2012; Betancur-R et al. 2013, 2017; Giles et al. 2017; Hughes et al. 2018), the earliest fossil evidence of major actinopterygian crown clades (including crown actinopterygians, crown neopterygians, and pan-acipenseriforms) appears 30 to 50 million years later (Argyriou et al. 2018, n.d.). This inconsistency suggests that molecular data alone cannot reliably determine the ages of *Actinopterygii* and its major internal divergences (*Actinopteri*, *Neopterygii*, and *Teleostei*). This result reemphasizes the importance of the fossil record for estimating the ages of this clades by providing minimum bounds.

The increasing availability of high-quality genomic data presents both opportunities and challenges in reconstructing the Tree of Life. Our phylogenetic analyses of 540 representative jawed vertebrates using genome-scale data corroborates several recently proposed relationships among ancient tetrapod, sarcopterygian, and ray-finned fishes (Figure 1)(Chakrabarty et al. 2017; Hughes et al. 2018; Thompson et al. 2021; Ghezelayagh et al. 2022; Melo et al. 2022; Foley et al. 2023; Parey et al. 2023), yet provide just as little clarity about others. Although comparisons of different types of genomic marker datasets show that deep-branching relationships across the vertebrate tree of life largely remain stable regardless of marker type (e.g., UCEs, exons, AHEs) used (Reddy et al. 2017; Alda et al. 2021; Arcila et al. 2021), analyses incorporating thousands loci still fail to unambiguously resolve the relationships of lineages in rapid radiations such as neoavian birds (Stiller et al. 2024) and placental mammals (Foley et al. 2023). Our phylogenetic analyses similarly fail to resolve neoavian, acanthomorph and laurasiatherian mammal relationships, which are largely unresolved in our multispecies coalescent analyses owing to intense gene tree discordance (Figure 2, Figure S2, Figure S3). To this end, the phylogeny that we infer cannot be considered a robust hypothesis of the relationships of these lineages and other rapidly diversifying clades.

However, our results are notable for facilitating direct comparisons of levels of support for particular relationships across unrelated vertebrate lineages. For example, our analyses demonstrate that the monophyly of recently proposed ray-finned fish lineages *Oseanacephala* and *Stomiati* receive as much support from the same genome-wide dataset as the monophyly of placental and marsupial mammals, squamates, and archosaurs (Figure 2). Conversely, our results are the first direct comparison of uncertainty surrounding the relationships of unrelated order-level clades of jawed vertebrates classically considered major post-Cretaceous radiations; although gene tree uncertainty for neoavian ordinal relationships far surpasses that in acanthomorph and otophysan fishes, placental mammals, and anguimorph lizards, for example, bootstrap support and site concordance factors are comparable across these radiations (Figure S7). Although we only use one marker set comprised of exons, which are expected to be under strong selection, the ages of rapid radiations that are poorly resolved in our phylogeny, especially birds, placental mammals, acanthomorph fishes, are closely comparable to those estimated in previous studies (Meredith et al. 2011; dos Reis et al. 2012, 2014; Jarvis et al. 2014; Prum et al. 2015; Hughes et al. 2018; Kuhl et al. 2021; Ghezelayagh et al. 2022; Álvarez-Carretero et al. 2022; Foley et al. 2023; Berv et al. 2024; Stiller et al. 2024). It is especially exciting to us that despite the obvious lack of resolution within *Neoaves* in our analyses, our time-calibrated phylogeny still places the rapid diversification of this lineage right at the K-Pg boundary (Chen et al. 2025), which has been found using genomic datasets containing different marker types, including exons (Jarvis et al. 2014), anchored hybrid enrichment loci (Prum et al. 2015; Berv et al. 2024), transcriptomes (Kuhl et al. 2021), and combinations of different marker sets (Stiller et al. 2024). Similarly, the middle Cretaceous age we infer for most divergences among placental mammal orders matches those found in phylogenomic analyses using different data types (Meredith et al. 2011; dos Reis et al. 2012, 2014; Álvarez-Carretero et al. 2022; Foley et al. 2023). Thus, our inference of the timing of these radiations cannot be attributed to data type, and the severity of phylogenetic discordance is indeed comparable across multiple distantly related jawed vertebrate lineages that diversified in response to the K-Pg mass extinction.

Our estimates of the ages of major jawed vertebrate clades are also affected by lineage-specific aspects of the genome, and most obviously by rates of sequence evolution (Figure 5). A striking example appears in ray-finned fishes, where substitution rate variation among the four earliest diverging lineages likely results in age overestimates of up to 70 million years for both the actinopterygian and teleost crown groups (Figure 5). These results challenge the current paradigm of phylogenetic reconstruction by demonstrating the limits of phylogenetic inference against the realities of confounding macroevolutionary events and organismal biology. Indeed, it is our inability to achieve complete phylogenetic resolution that illuminates macroevolutionary dynamics and lineage-specific patterns of genome evolution across more than 500 million years of jawed vertebrate evolution.

## Supplementary Material

Supplement 1

## Data Availability

All data needed to replicate the results in this study are provided in the Supplementary Information or on the associated Yale Dataverse Repository (https://doi.org/10.60600/YU/YL2LSZ). All sequences come from genomic data available on the NCBI sequence read archive or Hughes et al. (Hughes et al. 2018), and are feature in Table S1.
