# Supplementary material for: Genomic Distortion of Jawed Vertebrate Phylogeny
Source: bioRxiv. 2026 Jun 29:2026.06.28.735080. Preprint. [Version 1] doi: 10.64898/2026.06.28.735080 (PMC13345097; doi:10.64898/2026.06.28.735080)
Supplement: Supplement 1 [file NIHPP2026.06.28.735080v1-supplement-1.pdf]

bioRxiv preprint doi: https://doi.org/10.64898/2026.06.28.735080; this version posted June 29, 2026. The copyright holder for this preprint (which was not certified by peer review) is the author/funder, who has granted bioRxiv a license to display the preprint in perpetuity. It is made available under a CC-BY 4.0 International license.

Supplement To: Genomic Distortion of Jawed Vertebrate Phylogeny in Space and Time

Chase D. Brownstein, [1*] Liandong Yang,[2] Thomas J. Near[1, 3]

[1]Department of Ecology and Evolutionary Biology, Yale University, New Haven CT, USA

[2]Institute of Hydrobiology, Chinese Academy of Sciences, Beijing, China

[3]Peabody Museum, Yale University, CT, USA

*Corresponding author

Email: chase.brownstein@yale.edu

Table of Contents.

I. **List of Fossil Calibrations.**

II. **Supplementary Results and Discussion.**

III. **Supplementary References.**

IV. **Supplementary Tables.**

I. List of Fossil Calibrations.

†*Phoebodus saidselachus*

Justification of Placement: †*Phoebodus saidselachus* calibrates the most recent common ancestor (MRCA) of crown *Chondrichthyes* (specifiers: *Callorhinchus milii*, *Carcharodon carcharias*) in our node-dating analyses. The placement of †*P. saidselachus* within crown *Chondrichthyes* on the stem of *Elasmobranchii* (sharks, skates and rays) is supported by parsimony and Bayesian phylogenetic analyses of morphological characters, including the 221 character, 60 taxon matrix of Frey et al. (Frey et al. 2019), the 230 character, 64 taxon matrix of Frey et al. (Frey et al. 2020; Klug et al. 2023), and the 236 character, 36 taxon matrix of Brownstein et al. (Brownstein, Near, et al. 2024). All of these matrices are modified from the matrix of Coates et al. (Coates et al. 2018). For the purposes of this justification, we rely on the phylogeny presented in figure 4 in Frey et al. (Frey et al. 2019) as the reference tree. Phylogenetically optimized apomorphies uniting †*P. saidselachus* with Pan-*Elasmobranchii* are: smooth ceratohyal has a posterior, lateral fossa (character 51:1 in Frey et al. (Frey et al. 2019)), dorsal otic ridge forms a crest posteriorly (156:1 in Frey et al. (Frey et al. 2019)), slot-shaped endolymphatic fossa divides dorsal otic ridge along midline (158:1 in Frey et al. (Frey et al.

bioRxiv preprint doi: https://doi.org/10.64898/2026.06.28.735080; this version posted June 29, 2026. The copyright holder for this preprint (which was not certified by peer review) is the author/funder, who has granted bioRxiv a license to display the preprint in perpetuity. It is made available under a CC-BY 4.0 International license.

2019)), dorsal portion of occipital arch wedged between otic capsules (171:1 in Frey et al. (Frey et al. 2019)), posterior/pelvic-level dorsal fin possesses calcified base plate (200:1 in Frey et al. (Frey et al. 2019)).

Stratigraphic Horizon: Madene El Mrakib and Aguelmous, Ibâouane Formation (Lahfira Member, Thylacocephalan Layer)(Frey et al. 2020), southern Maïder region, eastern Anti-Atlas, Morocco; Middle Famennian Stage of the Devonian (Frey et al. 2019), 372.2 to 359.3-358.9 Ma (Becker et al. 2012; Gradstein et al. 2021).

Equivalent Fossil Calibrations: †*Ferromirum oukherbouchi* and †*Maghriboselache mohamezanei* are chondrichthyans known from whole-body fossils from the same stratigraphic horizon that are unambiguously placed as stem-holocephalans in crown *Chondrichthyes* in parsimony and Bayesian phylogenetic analyses of morphological characters (Frey et al. 2020; Klug et al. 2023; Brownstein, Near, et al. 2024).

Fossil tip age: 358.9 Ma. The node calibration was created such that 97.5% of the probability distribution fell before 358.9 Ma.

†*Guiyu oneiros*

Justification of Placement: †*Guiyu oneiros* calibrates the MRCA of crown *Osteichthyes* (specifiers: *Homo sapiens*, *Danio rerio*) in our node-dating analyses. The placement of †*Guiyu oneiros* within crown *Osteichthyes* is supported by multiple parsimony and Bayesian phylogenetic analyses of morphological characters, including the 153 character, 23 taxon matrix of Zhu et al. (Zhu et al. 2009), the 236 character, 78 taxon matrix of Giles et al.(Giles, Friedman, et al. 2015), the 182 character, 49 taxon matrix of Giles et al.(Giles, Darras, et al. 2015), the 269 character, 96 taxon matrix of Lu et al. (Lu, Giles, et al. 2016), the 335 character, 103 taxon matrix of Qiao et al. (Qiao et al. 2016), the 336 character, 104 taxon matrix of Choo et al. (Choo et al. 2017), the 347 character, 104 taxon matrix of Cui et al. (Cui et al. 2019), and the 694 character, 159 taxon matrix of Cui et al. (Cui et al. 2023). Notably, Lu et al. (Lu et al. 2017), King et al. (King et al. 2017), and Brazeau et al. (Brazeau et al. 2020) placed †*G. oneiros* within a clade of Devonian osteichthyans that is the sister lineage to crown *Osteichthyes*. However, analyses of an expanded version of the King et al. (King et al. 2017), and Brazeau et al.(Brazeau et al. 2020) matrices either place this clade in a polytomy with crown osteichthyans or on the sarcopterygian stem under a Bayesian tip-dating approach (Chase Doran Brownstein 2023). For

bioRxiv preprint doi: https://doi.org/10.64898/2026.06.28.735080; this version posted June 29, 2026. The copyright holder for this preprint (which was not certified by peer review) is the author/funder, who has granted bioRxiv a license to display the preprint in perpetuity. It is made available under a CC-BY 4.0 International license.

the purposes of this justification, we rely on the phylogeny presented in supplementary figure 6 in Cui et al. (Cui et al. 2023) Because no character state optimizations were provided in Cui et al. (Cui et al. 2023), we reran the matrix presented in the paper using TNT v 1.5 (Goloboff and Catalano 2016) to optimize apomorphies; we ran an initial Wagner search over 1000 replicates and default parameters for ratchet, tfuse, drift, and sectorial search followed by traditional bisection reconnection branch swapping over 100,000 trees. We summarized the topologies in a strict consensus and then recorded apomorphies of two nodes: the clade containing †*G. oneiros* and crown *Osteichthyes* and †*Guiyu oneiros* and Pan-*Sarcopterygii*. Phylogenetically optimized apomorphies uniting †*G. oneiros* with crown *Osteichthyes* are: presence of differentiated lepidotrichia (28:1 in Cui et al. (Cui et al. 2023)), posterior end of supraorbital canal contained in postparietal (178:0 in Cui et al. (Cui et al. 2023)), and posterior expansion of the maxilla (270:0 in Cui et al. (Cui et al. 2023)). Phylogenetically optimized apomorphies uniting †*G. oneiros* with Pan-*Sarcopterygii* are: dermal cranial joint at the level of sphenoid-otic junction (111:1 in Cui et al. (Cui et al. 2023)), presence of the supraorbital (125:1 in Cui et al. (Cui et al. 2023)), presence of an endoskeletal intracranial joint (371:1 in Cui et al. (Cui et al. 2023)), internasal vacuities shallow, paired pits with strong midline ridge (390:1 in Cui et al. (Cui et al. 2023)), and presence of an unconstricted cranial notochord (411:1 in Cui et al. (Cui et al. 2023)).

Stratigraphic Horizon: Qujing, Kuanti Formation, Yunnan Province, China; late Ludfordian Stage of the Silurian (Zhu et al. 2013). The Ludfordian Stage ranges between 425.6 and 423.0 Ma (Gradstein et al. 2021).

Equivalent Fossil Calibrations: None. Slightly younger calibrations of the same node include the 'psarolepids' †*Achoania jarvikii* (Zhu et al. 2001) and †*Psarolepis romeri* (Qu et al. 2013), as well as the stem actinopterygian †*Meemania eos* (Lu, Giles, et al. 2016). However, these taxa are younger than the age of the oldest stem-lungfishes (see below)(Cui et al. 2022) and are therefore redundant for fossil calibration.

Fossil tip age: 423.0 Ma. The node calibration was created such that 97.5% of the probability distribution fell before 423.0 Ma.

†*Youngolepis praecursor*

Justification of Placement: †*Youngolepis praecursor* calibrates the MRCA of *Dipnoi* and *Latimeria* (specifiers: *Neoceratodus forsteri*, *Latimeria chalumnae*) in our node dating analyses.

bioRxiv preprint doi: https://doi.org/10.64898/2026.06.28.735080; this version posted June 29, 2026. The copyright holder for this preprint (which was not certified by peer review) is the author/funder, who has granted bioRxiv a license to display the preprint in perpetuity. It is made available under a CC-BY 4.0 International license.

The placement of †*Y. praecursor* on the stem of *Dipnoi* is supported by parsimony and Bayesian phylogenetic analyses of morphological characters, including the 335 character, 103 taxon matrix of Qiao et al. (Qiao et al. 2016), the 242 character, 37 taxon matrix of Lu et al. (Lu, Zhu, et al. 2016), the 70 character, 25 taxon matrix of Friedman (FRIEDMAN 2007), and the 277 character, 88 taxon matrix of Cui et al. (Cui et al. 2022). For the purposes of this justification, we rely on the phylogenies presented in supplementary figure 2 in Cui et al.(Cui et al. 2022) Because no character state optimizations were provided by Cui et al. (Cui et al. 2022) for the placement of †*Y. praecursor* sister to all other members of Pan-*Dipnoi*, we reran the matrix presented in the paper using TNT v 1.5 (Goloboff and Catalano 2016) to optimize apomorphies; we ran an initial Wagner search over 1000 replicates and default parameters for ratchet, tfuse, drift, and sectorial search followed by traditional bisection reconnection branch swapping over 100,000 trees. We summarized the topologies in a strict consensus and then recorded apomorphies of †*Y. praecursor*+Pan-*Dipnoi*. Phylogenetically optimized apomorphies uniting †*Y. praecursor* with Pan-*Dipnoi* are: closed pineal opening (1:1 in Cui et al. (Cui et al. 2022)), quadratojugal as large as jugal (34:0 in Cui et al. (Cui et al. 2022)), adductor fossa more than 1/3 length of jaw (133:0 in Cui et al. (Cui et al. 2022)), addition of large dentine elements at regular intervals to lateral margin of pterygoid and/or prearticular (166:0 in Cui et al. (Cui et al. 2022)), dentine sheet on resorption areas within tooth plate on palate (174:1 in Cui et al. (Cui et al. 2022)), marginal tooth ridge continuous (185:1 in Cui et al. (Cui et al. 2022)), intracranial joint present as a ventral cranial fissure (190:1 in Cui et al. (Cui et al. 2022)), and a triangular hyomandibular (271:1 in Cui et al. (Cui et al. 2022)).

Stratigraphic Horizon: Yunnan, China; Xitun Formation, earliest Lochkovian Stage of the Devonian (Zhao et al. 2021; Cui et al. 2022). We followed Cui et al. (Cui et al. 2022) as treating its age as 418.0 Ma.

Equivalent Fossil Calibrations: †*Diabolepis speratus* is known from the same formation and is unambiguously placed as a stem-dipnoan crownward of †*Youngolepis praecursor* in the phylogeny of Cui et al. (Cui et al. 2022), as well as a pan-dipnoan in other phylogenetic analyses (Qiao et al. 2016; Kemp et al. 2017; Brazeau et al. 2020; Cloutier et al. 2020; Zhu et al. 2021).

Fossil tip age: 418.0 Ma. The node calibration was created such that 97.5% of the probability distribution fell before 418.0 Ma.

bioRxiv preprint doi: https://doi.org/10.64898/2026.06.28.735080; this version posted June 29, 2026. The copyright holder for this preprint (which was not certified by peer review) is the author/funder, who has granted bioRxiv a license to display the preprint in perpetuity. It is made available under a CC-BY 4.0 International license.

†*Ferganoceratodus martini*

Justification of Placement: †*Ferganoceratodus martini* calibrates the MRCA of *Neoceratodus forsteri* and *Protopterus annectens* in our node-dating analyses. The placement of †*F. martini* in crown *Dipnoi* is supported by parsimony-based phylogenetic analysis of morphological characters, including the 14 character, 16 taxon matrix of Cavin et al. (CAVIN et al. 2007) We use the tree supplied in figure 18 of Cavin et al. (CAVIN et al. 2007) as the reference phylogeny for this placement. Because not all character state optimizations were provided by Cavin et al. (CAVIN et al. 2007) for the placement of †*F. martini* in *Dipnoi*, we reran the matrix presented in the paper using TNT v 1.5 (Goloboff and Catalano 2016) to optimize apomorphies; we ran an initial Wagner search over 1000 replicates and default parameters for ratchet, tfuse, drift, and sectorial search followed by traditional bisection reconnection branch swapping over 100,000 trees. †*Dipterus valenciennesi* was used as the outgroup. We summarized the topologies in a strict consensus and then recorded apomorphies of crown *Dipnoi* and Pan-*Lepidosirenidae* (*Lepidosiren* + *Protopterus* total clade). Phylogenetically optimized apomorphies that †*F. martini* shares with the dipnoan crown clade are: median series with 3 bones or bone pairs (1:1 in Cavin et al. (CAVIN et al. 2007)), and mediolateral series with four or more bones (3:0 in Cavin et al. (CAVIN et al. 2007)). Phylogenetically optimized apomorphies that †*F. martini* shares with Pan-*Lepidosirenidae* are: skull roof anteriorly straight, with a short rostrum without a supraorbital sensory canal (5:2 in Cavin et al. (CAVIN et al. 2007)).

Stratigraphic Horizon: Phu Nam Jun, Kalasin Province, Thailand; Upper Phu Kradung Formation, Tithonian Stage of the Late Jurassic to Berriasian Stage of the Early Cretaceous (CAVIN et al. 2007; Kemp et al. 2017).

Equivalent Fossil Calibrations: Numerous extinct lungfishes from as old as the Permian and Triassic have been placed within the lungfish crown clade in phylogenetic analyses of morphological characters (CAVIN et al. 2007; Kemp et al. 2017). Our use of †*Ferganoceratodus martini* provides a fairly conservative young age (earliest Cretaceous) for the prior on the crown lungfish divergence.

Fossil tip age: 140.2 Ma. The node calibration was created such that 97.5% of the probability distribution fell before 140.2 Ma.

†*Triadobatrachus massinoti*

bioRxiv preprint doi: https://doi.org/10.64898/2026.06.28.735080; this version posted June 29, 2026. The copyright holder for this preprint (which was not certified by peer review) is the author/funder, who has granted bioRxiv a license to display the preprint in perpetuity. It is made available under a CC-BY 4.0 International license.

Justification of Placement: †*Triadobatrachus massinoti* calibrates the MRCA of crown *Batrachia* (specifiers: *Ambystoma mexicanum*, *Xenopus tropicalis*) in our node-dating analyses. The placement of †*T. massinoti* in crown *Amphibia* on the anuran stem is supported by multiple parsimony and Bayesian phylogenetic analyses of morphological characters, including the 360 character, 62 taxon matrix of Schoch et al. (Schoch et al. 2020) the 308 character, 53 taxon matrix of Jones et al. (Jones et al. 2022), and the 355 character, 63 taxon matrix of Kligman et al. (Kligman et al. 2023). For the purposes of this justification, we rely on the phylogeny presented in Kligman et al. (Kligman et al. 2023) Because no character state optimizations were provided by Kligman et al. (Kligman et al. 2023) for the placement of †*T. massinoti* in crown *Batrachia* sister to *Anura*, we reran the matrix presented in the paper using TNT v 1.5(Goloboff and Catalano 2016) to optimize apomorphies; we ran an initial Wagner search over 1000 replicates and default parameters for ratchet, tfuse, drift, and sectorial search followed by traditional bisection reconnection branch swapping over 100,000 trees. We summarized the topologies in a strict consensus and then recorded apomorphies of three nodes: crown *Amphibia*, crown *Batrachia*, and the clade in Pan-*Anura* uniting †*T. massinoti* with crown frogs. Phylogenetically optimized apomorphies uniting †*T. massinoti* with crown *Amphibia* are: weak torsion of the humerus (191:1 in Kligman et al. (Kligman et al. 2023)), absence of the postorbital (226:1 in Kligman et al. (Kligman et al. 2023)), tabular absent (234:1 in Kligman et al. (Kligman et al. 2023)), lateral process of pterygoid contribution to posttemporal fossa absent (253:0 in Kligman et al. (Kligman et al. 2023)), trunk intercentra absent (272:1 in Kligman et al. (Kligman et al. 2023)), ectopterygoid absent (340:1 in Kligman et al. (Kligman et al. 2023)), interclavicle absent (341:1 in Kligman et al. (Kligman et al. 2023)), cleithrum absent (342:1 in Kligman et al. (Kligman et al. 2023)), and vomer irregularly shaped, with a posteromedially expanded choana (343:1 in Kligman et al. (Kligman et al. 2023)). Phylogenetically optimized apomorphies uniting †*T. massinoti* with crown *Batrachia* are: nasal as wide as long (15:1 in Kligman et al. (Kligman et al. 2023)), orbit and naris separated only by a narrow gap of bone (24:1 in Kligman et al.(Kligman et al. 2023)), vertebral transverse processes distally extended with laterally directed diapophyses (156:1* in Kligman et al. (Kligman et al. 2023); this character is coded as states 0 and 1 in †*T. massinoti*), ilium shaft slender and elongated (194:1 in Kligman et al. (Kligman et al. 2023)), internal trochanter of femur reduced to a short crest (201:1 in Kligman et al. (Kligman et al. 2023)), ossified hyoids present (270:0 in Kligman et al. (Kligman et al. 2023)), skull table

bioRxiv preprint doi: https://doi.org/10.64898/2026.06.28.735080; this version posted June 29, 2026. The copyright holder for this preprint (which was not certified by peer review) is the author/funder, who has granted bioRxiv a license to display the preprint in perpetuity. It is made available under a CC-BY 4.0 International license.

T-shaped (307:1 in Kligman et al. (Kligman et al. 2023)), footplate of stapes much smaller than the fenestra vestibule (332:1 in Kligman et al. (Kligman et al. 2023)), and the presence of the batrachian operculum (350:1in Kligman et al. (Kligman et al. 2023)). Phylogenetically optimized apomorphies uniting †*T. massinoti* with Pan-*Anura* are: internarial distance wider than interorbital distance (14:1 in Kligman et al. (Kligman et al. 2023)), otic notch appears as a semicircular embayment between squamosal and posterior skull table (50:0 in Kligman et al. (Kligman et al. 2023)), main axis of ilium shaft inclined anteriorly (197:2 Kligman et al. (Kligman et al. 2023)), parahyoid present (271:1in Kligman et al. (Kligman et al. 2023)), single notochordal atlas centrum (283:1 in Kligman et al. (Kligman et al. 2023)), elongated tibiale and fibulare present (302:1 in Kligman et al. (Kligman et al. 2023)), and absence of a canal for palatal ramus of the facial nerve in antotic region (331:0 in Kligman et al. (Kligman et al. 2023)).

Stratigraphic Horizon: There is considerable uncertainty surrounding the provenance of the only known fossil of †*Triadobatrachus massinoti,* which was recovered from the Sakamena Group of Madagascar (Ascarrunz et al. 2016). Many analyses have used an uppermost Early Triassic age for this taxon (Ascarrunz et al. 2016; Jones et al. 2022), primarily based on observations of vertebrate biostratigraphy at the type locality (Ascarrunz et al. 2016). A younger age (Olenekian, 249.9-246.7 Ma) was suggested on the basis of invertebrate faunas from other localities corresponding to the Triassic Sakamena Group in Madagascar (Ascarrunz et al. 2016). The youngest possible age of this fossil would be Middle Triassic, which is the younger bound on the Sakamena Group itself (Wescott and Diggens 1998). As such, we set the age of this fossil to this minimum bound, 237.0 Ma (Gradstein et al. 2021). In addition to representing a conservative interpretation of the age of this fossil, a Middle Triassic age of †*T. massinoti* allows for recognition of several equivalent fossil calibrations (see below), which provides a robust minimum age for the frog-salamander divergence.

Equivalent Fossil Calibrations: †*Triassurus sixtelae*, a member of Pan-*Caudata* known from partial articulated skeletons from the Ladinian-Carnian Madygen Formation of Kyrgyzstan (Schoch et al. 2020; Jones et al. 2022; Kligman et al. 2023), and †*Czatkobatrachus polonicus*, a possible member of Pan-*Anura* known from hypodigms of isolated bones from the fissure fills of the Early-Middle Triassic of Poland (Evans and Borsuk-Bialynicka 1998), are equivalent fossil calibrations. We did not use †*Czatkobatrachus polonicus* because of conceptual issues with the

bioRxiv preprint doi: https://doi.org/10.64898/2026.06.28.735080; this version posted June 29, 2026. The copyright holder for this preprint (which was not certified by peer review) is the author/funder, who has granted bioRxiv a license to display the preprint in perpetuity. It is made available under a CC-BY 4.0 International license.

use of isolated bone hypodigms as fossil calibrations (Caldwell et al. 2015; Chase D. Brownstein et al. 2023).

Fossil tip age: 237.0 Ma. The node calibration was created such that 97.5% of the probability distribution fell before 237.0 Ma.

†*Rhadinosteus parvus*

Justification of Placement: †*Rhadinosteus parvus* calibrates the MRCA of crown *Pipanura* (specifiers: *Rana catesbeiana*, *Xenopus tropicalis*) in our node-dating analyses. The placement of †*R. parvus* along either the stem or within crown *Pipoidea* is supported by multiple parsimony phylogenetic analyses of morphological characters, including the 165 character, 36 taxon matrix of Gómez (Gómez 2016), the 18 character, 13 taxon matrix of Henrici (Henrici 1998), and the 72 character, 24 taxon matrix of Báez (Báez 2013) For the purposes of this justification, we rely on the phylogeny presented in Gómez (Gómez 2016). Because no character state optimizations were provided by Gómez (Gómez 2016) for the placement of †*R. parvus* in crown *Anura* sister to *Pipoidea*, we reran the matrix presented in the paper using TNT v 1.5 (Goloboff and Catalano 2016) to optimize apomorphies; we ran an initial Wagner search over 1000 replicates and default parameters for ratchet, tfuse, drift, and sectorial search followed by traditional bisection reconnection branch swapping over 100,000 trees. We summarized the topologies in a strict consensus and then recorded apomorphies of two nodes: crown *Pipanura* and the clade in Pan-*Pipoidea* including †*R. parvus.* Phylogenetically optimized apomorphies uniting †*R. parvus* with crown *Pipanura* are: scapula, ratio between maximum width of glenoid fossa and maximum width of shaft between 1.0 and 0.5 (109:1 in Gómez (Gómez 2016)), tibiale-fibulare less than 20% of the length of the hindlimb. Phylogenetically optimized apomorphies uniting †*R. parvus* with Pan-*Pipoidea* are: complete fusion of the frontoparietals (4:1 in Gómez (Gómez 2016)), absence of the subotic alae of the parasphenoid (17:1 in Gómez (Gómez 2016)), and anterior third of the cultriform process of the parasphenoid very narrow, such that the process is strongly tapering and pointed (19:2 in Gómez (Gómez 2016)).

Stratigraphic Horizon: Dinosaur National Monument (DNM) locality 96, Utah; Brushy Basin Member, Morrison Formation (Henrici 1998). The Brushy Basin Member of the Morrison Formation at DNM is considered to be form the Kimmeridgian to Tithonian Stages of the Late Jurassic (Henrici 1998); $^{40}Ar/^{39}Ar$ dating places this unit between 151 and 145 Ma (Kowallis et

bioRxiv preprint doi: https://doi.org/10.64898/2026.06.28.735080; this version posted June 29, 2026. The copyright holder for this preprint (which was not certified by peer review) is the author/funder, who has granted bioRxiv a license to display the preprint in perpetuity. It is made available under a CC-BY 4.0 International license.

al. 1991; Trujillo and Kowallis 2015). The dinosaur quarry at DNM has been dated to between 150.91 and 150.04 Ma using magnetostratigraphy (Maidment et al. 2017). Following previous work on Morrison Formation herpetofauna (Brownstein et al. 2022; Meyer et al. 2023), we use an age of 145.0 Ma.

Equivalent Fossil Calibrations: None.

Fossil tip age: 145.0 Ma. The node calibration was created such that 97.5% of the probability distribution fell before 145.0 Ma.

†*Henkelotherium guimarotae*

Justification of Placement: †*Henkelotherium guimarotae* calibrates the MRCA of crown *Mammalia* (specifiers: *Homo sapiens*, *Ornithorhynchus anatinus*) in our node-dating analyses. The placement of †*H. guimarotae* as a crown mammal on the stem of *Theria* (Pan-*Marsupialia* + Pan-*Placentalia*) is supported by multiple parsimony and Bayesian phylogenetic analyses of morphological characters, including the 4541 character, 126 taxon matrix of O'Leary et al. (O'Leary et al. 2013) (including as updated by Velazco et al. (Velazco et al. 2022)), the 491 character, 112 taxon matrix of Luo et al. (Luo, Meng, et al. 2015), the Luo et al., 497 character, 114 taxon matrix of Luo et al. (Luo, Gatesy, et al. 2015), the 505 character, 117 taxon matrix of Han et al. (Han et al. 2017), the 538 character, 125 taxon matrix of Huttenlocker et al. (Huttenlocker et al. 2018), the 530 character, 84 taxon matrix of Krause et al. (Krause et al. 2020) (including as modified by Hoffman et al. (Hoffmann et al. 2020)), the 615 character, 135 taxon matrix of Wang and Wang (Wang and Wang 2023). Notably, the position of †*H. guimarotae*

within crown *Mammalia* is unchanged regardless of the placement of problematic †*Haramiyida* and †*Gondwanatheria* among near-mammal mammaliaforms (Luo, Gatesy, et al. 2015; Han et al. 2017; Huttenlocker et al. 2018; Hoffmann et al. 2020; Krause et al. 2020; Wang and Wang 2023). For the purposes of this justification, we rely on the phylogenies presented in figure 1 of Hoffman et al. (Hoffmann et al. 2020). Because no character state optimizations were provided by Hoffman et al. (Hoffmann et al. 2020) for the placement of †*H. guimarotae* in crown *Mammalia*, we reran the matrix presented in the paper using TNT v 1.5 (Goloboff and Catalano 2016) to optimize apomorphies; we ran an initial Wagner search over 1000 replicates and default parameters for ratchet, tfuse, drift, and sectorial search followed by traditional bisection

bioRxiv preprint doi: https://doi.org/10.64898/2026.06.28.735080; this version posted June 29, 2026. The copyright holder for this preprint (which was not certified by peer review) is the author/funder, who has granted bioRxiv a license to display the preprint in perpetuity. It is made available under a CC-BY 4.0 International license.

reconnection branch swapping over 100,000 trees. We summarized the topologies in a strict consensus and then recorded apomorphies of two nodes: crown *Mammalia* and *Theria*+†*Dryolestidae* (incl. †*H. guimarotae*). Phylogenetically optimized apomorphies uniting †*H. guimarotae* with crown *Mammalia* are: lumbar ribs of dorsal vertebrae synostosed to vertebrae to form transverse processes (11:1 in Hoffman et al. (Hoffmann et al. 2020)), internal sutures of ilium, ischium, and pubis fused in adults (58:1 in Hoffman et al. (Hoffmann et al. 2020)), patellar groove of femur present (74:1 in Hoffman et al. (Hoffmann et al. 2020)), distinct medial malleolus of tibia (77:1 in Hoffman et al. (Hoffmann et al. 2020)), calcaneo-astragal facet of calcaneum placed medial to calcaneo-fibular facet (101:1 in Hoffman et al. (Hoffmann et al. 2020)), os calcaris present (111:1 in Hoffman et al. (Hoffmann et al. 2020)), dentary condyle of mandible positioned above the level of the postcanine alveoli (346:1 in Hoffman et al. (Hoffmann et al. 2020)), lower ultimate premolar (p5) hypoconid present (403:1 in Hoffman et al. (Hoffmann et al. 2020)), ml-2, protoconid far more bulging than paraconid and metaconid (432:1 in Hoffman et al. (Hoffmann et al. 2020)), ml-2, metacristid (protocristid) crest between protoconid (cusp a) and metaconid (cusp c) transversely oriented relative to long axis of lower molars (433:2 in Hoffman et al. (Hoffmann et al. 2020)), m1, main cusps of trigonid aligned at an acute angle (435:1 in Hoffman et al. (Hoffmann et al. 2020)), ml-2, primary functional cusps not labiolingually compressed (436:0 in Hoffman et al. (Hoffmann et al. 2020)), m1-2, lingual root present (506:1 in Hoffman et al. (Hoffmann et al. 2020)), and the presence of prevallum/postvallid shearing (522:1 in Hoffman et al.(Hoffmann et al. 2020)). Phylogenetically optimized apomorphies uniting †*H. guimarotae* and †*Dryolestidae* with *Theria* are: fusion of the neural hemiarches of the atlas (2:1 in Hoffman et al. (Hoffmann et al. 2020)), styloid process of radius present (45:1 in Hoffman et al. (Hoffmann et al. 2020)), styloid process of ulna present (50:1 in Hoffman et al. (Hoffmann et al. 2020)), femur neck distinct and long (64:1 in Hoffman et al. (Hoffmann et al. 2020)), lesser trochanter of femur positioned on ventromedial or ventral side of shaft (68:1 in Hoffman et al. (Hoffmann et al. 2020)), postpalatine torus of palate present (158:1 in Hoffman et al. (Hoffmann et al. 2020)), quadrate ramus of alisphenoid absent (208:2 in Hoffman et al. (Hoffmann et al. 2020)), absence of a diastema distal to lower canine (356:0 in Hoffman et al. (Hoffmann et al. 2020)), four lower incisors in each quadrant (357:1 in Hoffman et al. (Hoffmann et al. 2020)), four lower premolars in each quadrant (380:1 in Hoffman et al. (Hoffmann et al. 2020)),

bioRxiv preprint doi: https://doi.org/10.64898/2026.06.28.735080; this version posted June 29, 2026. The copyright holder for this preprint (which was not certified by peer review) is the author/funder, who has granted bioRxiv a license to display the preprint in perpetuity. It is made available under a CC-BY 4.0 International license.

lower ultimate premolar (p5) paraconid well-developed (401:1 in Hoffman et al. (Hoffmann et al. 2020)), interlocking distal postcanines/ml-2 present (425:1 in Hoffman et al. (Hoffmann et al. 2020)).

Stratigraphic Horizon: Guimarota coal mine, Portugal (Krebs 1991; Jäger et al. 2020; Luo and Martin 2023); Alcobaça Formation, Lower Kimmeridgian Stage of the Late Jurassic, 154.8-149.2 Ma (Gradstein et al. 2021).

Equivalent Fossil Calibrations: Highly dependent on phylogeny. Equivalent-age or older dryolestids and allotherians are known but occasionally placed outside the mammalian crown group. Refer to the cited phylogenetic analyses above. We chose †*Henkelotherium guimarotae* as the fossil calibration for crown mammals because it is reliably placed within the crown across multiple phylogenetic analyses and is known from a well-characterized cranium and postcranium, contrary to other mammals from the Jurassic with similar morphology (Krebs 1991; Jäger et al. 2020). However, if allotherians, and in particular haramiyidans, are crown group mammals, the age of crown *Mammalia* could be as old as the Triassic (see, for example, Huttenlocker et al. (Huttenlocker et al. 2018)).

Fossil tip age: 154.8 Ma. The node calibration was created such that 97.5% of the probability distribution fell before 154.8 Ma.

†*Deltatheridium pretrituberculare*

Justification of Placement: †*Deltatheridium pretrituberculare* calibrates the MRCA of crown *Marsupialia* (specifiers: *Monodelphis domestica*, *Macropus eugenii*) in our node-dating analyses. The placement of †*D. pretrituberculare* as a member of crown *Marsupialia* is supported by multiple parsimony and Bayesian phylogenetic analyses of morphological characters in a recent analysis incorporating new morphological data (Velazco et al. 2022). For the purposes of this justification, we rely on the phylogeny presented in figure 1 of Velazco et al. (Velazco et al. 2022). Phylogenetically optimized apomorphies uniting †*D. pretrituberculare* and crown *Theria* are: mandibular foramen positioned at or posterior to the midpoint of the ramus (1228:1 in Velazco et al. (Velazco et al. 2022)), pterygoid crest absent (1230:1 in Velazco et al. (Velazco et al. 2022)), Meckelian groove absent (1236:1 in Velazco et al. (Velazco et al. 2022)), Li2 spatulate in shape (1308:1 in Velazco et al. (Velazco et al. 2022)), Li3 spatulate in shape (1325:1 in Velazco et al. (Velazco et al. 2022)), upper canine Uc1 larger than lower canine Lc1

bioRxiv preprint doi: https://doi.org/10.64898/2026.06.28.735080; this version posted June 29, 2026. The copyright holder for this preprint (which was not certified by peer review) is the author/funder, who has granted bioRxiv a license to display the preprint in perpetuity. It is made available under a CC-BY 4.0 International license.

(1409:0 in Velazco et al. (Velazco et al. 2022)), talonid present on Lp2 (1492:1 in Velazco et al.(Velazco et al. 2022)), Lp3 absent (1499:1 in Velazco et al. (Velazco et al. 2022)), protoconid height on Lp4 subequal to height of p5 protoconid (1532:1 in Velazco et al. (Velazco et al. 2022)), degree of hypsodonty of UP1 VAR more that 1.0 (hypsodont, 1718:1 in Velazco et al. (Velazco et al. 2022)), UP2 crown with two or more clearly defined cusps (1735:1 in Velazco et al. (Velazco et al. 2022)), P4 height less than that of P5 but greater than half the height of P5 (1792:3 in Velazco et al. (Velazco et al. 2022)), UP4 crown with two or more clearly defined cusps (1794:1 in Velazco et al. (Velazco et al. 2022)), one premolar trenchant in penultimate premolar position (2024:1 in Velazco et al. (Velazco et al. 2022)), precingulid present on Lm1 (2042:1 in Velazco et al. (Velazco et al. 2022)), paraconid size on Lm1 moderate (approx. 75 percent the height of the protoconid; 2058:1 in Velazco et al. (Velazco et al. 2022)), protocristid transversely oriented relative to the longitudinal axis of Lm1 (2076:1 in Velazco et al. (Velazco et al. 2022)), cristid obliqua (=ectolophid) development on Lm1 very strong (2100:1 in Velazco et al. (Velazco et al. 2022)), precingulid present on Lm2 (2166:1 in Velazco et al. (Velazco et al. 2022)), entoconid in straight alignment with the paraconid and metaconid on Lm2 (2188:1 in Velazco et al. (Velazco et al. 2022)), protocristid transversely oriented relative to the longitudinal axis of Lm2 (2196:1 in Velazco et al. (Velazco et al. 2022)), hypoconulid buccally position to midline relative to the entoconid on Lm2 (2251:1 in Velazco et al. (Velazco et al. 2022)), 3 UM1 roots (2307:3 in Velazco et al. (Velazco et al. 2022)), M1 stylocone subequal to paracone (2344:1 in Velazco et al. (Velazco et al. 2022)), M1 preprotocrista on UM1 extends to the base of the paracone (2411:1 in Velazco et al. (Velazco et al. 2022)), area of UM3 relative to UM1 VAR very small (2582:0 in Velazco et al. (Velazco et al. 2022)), diastema present between UP1 and UP2 (2700:1 in Velazco et al. (Velazco et al. 2022)), supinator well-developed, extends proximal to the proximal opening of the entepicondylar foramen but does not approach the midshaft region (3033:1 in Velazco et al. (Velazco et al. 2022)), and unfused pubes (3312:0 in Velazco et al. (Velazco et al. 2022)).

Stratigraphic Horizon: Ukhaa Tolgod, Gobi Desert, Mongolia; Djadokhta Formation, precise age within the Campanian is controversial (Dingus et al. 2008), but we follow Velazco et al. (Velazco et al. 2022) in recognizing this age as early Campanian, 83.6 Ma (Gradstein et al. 2021).

bioRxiv preprint doi: https://doi.org/10.64898/2026.06.28.735080; this version posted June 29, 2026. The copyright holder for this preprint (which was not certified by peer review) is the author/funder, who has granted bioRxiv a license to display the preprint in perpetuity. It is made available under a CC-BY 4.0 International license.

Equivalent Fossil Calibrations: None. See Velazco et al. (Velazco et al. 2022). For the sensitivity analysis, †*Djarthia murgonensis*, a member of the total clade of *Australidelphia*, was used; this taxon is known from fossils dating to 54.6 Ma (Beck et al. 2008).

Fossil tip age: 83.6 Ma. The node calibration was created such that 97.5% of the probability distribution fell before 83.6 Ma.

†*Riostegotherium yanei*

Justification of Placement: †*Riostegotherium yanei* calibrates the MRCA of crown *Xenarthra* (specifiers: *Bradypus variegatus*, *Dasypus novemcinctus*) in our node-dating analyses. The placement of †*R. yanei* as a member of crown *Xenarthra* is supported by multiple morphological characters, including the presence of subrectangular osteoderms with characteristic microstructure (Anon 1998; Bergqvist et al. 2019). A phylogeny including this taxon is not yet available, but †*R. yanei* has been used to calibrate the xenarthran crown in multiple analyses (see Foley et al. (Foley et al. 2023)).

Stratigraphic Horizon: Fissure-fill deposits corresponding to the Itaboraian SALMA of the Rio de Janeiro State, Brazil (Bergqvist et al. 2019); we follow Foley et al. (Foley et al. 2023) in using a terminal Ypresian age.

Equivalent Fossil Calibrations: None.

Fossil tip age: 47.8 Ma. The node calibration was created such that 97.5% of the probability distribution fell before 47.8 Ma.

†*Eritherium azzouzorum*

Justification of Placement: †*Eritherium azzouzorum* calibrates the MRCA of crown *Afrotheria* (specifiers: *Loxodonta africana*, *Orycteropus afer*) in our node-dating analyses.This is a somewhat conservative placement since multiple phylogenetic analyses of morphological characters resolve †*Eritherium azzouzorum* as a stem-probiscidean, including the 143 character, 20 taxon matrix of Gheerbrant (Gheerbrant 2009) and the 109 character, 44 taxon matrix of Avilla and Mothé (Avilla and Mothé 2021). Other analyses of the latter matrix resolve this taxon on the stem of *Sirenia* (Kramarz and MacPhee 2022). Finally, the 209 character, 33 taxon matrix of Gheerbrant et al. (Gheerbrant et al. 2018) resolves †*E. azzouzorum* on the stem of *Sirenia*+*Probiscidea*. Because of this uncertainty in the placement of †*E. azzouzorum* among

bioRxiv preprint doi: https://doi.org/10.64898/2026.06.28.735080; this version posted June 29, 2026. The copyright holder for this preprint (which was not certified by peer review) is the author/funder, who has granted bioRxiv a license to display the preprint in perpetuity. It is made available under a CC-BY 4.0 International license.

Paenungulata (*Probiscidea*, *Hyracoidea,* and *Sirenia*), which itself evades resolution using genomic data (Seiffert 2007; Bowman et al. 2023; Foley et al. 2023; Liu et al. 2024), we used this taxon to calibrate the MRCA at the base of *Afrotheria*. For the purposes of this justification, we rely on the phylogeny presented in figure 4 of Gheerbrant et al. (Gheerbrant et al. 2018). Because no character state optimizations were provided by Gheerbrant et al. (Gheerbrant et al. 2018) for the placement of †*E. azzouzorum* in crown *Afrotheria* on the stem of *Probiscidea*+*Sirenia* (=*Tethytheria*), we reran the matrix presented in the paper using TNT v 1.5 (Goloboff and Catalano 2016) to optimize apomorphies; we ran an initial Wagner search over 1000 replicates and default parameters for ratchet, tfuse, drift, and sectorial search followed by traditional bisection reconnection branch swapping over 100,000 trees. We summarized the topologies in a strict consensus and then recorded apomorphies of two nodes: crown *Afrotheria* and Pan-*Tethytheria.* Phylogenetically optimized apomorphies uniting †*E. azzouzorum* and crown *Afrotheria* are: postmetacrista predominantly longitudinal (110:1 in Gheerbrant et al. (Gheerbrant et al. 2018)) and preparacrista oriented longitudinally (111:1 in Gheerbrant et al. (Gheerbrant et al. 2018)). Phylogenetically optimized apomorphies uniting †*E. azzouzorum* and *Tethytheria* are: preparacrista absent or weak (112:1 in Gheerbrant et al. (Gheerbrant et al. 2018)),and external auditory meatus elevated high above tooth row (181:1 in Gheerbrant et al. (Gheerbrant et al. 2018)).

Stratigraphic Horizon: Sidi Chennane quarries, Ouled Abdoun phosphate basin, Morocco; early Thanetian (Gheerbrant 2009). we follow Foley et al. (Foley et al. 2023) in using an age of 59.2 Ma.

Equivalent Fossil Calibrations: Highly dependent on phylogeny. Equivalent-age or older fossils potentially assignable to crown *Afrotheria* are known, but their placement is unclear. See cited phylogenetic studies in “Justification of Placement” section above.

Fossil tip age: 59.2 Ma. The node calibration was created such that 97.5% of the probability distribution fell before 59.2 Ma.

†*Acritoparamys atavus*

Justification of Placement: †*Acritoparamys atavus* calibrates the MRCA of crown *Rodentia* (specifiers: *Mus musculus*, *Hydrochoerus hydrochaeris*) in our node-dating analyses. The placement of †*A. atavus* as a member of crown *Rodentia* in the stem-sciuromorph clade

bioRxiv preprint doi: https://doi.org/10.64898/2026.06.28.735080; this version posted June 29, 2026. The copyright holder for this preprint (which was not certified by peer review) is the author/funder, who has granted bioRxiv a license to display the preprint in perpetuity. It is made available under a CC-BY 4.0 International license.

†*Ischyromyidae* is supported by parsimony phylogenetic analyses of morphological characters, including the 106 character, 91 taxon matrix of Marivaux et al (Marivaux et al. 2004) and the 343 character, 45 taxon matrix of Vianey-Laud and Marivaux (Vianey-Liaud and Marivaux 2021). For this purposes of this justification, we rely on the phylogeny presented in figure 1 of Marivaux et al. (Marivaux et al. 2004), which unites this taxon with other early-diverging 'ischyromiform' rodents primarily on the basis of protrogomorphy and the presence of pauciserial enamel.

Stratigraphic Horizon: Bear Creek, Montana, USA; Fort Union Formation, Clarkfordian, 56.0 Ma (Korth 1994; Dawson and Beard 1996; Dawson et al. 2008; Casanovas-Vilar et al.).

Equivalent Fossil Calibrations: †*Paramys adamus* is an equivalent fossil calibration of similar placement in rodent phylogeny (within "†*Ischyromyidae*") (Marivaux et al. 2004). †*Eliwourus topernawiensis* is a crown rodent from 29.5 Ma in Kenya, and is used as the calibration for crown *Rodentia* in the sensitivity analysis (Seiffert et al. 2025).

Fossil tip age: 56.0 Ma. The node calibration was created such that 97.5% of the probability distribution fell before 56.0 Ma.

†*Purgatorius janisae*

Justification of Placement: †*Purgatorius janisae* calibrates the MRCA of *Primates* and *Dermoptera* (specifiers: *Galeopterus variegatus*, *Homo sapiens*) in our node-dating analyses. The placement of †*Purgatorius* as a member of Pan-*Primates* is supported by parsimony phylogenetic analyses of morphological characters, including the 415 character, 85 taxon matrix of Chester et al. (Chester et al. 2015) and the 240 character, 30 taxon matrix of Chester et al. (Chester et al. 2017) For the purposes of this justification, we rely on the phylogeny presented in figure S2 of Chester et al. (Chester et al. 2017) Phylogenetically optimized apomorphies uniting †*Purgatorius* spp. and *Primatomorpha* (*Dermoptera* + *Primates*) are: plantar pit on cuboid facet of calcaneum present (51:1 in Chester et al. (Chester et al. 2017)), m1 metastyle absent (163:0 in Chester et al. (Chester et al. 2017)), and m1, both conules present (165:0 in Chester et al. (Chester et al. 2017)). Phylogenetically optimized apomorphies uniting †*Purgatorius* spp. and Pan-*Primates* are: m1, precingulum present and not connected to postcingulum (156:0 in Chester et al. (Chester et al. 2017)), m1, entoconid notch absent (219:0 in Chester et al. (Chester et al. 2017)), m3 greater in length than m2 (226:1 in Chester et al. (Chester et al. 2017)), and lower

bioRxiv preprint doi: https://doi.org/10.64898/2026.06.28.735080; this version posted June 29, 2026. The copyright holder for this preprint (which was not certified by peer review) is the author/funder, who has granted bioRxiv a license to display the preprint in perpetuity. It is made available under a CC-BY 4.0 International license.

molars that get progressively larger from m1 to m3 (229:1 in Chester et al. (Chester et al. 2017)). See Mantilla et al. (Wilson Mantilla et al. 2021) for comparisons of dental morphology across the earliest known †*Purgatorius* species.

Stratigraphic Horizon: 'Harley's Point' UCMP locality V77087, Montana, USA; Tullock Member of the Fort Union Formation, early Puercan, lowermost Paleocene (Wilson Mantilla et al. 2021). We follow Foley et al. in using an age of 65.5 Ma (Foley et al. 2023).

Equivalent Fossil Calibrations: Several other species of †*Purgatorius.* See Mantilla et al. (Wilson Mantilla et al. 2021). †*Archicebus achilles* from the lower Eocene of China (54.8 Ma), is a probable crown primate that was used to calibrate the node of crown primates (this same node excluding *Galeopterus*) in the sensitivity analysis (Ni et al. 2013).

Fossil tip age: 65.5 Ma. The node calibration was created such that 97.5% of the probability distribution fell before 65.5 Ma.

†*Hesperocyon gregarious*

Justification of Placement: †*Hesperocyon gregarious* calibrates the MRCA of *Carnivora* (specifiers: *Panthera onca*, *Canis lupus*) in our node-dating analyses. The placement of †*Hesperocyon gregarious* as a member of *Carnivora* on the stem of *Canidae* is supported by parsimony phylogenetic analyses of morphological characters, including the 99 character, 48 taxon matrix of Wesley-Hunt and Flynn (Wesley-Hunt and Flynn 2005), the 98 character, 36 taxon matrix of Wesley-Hunt and Werdelin (Wesley-Hunt and Werdelin 2005), the 98 character, 50 taxon matrix of Tomiya (Tomiya 2011), the 123 character, 121 taxon matrix of Slater (Slater 2015), and the 108 character, 28 taxon matrix of Tomiya and Tseng (Tomiya and Tseng 2016). Because of instability in the placement of different lineages in *Carnivora* in these analyses, we chose to conservatively use †*H. gregarious* to calibrate the crown of *Carnivora* in our analyses. For the purposes of this justification, we rely on the phylogeny presented in figure 1 in Tomiya and Tseng (Tomiya and Tseng 2016). Phylogenetically optimized apomorphies uniting †*H. gregarious* to *Canoidea* in crown *Carnivora* are: infraorbital foramen above P4 anterior edge (4:1 in Tomiya and Tseng (Tomiya and Tseng 2016)), basioccipital lateral flange large (34:2 in Tomiya and Tseng (Tomiya and Tseng 2016)), M3 absent (53:1 in Tomiya and Tseng (Tomiya and Tseng 2016)), and M1 postprotocrista posteriorly directed (102:1 in Tomiya and Tseng (Tomiya and Tseng 2016)).

bioRxiv preprint doi: https://doi.org/10.64898/2026.06.28.735080; this version posted June 29, 2026. The copyright holder for this preprint (which was not certified by peer review) is the author/funder, who has granted bioRxiv a license to display the preprint in perpetuity. It is made available under a CC-BY 4.0 International license.

Stratigraphic Horizon: northeastern Colorado, USA; White River Formation. Also known from the Cypress Hills Formation, Saskatchewan, Canada, the Renova Formation, western Montana, USA ; Chadron and Brule Formations, Colorado, Nebraska, North Dakota, South Dakota, and Wyoming, USA (Wang and Tedford 1996). Duchesnian to Whitneyan Land Mammal Stages (Wang and Tedford 1996); following Foley et al. (Foley et al. 2023) we use the midpoint of 37.71 Ma.

Equivalent Fossil Calibrations: Several in the genera †*Daphoenus* and †*Hesperocyon*. See Wesley-Hunt and Flynn (Wesley-Hunt and Flynn 2005), Benton and Donoghue (Benton and Donoghue 2007), and Slater (Slater 2015).

Fossil tip age: 37.71 Ma. The node calibration was created such that 97.5% of the probability distribution fell before 37.71 Ma.

†*Icaronycteris spp.*

Justification of Placement: †*Icaronycteris* spp. calibrates the MRCA of *Chiroptera* (specifiers: *Pteronotus parnellii*, *Megaderma lyra*) in our node-dating analyses. The placement of †*Icaronycteris spp.* as a member of *Chiroptera* in a polytomy with crown clades is supported by parsimony phylogenetic analyses of morphological characters, including the 568 character, 14 taxon matrix of Reitbergen et al. (Rietbergen et al. 2023), and the 699 character, 82 taxon matrix of Jones et al. (Jones et al. 2024). Several analyses have also recovered †*Icaronycteris* spp. on the stem of *Chiroptera*, but close to the crown clade compared to other Eocene bats. These phylogenies include the 50% majority rule tree in Jones et al. (Jones et al. 2024), phylogenies generated from the 172 character, 30 taxon matrix of Simmons and Geisler (Simmons and Geisler 1998), and the 2665 character, 30 taxon matrix of Hand et al. (Hand et al. 2023). However, because several similarly aged Eocene bats of potential crown group affinity are known, and given the unstable relationships of extinct Eocene bats to the crown (Simmons and Geisler 1998; Hand et al. 2023; Rietbergen et al. 2023; Jones et al. 2024), we use †*Icaronycteris* spp. to calibrate the crown. For the purposes of this justification, we rely on the phylogeny presented in figure 2 in Jones et al. (Jones et al. 2024). Phylogenetically optimized apomorphies uniting †*Icaronycteris spp.* to the most inclusive clade containing these Green River bats and crown bat clades (node 19 in Jones et al. (Jones et al. 2024)) are (NB: some of these are variable in different species of †*Icaronycteris*): upper canine with flattened posterolabial surface (38:1 in

bioRxiv preprint doi: https://doi.org/10.64898/2026.06.28.735080; this version posted June 29, 2026. The copyright holder for this preprint (which was not certified by peer review) is the author/funder, who has granted bioRxiv a license to display the preprint in perpetuity. It is made available under a CC-BY 4.0 International license.

Jones et al. (Jones et al. 2024)), m1-2 with lingual cingulum (75:1 in Jones et al. (Jones et al. 2024)), longitudinally oriented roots of p3 (177:0 in Jones et al. [180,181](Jones et al. 2024)), p3 slightly shorter than p4 (187:3 in Jones et al. (Jones et al. 2024)), p3 between 50% to 100% the size of p4 (188:2 in in Jones et al. (Jones et al. 2024)), p4 with one metaconid (203:1 in in Jones et al. (Jones et al. 2024)), and m3 hypoconulid small and low (280:1 in in Jones et al. (Jones et al. 2024)).

Stratigraphic Horizon: The oldest species of †*Icaronycteris* is from the American Fossil Quarry Wyoming, USA; Fossil Butte Member of the Green River Formation. This locality matches the age of Fossil Lake, which is minimally dated to 51.98 Ma (Smith et al. 2008; Rietbergen et al. 2023).

Equivalent Fossil Calibrations: Various, including the perhaps slightly (47.8 Ma) younger (though still Eocene) †*Tachypteron franzeni*, †*Witwatia sigei*, and †*Dizzya exsultans*, which have previously been used to constrain the age of *Chiroptera* (Foley et al. 2023) but are also unstable in phylogenetic position and are represented by fossils that are less character-rich than those of many species of †*Icaronycteris* (Jones et al. 2024). These other fossils were used in place of †*Icaronycteris* in the sensitivity analysis.

Fossil tip age: 51.98 Ma. The node calibration was created such that 97.5% of the probability distribution fell before 51.98 Ma.

†*Enaliarctos tedfordi*

Justification of Placement: †*Enaliarctos tedfordi* calibrates the MRCA of *Arctoidea* (specifiers: *Ailurus stylani*, *Ailuropoda melanoleuca*) in our node-dating analyses. The placement of †*E. tedfordi* in crown *Arctoidea* on the stem of *Pinnipedia* is supported by parsimony and Bayesian phylogenetic analyses of morphological characters, including the 52 character, 9 taxon matrix of Berta (Berta 1991) and the 204 character, 65 taxon matrix of Paterson et al. (Paterson et al. 2020). For the purposes of this justification, we rely on the phylogeny presented in figure 3 in Paterson et al. (Paterson et al. 2020). Phylogenetically optimized apomorphies uniting †*E. tedfordi* with Pan-*Pinnipedia* (=*Pinnipedomorpha* + *Puijila* clade in Paterson et al. (Paterson et al. 2020)) are: postorbital constriction present, long (3:2 in Paterson et al. (Paterson et al. 2020)), prominent pseudosylvian sulcus on braincase (36:1 in 1 in Paterson et al. (Paterson et al. 2020)),

bioRxiv preprint doi: https://doi.org/10.64898/2026.06.28.735080; this version posted June 29, 2026. The copyright holder for this preprint (which was not certified by peer review) is the author/funder, who has granted bioRxiv a license to display the preprint in perpetuity. It is made available under a CC-BY 4.0 International license.

fenestra cochleae larger than oval window (70:1 in Paterson et al. (Paterson et al. 2020)), and m1 reduced relative to p4 (95:1 in Paterson et al. (Paterson et al. 2020)).

Stratigraphic Horizon: Beaver Creek, Lincoln County, Oregon; Yaquina Formation, Oligocene, 28.1 Ma (Prothero et al. 2001; Poust and Boessenecker 2018; Paterson et al. 2020; Foley et al. 2023).

Equivalent Fossil Calibrations: Potentially the pan-mustelids †*Mustelavus priscus*, †*Pseudobassaris riggsi*, the arctoid †*Amphicticeps shackelfordi*, and the pan-arctids †*Allocyon loganensis* and †*Phoberogale* sp. (Paterson et al. 2020).

Fossil tip age: 28.1 Ma. The node calibration was created such that 97.5% of the probability distribution fell before 28.1 Ma.

†*Ravenictis krausei*

Justification of Placement: †*Ravenictis krausei* calibrates the MRCA of *Ferae* (specifiers: *Ailurus stylani*, *Manis pentadactyla*) in our node-dating analyses. The identification of †*Ravenictis krausei* as a viverrid pan-carnivoran is based on the following morphological features: crown asymmetry, and the development of the protocone cingula (Fox and Youzwyshyn 1994). Wear patterns and the relative development of the cusps were also cited as support for a pan-carnivoran identity (Fox and Youzwyshyn 1994). We follow previous analyses (Foley et al. 2023) in using †*Ravenictis krausei* as a calibration for the split between *Carnivora* and its sister lineage *Pholidota.*

Stratigraphic Horizon: Saskatchewan, Canada; Rav W-1 Horizon, Ravenscrag Formation, Paleocene (Clarkfordian), 64.0 Ma (Fox and Youzwyshyn 1994; Foley et al. 2023).

Equivalent Fossil Calibrations: Various, including several slightly younger (62.0-56.0 Ma) stem-carnivorans in †*Viverridae* and †*Miacidae* (Fox and Youzwyshyn 1994; Wesley-Hunt and Flynn 2005; Tomiya 2011; Tomiya and Tseng 2016). This slightly younger age (56.0 Ma) was used for the sensitivity analysis.

Fossil tip age: 64.0 Ma. The node calibration was created such that 97.5% of the probability distribution fell before 64.0 Ma.

†*Mystacodon selensis*

bioRxiv preprint doi: https://doi.org/10.64898/2026.06.28.735080; this version posted June 29, 2026. The copyright holder for this preprint (which was not certified by peer review) is the author/funder, who has granted bioRxiv a license to display the preprint in perpetuity. It is made available under a CC-BY 4.0 International license.

Justification of Placement: †*Mystacodon selensis* calibrates the MRCA of *Cetacea* (specifiers: *Orcinus orca*, *Balaenoptera acutorostrata*) in our node-dating analyses. The placement of †*M. selensis* in crown *Cetacea* on the stem of *Mysticeti* is supported by parsimony and Bayesian phylogenetic analyses of morphological characters, including the 272 character, 38 taxon matrix of Lambert et al. (Lambert et al. 2017), the 391 character, 138 taxon dataset of Boessenecker et al. (Boessenecker et al. 2023), and the 282 character, 35 taxon matrix of Muizon et al. (Anon 2019). For the purposes of this justification, we rely on the phylogeny presented in figure 52 of Muizon et al.(Anon 2019). Phylogenetically optimized apomorphies uniting †*M. selensis* with Pan-*Mysticeti* in crown *Cetacea* are: maxilla roughly as high as wide or lower relative to width at transverse midpoint of section anteroposteriorly located at the level of posterior third of the bone length but anterior to antorbital process (5:1 in Muizon et al. (Anon 2019)), anteriormost portion of jugal broadly underlapped by maxilla (54:1 in Muizon et al. (Anon 2019)), presence of the maxillary infraorbital plate (57:1 in Muizon et al. (Anon 2019)), zygomatic process apex of squamosal closely apposed or situated ventral to postorbital process (102:1 in Muizon et al. (Anon 2019)), supraoccipital triangular in dorsal view (115:1 in Muizon et al. (Anon 2019)), external occipital crest restricted to anterior half of supraoccipital shield (117:1 in Muizon et al. (Anon 2019)), height of dentary does not taper ventrally at anterior extremity (226:1 in Muizon et al. (Anon 2019)), and humeral head oriented posteriorly to posteroproximally (265:1 in Muizon et al. (Anon 2019)).

Stratigraphic Horizon: Playa Media Luna locality, Pisco Basin, Peru; Yumaque Member of the Paracas Formation, Eocene (Priabonian), 36.4 Ma (Lambert et al. 2017; Anon 2019).

Equivalent Fossil Calibrations: None. The closest fossil calibration is the slightly younger †*Llanocetus denticrenatus*, which is another pan-mysticete that is closer to the crown clade than †*Mystacodon selensis* (Lambert et al. 2017; Fordyce and Marx 2018; Fordyce and Marx 2018; Anon 2019; Boessenecker et al. 2023).

Fossil tip age: 36.4 Ma. The node calibration was created such that 97.5% of the probability distribution fell before 36.4 Ma.

†*Megachirella watchleri*

Justification of Placement: †*Megachirella watchleri* calibrates the MRCA of crown *Lepidosauria* (specifiers: *Sphenodon punctatus*, *Python molurus*) in our node-dating analyses.

bioRxiv preprint doi: https://doi.org/10.64898/2026.06.28.735080; this version posted June 29, 2026. The copyright holder for this preprint (which was not certified by peer review) is the author/funder, who has granted bioRxiv a license to display the preprint in perpetuity. It is made available under a CC-BY 4.0 International license.

The placement of †*M. watchleri* within crown *Lepidosauria* is supported by parsimony and Bayesian phylogenetic analyses of morphological characters, including the 347 character, 129 taxon dataset of Simões et al. (Simões et al. 2018) (including its modifications in Bittencourt et al. (Bittencourt et al. 2020), Brownstein et al. (Chase D. Brownstein et al. 2023), Martinez et al. (Martínez et al. 2021), and Sobral et al. (Sobral et al. 2020)), the 348 character, 125 taxon matrix of Simões et al. (Simões et al. 2022), the 381 character, 116 taxon matrix of Ford et al. (Ford et al. 2021), the 377 character, 115 taxon matrix of Griffiths et al. (Griffiths et al. 2021), and the 131 character, 29 taxon matrix of Simões et al. (Simões et al. 2020) (including as modified by Freisem et al. (Freisem et al. 2024) The modified version of the matrix in Griffiths et al. (Griffiths et al. 2021)/ Ford et al. (Ford et al. 2021) presented in Tałanda et al. (Tałanda et al. 2022) recovers †*M. watchleri* within a clade of Early Triassic to Middle Jurassic pan-lepidosaurs that forms the sister to the crown group. However, the varied morphology of members of this clade and the incomplete nature of some of these specimens (see Griffiths et al. (Griffiths et al. 2021) and Ford et al. (Ford et al. 2021)) means that its monophyly should be viewed with caution, especially since different methodologies applying molecular constraints induce the collapse of this clade into a polytomy of lepidosaur total group lineages (see extended data figure 8b in Tałanda et al. (Tałanda et al. 2022)). In any case, equivalent calibrations exist (see below). For the purposes of this justification, we rely on the phylogeny presented in figure 2 of Simões et al. (Simões et al. 2018). Phylogenetically optimized apomorphies uniting †*M. wetmorei* with Pan-*Lepidosauria* are: posterior dentary teeth

delimited by a labial wall only (212:0 in Simões et al. (Simões et al. 2018)), posterior maxillary teeth delimited by a labial wall only (213:0 in Simões et al. (Simões et al. 2018)), and presacral pleurocentra, midventral crest present (232:1 in Simões et al. (Simões et al. 2018)). †*M. wetmorei* is united with *Lepidosauria* based on a single phylogenetically optimized apomorphy: frontals fused (67:1 in Simões et al. (Simões et al. 2018)). Phylogenetically optimized apomorphies uniting †*M. wetmorei* with Pan-*Squamata* are: absence of squamosal anteroventral process (50:0 in Simões et al. (Simões et al. 2018)), lateral process of ectopterygoid absent (114:0 in Simões et al. (Simões et al. 2018)), alar crest of prootic present (142:1 in Simões et al.

1. (Simões et al. 2018)), clavicles secondarily curved anteroposteriorly (287:1 in Simões et al. (Simões et al. 2018)), developed radial condyle on humerus present (310:0 in Simões et al. (Simões et al. 2018)), distal carpal 1 fused to metacarpal (324:1 in Simões et al. (Simões et al.

bioRxiv preprint doi: https://doi.org/10.64898/2026.06.28.735080; this version posted June 29, 2026. The copyright holder for this preprint (which was not certified by peer review) is the author/funder, who has granted bioRxiv a license to display the preprint in perpetuity. It is made available under a CC-BY 4.0 International license.

2018)).

Stratigraphic Horizon: Monte Prà della Vacca, Braies Dolomites, Bolzano, Italy (Renesto and Bernardi 2014); Dont Formation, Braies Group, Anisian Stage of the Early Triassic, approximately 242.0 Ma (Renesto and Bernardi 2014; Simões et al. 2018).

Equivalent Fossil Calibrations: †*Wirtembergia hauboldae* from the Ladinian (242.0 to 237.0 Ma)(Gradstein et al. 2021) Erfut Formation of Germany is the oldest known rhychocephalian and is only slightly younger than †*Megachirella watchleri* from the Anisian of Italy. †*Fraxinisaura rozynekae* is a lepidosaur that is also known from the Erfut Formation of Germany, but whose affinities to the lepidosaur crown clade remain unresolved (Schoch and Sues 2018; Ford et al. 2021; Griffiths et al. 2021; Chase D. Brownstein et al. 2023).

Fossil tip age: 242.0 Ma. The node calibration was created such that 97.5% of the probability distribution fell before 242.0 Ma.

†*Eoscincus ornatus*

Justification of Placement: †*Eoscincus ornatus* calibrates the MRCA of crown *Unidentata* (specifiers: *Hemicordylus capensis*, *Python molurus*) in our node-dating analyses. The placement of †*E. ornatus* within crown *Unidentata* is supported by parsimony and Bayesian phylogenetic analyses of morphological characters, including the 622 character, 119 taxon matrix of Brownstein et al. (Brownstein et al. 2022) and the 637 character, 154 taxon matrix of Meyer et al. (Meyer et al. 2023). For the purposes of this justification, we use the phylogeny presented in figure s10 of Brownstein et al. (Brownstein et al. 2022). Phylogenetically optimized apomorphies uniting †*E. ornatus* with Pan-*Scincoidea* in crown *Squamata* are: surangular adductor fossa of the external face of mandible is deep and extends ventrally more than halfway down (399:1 in Brownstein et al. (Brownstein et al. 2022)). Absence of the lateral exposure of the jugal below the orbit due to obscuration by the maxilla jugal process (149:0 in Brownstein et al. (Brownstein et al. 2022)) is also found to be a character uniting †*E. ornatus* with Pan-*Scincoidea* in the analysis constrained based on molecular phylogenies. Phylogenetically optimized apomorphies uniting †*E. ornatus* with †*Paramacellodidae* in Pan-*Scincoidea* are: jugal broadly separated from prefrontal (144:0 in Brownstein et al. (Brownstein et al. 2022)), jugal medial ridge weakly developed such that the jugal is positioned lateral to the ectopterygoid at base in dorsal view (157:0 in Brownstein et al. (Brownstein et al. 2022)), 10 to 20 dentary

bioRxiv preprint doi: https://doi.org/10.64898/2026.06.28.735080; this version posted June 29, 2026. The copyright holder for this preprint (which was not certified by peer review) is the author/funder, who has granted bioRxiv a license to display the preprint in perpetuity. It is made available under a CC-BY 4.0 International license.

teeth (421:2 in Brownstein et al. (Brownstein et al. 2022)), posterior teeth unicuspid (434:0 in in Brownstein et al. (Brownstein et al. 2022)), and dermal skull roof bones ornamented with slight rugosities about the frontoparietal suture (572:1 in Brownstein et al. (Brownstein et al. 2022)).

Stratigraphic Horizon: DINO 317 Quarry, Dinosaur National Monument, Utah, USA; Brushy Basin Member of the Morrison Formation, Tithonian Stage of the Late Jurassic, approximately 145.0 Ma.(Brownstein et al. 2022)

Equivalent Fossil Calibrations: †*Microteras borealis*, known from the Tithonian Morrison Formation of Wyoming, is an equivalent fossil calibration (Brownstein et al. 2022).

Fossil tip age: 145.0 Ma. The node calibration was created such that 97.5% of the probability distribution fell before 145.0 Ma.

†*Chthonophis subterraneus*

Justification of Placement: †*Chthonophis subterraneus* calibrates the MRCA of *Lacertoidea* (specifiers: *Lacerta agilis*; *Salvator merianae*) in our node-dating analyses. The placement of †*C. subterraneus* within *Lacertoidea* as a member of Pan-*Amphisbaenia* is supported by parsimony phylogenetic analyses of morphological characters, including the 308 character, 51 taxon matrix of Longrich et al. (Longrich et al. 2015). For the purposes of this justification, we use the phylogeny presented in figure S1 of Longrich et al. (Longrich et al. 2015). Because no character state optimizations were provided by Longrich et al. (Longrich et al. 2015) for the placement of †*C. subterraneus* within *Lacertoidea* as a member of Pan-*Amphisbaenia*, we reran the matrix presented in the paper using TNT v 1.5(Goloboff and Catalano 2016) to optimize apomorphies; we ran an initial Wagner search over 1000 replicates and default parameters for ratchet, tfuse, drift, and sectorial search followed by traditional bisection reconnection branch swapping over 100,000 trees. We summarized the topologies in a strict consensus and then recorded apomorphies of Pan-*Amphisbaenia*. Phylogenetically optimized apomorphies uniting †*C. subterraneus* with Pan-*Amphisbaenia* are: dentary symphysis with extensive development of a symphyseal facet extending caudally below the Meckelian groove (165:1 in Longrich et al. (Longrich et al. 2015)), Fusion of marginal teeth: unfused to each other (0); fused to each other (1). Marginal tooth crowns separated by large gaps (220: 1 in Longrich et al. (Longrich et al. 2015)), and eight dentary teeth present (231:2 in Longrich et al. (Longrich et al. 2015)).

bioRxiv preprint doi: https://doi.org/10.64898/2026.06.28.735080; this version posted June 29, 2026. The copyright holder for this preprint (which was not certified by peer review) is the author/funder, who has granted bioRxiv a license to display the preprint in perpetuity. It is made available under a CC-BY 4.0 International license.

Stratigraphic Horizon: Bug Creek Anthills, Fort Union Formation, Montana, USA; Maastrichtian Stage of Late Cretaceous to earliest Danian Stage of Paleocene (Longrich et al. 2015), 66.02 Ma (Gradstein et al. 2021).

Equivalent Fossil Calibrations: None.

Fossil tip age: 66.02 Ma. The node calibration was created such that 97.5% of the probability distribution fell before 66.02 Ma.

†*Priscagama gobiensis*

Justification of Placement: †*Priscagama gobiensis* calibrates the MRCA of *Iguania* (specifiers: *Pogona vitticeps*, *Anolis carolinensis*) in our node-dating analyses. The placement of †*P. gobiensis* within *Iguania* as a member of Pan-*Acrodonta* is supported by parsimony and Bayesian phylogenetic analyses of morphological characters, including the 610 character, 192 taxon matrix of Gauthier et al. (Gauthier et al. 2012), the 347 character, 129 taxon dataset of Simões et al.,(Simões et al. 2018) the 622 character, 119 taxon matrix of Brownstein et al. (Brownstein et al. 2022), and the 155 character, 133 taxon matrix of Scarpetta (Scarpetta 2024). For the purposes of this justification, we use the phylogeny presented in figure S10 of Brownstein et al. (Brownstein et al. 2022). Because no character state optimizations were provided by Brownstein et al. (Brownstein et al. 2022) for the placement of †*P. gobiensis* within *Iguania* as a member of Pan-*Acrodonta*, we reran the matrix presented in the paper using TNT v 1.5 (Goloboff and Catalano 2016) to optimize apomorphies; we ran an initial Wagner search over 1000 replicates and default parameters for ratchet, tfuse, drift, and sectorial search followed by traditional bisection reconnection branch swapping over 100,000 trees. We summarized the topologies in a strict consensus and then recorded apomorphies of *Iguania* and Pan-*Acrodonta*. Phylogenetically optimized apomorphies uniting †*P. gobiensis* with *Iguania* are: nasals in contact dorsal to premaxillary nasal process (25:1 in Brownstein et al. (Brownstein et al. 2022)), postorbital contacts parietal ventrolaterally at frontoparietal suture (71:2 in Brownstein et al. (Brownstein et al. 2022)), and dentary coronoid process posteriorly terminates just behind level of coronoid apex (364:1 in Brownstein et al. (Brownstein et al. 2022)). Phylogenetically optimized apomorphies uniting †*P. gobiensis* with Pan-*Acrodonta* are: frontal interorbital width 28-34% of frontoparietal suture width (48:3 in Brownstein et al. (Brownstein et al. 2022)) and

bioRxiv preprint doi: https://doi.org/10.64898/2026.06.28.735080; this version posted June 29, 2026. The copyright holder for this preprint (which was not certified by peer review) is the author/funder, who has granted bioRxiv a license to display the preprint in perpetuity. It is made available under a CC-BY 4.0 International license.

maxillary tooth row extends to posterior third of orbit (415:1 in Brownstein et al. (Brownstein et al. 2022)).

Stratigraphic Horizon: Campanian-Maastrichtian of the Gobi Desert of Mongolia and China;(Borsuk-Bialynicka and Moody 1984; Keqin and Lianhai 1995; Gao and Norell 2000) following Brownstein et al. (Brownstein et al. 2022) we use an age of 71.0 Ma.

Equivalent Fossil Calibrations: At least 10 other species of iguanian have been reported from the same Campanian units of the Gobi Desert. Among these, †*Ctenomastax parva*, †*Mimeosaurus crassus*, †*Phrynosomimus asper*, †*Isodontosaurus gracilis*, †*Zapsosaurus sceliphros*, and †*Polrussia mongoliensis* have been placed within the crown group on the stems of *Pleurodonta* and *Acrodonta* in multiple phylogenetic analyses (Gauthier et al. 2012; Longrich et al. 2012; Simões et al. 2018; Brownstein et al. 2022; Scarpetta 2024).

Fossil tip age: 71.0 Ma. The node calibration was created such that 97.5% of the probability distribution fell before 71.0 Ma.

†*Arcanosaurus ibericus*

Justification of Placement: †*Arcanosaurus ibericus* calibrates the MRCA of crown *Anguimorpha* (specifiers: *Heloderma charlesbogerti*, *Varanus komodoensis*) in our node-dating analyses. The identification of †*A. ibericus* as a member of Pan-*Varanidae* in *Anguimorpha* is based on the following morphological features: cervical vertebrae with a hypapophysis originating from the centrum with individualized intercentral elements, obliquity of the axis of the cotyle–condyle system, articulated caudal chevron intercentra present (Houssaye et al. 2013).

Stratigraphic Horizon: Viajete site, Salas de los Infantes, Cameros Basin, Iberian Peninsula, Burgos, Spain; Castrillo de la Reina Formation, Barremian to Aptian Stages of the Early Cretaceous.(Houssaye et al. 2013) We use an age of 121.4 Ma, which represents the boundary for these two stages.(Gradstein et al. 2021)

Equivalent Fossil Calibrations: †*Dorsetisaurus* spp. is a crown squamate known from fossils as old as the Tithonian Stage of the Late Jurassic (~145 Ma) that has variously been placed in Pan-*Anguimorpha* or within the crown group (Evans et al. 2006; Conrad 2008).

Fossil tip age: 121.4 Ma. The node calibration was created such that 97.5% of the probability distribution fell before 121.4 Ma.

bioRxiv preprint doi: https://doi.org/10.64898/2026.06.28.735080; this version posted June 29, 2026. The copyright holder for this preprint (which was not certified by peer review) is the author/funder, who has granted bioRxiv a license to display the preprint in perpetuity. It is made available under a CC-BY 4.0 International license.

†*Messelopython freyi*

Justification of Placement: †*Messelopython freyi* calibrates the MRCA of crown *Pythonomorpha* (specifiers: *Python molurus*, *Naja naja*) in our node-dating analyses. The placement of †*M. freyi* within *Pythonomorpha* as a member of Pan-*Pythonidae* (and indeed, the pythonid crown group) is supported by parsimony and Bayesian phylogenetic analyses of morphological characters, including the 785 character, 90 taxon matrix of Zaher and Smith,(Zaher and Smith 2020) the 264 character, 52 taxon matrix of Onary et al. (Onary et al. 2021), the 240 character, 50 taxon matrix of Palci et al. (Palci et al. 2024), and the 240 character, 51 taxon matrix of Croghan et al. (Croghan et al. 2024). For the purposes of this justification, we use the phylogeny presented in figure 2a of Zaher and Smith (Zaher and Smith 2020). Because no character state optimizations were provided by Zaher and Smith (Zaher and Smith 2020) for the placement of †*M. freyi* within *Pythonomorpha* as a member of Pan-*Pythonidae*, we reran the matrix presented in the paper using TNT v 1.5 (Goloboff and Catalano 2016) to optimize apomorphies; we ran an initial Wagner search over 1000 replicates and default parameters for ratchet, tfuse, drift, and sectorial search followed by traditional bisection reconnection branch swapping over 100,000 trees. We summarized the topologies in a strict consensus and then recorded apomorphies of Pan-*Pythonidae*. Phylogenetically optimized apomorphies uniting †*Messelopython freyi* with Pan-*Pythonidae* include: quadrate lateral head off crest borders ventral fossa (231:1 in Zaher and Smith (Zaher and Smith 2020)), palatine ventromedial extension from maxillary process present but does not extend ventromedially (296:0 in Zaher and Smith (Zaher and Smith 2020)), palatine choanal process forms a short vertical or horizontal lamina that does not reach the vomer (312:2 in Zaher and Smith (Zaher and Smith 2020)), and posterior border of the palatine process of the maxilla inconspicuous or rounded, not projecting posteromedially (771:0 in Zaher and Smith (Zaher and Smith 2020)).

Stratigraphic Horizon: Messel Pit, Germany; Middle Messel Formation, which is at minimum the upper bound of the Geiseltalian Mammal Stage at 46.5 Ma (Mertz and Renne 2005).

Equivalent Fossil Calibrations: Several, including the booids †*Messelophis variatus*, †*Rieppelophis ermannorum,* †*Eoconstrictor fischeri*, †*E. spinifer*, †*E. barnesi*, and †*Rageryx schmidi* (Zaher and Smith 2020; Croghan et al. 2024; Palci et al. 2024).

Fossil tip age: 46.5 Ma. The node calibration was created such that 97.5% of the probability distribution fell before 46.5 Ma.

bioRxiv preprint doi: https://doi.org/10.64898/2026.06.28.735080; this version posted June 29, 2026. The copyright holder for this preprint (which was not certified by peer review) is the author/funder, who has granted bioRxiv a license to display the preprint in perpetuity. It is made available under a CC-BY 4.0 International license.

†*Notoemys oxfordiensis*

Justification of Placement: †*Notoemys oxfordiensis* calibrates the MRCA of crown *Testudines* (specifiers: *Podocnemis expansa*, *Chelydra serpentina*) in our node-dating analyses. The placement of †*N. oxfordiensis* within *Testudines* in Pan-*Pleurodira* is supported by parsimony phylogenetic analyses of morphological characters, including the 245 character, 100 taxon matrix of Conde et al. (López-Conde et al. 2017). For the purposes of this justification, we rely on the phylogeny presented in figure s6 of Conde et al. (López-Conde et al. 2017). Phylogenetically optimized characters uniting †*N. oxfordiensis* with Pan-*Pleurodira* are: distinct anal notch of xiphiplastron present (164:1 in Conde et al. (López-Conde et al. 2017)), gular, only one scute (168:1 in Conde et al. (López-Conde et al. 2017)), and ischium attached to plastron by a broad suture (225:1 in Conde et al. (López-Conde et al. 2017)).

Stratigraphic Horizon: Viñales, Cuba; Jagua Vieja Member of the Jagua Formation, Oxfordian (de la Fuente and Iturralde-Vinent 2001), 161.5 to 154.8 Ma (Gradstein et al. 2021).

Equivalent Fossil Calibrations: None. Several other species of †*Notoemys* do occur in slightly younger geological units (López-Conde et al. 2017).

Fossil tip age: 154.8 Ma. The node calibration was created such that 97.5% of the probability distribution fell before 154.8 Ma.

†*Kappachelys okurai*

Justification of Placement: †*Kappachelys okurai* calibrates the MRCA of *Trionychia* (specifiers: *Apalone spinifera*, *Carettochelys insculpta*) in our node-dating analyses. The placement of †*K. okurai* within *Trionychia* on the stem (but not crown) of *Trionychidae* is supported by morphological characters, including: 20 or fewer peripheral bones, partial loss of keratinous scutes in the carapace, and loss of the costal-peripheral suture (Hirayama et al. 2013; 2017).

Stratigraphic Horizon: Oarashidani locality, Shiramine area, Hakusan City, Ishikawa Prefecture, Honshu, Japan; lower member of the Akaiwa Formation, Tetori Group (2017). We use 131.7 Ma, which is the upper bound for the age of the perhaps contemporaneous lower member of the Kuwajima Formation (2017).

bioRxiv preprint doi: https://doi.org/10.64898/2026.06.28.735080; this version posted June 29, 2026. The copyright holder for this preprint (which was not certified by peer review) is the author/funder, who has granted bioRxiv a license to display the preprint in perpetuity. It is made available under a CC-BY 4.0 International license.

Equivalent Fossil Calibrations: Unclear; some fossils from China and Japan may belong to trionychids of similar age (2017).

Fossil tip age: 131.7 Ma. The node calibration was created such that 97.5% of the probability distribution fell before 131.7 Ma.

†"*Trachemys*" *antiqua*

Justification of Placement: †"*Trachemys*" *antiqua* calibrates the MRCA of *Emydidae* (specifiers: *Cuora amboinensis*, *Mauremys reevesii*) in our node-dating analyses. The placement of †"*T.*" *antiqua* in *Emydidae* is supported by phylogenetic analysis of morphological characters, including the 170 character, 125 taxon matrix of Vlachos (Vlachos 2018). For the purposes of this justification, we rely on the phylogeny presented in figure 1 of Vlachos (Vlachos 2018). Phylogenetically optimized apomorphies uniting †"*T.*" *antiqua* with *Emydidae* are: anterior musk duct foramina absent (134:1 in Vlachos (Vlachos 2018)), posterior musk duct foramina absent (135:1 in Vlachos (Vlachos 2018)).

Stratigraphic Horizon: Various from North Dakota, South Dakota, Colorado, and Nebraska, USA; Chadron and White River formations, Priabonian Stage of the Eocene to Brule Formation, Rupelian Stage of the Oligocene (Vlachos 2018). We follow Joyce et al. (Joyce et al. 2013) in using an age of 32.0 Ma for this taxon.

Equivalent Fossil Calibrations: None.

Fossil tip age: 32.0 Ma. The node calibration was created such that 97.5% of the probability distribution fell before 32.0 Ma.

†*Protorosaurus speneri*

Justification of Placement: †*Protorosaurus speneri* calibrates the MRCA of *Archelosauria* (specifiers: *Alligator mississippiensis*, *Chelydra serpentina*) in our node-dating analyses. The placement of †*P. speneri* within *Archelosauria* as a member of Pan-*Archosauria* is supported by parsimony and Bayesian phylogenetic analyses of morphological characters, including the 144 character, 17 taxon matrix of Gottmann-Quesada and Sander,(Gottmann-Quesada and Sander 2009) the 219 character, 40 taxon matrix of Ezcurra et al. (Ezcurra et al. 2014), the 822 character, 157 taxon matrix of Ezcurra et al. (Ezcurra et al. 2020), and the 307 character, 42 taxon matrix of Spiekman et al. (Spiekman et al. 2021). For the purposes of this justification, we

bioRxiv preprint doi: https://doi.org/10.64898/2026.06.28.735080; this version posted June 29, 2026. The copyright holder for this preprint (which was not certified by peer review) is the author/funder, who has granted bioRxiv a license to display the preprint in perpetuity. It is made available under a CC-BY 4.0 International license.

rely on the phylogeny presented in figure 33 of Spiekman et al. (Spiekman et al. 2021). Phylogenetically optimized apomorphies supporting placement of †*P. spleneri* within *Archelosauria* in Pan-*Archosauria* are:

maxilla alveolar margin concave in lateral view (25:1 in Spiekman et al. (Spiekman et al. 2021)), medially expanded supratemporal fossa resulting in mediolaterally narrow parietal table (83:1 in Spiekman et al. (Spiekman et al. 2021)), anterior portion of surangular-angular suture appears anteroposteriorly concave ventrally in lateral view (158:0 in Spiekman et al. (Spiekman et al. 2021)), retroarticular process upturned (163:1 in Spiekman et al. (Spiekman et al. 2021)), diapophyses and parapophyses of anterior to mid-postaxial cervical vertebrae on different processes nearly touch (188:2 in Spiekman et al. (Spiekman et al. 2021)), anterior margin of neural spine of anterior to mid-postaxial cervical vertebrae anterodorsally inclined at more than 60° from horizontal plane (194:1 in Spiekman et al. (Spiekman et al. 2021)), long transverse processes of mid-dorsal vertebrae (207:1 in Spiekman et al. (Spiekman et al. 2021)), shallow fossa lateral to base of neural spine of each dorsal vertebra (209:1 in Spiekman et al. (Spiekman et al. 2021)), coracoid moderately expanded posteriorly in lateral view (232:1 in Spiekman et al. (Spiekman et al. 2021)), proximal portion of interclavicle subtriangular to diamond-shaped (237:0 in Spiekman et al. (Spiekman et al. 2021)), weak (<35°) torsion between proximal and distal ends of humerus (241:1 in Spiekman et al. (Spiekman et al. 2021)), and entepicondylar foramen of humerus absent (246:1 in Spiekman et al. (Spiekman et al. 2021)).

Stratigraphic Horizon: Middridge and Quarrington Quarries, Durham, England, and various localities in central Germany (Gottmann-Quesada and Sander 2009; Spiekman et al. 2021); Marl Slate and Kupferschiefer Formation, Wuchiapingian Stage of Permian. We use an age of 256.0 Ma (Simões et al. 2018).

Equivalent Fossil Calibrations: None, although several 'protorosaurian'-grade pan-archosaurs of unclear phylogenetic placement are known from the latest Permian, as are the earliest archosauriforms (Ezcurra et al. 2014; Ezcurra et al. 2014; Bernardi et al. 2015; Spiekman et al. 2021).

Fossil tip age: 256.0 Ma. The node calibration was created such that 97.5% of the probability distribution fell before 256.0 Ma.

†*Teleocrater rhadinus*

bioRxiv preprint doi: https://doi.org/10.64898/2026.06.28.735080; this version posted June 29, 2026. The copyright holder for this preprint (which was not certified by peer review) is the author/funder, who has granted bioRxiv a license to display the preprint in perpetuity. It is made available under a CC-BY 4.0 International license.

Justification of Placement: †*Teleocrater rhadinus* calibrates the MRCA of *Archosauria* (specifiers: *Alligator mississippiensis*, *Columbia livia*) in our node-dating analyses. The placement of †*T. rhadinus* within *Archosauria* and in Pan-*Aves* is supported by parsimony and Bayesian analyses of morphological characters, including the 606 character, 86 taxon matrix of Nesbitt et al. (Nesbitt et al. 2017), the 822 character, 157 taxon matrix of Ezcurra et al. (Ezcurra et al. 2020), and the

823 character, 159 taxon matrix of Nesbitt et al. (Nesbitt et al. 2023). For the purposes of this justification, we rely on the phylogeny presented in figure 21 of Nesbitt et al. (Nesbitt et al. 2023). Because no character state optimizations were provided by Nesbitt et al. (Nesbitt et al. 2023) for the placement of †*T. rhadinus* within *Archosauria* as a member of Pan-*Aves*, we reran the matrix presented in the paper using TNT v 1.5 (Goloboff and Catalano 2016) to optimize apomorphies; we ran an initial Wagner search over 1000 replicates and default parameters for ratchet, tfuse, drift, and sectorial search followed by traditional bisection reconnection branch swapping over 100,000 trees. We summarized the topologies in a strict consensus and then recorded apomorphies of *Archosauria*. Phylogenetically optimized apomorphies supporting placement of †*T. rhadinus* within *Archosauria* are: antorbital fossa on the lateral surface of the maxilla present on the horizontal process (= posterior process) of the maxilla ventral to the antorbital fenestra, but not reaching the posteroventral corner of the maxillary contribution to the border of the opening (54:2 in Nesbitt et al. (Nesbitt et al. 2023)), fibula transverse width at mid-length distinctly narrower than transverse width of the tibia (529:1 in Nesbitt et al. (Nesbitt et al. 2023)), fibula distal end in lateral view rounded or flat (symmetrical; 532:1 in Nesbitt et al. (Nesbitt et al. 2023)), ventral notch between main body of calcaneum and the calcaneal tuber present (552:1 in Nesbitt et al. (Nesbitt et al. 2023)), and articular facets for the fibula and astragalus on calcaneum separated (555:1 in Nesbitt et al. (Nesbitt et al. 2023)).

Stratigraphic Horizon: Ruhuhu Basin, Tanzania; Lifua Member, Manda Beds;(Nesbitt et al. 2017) Anisian, 247.2 to 242.0 Ma (Gradstein et al. 2021).

Equivalent Fossil Calibrations: The aphanosaurs †*Dongusuchus efremovi* and †*Yarasuchus deccanensis* are equivalent calibrations (Nesbitt et al. 2017; Nesbitt et al. 2023).

Fossil tip age: 242.0 Ma. The node calibration was created such that 97.5% of the probability distribution fell before 242.0 Ma.

bioRxiv preprint doi: https://doi.org/10.64898/2026.06.28.735080; this version posted June 29, 2026. The copyright holder for this preprint (which was not certified by peer review) is the author/funder, who has granted bioRxiv a license to display the preprint in perpetuity. It is made available under a CC-BY 4.0 International license.

†*Deinosuchus rugosus*

Justification of Placement: †*Deinosuchus rugosus* calibrates the most recent common ancestor of *Crocodylia* (specifiers: *Alligator mississippiensis*, *Crocodylus porosus*) in our node-dating analyses. The placement of †*D. rugosus* as a member of crown *Crocodylia* on the stem *Caimaninae* is supported by parsimony analyses of morphological characters, including the 163 character, 83 taxon matrix of Cossette and Brochu (Cossette and Brochu 2020). The use of †*D. rugosus* as a calibration point for the crocodylian crown clade is conservative; we employed a conservative approach due to the unstable placement of several putatively older crown crocodylian species in phylogenetic analyses of morphological characters (Brochu 1997; Bona et al. 2018; Lee and Yates 2018; Mateus et al. 2019; Melstrom and Irmis 2019; Rio and Mannion 2021; Darlim et al. 2022). For the purposes of this justification, we rely on the phylogeny presented in figure 28 of Cossette and Brochu.(Cossette and Brochu 2020). Phylogenetically optimized apomorphies uniting †*D. rugosus* (=*schwimmeri*) and equivalent-aged gobidontan crocodylians with Pan-*Alligatoridae* are: subequal anterior processes of surangular (54:1 in Cossette and Brochu (Cossette and Brochu 2020)), foramen aerum of articular inset from retroarticular process margin (63:1 in Cossette and Brochu (Cossette and Brochu 2020)), all dentary teeth occlude lingual to maxillary teeth (82:0 in Cossette and Brochu (Cossette and Brochu 2020)), high quadratojugal spine sits between posterior and superior infratemporal fenestra angles (121:1 in Cossette and Brochu (Cossette and Brochu 2020)).

Stratigraphic Horizon: The oldest definitive occurrences of †*Deinosuchus rugosus* are from the Ellisdale Site, Monmouth County, New Jersey (Schwimmer 2002; Milàn et al. 2010; Brownstein 2018; Brownstein 2019) (basal Marshalltown Formation, Campanian, between 75.7 and 71.2 Ma, though possibly older)(Miller et al. 2004; Denton 2022) and various localities corresponding to the Tar Heel Formation of North Carolina (Campanian, 78.7-74.5 Ma)(Schwimmer 2002; Cossette and Brochu 2020; Anon). Equivalently old fossils of †*Deinosuchus* are also known from western North America (Schwimmer 2002; Mohler et al. 2021).

Equivalent Fossil Calibrations: Various, dependent on phylogenetic topology (see earlier).

Fossil tip age: 75.7 Ma. The node calibration was created such that 97.5% of the probability distribution fell before 75.7 Ma.

†*Asteriornis maastrichtensis*

bioRxiv preprint doi: https://doi.org/10.64898/2026.06.28.735080; this version posted June 29, 2026. The copyright holder for this preprint (which was not certified by peer review) is the author/funder, who has granted bioRxiv a license to display the preprint in perpetuity. It is made available under a CC-BY 4.0 International license.

Justification of Placement: †*Asteriornis maastrichtensis* calibrates the MRCA of *Neognathae* (specifiers: *Columbia livia*, *Gallus gallus*) in our node-dating analyses. The placement of †*A. maastrichtensis* within crown *Neognathae* as a member of Pan-*Galloanserae* is supported by parsimony and Bayesian phylogenetic analyses of morphological characters, including the 297 character, 39 taxon matrix of Field et al. (Field et al. 2020), including as modified by Houde et al. (Houde et al. 2023) and Crane et al. (Crane et al. 2024) and the 234 character, 49 taxon matrix of Benito et al. (Benito, Kuo, et al. 2022) (as modified from Benito et al. (Benito, Chen, et al. 2022) and thus Torres et al. (Torres et al. 2021)). For the purposes of this justification, we rely on the phylogeny presented in figure 5 of Crane et al. (Crane et al. 2024). Crane et al. (Crane et al. 2024) find †*A. maastrichtensis* to be a possible pan-galliform in agreement with some trees resolved by Field et al. (Field et al. 2020); however, we conservatively use this taxon to calibrate the MRCA of *Neognathae* rather than *Galloanserae* given its placement on the stem of *Galloanserae* in other analyses (Benito, Chen, et al. 2022; Benito, Kuo, et al. 2022). Phylogenetically optimized apomorphies uniting †*A. maastrichtensis* with Pan-*Neognathae* are: poorly define impression for cranial cruciate ligament (221:1 in Crane et al. (Crane et al. 2024)). Phylogenetically optimized characters uniting †*A. maastrichtensis* with *Neognathae* are: subcapitular tubercle on quadrate present (50:1 in Crane et al. (Crane et al. 2024)). Phylogenetically optimized characters uniting †*A. maastrichtensis* with *Galloanserae* are: long, narrow, and dorsally oriented medial mandibular process (68:1 in Crane et al. (Crane et al. 2024)).

Stratigraphic Horizon: CBR-Romontbos Quarry, Eben-Emael, Liège, Belgium(Field et al. 2020); Maastricht Formation, late Maastrichtian, between approximately 69.5 and 66.5 Ma (Kroth et al. 2024). Field et al. (Field et al. 2020) reported that the Valkenburg Member of the Maastricht Formation had yielded holotype and only known specimen of †*Asteriornis maastrichtensis*. However, the division of the Maastricht Formation into six members has recently been challenged (Kroth et al. 2024). Because the Valkenburg Member generally corresponds to lower horizons in the Maastricht Formation, we use an age of 68.0 Ma, which is approximately the midpoint of the Cretaceous portion of this unit (see (Kroth et al. 2024)).

Equivalent Fossil Calibrations: None.

Fossil tip age: 68.0 Ma. The node calibration was created such that 97.5% of the probability distribution fell before 68.0 Ma.

bioRxiv preprint doi: https://doi.org/10.64898/2026.06.28.735080; this version posted June 29, 2026. The copyright holder for this preprint (which was not certified by peer review) is the author/funder, who has granted bioRxiv a license to display the preprint in perpetuity. It is made available under a CC-BY 4.0 International license.

†*Conflicto antarcticus*

Justification of Placement: †*Conflicto antarcticus* calibrates the MRCA of crown *Galloanserae* (specifiers: *Anas platyrhynchos*, *Gallus gallus*) in our node-dating analyses. The placement of †*C. antarcticus* in crown *Galloanserae* in Pan-*Anseriformes* is supported by Bayesian and parsimony phylogenetic analyses of morphological characters, including 290 character, 51 taxon matrix of Tambussi et al. (Tambussi et al. 2019), the 297 character, 39 taxon matrix of Field et al. (Field et al. 2020), including as modified by Houde et al. (Houde et al. 2023) and Crane et al. (Crane et al. 2024), the 719 character, 41 taxon matrix of Musser and Clarke (Musser and Clarke 2024), and the 100 character, 34 taxon matrix of McInerney et al. (McInerney et al. 2024). For the purposes of this justification, we rely on the phylogeny presented in figure 14 of Tambussi et al. (Tambussi et al. 2019). Phylogenetically optimized apomorphies uniting †*Conflicto antarcticus* with Pan-*Anseriformes* are: caudally divergent borders of premaxilla (3:0 in Tambussi et al. (Tambussi et al. 2019)), processus basipterygoidei on sides of rostrum parasphenoidale anterior to caudal end of rostrum (28:2 in Tambussi et al. (Tambussi et al. 2019)), fonticuli present on occipital region of skull (30:1 in Tambussi et al. (Tambussi et al. 2019)), distinct foramen pneumaticum caudomediale present on processus oticus of the quadrate (51:1 in Tambussi et al. (Tambussi et al. 2019)), long, narrow interramal region on mandibular rami (69:1 in Tambussi et al. (Tambussi et al. 2019)), processus ventralis of the axis pronounced (75:1 in Tambussi et al. (Tambussi et al. 2019)), foramen enclosed by processus transversus and processus articularis caudalis of third cervical vertebra absent (76:0 in Tambussi et al. (Tambussi et al. 2019)), os metacarpale minor extends dorsal to ventral rim of trochlea carpalis (159:0 in Tambussi et al. (Tambussi et al. 2019)), length of fornix between the os metacarpale minor and major exceeds width (171:1 in Tambussi et al. (Tambussi et al. 2019)), sulcus patellaris of femur narrow and deep (213:1 in Tambussi et al. (Tambussi et al. 2019)), and pronounced medial displacement of the condylus medialis compared to facies medialis on tibiotarsus (244:1 in Tambussi et al. (Tambussi et al. 2019)).

Stratigraphic Horizon: Southern part of Seymour Island, near Punta Pingüino on the Weddel Sea, Antarctica; López de Bertodano Formation, Danian Stage of the Paleogene, 64.0–61.1 Ma (Tambussi et al. 2019).

Equivalent Fossil Calibrations: None.

bioRxiv preprint doi: https://doi.org/10.64898/2026.06.28.735080; this version posted June 29, 2026. The copyright holder for this preprint (which was not certified by peer review) is the author/funder, who has granted bioRxiv a license to display the preprint in perpetuity. It is made available under a CC-BY 4.0 International license.

Fossil tip age: 61.1 Ma. The node calibration was created such that 97.5% of the probability distribution fell before 61.1 Ma.

†*Eogrus* spp.

Justification of Placement: †*Eogrus* spp. calibrates the MRCA of *Paleognathae* (specifiers: *Struthio camelus*, *Tinamus guttatus*) in our node-dating analyses. The placement of †*Eogrus* spp. in crown *Paleognathae* as a member of Pan-*Struthio* is supported by the following characters, including the presence of shortened pedal phalanges II-2 through II-4, presence of a tubercle on the distal end of the tibiotarsus, an elongated tarsometatarsus, and the reduction of the trochlea of metatarsal II such that it is smaller than the trochlea of metatarsal IV (Mayr and Zelenkov 2021).

Stratigraphic Horizon: Various localities, Shara Murun region, China; Ulan Shireh and Irdin Manha formations, middle Eocene (Russell 1987; Clarke et al. 2005; Mayr and Zelenkov 2021). We assign an age of 47.8 Ma following the age of the oldest †*Eogrus* fossils in figure 7 of Mayr and Zelenkov (Mayr and Zelenkov 2021).

Equivalent Fossil Calibrations: Dependent on phylogeny. Members of the †*Palaeotididae* and †*Lithornithidae* are older than †*Eogrus* but have highly unstable positions in paleognath phylogeny relative to the crown group (Mayr 2015; Nesbitt and Clarke 2016; Worthy et al. 2017; Field et al. 2020; Houde et al. 2023; McInerney et al. 2024; Musser and Clarke 2024).

Fossil tip age: 47.8 Ma. The node calibration was created such that 97.5% of the probability distribution fell before 47.8 Ma.

†*Tsidiiyazhi abini*

Justification of Placement: †*Tsidiiyazhi abini* calibrates the MRCA of Pan-*Coliiformes* with other *Neoaves* (specifiers: *Colius striatus*, *Cuculus canorus*). This is a conservative fossil calibration that results from (1) the lack of Cretaceous to earliest Danian neoavian fossils assignable to that clade based on phylogenetic analyses of morphological characters, and (2) the high degree of uncertainty surrounding the relationships of neoavian orders in our phylogeny that causes the topology presented in Figure 1 to disagree with previous analyses of avian relationships (Hackett et al. 2008; Jarvis et al. 2014; Prum et al. 2015; Reddy et al. 2017; Kimball et al. 2019; Kuhl et al. 2021; Berv et al. 2024; Stiller et al. 2024). We refer the reader to recently published phylogenies of *Neoaves* based on larger avian-specific loci sets for resolution

bioRxiv preprint doi: https://doi.org/10.64898/2026.06.28.735080; this version posted June 29, 2026. The copyright holder for this preprint (which was not certified by peer review) is the author/funder, who has granted bioRxiv a license to display the preprint in perpetuity. It is made available under a CC-BY 4.0 International license.

of backbone neoavian relationships (Stiller et al. 2024). The placement of †*T. abini* in *Neoaves* as a member of Pan-*Coliiformes* is supported by parsimony analyses of morphological characters, including the 111 character, 48 taxon matrix of Ksepka et al. (Ksepka et al. 2017) and the 146 character, 62 taxon matrix of Ksepka et al. (Ksepka et al. 2019) For the purposes of this justification, we rely on the phylogeny presented in figure 2 of Ksepka et al. (Ksepka et al. 2019). Because no character state optimizations were provided by Ksepka et al. (Ksepka et al. 2019) for the placement of †*T. abini* in *Neoaves* as a member of Pan-*Coliiformes*, we reran the matrix presented in the paper using TNT v 1.5 (Goloboff and Catalano 2016) to optimize apomorphies; we ran an initial Wagner search over 1000 replicates and default parameters for ratchet, tfuse, drift, and sectorial search followed by traditional bisection reconnection branch swapping over 100,000 trees. We summarized the topologies in a strict consensus and then recorded apomorphies of Pan-*Coliiformes.* Phylogenetically optimized apomorphies uniting †*T. abini* with Pan-*Coliiformes* are: processus acrocoracoideus of coracoid with hooked outline (49:1 in Ksepka et al. (Ksepka et al. 2019)) and crista trochanteris of femur does not surpass level of the femoral head (96:1 in Ksepka et al. (Ksepka et al. 2019)).

Stratigraphic Horizon: NMMNH locality L-6898, West Flank of Torreon Wash, San Juan Basin, Sandoval County, New Mexico, USA; Ojo Encino Member, Nacimiento Formation (Ksepka et al. 2017), between 62.221–62.517 Ma (Ogg et al. 2016; Ksepka et al. 2017).

Equivalent Fossil Calibrations: None.

Fossil tip age: 62.221 Ma. The node calibration was created such that 97.5% of the probability distribution fell before 62.221 Ma.

†*Waimanu manneringi*

Justification of Placement: †*Waimanu manneringi* calibrates the MRCA of *Pygoscelis adeliae* and *Phalacrocorax brasilianus* in our node-dating analyses. The placement of †*W. manneringi* in Pan-*Spheniscidae* is supported by Bayesian and parsimony phylogenetic analyses of morphological characters, including the 148 character, 49 taxon matrix of Slack et al.(Slack et al. 2006), the 181 character, 50 taxon matrix of Ksepka et al. (Ksepka et al. 2006), the 75 character, 50 taxon matrix of Mayr et al. (Mayr, De Pietri, et al. 2017), the 274 character, 66 taxon matrix of Thomas et al. (Thomas et al. 2020), the 281 character, 74 taxon matrix of Cole et al. (Cole et al. 2022), the 279 character, 66 taxon matrix of Ksepka et al. (Ksepka et al. 2023), and the 281

bioRxiv preprint doi: https://doi.org/10.64898/2026.06.28.735080; this version posted June 29, 2026. The copyright holder for this preprint (which was not certified by peer review) is the author/funder, who has granted bioRxiv a license to display the preprint in perpetuity. It is made available under a CC-BY 4.0 International license.

character, 70 taxon matrix of Thomas et al.(Thomas et al. 2023) (including as modified by Mayr et al. (Mayr, Pietri, et al. 2017)). For the purposes of this justification, we rely on figure S3 of Slack et al. (Slack et al. 2006). Phylogenetically optimized characters uniting †*W. manneringi* with Pan-*Sphenisciformes* are: thoracic vertebrae variably not heterocoelous (57:0 in Slack et al. (Slack et al. 2006)), synsacrum composed of 11 to 12 ankylozed vertebrae (91:1 in Slack et al. (Slack et al. 2006)), and poorly developed hypotarsal crests and grooves on tarsometatarsus (103:0 in Slack et al. (Slack et al. 2006)).

Stratigraphic Horizon: Waipara River, New Zealand; Basal Waipara Greensand, Paleocene, 60.5–61.6 Ma (Slack et al. 2006).

Equivalent Fossil Calibrations: None, though several other stem-penguins are known from slightly younger horizons in the Waipara Greensand (Slack et al. 2006; Mayr, Pietri, et al. 2017).

Fossil tip age: 60.5 Ma. The node calibration was created such that 97.5% of the probability distribution fell before 60.5 Ma.

†*Protopsephurus liui*

Justification of Placement: †*Protopsephurus liui* calibrates the MRCA of *Acipenseriformes* (*Polyodon spathula*, *Acipenser ruthenus*) in our node-dating analyses. The placement of †*P. liui* in *Acipenseriformes* on the stem of *Polyodontidae* is supported by parsimony phylogenetic analyses of morphological characters, including the 44 character, 10 taxon matrix of Grande et al. (Grande et al. 2002) and the 62 character, 18 taxon matrix of Hilton et al. (Hilton et al. 2011). For the purposes of this justification, we rely on the phylogeny presented in figure 20 of Grande et al. (Grande et al. 2002). Phylogenetically optimized characters supporting the placement of †*P. liui* as a member of Pan-*Polyodontidae* are: body scales tiny and non-overlapping or absent (4:1 in Grande et al. (Grande et al. 2002)), dorsal end of quadratojugal with anterior projection (9:1 in Grande et al. (Grande et al. 2002)), elongate dorsal median rostral bones present (21:1 in Grande et al. (Grande et al. 2002)), well developed anterior and posterior divisions of fenestra longitudinalis present (24:1 in Grande et al. (Grande et al. 2002)), stellate bones supporting paddle present (26:1 in Grande et al. (Grande et al. 2002)), and parietals extend posterior to posttemporals (32:1 in Grande et al. (Grande et al. 2002)).

Stratigraphic Horizon: Songzhangzi and Fanzhangzi area, Lingyuan City, western Liaoning Province, China; Dawangzhangzi Beds, Yixian Formation, Jehol Group, Aptian-Albian (Hilton

bioRxiv preprint doi: https://doi.org/10.64898/2026.06.28.735080; this version posted June 29, 2026. The copyright holder for this preprint (which was not certified by peer review) is the author/funder, who has granted bioRxiv a license to display the preprint in perpetuity. It is made available under a CC-BY 4.0 International license.

et al. 2011). We use an age of 121.4 Ma, which represents the boundary for these two stages (Gradstein et al. 2021).

Equivalent Fossil Calibrations: None.

Fossil tip age: 121.4 Ma. The node calibration was created such that 97.5% of the probability distribution fell before 121.4 Ma.

†*Watsonulus eugnathoides*

Justification of Placement: †*Watsonulus eugnathoides* calibrates the MRCA of *Holostei* (specifiers: *Amia calva*, *Lepisosteus osseus*) in our node-dating analyses. The placement of †*W. eugnathoides* in *Holostei* as a member of Pan-*Amia* is supported by Bayesian and parsimony analyses of morphological characters, including the 90 character, 37 taxon matrix of López-Arbarello (López-Arbarello 2012), the 96 character, 28 taxon matrix of Xu et al. (Xu et al. 2014), the 265 character, 93 taxon matrix of Giles et al. (Giles et al. 2017), the 339 character, 99 taxon matrix of López-Arbarello and Sferco (López-Arbarello and Sferco 2018), the 275 character, 97 taxon dataset of Argyriou et al. (Argyriou et al. 2018), the 224 character, 60 taxon dataset of Xu (Xu 2019), and the 300 character, 117 taxon matrix of Argyriou et al. (Argyriou et al.). For the purposes of this justification, we rely on the phylogeny presented in figure 11 of Argyriou et al. (Argyriou et al.). Phylogenetically optimized apomorphies uniting †*W. eugnathoides* with Pan-*Amia* are: posterior maxillary notch present (75:1 in Argyriou et al. (Argyriou et al.)), single supramaxilla (76:1 in Argyriou et al. (Argyriou et al.)), accessory hyoid element involved in jaw joint (97:1 in Argyriou et al. (Argyriou et al.)), foramen for abducens nerve (VI) level with optic foramen (II) (135:1 in Argyriou et al. (Argyriou et al.)), anterior ossification of ceratohyal unconstructed (220:0), and jaw articulation posterior to orbit (280:0 in Argyriou et al. (Argyriou et al.)).

Stratigraphic Horizon: Ambilombe, Madagascar; Sakamena Formation, Early Triassic, Induan (Olsen 1984). We use an age of 251.2 Ma, which is the base of the Induan (Gradstein et al. 2021), following previous studies (Benton et al. 2015).

Equivalent Fossil Calibrations: None.

Fossil tip age: 251.2 Ma. The node calibration was created such that 97.5% of the probability distribution fell before 251.2 Ma.

bioRxiv preprint doi: https://doi.org/10.64898/2026.06.28.735080; this version posted June 29, 2026. The copyright holder for this preprint (which was not certified by peer review) is the author/funder, who has granted bioRxiv a license to display the preprint in perpetuity. It is made available under a CC-BY 4.0 International license.

†*Atractosteus falipoui*

Justification of Placement: †*Atractosteus falipoui* calibrates the MRCA of *Lepisosteidae* (specifiers: *Atractosteus tropicus*, *Lepisosteus osseus*) in our node-dating analyses. The placement of †*A. falipoui* in crown *Lepisosteidae* as a member of Pan-*Atractosteus* is supported by Bayesian and parsimony analyses of morphological characters, including the 105 character, 32 taxon matrix of Grande (Grande 2010) (including as modified by Brito et al. (Brito et al. 2017) and Brownstein and Lyson) (Brownstein and Lyson 2022), and the 104 character, 38 taxon matrix of Brownstein et al. (Chase Doran Brownstein et al. 2023). For the purposes of this justification, we rely on the phylogeny presented in supplementary figure 3 of Brito et al. (Brito et al. 2017). Phylogenetically optimized apomorphies that unite †*A. falipoui* with Pan-*Atractosteus* are: collective shape of laterally expanded vomerine heads form a shape roughly like an equilateral triangle (40:1 in Grande (Grande 2010)/Brito et al. (Brito et al. 2017)), tooth plates associated with second and third hypobranchials (80:1 in Grande (Grande 2010)/Brito et al. (Brito et al. 2017)), and anterior end of first coronoid curves medially and expands broadly to a flat symphysis (104:1 in Grande (Grande 2010)/Brito et al. (Brito et al. 2017)).

Stratigraphic Horizon: Various localities, Kem Beds, Morocco; Auoufous Formation, Cenomanian (Grande 2010). We use an age of 97.2 Ma following previous studies (Brownstein and Lyson 2022; Chase Doran Brownstein et al. 2023).

Equivalent Fossil Calibrations: None.

Fossil tip age: 97.2 Ma. The node calibration was created such that 97.5% of the probability distribution fell before 97.2 Ma.

†*Ichthyemidion vidali*

Justification of Placement: †*Ichthyemidion vidali* calibrates the MRCA of *Elopomorpha* (specifiers: *Megalops atlanticus*, *Anguilla anguilla*) in our node-dating analyses. The placement of †*I. vidali* in *Elopomorpha* on the stem of *Elopiformes* is supported by Bayesian and parsimony analyses, including the morphological + molecular dataset of Dornburg et al. (Dornburg et al. 2015), the 74 character, 27 taxon dataset of Figueiredo et al. (Hernández-Guerrero et al. 2021), and the 75 character, 30 taxon dataset of Hernández-Guerrero et al. (Hernández-Guerrero et al. 2021). Given the considerable uncertainty surrounding the ages and phylogenetic positions of Late Jurassic to Early Cretaceous members of *Elopomorpha* and its constituent clades (Arratia

bioRxiv preprint doi: https://doi.org/10.64898/2026.06.28.735080; this version posted June 29, 2026. The copyright holder for this preprint (which was not certified by peer review) is the author/funder, who has granted bioRxiv a license to display the preprint in perpetuity. It is made available under a CC-BY 4.0 International license.

2000; de Figueiredo et al. 2012; Dornburg et al. 2015; Davesne et al. 2021), this is a conservative fossil calibration. For the purposes of this justification, we rely on the phylogeny presented in figure 6 of Hernández-Guerrero et al. (Hernández-Guerrero et al. 2021). Because no character state optimizations were provided by Hernández-Guerrero et al. (Hernández-Guerrero et al. 2021) for the placement of †*I. vidali* in *Elopomorpha* as a member of Pan-*Elopiformes*, we reran the matrix presented in the paper using TNT v 1.5 (Goloboff and Catalano 2016) to optimize apomorphies; we ran an initial Wagner search over 1000 replicates and default parameters for ratchet, tfuse, drift, and sectorial search followed by traditional bisection reconnection branch swapping over 100,000 trees. We summarized the topologies in a strict consensus and then recorded apomorphies of *Elopomorpha*, Pan-*Elopiformes*, and the clade containing †*I. vidali* + *Elopiformes*. Phylogenetically optimized apomorphies that unite †*I. vidali* with *Elopomorpha* are: compound neural arch over Pu1 and U1 present (62:1 in Hernández-Guerrero et al. (Hernández-Guerrero et al. 2021)), fringing fulcra present (62:1 in Hernández-Guerrero et al. (Hernández-Guerrero et al. 2021)), and pelvic splint present (69:1 in Hernández-Guerrero et al. (Hernández-Guerrero et al. 2021)). Phylogenetically optimized apomorphies that unite †*I. vidali* with Pan-*Elopiformes* are: elongate jaw as bearing numerous villiform teeth (37:1 in Hernández-Guerrero et al. (Hernández-Guerrero et al. 2021)). Phylogenetically optimized apomorphies that unite †*I. vidali* and *Elopiformes* are: preopercle posteroventrally expanded (33:1 in Guerrero et al. (Hernández-Guerrero et al. 2021)).

Stratigraphic Horizon: El Montsec, Lérida Province, Spain; Berriasian–Barremian (Dornburg et al. 2015). We use an age of 125.0 Ma, which is the traditional upper bound of the Barremian (Gradstein et al. 2021).

Equivalent Fossil Calibrations: Dependent on the phylogenetic resolution of fossils placed stemward in Pan-*Elopiformes* in the phylogeny presented by Hernández-Guerrero et al. (Hernández-Guerrero et al. 2021).

Fossil tip age: 125.0 Ma. The node calibration was created such that 97.5% of the probability distribution fell before 125.0 Ma.

†*Laeliichthys ancestralis*

Justification of Placement: †*Laeliichthys ancestralis* calibrates the MRCA of *Osteoglossiformes* (specifiers: *Pantodon buchholzi*, *Osteoglossum bicirrhosum*) in our node-

bioRxiv preprint doi: https://doi.org/10.64898/2026.06.28.735080; this version posted June 29, 2026. The copyright holder for this preprint (which was not certified by peer review) is the author/funder, who has granted bioRxiv a license to display the preprint in perpetuity. It is made available under a CC-BY 4.0 International license.

dating analyses. The placement of †*L. ancestralis* in crown *Osteoglossiformes* is supported by Bayesian and parsimony analyses of morphological characters, including the 87 character, 28 taxon matrix of Brito et al.,(Brito et al. 2020) the 96 character, 34 taxon matrix of Capobianco et al. (Capobianco et al. 2025), and the 96 character, 56 taxon matrix of Capobianco and Friedman (Capobianco and Friedman 2024). For the purposes of this justification, we rely on the phylogeny presented in figure s1 of Brito et al. (Brito et al. 2020). Phylogenetically optimized apomorphies that unite †*L. ancestralis* with crown *Osteoglossiformes* are: supratemportal commissure passes through the parietals (10:1 in Brito et al. (Brito et al. 2020)), dorsal arm of posttemporal less than 1.5x as long as ventral arm (57:0 in Brito et al. (Brito et al. 2020)), and epurals absent (68:2 in Brito et al.(Brito et al. 2020)).Phylogenetically optimized apomorphies that unite †*L. ancestralis* with Pan-*Notopteridae* are: extrascapular reduced and irregularly shaped (2:1 in Brito et al. (Brito et al. 2020)), frontal anterior margin width equal to posterior margin width (3:1 in Brito et al. (Brito et al. 2020)), nasal bones meet at midline (6:2 in Brito et al. (Brito et al. 2020)), subopercle absent (35:2 in Brito et al. (Brito et al. 2020)), posterior portion of maxilla lies on angular (39:0 in Brito et al. (Brito et al. 2020)), and first infrapharyngobranchial present (53:1 in Brito et al. (Brito et al. 2020)).

Stratigraphic Horizon: Quarries of São José do Geribá farmstead, near Patos de Minas Sanfranciscana Basin, Minas Gerais, Brazil (Brito et al. 2020); Upper Quiricó Formation, Barremian-Aptian (Brito et al. 2020; Celerino de Carvalho and Santucci 2024), which range from 125.77 to 113.0 Ma (Gradstein et al. 2021). We use an age of 113.0 Ma.

Equivalent Fossil Calibrations: None.

Fossil tip age: 113.0 Ma. The node calibration was created such that 97.5% of the probability distribution fell before 113.0 Ma.

†*Tischlingerichthys viohli*

Justification of Placement: †*Tischlingerichthys viohli* calibrates the MRCA of *Otocephala* (specifiers: *Clupea harengus*, *Danio rerio*) in our node-dating analyses. The placement of †*T. viohli* in crown *Otocephala* is supported by parsimony analyses of morphological characters, including the 74 character, 27 taxon dataset of Figueiredo et al. (de Figueiredo et al. 2012) and the 75 character, 31 taxon matrix of L-Recinos et al. (L-Recinos et al. 2023). For the purposes of this justification, we rely on the phylogeny presented in figure 15 of L-Recinos et al. (L-Recinos

bioRxiv preprint doi: https://doi.org/10.64898/2026.06.28.735080; this version posted June 29, 2026. The copyright holder for this preprint (which was not certified by peer review) is the author/funder, who has granted bioRxiv a license to display the preprint in perpetuity. It is made available under a CC-BY 4.0 International license.

et al. 2023). Because no character state optimizations were provided by L-Recinos et al. (L-Recinos et al. 2023) for the placement †*T. viohli* in crown *Otocephala* as a member of Pan-*Ostariophysi*, we reran the matrix presented in the paper using TNT v 1.5 (Goloboff and Catalano 2016) to optimize apomorphies; we ran an initial Wagner search over 1000 replicates and default parameters for ratchet, tfuse, drift, and sectorial search followed by traditional bisection reconnection branch swapping over 100,000 trees. We summarized the topologies in a strict consensus and then recorded apomorphies of *Otocephala* and Pan-*Ostariophysi*. Phylogenetically optimized apomorphies that unite †*T. viohli* with crown *Otocephala* are: retroarticular excluded from jaw joint (42:1 in L-Recinos et al. (L-Recinos et al. 2023)). Phylogenetically optimized apomorphies that unite †*T. viohli* with Pan-*Ostariophysi* are: parietal shorter than wide (2:1 in L-Recinos et al. (L-Recinos et al. 2023)), preopercle posteroventrally expanded (33:1 in L-Recinos et al. (L-Recinos et al. 2023)), ventral margin of quadrate horizontal (39:1 in L-Recinos et al. (L-Recinos et al. 2023)), and first preural and first ural fused (59:1 in L-Recinos et al. (L-Recinos et al. 2023)).

Stratigraphic Horizon: Mühlheim, Bavaria, Germany; Mörnsheim Formation, lower Tithonian (Arratia 2000). We use an age of 151.5 Ma, which is the maximum upper bound on the age of the Tithonian (Gradstein et al. 2021).

Equivalent Fossil Calibrations: None.

Fossil tip age: 151.5 Ma. The node calibration was created such that 97.5% of the probability distribution fell before 151.5 Ma.

†*Rubiesichthys gregalis*

Justification of Placement: †*Rubiesichthys gregalis* calibrates the MRCA of *Ostariophysi* (specifiers: *Chanos chanos*, *Danio rerio*) in our node-dating analyses. The placement of †*R. gregalis* in *Ostariophysi* as a member of *Gonorynchiformes* is supported by Bayesian and parsimony analyses of morphological characters, including the 94 character, 22 taxon matrix of Grande and Poyato-Ariza (GRANDE and POYATO-ARIZA 1999), the 106 character, 14 taxon matrix of Ribiero et al. (Ribeiro et al. 2018), the 130 character, 22 taxon matrix of Grande et al. (Diogo 2009) (including as modified by Amaral et al. (Amaral et al. 2013)), and the 128 character, 20 taxon matrix used with molecular data by Near et al. (Near et al. 2014). For the purposes of this justification, we rely on the phylogeny presented in figure 7.10 of Grande et al.

bioRxiv preprint doi: https://doi.org/10.64898/2026.06.28.735080; this version posted June 29, 2026. The copyright holder for this preprint (which was not certified by peer review) is the author/funder, who has granted bioRxiv a license to display the preprint in perpetuity. It is made available under a CC-BY 4.0 International license.

(Diogo 2009). Phylogenetically optimized apomorphies that unite †*R. gregalis* with *Gonorynchiformes* are: orbitosphenoid absent (1:1 in Grande et al. (Diogo 2009)), parietals completely separated by supraoccipital (13:2 in Grande et al. (Diogo 2009)), reduced, flat, and blade-like parietals (15:1 in Grande et al. (Diogo 2009)), teeth absent in premaxilla, maxilla, dentary (20:1 in Grande et al. (Diogo 2009)), premaxillary ascending process absent (24:1 in Grande et al. (Diogo 2009)), and rib on third vertebral centrum wide and short (82:1 in Grande et al. (Diogo 2009)).

Stratigraphic Horizon: El Montsec and Las Hoyas, Lérida and Cuenca Provinces, Spain (Poyato-Ariza 1996a; Poyato-Ariza 1996b); Berriasian–Barremian (Dornburg et al. 2015). El Montsec is Barriasian through lower Barremian, whereas Las Hoyas is Barremian (Gil-Delgado et al. 2023; Marugán-Lobón et al. 2023). We use an age of 121.4 Ma, which is the upper bound of the Barremian (Gradstein et al. 2021).

Equivalent Fossil Calibrations: †*Gordichthys conquensis*, also from the Early Cretaceous of Spain, is a Pan-*Chanos* and is an equivalent fossil calibration (Poyato-Ariza 1996a).

Fossil tip age: 121.4 Ma. The node calibration was created such that 97.5% of the probability distribution fell before 121.4 Ma.

†*Santanichthys diasii*

Justification of Placement: †*Santanichthys diasii* calibrates the MRCA of *Otophysi* (specifiers: *Corydoras julii*, *Danio rerio*) in our node-dating analyses. The placement of †*S. diasii* in *Otophysi* is supported by the following characters: basisphenoid absent, dermopalatine absent, expanded dorsomedial portion of anterior neural arches, the expansion of the anterior supraneural, modification of the first neural arch into the scaphium, modification of the second neural arch into the intercalarium, first four centra shortened, and modification of the third centrum rib parapophysis into the tripus (Filleul and Maisey 2004).

Stratigraphic Horizon: Araripe Basin, northeastern Brazil; Santana Formation, Albian Stage of the Early Cretaceous.(Filleul and Maisey 2004) Following previous studies, we use an age of 112.5 Ma (Chase Doran Brownstein et al. 2023; Brownstein and Near 2024).

Equivalent Fossil Calibrations: None.

Fossil tip age: 112.5 Ma. The node calibration was created such that 97.5% of the probability distribution fell before 112.5 Ma.

bioRxiv preprint doi: https://doi.org/10.64898/2026.06.28.735080; this version posted June 29, 2026. The copyright holder for this preprint (which was not certified by peer review) is the author/funder, who has granted bioRxiv a license to display the preprint in perpetuity. It is made available under a CC-BY 4.0 International license.

†*Wilsonium brevipinne*

Justification of Placement: †*Wilsonium brevipinne* calibrates the MRCA of *Cypriniformes* (specifiers: *Gyrinocheilus aymonieri*, *Danio rerio*) in our node-dating analyses. The placement of †*W. brevipinne* in *Cypriniformes* as a member of Pan-*Catostomidae* is supported by parsimony analysis of morphological characters, including the 83 character, 71 taxon matrix of Liu et al (Liu 2021). For the purposes of this justification, we rely on the phylogeny presented in figure 6 of Liu et al (Liu 2021). Phylogenetically optimized apomorphies that unite †*W. brevipinne*

with Pan-*Catostomidae* are: pharyngeal toothplate falcate (Liu 2021).

Stratigraphic Horizon: North Fork of the Similkameen River, Pleasant Valley, British Columbia, Canada; Allenby Formation, Eocene (Liu 2021). Following Bagley et al. (Bagley et al. 2018), we use an age of 48.88 Ma for this taxon.

Equivalent Fossil Calibrations: Various pan-catostomids, see Bagley et al. (Bagley et al. 2018).

Fossil tip age: 48.88 Ma. The node calibration was created such that 97.5% of the probability distribution fell before 48.88 Ma.

†*Siluroidei* indet.

Justification of Placement: Indeterminate siluroid fossils calibrate the MRCA of *Siluriformes* (specifiers: *Corydoras julii*, *Silurus asotus*) in our node-dating analyses. The placement of these fossils, which include the apomorphic pectoral fin spines of catfishes, in the crown clade as potential representatives of living catfish families is supported by the presence of large, compressed, and hooked posterior dentations and the absence of anterior dentations (Alves et al. 2019). Although Alves et al. (Alves et al. 2019) suggested affinities with several families of siluroid siluriform for these elements, we conservatively use them to calibrate the catfish crown group as they were not included in a phylogenetic analysis.

Stratigraphic Horizon: Santo Anastácio, São Paulo State, Brazil; Adamantina Formation, Turonian-Santonian Stages of the Late Cretaceous (Alves et al. 2019), which range from 93.9 to 83.6 Ma (Alves et al. 2019).

Equivalent Fossil Calibrations: Various isolated remains, see Alves et al. (Alves et al. 2019) for a review.

bioRxiv preprint doi: https://doi.org/10.64898/2026.06.28.735080; this version posted June 29, 2026. The copyright holder for this preprint (which was not certified by peer review) is the author/funder, who has granted bioRxiv a license to display the preprint in perpetuity. It is made available under a CC-BY 4.0 International license.

Fossil tip age: 83.6 Ma. The node calibration was created such that 97.5% of the probability distribution fell before 83.6 Ma.

†*Cretazeus rinaldi*

Justification of Placement: †*Cretazeus rinaldi* calibrates the MRCA of *Zeiogadaria* (specifiers: *Gadus morhua*, *Zeus faber*) in our node-dating analyses. The placement of †*C. rinaldi* in *Zeiogadaria* as a member of Pan-*Zeiformes* is supported by parsimony analyses of morphological characters, including the 107 character, 43 taxon matrix of Tyler and Santini (Tyler and Santini 2005) (including as modified by Davesne et al. (Davesne et al. 2017)) and the combined dataset (including 105 characters and 40 taxa) of Grande et al. (Grande et al. 2018). For the purposes of this justification, we rely on the phylogeny presented in figure 7 of Tyler and Santini (Tyler and Santini 2005). Phylogenetically optimized apomorphies uniting †*C. rinaldi* with Pan-*Zeiformes* are: multiple serrations present on lower border of dentary (22:2 in Tyler and Santini (Tyler and Santini 2005)), parahypural slightly removed from and does not embrace urostylar centrum (52:1 in Tyler and Santini (Tyler and Santini 2005)), absence of uroneural (54:1 in Tyler and Santini (Tyler and Santini 2005)), and two anal fin spines present (100:2 in Tyler and Santini (Tyler and Santini 2005)).

Stratigraphic Horizon: Nardò, Italy; Santonian-Maastrichtian Stages of the Late Cretaceous (Tyler and Santini 2005; Santaquiteria et al. 2021). Although traditionally considered to be Campanian-Maastrichtian, there is some evidence that the fossils at Nardò are actually older, from the Santonian (Santaquiteria et al. 2021). As such, we assign a basal Campanian age of 83.0 Ma to this fossil (Santaquiteria et al. 2021). Note that we assign an age of 86.0 Ma to †*Gasterorhamposus zuppichini*, as fossils of that taxon, which also appears at Nardò, may extend into the Santonian (Santaquiteria et al. 2021).

Equivalent Fossil Calibrations: None.

Fossil tip age: 83.0 Ma. The node calibration was created such that 97.5% of the probability distribution fell before 83.0 Ma.

†*Iridopristis parrisi*

Justification of Placement: †*Iridopristis parrisi* calibrates the MRCA of *Beryciformes* (specifiers: *Beryx splendens*, *Sargocentron rubrum*) in our node-dating analyses. The placement

bioRxiv preprint doi: https://doi.org/10.64898/2026.06.28.735080; this version posted June 29, 2026. The copyright holder for this preprint (which was not certified by peer review) is the author/funder, who has granted bioRxiv a license to display the preprint in perpetuity. It is made available under a CC-BY 4.0 International license.

of †*Iridopristis parrisi* in *Beryciformes* as a member of Pan-*Holocentridae* is supported by Bayesian and parsimony analysis of morphological characters, including the 82 character, 18 taxon matrix of Andrews et al. (Andrews et al. 2023). Phylogenetically optimized apomorphies that unite †*I. parrisi* with Pan-*Holocentridae* are: transverse crest present on supraoccipital (2:1 in Andrews et al. (Andrews et al. 2023)), separate opening present for orbital branch of supraorbital sensory canal (3:1 in Andrews et al. (Andrews et al. 2023)), ventrolateral wing-like expansion of parasphenoid present (6:1 in Andrews et al. (Andrews et al. 2023)), anterior ceratohyal imperforate (8:1 in Andrews et al. (Andrews et al. 2023)), presence of deep notches along ventral margin of anterior ceratohyal (9:1 in Andrews et al. (Andrews et al. 2023)), alveolar platform near symphysis of dentary expanded and overhangs lateral margin of bone (27:1 in Andrews et al. (Andrews et al. 2023)), and presence of a concave premaxillary tooth gap at premaxillary median margin ventral to the premaxillary ascending process (28:1 in Andrews et al. (Andrews et al. 2023)).

Stratigraphic Horizon: Inversand Quarry (now the Edelman Fossil Park), Sewell, New Jersey, USA; Main Fossiliferous Layer, Hornerstown Formation, earliest Danian, Paleocene (Andrews et al. 2023), approximately 66.02 Ma (Gradstein et al. 2021).

Equivalent Fossil Calibrations: None.

Fossil tip age: 66.02 Ma. The node calibration was created such that 97.5% of the probability distribution fell before 66.02 Ma.

†*Gasterorhamposus zuppichini*

Justification of Placement: †*Gasterorhamposus zuppichini* calibrates the MRCA of *Syngnathiformes* (specifiers: *Aulostomus maculatus*, *Hippocampus erectus*) in our node-dating analyses. The placement of †*G. zuppichini* in *Syngnathiformes* is supported by parsimony and Bayesian analyses of morphological characters, including the 101 character, 36 taxon dataset of Brownstein (C D Brownstein 2023). For the purposes of this justification, we rely on the phylogeny presented in figure S2 of Brownstein (C D Brownstein 2023). Phylogenetically optimized apomorphies that unite †*G. zuppichini* with *Syngnathiformes* are: infraorbital bones do not form a complete ventral ring (6:1 in Brownstein (C D Brownstein 2023)), parietals absent (9:1 in Brownstein (C D Brownstein 2023)), preopercle articular socket for interhyal present

bioRxiv preprint doi: https://doi.org/10.64898/2026.06.28.735080; this version posted June 29, 2026. The copyright holder for this preprint (which was not certified by peer review) is the author/funder, who has granted bioRxiv a license to display the preprint in perpetuity. It is made available under a CC-BY 4.0 International license.

(34:1 in Brownstein (C D Brownstein 2023)), and pelvic spines absent (77:1 in Brownstein (C D Brownstein 2023)).

Stratigraphic Horizon: Porto Selvaggio, Lecce province, Italy; "Calcari di Melissano", Santonian-Campanian (Santaquiteria et al. 2021). We use a Santonian age here (86.0 Ma).

Equivalent Fossil Calibrations: None.

Fossil tip age: 86.0 Ma. The node calibration was created such that 97.5% of the probability distribution fell before 86.0 Ma.

†*Uylyaichthys eugeniae*

Justification of Placement: †*Uylyaichthys eugeniae* calibrates the MRCA of *Carangioidei* (specifiers: *Caranx ignobilis*, *Coryphaena hippurus*) in our node-dating analyses. The placement of †*Uylyaichthys eugeniae* in *Carangioidei* as a member of *Carangidae* is supported by the following morphological characters: gap between last two anal fin spines (Prokofiev 2002).

Stratigraphic Horizon: Uylya-Kushlyuk locality, 2 km northeast of Uylya-Kushlyuk village, Turkmenistan; Danata Formation, uppermost Thanetian-lowermost Ypresian (Paleocene to Eocene) (Prokofiev 2002; Ghezelayagh et al. 2022). Following Ghezelayagh et al. (Ghezelayagh et al. 2022), we use an age of 55.8 Ma.

Equivalent Fossil Calibrations: †*Archaeus oblongus* and †*Trachicaranx tersus*, both from the same geological unit as †*Uylyaichthys eugeniae*, are equivalent fossil calibrations (Prokofiev 2002).

Fossil tip age: 55.8 Ma. The node calibration was created such that 97.5% of the probability distribution fell before 55.8 Ma.

†*Eocoelopoma portentosum*

Justification of Placement: †*Eocoelopoma portentosum* calibrates the MRCA of *Scombriformes* (specifiers: *Nomeus gronovii*, *Thunnus albacares*) in our node-dating analyses. The placement of †*E. portentosum* in *Scombriformes* as a member of *Scombroidea* is supported by the following morphological characters: premaxillary fangs absent, caudal hypurostegy present, two epurals present, and single uroneural present (Friedman et al. 2019).

bioRxiv preprint doi: https://doi.org/10.64898/2026.06.28.735080; this version posted June 29, 2026. The copyright holder for this preprint (which was not certified by peer review) is the author/funder, who has granted bioRxiv a license to display the preprint in perpetuity. It is made available under a CC-BY 4.0 International license.

Stratigraphic Horizon: Uylya-Kushlyuk locality, 2 km northeast of Uylya-Kushlyuk village, Turkmenistan; Danatinsk Formation, Thanetian to Ypresian (Friedman et al. 2019). We use an age of 54.17 Ma following previous studies (Monsch and Bannikov 2011).
Equivalent Fossil Calibrations: Various, see Monsch and Bannikov (Monsch and Bannikov 2011) for a review of Paleogene scombroids from northern Eurasia.
Fossil tip age: 54.17 Ma. The node calibration was created such that 97.5% of the probability distribution fell before 54.17 Ma.

†*Phyllopharyngodon longipinnis*
Justification of Placement: †*Phyllopharyngodon longipinnis* calibrates the node representing the MRCA of *Labridae* (specifiers: *Thalassoma bifasciatum*, *Scarus ghobban*). The placement of †*P. longipinnis* within *Labridae* is supported by the following morphological characters: single, posteriorly oriented supraneural; fused pharyngeal jaw present; scales cycloid (Bellwood 1990). The placement of †*P. longipinnis* in Pan-*Hypsigenyinae* is supported by the following morphological character: phyllodonty (Bellwood 1990).
Stratigraphic Horizon: Monte Bolca, near Verona, Italy; Early-late Ypresian, Eocene, Paleogene 48.96 to 48.5 Ma (Friedman and Carnevale 2018).
Equivalent Fossil Calibrations: †*Bellwoodilabrus landinii*, also from Monte Bolca, is an equivalent fish calibration (Bannikov and Carnevale 2010).
Fossil tip age: 48.5 Ma. The node calibration was created such that 97.5% of the probability distribution fell before 48.5 Ma.

†*Caruso brachysomus*
Justification of Placement: †*Caruso brachysomus* calibrates the node representing the MRCA of *Lophioidei* (specifiers: *Antennarius striatus*, *Lophius piscatorius*) in our node-dating analyses. For a detailed discussion of morphological characters uniting †*C. brachysomus* with crown *Lophioidei* and *Lophiidae*, see Brownstein et al. (Brownstein, Zapfe, et al. 2024).
Stratigraphic Horizon: Monte Bolca, near Verona, Italy: early-late Ypresian, Eocene, Paleogene 48.96 to 48.5 Ma (Friedman and Carnevale 2018).
Equivalent Fossil Calibrations: †*Eosladenia caucasica* from the Middle Eocene of the northern Caucasus (A.f 2004), the Monte Bolca antennariids †*Eophryne barbutii* (Carnevale and Pietsch

bioRxiv preprint doi: https://doi.org/10.64898/2026.06.28.735080; this version posted June 29, 2026. The copyright holder for this preprint (which was not certified by peer review) is the author/funder, who has granted bioRxiv a license to display the preprint in perpetuity. It is made available under a CC-BY 4.0 International license.

2009), †*Histionotophorus bassani*, and †*Orrichthys longimanus* (Carnevale and Pietsch 2010), and the Monte Bolca ogcocephalid †*Tarkus squirei* (Carnevale and Pietsch 2011) are all equivalent fossil calibrations.

Fossil tip age: 48.5 Ma. The node calibration was created such that 97.5% of the probability distribution fell before 48.5 Ma.

†*Moclaybalistes danekrus*

Justification of Placement: †*Moclaybalistes danekrus* calibrates the MRCA of *Tetraodontoidei* (specifiers: *Mola mola*, *Pseudobalistes fuscus*) in our node-dating analyses. The placement of †*M. danekrus* in *Tetraodontoidei* is supported by parsimony analyses of morphological characters, including the 210 character, 56 taxon dataset of Santini and Tyler (SANTINI and TYLER 2003). For the purposes of this justification, we rely on the phylogeny presented in figure 4a of Santini and Tyler (SANTINI and TYLER 2003). Phylogenetically optimized apomorphies that unite †*M. danekrus* with *Tetraodontoidei* are: infraorbitals absent (24:1 in Santini and Tyler (SANTINI and TYLER 2003)), sensory groove on dentary absent (35:1 in Santini and Tyler (SANTINI and TYLER 2003)), mouth gape moderate to small (39:1 in Santini and Tyler (SANTINI and TYLER 2003)), skull bone lateral line canals absent (66:1 in Santini and Tyler (SANTINI and TYLER 2003)), thick caniniform teeth (69:3 in Santini and Tyler (SANTINI and TYLER 2003)), second dorsal spine elongated (159:2 in Santini and Tyler (SANTINI and TYLER 2003)), first anal fin pterygiophore positioned along posterior edge of first caudal haemal spine (164:1 in Santini and Tyler (SANTINI and TYLER 2003)), single anal fin pterygiophore in first interhaemal space (166:1 in Santini and Tyler (SANTINI and TYLER 2003)), procurrent caudal fin rays absent (178:1 in Santini and Tyler (SANTINI and TYLER 2003)), and 10 to 14 anal fin rays present (209:2 in Santini and Tyler (SANTINI and TYLER 2003)).

Stratigraphic Horizon: Various localities, Denmark; Fur Formation, Ypresian Stage of the Eocene, 56.0 Ma (Benton and Donoghue 2007).

Equivalent Fossil Calibrations: None.

Fossil tip age: 56.0 Ma. The node calibration was created such that 97.5% of the probability distribution fell before this 56.0 Ma.

bioRxiv preprint doi: https://doi.org/10.64898/2026.06.28.735080; this version posted June 29, 2026. The copyright holder for this preprint (which was not certified by peer review) is the author/funder, who has granted bioRxiv a license to display the preprint in perpetuity. It is made available under a CC-BY 4.0 International license.

II. Supplementary Results and Discussion.

General Observations.

We conducted phylogenetic analyses of the exon dataset at different levels of completeness (all 1105 exons, and a 75% complete matrix) using both multispecies coalescent (Zhang et al. 2018) and concatenation approaches to explore how alternative strategies affected inference of the jawed vertebrate tree. Generally, we inferred similar phylogenies across different sequence matrix completeness levels and analytical methods (Extended Data Figure 1). However, phylogenies inferred from the concatenated matrix inferred widely-accepted relationships, such as monophyly of *Syngnathiformes* and the placement of *Leiognathidae* within *Eupercaria*, that the ASTRAL-III multispecies coalescent phylogenies failed to resolve. Among some of the deepest divergences in jawed vertebrates, we also observe incongruence across trees inferred using different methodologies. For example, salamanders (*Caudata*) and caecilians (*Gymnophiona*) are consistently inferred as sister clades in phylogenies built using ASTRAL-III, whereas the conventional arrangement of amphibian relationships in which frogs (*Anura*) are sister to *Caudata* is recovered in phylogenies inferred from the concatenated sequences under maximum likelihood (Extended Data Figure 1). These and other conflicts are discussed in the following sections. Generally, gene and site concordance factors, bootstrap supports, and branch lengths (in substitutions per site) show similar linear relationships across analyses of the exon matrix at different completeness levels (Extended Data Figure 2). Across the jawed vertebrate phylogenies that we infer, coalescent support values, anomalous branch counts, and gene and site concordance factors do not substantially decrease in value across different matrix completeness levels (Extended Data Figure 3). Jawed vertebrate clades sampled in our phylogeny show clear patterns in time; a pronounced drop in nodal support and branch lengths (both in substitutions per site and in millions of years) is associated with the Cretaceous-Paleogene Mass Extinction (Figure 3; Figure 4; Extended Data Figure 7). Heirarchical clustering of jawed vertebrate node ages (nodes sampled are all older than 56 Ma) also shows a clear split between nodes dating to before the Late Cretaceous and from the Late Cretaceous onward, suggesting a signature of divergence associated with the Cretaceous Terrestrial Revolution and Cretaceous-Paleogene Mass Extinction.

bioRxiv preprint doi: https://doi.org/10.64898/2026.06.28.735080; this version posted June 29, 2026. The copyright holder for this preprint (which was not certified by peer review) is the author/funder, who has granted bioRxiv a license to display the preprint in perpetuity. It is made available under a CC-BY 4.0 International license.

Resolution of Earliest Divergences in Sarcopterygii.

Our phylogenetic analyses highlight several regions of uncertainty in early jawed vertebrate relationships. We highlight two key results: the resolution of a monophyletic group containing coelacanths (*Latimeria*) and lungfishes *(Dipnoi*) that forms the sister clade to *Tetrapoda*, and the uncertainty surrounding the living sister clade to other amphibians. We consistently resolve a lungfish-coelacanth sister lineage relationship with strong nodal support across all phylogenetic analyses (Figure 2; Extended Data Figure 1). This result contrasts with many phylogenomic analyses of sarcopterygians built using genome-scale data (Chen et al. 2015; Irisarri and Meyer 2016; Irisarri et al. 2017; Hime et al. 2020; Meyer et al. 2021; Wang et al. 2021; Schartl et al. 2024). However, we note that only one of these analyses fully sampled all major early divergences in jawed vertebrates, including the four oldest-diverging clades in ray-finned fishes (*Actinopterygii*): *Polypteridae*, *Acipenseriformes*, *Holostei*, and *Oseanacephala*,(Chen et al. 2015) and even this study does not sample *Amia*, which diverged from the only other living holosteans, gars (*Lepisosteidae*), at least 247.2 million years ago (given the position of †*Watsonulus eugnathoides* on the stem of *Amia*; see fossil calibration justification). This result is also not due to uncertain resolution of conflict among genes and sites, as gene and site concordance factors estimated for the coelacanth-lungfish node are comparable to or higher than widely accepted clades, including many ray-finned fish orders, laurasiatherian mammals, lepidosaurs, and archosaurs (Figure 2). Our result highlights that the classic question of what lobe-finned fish clade is the living sister to tetrapods (Brinkmann et al. 2004; Shan and Gras 2011; Irisarri and Meyer 2016; Takezaki and Nishihara 2017; Meyer et al. 2021) is arguably still unresolved and suggests that capturing all deep divergences in jawed vertebrates affects inference of this region of the phylogeny. We also note that the rapid successive divergences of coelacanths, lungfishes, and tetrapods suggested by the fossil record and supported by molecular phylogenies (Zhu et al. 2001; Irisarri et al. 2017; Wang et al. 2021; Cui et al. 2022; Schartl et al. 2024)(Figure 1) also provide a mechanism by which factors like incomplete lineage sorting and introgression interfere with phylogenetic resolution. In our main phylogeny (Figure 1), the age of crown *Sarcopterygii* is 444.02 Ma (95% highest posterior density, HPD: 413.95, 467.73 Ma) and the age of the lungfish-coelacanth split is 417.71 Ma (95% HPD: 383.17, 445.54 Ma), which is 270 kya younger than 97.5% of the prior distribution as given by the fossil †*Youngolepis*

bioRxiv preprint doi: https://doi.org/10.64898/2026.06.28.735080; this version posted June 29, 2026. The copyright holder for this preprint (which was not certified by peer review) is the author/funder, who has granted bioRxiv a license to display the preprint in perpetuity. It is made available under a CC-BY 4.0 International license.

praecursor (see earlier), though the 95% highest posterior density interval overlaps considerably with this prior specification.

The Earliest Divergence in Amphibia.

We find uncertain relationships among the three living lineages of amphibians. As noted above, the resolution of two alternative hypotheses of living amphibian relationships, the *Procera* (*Anura*, (*Caudata*, *Gymnophiona*)) and the *Batrachia* (*Gymnophiona*, (*Anura*, *Caudata*)) differs according to whether a multispecies coalescent or a concatenated approach is employed (Extended Data Figure 1). Either hypothesis is supported by bootstrap or coalescent values of 100% in trees in which *Batrachia* or *Procera* is resolved (Figure 2, Extended Data Figure 1). Different analyses of genome scale data have highlighted inadvertent paralog inclusion and considerable gene discordance as causes for this uncertainty.(Siu-Ting et al. 2019; Hime et al. 2020) Our results are generally supportive of these hypothesized drivers of uncertain relationships among the three major living clades of amphibians. Our inference of Permo-Triassic divergences of these three lineages also supports the more recent timescale of amphibian origination proposed by Hime et al. (Hime et al. 2020). In our main time tree, we estimate an age of 263.19 Ma (95% HPD: 239.87, 290.99 Ma) for crown *Amphibia* and an age of 249.94 Ma (95% HPD: 195.86, 283.77 Ma) for crown *Batrachia*. These estimates, which suggest that the amphibian crown diversified over a period of approximately 15 million years in the early Triassic, should be viewed as conservatively young since we use a conservative age for the calibration of crown *Batrachia* (see above). In any case, they support either a latest Permian or early Triassic origin of the three living amphibian clades, which is entirely concordant with the amphibian fossil record (Evans and Borsuk-Bialynicka 1998; Anderson et al. 2008; Schoch et al. 2020; Jones et al. 2022; Kligman et al. 2023).

Uncertainty in Mammal Order-Level Relationships and the Age of Placentals.

The relationships among placental mammals, as well as their timescale of evolution, remain debated, with the principal issues concerning what clade represents the living sister to all other placentals (dos Reis et al. 2012; dos Reis et al. 2014; Tarver et al. 2016; Esselstyn et al. 2017; Álvarez-Carretero et al. 2022), the relationships among laurasiatherians (Tarver et al. 2016; Esselstyn et al. 2017; Foley et al. 2023), and whether placentals represent an earlier Paleogene

bioRxiv preprint doi: https://doi.org/10.64898/2026.06.28.735080; this version posted June 29, 2026. The copyright holder for this preprint (which was not certified by peer review) is the author/funder, who has granted bioRxiv a license to display the preprint in perpetuity. It is made available under a CC-BY 4.0 International license.

diversification (Springer et al. 2003; Wible et al. 2007; Meredith et al. 2011; O'Leary et al. 2013; dos Reis et al. 2014; Phillips and Fruciano 2018; Velazco et al. 2022; Budd and Mann 2023; Foley et al. 2023). Our time-calibrated phylogenies unambiguously infer that the earliest divergences among crown placentals took place during the Late Cretaceous, between 100 and 80 million years ago (Figure 1, Extended Data Figure 4, Extended Data Figure 6). Our main time tree places the MRCA of crown placentals at 111.65 Ma (95% HPD: 92.7, 136.51 Ma). Our ASTRAL-III species trees and our concatenated maximum likelihood phylogenies disagree on what clade forms the living sister to other placental mammals; the former resolve a clade, *Atlantogenata*, containing both *Afrotheria* and *Xenarthra*, whereas the latter resolve *Xenarthra* as the living sister to all other mammals. Our time calibrated phylogeny indicates that the unclear resolution of the living sister lineage of other placentals may be a consequence of a rapid diversification of placentals in the Aptian, as the clade (*Epitheria*) containing *Afrotheria* and *Boreoeutheria* appears at 111.58 Ma (95% HPD: 92.7, 136.51 Ma). Similarly rapid diversification took place in neoavian birds after the Cretaceous-Paleogene Mass Extinction and essentially renders several regions of neoavian phylogeny unresolvable (Hackett et al. 2008; Jarvis et al. 2014; Prum et al. 2015; Reddy et al. 2017; Kuhl et al. 2021; Stiller et al. 2024). As such, resolving the basal split among crown placentals might be nearly impossible even with genomic data and require mammal-specific analyses to maximize the number of orthologous genes available.(Tarver et al. 2016; Esselstyn et al. 2017) Similarly, we are unable to robustly resolve the relationships among four major groups of laurasiatherians: bats (*Chiroptera*), whales, hippos, pigs, bovids, giraffes, camels, and relatives (*Cetoartiodactyla*), horses, tapirs, rhinoceroses, and relatives (*Perissodactyla*), and a clade containing pangolins and carnivorans (*Ferae*). The relationships of these lineages remain unresolved in other phylogenomic analyses of placentals (dos Reis et al. 2012; Tarver et al. 2016; Esselstyn et al. 2017; Foley et al. 2023). It is notable, however, that with the exception of the placement of bats as sister to *Cetoartiodactyla* rather than as the sister clade to *Cetoartiodactyla* + *Ferae* + *Perissodactyla*, our topology is concordant with previous analyses in recovering a sister relationship between *Ferae* and *Perissodactyla* (dos Reis et al. 2012; Tarver et al. 2016; Esselstyn et al. 2017; Álvarez-Carretero et al. 2022; Foley et al. 2023). As in previous analyses, our result supports a 'soft explosive' model of placental mammal diversification in which diversification among most orders occurred in the Cretaceous and diversification of placental order crown clades occurred in the Paleocene

bioRxiv preprint doi: https://doi.org/10.64898/2026.06.28.735080; this version posted June 29, 2026. The copyright holder for this preprint (which was not certified by peer review) is the author/funder, who has granted bioRxiv a license to display the preprint in perpetuity. It is made available under a CC-BY 4.0 International license.

(Meredith et al. 2011; dos Reis et al. 2014; Phillips and Fruciano 2018; Upham et al. 2021; Álvarez-Carretero et al. 2022; Budd and Mann 2023; Foley et al. 2023).

Resolution of Lepidosaur and Archelosaur Relationships.

One classically (Estes et al. 1988; Lee 1993; Gauthier et al. 2012; Hedges 2012; Bever et al. 2015) controversial region of jawed vertebrate phylogeny that has been rather conclusively resolved using genomic data is the phylogeny of living reptile clades. As in previous analyses, our phylogenetic analyses all strongly support the placement of turtles as sister to archosaurs (birds and crocodylians) to form *Archelosauria* (Iwabe et al. 2005; Chiari et al. 2012; Crawford et al. 2012; Hedges 2012; Wang et al. 2013) and the placement of iguanians deep within the living lineages of crown squamates to form a clade with snakes and anguimorphs (*Toxicofera*) (Townsend et al. 2004; Vidal and Hedges 2005; Crawford et al. 2012; Pyron et al. 2013; Zheng and Wiens 2016; Streicher and Wiens 2017; Simões et al. 2018; Burbrink et al. 2020; Singhal et al. 2021; Title et al. 2024). Resolution of these key deep divergences in reptiles is arguably one of the great accomplishments of the phylogenomic program in jawed vertebrates. However, the relationships among the tree major lineages of toxicoferans conflict among our analyses (Figure 2; Extended Data Figure 1; Extended Data Figure 4), as in previous studies, and the resolution of the living sister to snakes therefore remains an open question (Townsend et al. 2004; Vidal and Hedges 2005; Crawford et al. 2012; Pyron et al. 2013; Zheng and Wiens 2016; Streicher and Wiens 2017; Simões et al. 2018; Burbrink et al. 2020; Singhal et al. 2021; Title et al. 2024). Also notable is the latest Permian age of crown *Lepidosauria* estimated in our node-dating analyses (median MRCA age: 263.27 Ma, 95% HPD: 241.13, 285.25 Ma; Figure 1, Extended Data Figure 4, Extended Data Figure 6), which contrasts with the earliest Triassic origin of the lepidosaur crown group found in a number of studies using genomic data (Jones et al. 2013; Burbrink et al. 2020) but is congruent with several time-calibrated phylogenies built using morphological characters (Simões et al. 2018; Brownstein et al. 2022).

Crown Bird Diversification and Phylogenetic Discordance.

The most poorly resolved portion of jawed vertebrate phylogeny in our analyses is undisputably *Neoaves* (Figure 1, Figure S1, Extended Data Figure 4, Extended Data Figure 7). The backbone of *Neoaves* is supported by the lowest average gene and site concordance factors among post-

bioRxiv preprint doi: https://doi.org/10.64898/2026.06.28.735080; this version posted June 29, 2026. The copyright holder for this preprint (which was not certified by peer review) is the author/funder, who has granted bioRxiv a license to display the preprint in perpetuity. It is made available under a CC-BY 4.0 International license.

Cretaceous radiations in our analyses (Extended Data Figure 7), making *Neoaves* an unusually difficult problem. Over the past decade, nearly a dozen phylogenomic analyses have failed to provide a clear resolution of the relationships of crown birds (Hackett et al. 2008; Jarvis et al. 2014; Claramunt and Cracraft 2015; Prum et al. 2015; Reddy et al. 2017; Kimball et al. 2019; Kuhl et al. 2021; Berv et al. 2024; Stiller et al. 2024). This problem is attributable to a combination of phenomena that occurred in birds during the Cretaceous-Paleogene transition, including strong selection for certain ecologies and life histories (Berv and Field 2018; Field et al. 2018; Berv et al. 2024), a rapid episode of body size reduction (Berv and Field 2018) suppressed recombination across long segments of the avian genome (Mirarab et al. 2024), and rapid successive divergences associated with the initial adaptive radiation of *Neoaves* (Figure 1, Figure 2, Figure 3, Extended Data Figure 4, Extended Data Figure 7, Extended Data Figure 8)(Prum et al. 2015; Stiller et al. 2024). Our phylogeny supports the hypothesis that, as with placental mammals, a huge proportion of orthologous genes are needed to resolve avian phylogeny (Stiller et al. 2024); this arguably requires focusing on neoavians to the exclusion of other jawed vertebrates. As with other analyses, our results suggest that only three or four major bird clades survived the K-Pg: *Paleognathae*, *Galloanserae*, and one or two lineages of *Neoaves* (*Mirandornithes*, all others)(Jarvis et al. 2014; Prum et al. 2015; Berv et al. 2024; Stiller et al. 2024). In our analyses, the origin of *Aves* is estimated to lie in the Late Cretaceous, 97.42 Ma (95% HPD: 83.15, 132.22 Ma), and the origin of *Neognathae* (*Neoaves* + *Galloanserae*) lies within the Campanian, 78.88 Ma (95% HPD: 73.32, 84.54 Ma). The relationships of most major neoavian clades remain unresolved in our analysis, and we emphasize that the phylogeny of *Aves* presented in this manuscript is less robust than previously inferred phylogenies that have focused on this clade. Odd relationships, such as the paraphyly of lineages in *Strisores* and *Coraciimorphae*, inferred in our phylogenies are probably caused by the absence of informative sequences for avian phylogeny included in our dataset, an inference that is supported by the low gene and site concordance factors for nodes across *Neoaves*.

Early-Diverging Actinopterygian Phylogeny.

As in previous studies, we find that four major lineages comprise the earliest divergences among ray-finned fishes (*Actinopterygii*): bichirs and Reedfish (*Polypteridae*), sturgeons and paddlefishes (*Acipenseriformes*), gars and bowfins (*Holostei*), and all other ray-finned fishes

bioRxiv preprint doi: https://doi.org/10.64898/2026.06.28.735080; this version posted June 29, 2026. The copyright holder for this preprint (which was not certified by peer review) is the author/funder, who has granted bioRxiv a license to display the preprint in perpetuity. It is made available under a CC-BY 4.0 International license.

(*Teleostei*)(Grande 2010; Near et al. 2012; Betancur-R et al. 2013; Braasch et al. 2016; Hughes et al. 2018; Du et al. 2020; Bi et al. 2021a; Thompson et al. 2021; Mallik et al. 2025). Both analyses in which the multispecies coalescent or concatenation approaches are employed resolve *Polypteridae*, *Acipenseriformes*, and *Holostei* as the successive sister lineages to *Teleostei*; however, some ASTRAL-III trees unite *Polypteridae* and *Acipenseriformes* to the exclusion of other actinopterygians (Extended Data Figure 1). Uncertainty over the earliest divergence in ray-finned fishes has mainly centered around the conflict between morphological and molecular data, in which the latter unites *Polypteridae* and *Acipenseriformes* (Argyriou et al. 2018; Argyriou et al.) or find them in a polytomy including fossils of early actinopterygians (Giles et al. 2017).One previous study of actinopterygian genomes did find only moderate (bootstrap < 100) support for *Actinopteri*, the clade uniting *Acipenseriformes*, *Holostei*, and *Teleostei* (Bi et al. 2021a); like ours, this study sampled several non-ray-finned fish outgroups (Bi et al. 2021b). The ages of these successive divergences are even less clear, as excluding representatives two of these lineages (*Acipenseriformes*, *Holostei*) notable for their exceptionally slow rates of genome sequence evolution (Braasch et al. 2016; Takezaki 2018; Brownstein, MacGuigan, et al. 2024) shift the crown ages of *Actinopterygii* and *Teleostei* forward in time by up to 50 million years (Figure 5; Extended Data Figure 9, Extended Data Figure 10). The exceptionally rapid rates of molecular evolution in teleost fishes (Brownstein, MacGuigan, et al. 2024), which appear to slip towards the root of the ray-finned fish crown clade in our analyses (Extended Data Figure 9, Extended Data Figure 10), probably also play a role in uncertainty surrounding the ages of these divergences, which conflict with the much younger ages of *Actinopterygii* and *Teleostei* implied by the fossil record (Hurley et al. 2006; Near et al. 2012; Friedman 2022). Our results suggest that, in the absence of additional fossil discoveries, the ages of these clades, which contain nearly half of living vertebrate diversity, are unknowable owing to biological variation in the tempo of genome evolution. In contrast, our analyses contribute to consilience around the earliest divergence in teleosts. Recently, some analyses of whole genomes using traditional phylogenetic methodology (Bian et al. 2016; Hao et al. 2020; Takezaki 2021) and synteny analysis (Parey et al. 2023) resolved a clade containing mooneyes and bonytongues (*Osteoglossomorpha*) and eels, tarpons, bonefishes, and halosaurs (*Elopomorpha*) that forms the sister clade to all other teleosts (*Clupeocephala*). However, studies of legacy nuclear and mitochondrial markers (Near et al. 2012; Betancur-R et al. 2013) and at least some analyses of genome-wide sequence data(Hughes

bioRxiv preprint doi: https://doi.org/10.64898/2026.06.28.735080; this version posted June 29, 2026. The copyright holder for this preprint (which was not certified by peer review) is the author/funder, who has granted bioRxiv a license to display the preprint in perpetuity. It is made available under a CC-BY 4.0 International license.

et al. 2018) resolve these lineages as successive sister clades to *Clupeocephala.* Our analyses, which use the same exon set as Hughes et al.,(Hughes et al. 2018) unambiguously resolve a sister lineage relationship between *Elopomorpha* and *Osteoglossomorpha*, a result also obtained in another recent analysis of these loci (Hughes et al. 2021). Thus, across genomic datasets, there is little to no conflict over the split at the base of teleosts.

Ostariophysan Relationships and the Recognition of Cithariniformes.

Another problem in ray-finned fish phylogenetics that our results contribute towards resolving are the relationships of the major freshwater fish clades that form the clade *Ostariophysi.* Analyses of genomic markers, including ultraconserved elements (Chakrabarty et al. 2017; Melo et al. 2022) and exons (Hughes et al. 2021), find the traditional *Characiformes* to be paraphyletic, with the *Citharíniformes* sensu Near and Thacker (Near and Thacker 2024) forming the sister lineage to a clade containing catfishes (*Siluriformes*) and *Characiformes* sensu lato. However, some studies of exon loci regard the traditional placement of *Citharíniformes* as the sister to other *Characiformes* to be more likely (Arcila et al. 2017; Hughes et al. 2018; Betancur-R. et al. 2019). Our analyses unambiguously reject the monophyly of traditional *Characiformes* and place *Citharíniformes* sister to a clade containing *Siluriformes*, *Characiformes* sensu lato, and *Gymnotiformes*, which contains the Neotropical electric fishes (Figure 1, Figure 2). Although the clade containing *Siluriformes* and *Characiformes* sensu lato to the exclusion of other otophysans is always supported by high bootstrap, coalescent, gene, and site concordance factors, the placement of *Gymnotiformes* as the sister lineage to this clade is only moderately supported (coalescent value < 1) in some analyses (Figure 2). As has the divergence at the base of teleosts (Parey et al. 2023), resolving the relationships of order-level clades in *Ostariophysi* would probably benefit from an analysis of genome structural conservation.

The Black Box of Euteleost Relationships.

A problem in vertebrate phylogeny whose lack of resolution has decidedly gone rather unnoticed is the relationships among the major lineages of euteleosts; these differ across essentially every analysis of ray-finned fish phylogeny (Near et al. 2012; Betancur-R et al. 2013; Hughes et al. 2018; Straube et al. 2018; Hughes et al. 2021). Although we find support for several key clades,

bioRxiv preprint doi: https://doi.org/10.64898/2026.06.28.735080; this version posted June 29, 2026. The copyright holder for this preprint (which was not certified by peer review) is the author/funder, who has granted bioRxiv a license to display the preprint in perpetuity. It is made available under a CC-BY 4.0 International license.

including the *Stomiati*, containing the dragonfishes and hatchetfishes (*Stomiiformes*) and smelts (*Osmeriformes*), and the *Salmoniformes*, containing the salmons, charrs, and trouts (*Salmonidae*) and mudminnows and pikes (*Esocidae*), we fail to consistently resolve the positions of marine smelts and barreleyes (*Osmeriformes*) or the galaxiids (*Galaxiidae*) among euteleosts (Figure 2; Extended Data Figure 1). Given that the placement of these lineages might have major implications for reconstructing the ancestral habitat (deep marine, shallow marine, or freshwater)(Miller et al. 2022) of ray-finned fishes, we highlight this region of the tree as ripe for investigation.

Phylogenetic Resolution of Acanthomorpha.

Our analyses provide a degree of consilience around the relationships of spiny-rayed fishes (*Acanthomorpha*). Resolution of this region of ray-finned fish phylogeny is recognized as an important milestone of phylogenomics (Dornburg and Near 2021; Near and Thacker 2024). As in several recent studies of genome-wide markers (Alfaro et al. 2018; Hughes et al. 2018; Hughes et al. 2021; Ghezelayagh et al. 2022), we find that four lineages form the successive four or five divergences among acanthomorphs: beardfishes (*Polymixia*), opahs and oarfishes (*Lampriformes*), codfishes, granadiers, Tube-Eye, oreos, and John Dories (*Zeiogadaria*), and Pirate Perch, trout-perches, and North American cavefishes (*Percopsiformes*)(Figure 1). The positions of *Lampriformes*, *Percopsiformes,* and *Polymixia* change depending on analytical method employed and exon sample used in our analyses (see Figure 2), underscoring the uncertain positions of these lineages (Alfaro et al. 2018; Hughes et al. 2018; Ghezelayagh et al. 2022). Next to diverge are an oddball clade containing flashlightfishes, porcupinefishes, roughies, and fangtooths (*Trachichthyiformes*), the squirrelfishes and soldierfishes (*Holocentridae)*, and the alfonsinos, whalefishes, gibberfishes, and bigscales (*Berycioidei*); the latter two form a clade (*Beryciformes*) in all of our phylogenies produced using concatenated exons and in some of our phylogenies produced using the multispecies coalescent in ASTRAL-III. Although phylogenies that we generated using concatenated exon sequences are consistent with the hypothesis that *Trachichthyiformes* is the sister lineage to *Beryciformes* and other acanthomorphs (*Percomorpha*), as supported by analyses of ultraconserved elements (Ghezelayagh et al. 2022; Brownstein et al. 2025), anchored hybrid enrichment loci (Dornburg et al. 2017), and exons (Hughes et al. 2018; Musilova et al. 2019; Hughes et al. 2021) (though see

bioRxiv preprint doi: https://doi.org/10.64898/2026.06.28.735080; this version posted June 29, 2026. The copyright holder for this preprint (which was not certified by peer review) is the author/funder, who has granted bioRxiv a license to display the preprint in perpetuity. It is made available under a CC-BY 4.0 International license.

(Hughes et al. 2018)), ASTRAL-III phylogenies that we generated find that these three lineages form a clade sister to *Percomorpha*. We fail to recover *Holocentridae* as the sister lineage to *Percomorpha*, in contrast to a previous study of ray-finned fish phylogeny using the same exon loci (Hughes et al. 2018). We consistently find that cusk eels and brotulas (*Ophidiiformes*) and toadfishes *(Batrachoididae*) are the first two lineages of percomorphs to diverge. The relationships of the remaining percomorph lineages break down in the ASTRAL-III topologies, owing largely to the low level of concordance among gene trees (Figure 2). However, the phylogenies built from the maximum likelihood analyses of the concatenated exon sequence set unite seahorses, trumpetfishes, goatfishes, gurnards, and searobins (*Syngnathiformes*) and tunas, mackerels, swallowers, pomfrets, and driftfishes (*Scombriformes*) in a clade that forms the third major divergence in *Percomorpha* (Figure 1). The remaining order-level clades of percomorphs are organized into two major lineages. One, *Eupercaria*, comprises the *Perciformes* (sea basses and basslets, groupers, Antarctic icefishes, darters, sculpins, scorpionfishes, rockfishes, snailfishes, eelpouts, sticklebacks, searobins, and relatives), *Gerreidae* (mojarras), *Labriformes* (wrasses, parrotfishes, stargazers, sandlances, southern sandfishes, sandperches, and torrentfishes), *Centrarchiformes* (sunfishes, black basses, temperate perches, flagtails, knifejaws, kelpfishes, morwongs, and relatives), and *Acanthuriformes* (butterflyfishes, surgeonfishes, pufferfishes, triggerfishes, Mola mola, spadefishes, anglerfishes, drums, rabbitfishes, ponyfishes, Moorish Idol, angelfishes, snappers, grunts, and relatives) (Hughes et al. 2018; Ghezelayagh et al. 2022; Near and Thacker 2024). As in previous studies, *Perciformes* is the sister lineage to the rest of these clades and the position of *Gerreidae* is highly unstable (Alfaro et al. 2018; Hughes et al. 2018; Musilova et al. 2019; Hughes et al. 2021; Ghezelayagh et al. 2022). The other consists of four order-level clades: *Carangiformes* (jacks, trevallies, swordfishes, remoras, Mahi Mahi, flatfishes, flounders, soles, archerfishes, threadfishes, snooks, barracudas, and giant perches), *Synbranchiformes* (swamp eels, betta fishes, and climbing perches), *Atheriniformes* (New World silversides, flying fishes, halfbeaks, ricefishes, pupfishes, killifishes, and relatives), and *Blenniiformes* (blennies, cichlids, damselfishes and anemonefishes, mullets, surfperches, and relatives) (Hughes et al. 2018; Ghezelayagh et al. 2022; Near and Thacker 2024). As in previous studies, we find a Jurassic age for the origin of *Acanthomorpha* and its initial divergences (Figure 1, Extended Data Figure 5), followed by the diversification of most percomorph orders and their constituent lineages throughout the Cretaceous and into the Paleocene (Alfaro et al.

bioRxiv preprint doi: https://doi.org/10.64898/2026.06.28.735080; this version posted June 29, 2026. The copyright holder for this preprint (which was not certified by peer review) is the author/funder, who has granted bioRxiv a license to display the preprint in perpetuity. It is made available under a CC-BY 4.0 International license.

2018; Hughes et al. 2018; Musilova et al. 2019; Ghezelayagh et al. 2022). Along with birds and mammals, acanthomorph fishes show a high degree of phylogenetic discordance associated with diversification occurring around the Cretaceous-Paleogene Mass Extinction (Figure 3, Figure 4, Extended Data Figure 7).

IV. Supplementary Tables.

Table S1. Genome Resources.

Acipenseriformes_Acipenser_ruthenus	GCF_010645085.1
Acipenseriformes_Acipenser_sinensis	Hughes, 2018, PNAS
Acipenseriformes_Polyodon_spathula	GCF_017654505.1
Polypteridae_Erpetoichthys_calabaricus	Hughes, 2018, PNAS
Polypteridae_Polypterus_bichir	Hughes, 2018, PNAS
Polypteridae_Polypterus_endlicheri	Hughes, 2018, PNAS
Sharks_Callorhinchus_milii	GCF_000165045.1
Hydrolagus_affinis	GCA_012026655.1
Sharks_Amblyraja_radiata	GCA_010909765.2
Leucoraja_erinacea	GCF_028641065.1
Sharks_Potamotrygon_leopoldi	https://download.cncb.ac.cn/gwh/Animals/Potamotrygon_leopoldi_white-blotched_river_stingray_GWHAOTN00000000/
Sharks_Pristis_pectinata	GCF_009764475.1
Pristis_pectinata	GCF_009764475.1
Sharks_Chiloscyllium_punctatum	GCA_003427335.1
Sharks_Rhincodon_typus	GCF_001642345.1
Sharks_Carcharodon_carcharias	GCA_017639515.1
Isurus_oxyrinchus	GCA_026770705.1
Sharks_Scyliorhinus_torazame	GCA_003427355.1
Sphyrna_mokarran	GCA_024679065.1
Sarcopterygii_Latimeria_chalumnae	GCA_000225785.1
Sarcopterygii_Neoceratodus_forsteri	GCA_016271365.1
Sarcopterygii_Protopterus_annectens	GCA_019279795.1
Amphibians_Xenopus_tropicalis	GCF_000004195.3
Amphibians_Rhinella_marina	GCA_900303285.1
Amphibians_Nanorana_parkeri	GCF_000935625.1
Amphibians_Rana_catesbeiana	GCA_002284835.2
Amphibians_Geotrypetes_seraphini	GCA_902459505.2
Amphibians_Microcaecilia_unicolor	GCA_901765095.2
Amphibians_Ambystoma_mexicanum	GCA_002915635.2

bioRxiv preprint doi: https://doi.org/10.64898/2026.06.28.735080; this version posted June 29, 2026. The copyright holder for this preprint (which was not certified by peer review) is the author/funder, who has granted bioRxiv a license to display the preprint in perpetuity. It is made available under a CC-BY 4.0 International license.

Desmognathus_wrighti	GCA_030265035.1
Desmognathus_conanti	GCA_030264475.1
Desmognathus_aeneus	GCA_030264635.1
Desmognathus_apalachicolae	GCA_030264955.1
Monotremes_Ornithorhynchus_anatinus	GCA_004115215.4
Monotremes_Tachyglossus_aculeatus	GCA_015852505.1
Marsupials_Monodelphis_domestica	GCF_000002295.2
Gracilinanus_agilis	GCF_016433145.1
Marsupials_Sarcophilus_harrisii	GCF_000189315.1
Dasyurus_viverrinus	GCA_020854095.1
Marsupials_Macropus_eugenii	GCA_000004035.1
Marsupials_Phascolarctos_cinereus	GCF_002099425.1
Vombatus_ursinus	GCF_900497805.2
Nesogale_talazaci	GCA_004026705.1
Orycteropus_afer	GCF_000298275.1
Placentals_Trichechus_manatus	GCF_000243295.1
Placentals_Loxodonta_africana	GCF_000001905.1
Chaetophractus_vellerosus	GCA_004027955.1
Dasypus_novemcinctus	GCF_030445035.1
Myrmecophaga_tridactyla	GCA_004026745.1
Bradypus_variegatus	GCA_004027775.1
Choloepus_didactylus	GCF_015220235.1
Choloepus_hoffmanni	GCA_000164785.2
Placentals_Oryctolagus_cuniculus	GCF_000003625.3
Placentals_Hydrochoerus_hydrochaeris	GCA_004027455.1
Placentals_Mus_musculus	GCF_000001635.26
Placentals_Rattus_norvegicus	GCF_000001895.5
Galeopterus_variegatus	GCA_004027775.1
Placentals_Otolemur_garnettii	GCF_000181295.1
Placentals_Callithrix_jacchus	GCF_000004665.1
Placentals_Macaca_fascicularis	GCA_000222185.1
Placentals_Pongo_abelii	GCF_002880775.1
Placentals_Gorilla_gorilla	GCF_000151905.2
Placentals_Homo_sapiens	GCF_000001405.38
Placentals_Pan_paniscus	GCF_000258655.2
Placentals_Pan_troglodytes	GCF_002880755.1
Placentals_Erinaceus_europaeus	GCF_000296755.1
Solenodon_paradoxus	GCA_004363575.1
Placentals_Sus_scrofa	GCF_000003025.6
Giraffa_camelopardalis	GCA_013496395.1

bioRxiv preprint doi: https://doi.org/10.64898/2026.06.28.735080; this version posted June 29, 2026. The copyright holder for this preprint (which was not certified by peer review) is the author/funder, who has granted bioRxiv a license to display the preprint in perpetuity. It is made available under a CC-BY 4.0 International license.

Okapia_johnstoni	GCA_024291935.2
Placentals_Bos_mutus	GCF_000298355.1
Placentals_Bos_indicus	GCF_000247795.1
Placentals_Bos_taurus	GCF_002263795.1
Placentals_Balaenoptera_acutorostrata	GCF_000493695.1
Placentals_Balaenoptera_bonaerensis	GCA_000978805.1
Placentals_Delphinapterus_leucas	GCF_002288925.1
Placentals_Neophocaena_asiaeorientalis	GCF_003031525.1
Placentals_Tursiops_truncatus	GCF_001922835.1
Placentals_Orcinus_orca	GCF_000331955.2
Placentals_Pteronotus_parnellii	GCA_000465405.1
Placentals_Myotis_lucifugus	GCF_000147115.1
Placentals_Eidolon_helvum	GCA_000465285.1
Placentals_Pteropus_vampyrus	GCF_000151845.1
Placentals_Megaderma_lyra	GCA_004026885.1
Placentals_Rhinolophus_ferrumequinum	GCA_004115295.1
Dicerorhinus_sumatrensis	GCA_014189135.1
Diceros_bicornis	GCF_020826845.1
Placentals_Equus_asinus	GCF_001305755.1
Placentals_Equus_caballus	GCF_002863925.1
Placentals_Equus_przewalskii	GCF_000696695.1
Manis_pentadactyla	GCF_030020395.1
Hyaena_hyaena	GCF_003009895.1
Placentals_Felis_catus	GCF_000181335.3
Placentals_Panthera_tigris_altaica	GCF_000464555.1
Placentals_Panthera_onca	GCA_004023805.1
Placentals_Panthera_pardus	GCF_001857705.1
Placentals_Canis_lupus_familiaris	GCF_000002285.3
Placentals_Ailuropoda_melanoleuca	GCF_000004335.2
Ursus_arctos	GCF_023065955.2
Placentals_Odobenus_rosmarus	GCF_000321225.1
Placentals_Mustela_putorius_furo	GCF_000215625.1
Ailurus_styani	GCA_002007465.1
Lepidosaurs_Sphenodon_punctatus	GCA_003113815.1
Lepidosaurs_Gekko_japonicus	GCF_001447785.1
Lepidosaurs_Paroedura_picta	GCA_003118565.1
Hemicordylus_capensis	GCF_027244095.1
Lepidosaurs_Aspidoscelis_marmoratus	GCA_014337955.1
Lepidosaurs_Salvator_merianae	GCA_003586115.2
Lepidosaurs_Podarcis_muralis	GCF_004329235.1

bioRxiv preprint doi: https://doi.org/10.64898/2026.06.28.735080; this version posted June 29, 2026. The copyright holder for this preprint (which was not certified by peer review) is the author/funder, who has granted bioRxiv a license to display the preprint in perpetuity. It is made available under a CC-BY 4.0 International license.

Lepidosaurs_Zootoca_vivipara	GCF_011800845.1
Lepidosaurs_Lacerta_agilis	GCF_016433145.1
Lepidosaurs_Lacerta_bilineata	GCA_900245895.1
Lepidosaurs_Lacerta_viridis	GCA_900245905.1
Lepidosaurs_Pogona_vitticeps	GCF_900067755.1
Lepidosaurs_Anolis_carolinensis	GCF_000090745.1
Phrynosoma_platyrhinos	GCA_020142125.1
Lepidosaurs_Varanus_komodoensis	GCA_004798865.1
Lepidosaurs_Shinisaurus_crocodilurus	http://gigadb.org/dataset/100315
Lepidosaurs_Dopasia_gracilis	http://gigadb.org/dataset/100119
Heloderma_charlesbogerti	GCA_026122225.1
Lepidosaurs_Python_molurus_bivittatus	GCF_000186305.1
Lepidosaurs_Vipera_berus	GCA_000800605.1
Lepidosaurs_Protobothrops_flavoviridis	GCA_003402635.1
Lepidosaurs_Protobothrops_mucrosquamatus	GCF_001527695.2
Lepidosaurs_Crotalus_horridus	GCA_001625485.1
Lepidosaurs_Crotalus_tigris	GCF_016545835.1
Lepidosaurs_Crotalus_viridis	GCA_003400415.2
Lepidosaurs_Thamnophis_elegans	GCF_009769535.1
Lepidosaurs_Thamnophis_sirtalis	GCF_001077635.1
Lepidosaurs_Thermophis_baileyi	GCA_003457575.1
Lepidosaurs_Ptyas_mucosa	GCA_012654045.1
Lepidosaurs_Pantherophis_guttatus	GCA_001185365.1
Lepidosaurs_Pantherophis_obsoletus	GCA_012654085.1
Lepidosaurs_Ophiophagus_hannah	GCA_000516915.1
Lepidosaurs_Naja_naja	GCA_009733165.1
Lepidosaurs_Laticauda_colubrina	GCA_015471245.1
Lepidosaurs_Laticauda_laticaudata	GCA_004320025.1
Lepidosaurs_Pseudonaja_textilis	GCF_900518735.1
Lepidosaurs_Notechis_scutatus	GCF_900518725.1
Lepidosaurs_Hydrophis_cyanocinctus	GCA_004023725.1
Lepidosaurs_Hydrophis_melanocephalus	GCA_004320005.1
Turtles_Emydura_subglobosa	GCA_007922225.1
Turtles_Podocnemis_expansa	GCA_007922195.1
Turtles_Pelusios_castaneus	GCA_007922175.1
Turtles_Carettochelys_insculpta	GCA_007922185.1
Turtles_Apalone_spinifera	GCA_000385615.1
Turtles_Pelodiscus_sinensis	GCF_000230535.1
Turtles_Chelonia_mydas	GCF_015237465.1

bioRxiv preprint doi: https://doi.org/10.64898/2026.06.28.735080; this version posted June 29, 2026. The copyright holder for this preprint (which was not certified by peer review) is the author/funder, who has granted bioRxiv a license to display the preprint in perpetuity. It is made available under a CC-BY 4.0 International license.

Turtles_Dermochelys_coriacea	GCF_009764565.2
Turtles_Dermatemys_mawii	GCA_007922305.1
Turtles_Chelydra_serpentina	GCA_007922165.1
Turtles_Mauremys_reevesii	GCF_016161935.1
Turtles_Cuora_amboinensis	GCA_004028625.2
Turtles_Cuora_mccordi	GCA_003846335.1
Turtles_Chelonoidis_abingdonii	GCF_003597395.1
Turtles_Gopherus_agassizii	GCA_002896415.1
Turtles_Gopherus_evgoodei	GCF_007399415.2
Turtles_Platysternon_megacephalum	GCA_003942145.1
Turtles_Actinemys_marmorata	GCA_009430475.1
Turtles_Terrapene_carolina	GCF_002925995.2
Turtles_Chrysemys_picta	GCF_000241765.3
Turtles_Trachemys_scripta	GCF_013100865.1
Turtles_Malaclemys_terrapin	GCA_001728815.2
Crocodylians_Alligator_mississippiensis	GCF_000281125.3
Crocodylians_Alligator_sinensis	GCF_000455745.1
Crocodylians_Crocodylus_porosus	GCF_001723895.1
Crocodylians_Gavialis_gangeticus	GCF_001723915.1
Birds_Struthio_camelus_australis	GCF_000698965.1
Birds_Tinamus_guttatus	GCF_000705375.1
Birds_Anas_platyrhynchos	GCF_003850225.1
Birds_Gallus_gallus	GCF_000002315.6
Birds_Pavo_cristatus	GCA_005519975.1
Birds_Meleagris_gallopavo	GCF_000146605.2
Birds_Tetrao_tetrix	GCA_000586395.1
Birds_Picoides_pubescens	GCF_000699005.1
Birds_Merops_nubicus	GCF_000691845.1
Birds_Acanthisitta_chloris	GCF_000695815.1
Birds_Manacus_vitellinus	GCF_001715985.2
Birds_Corvus_brachyrhynchos	GCF_000691975.1
Birds_Ficedula_albicollis	GCF_000247815.1
Birds_Geospiza_fortis	GCF_000277835.1
Birds_Taeniopygia_guttata	GCF_000151805.1
Birds_Chaetura_pelagica	GCF_000747805.1
Birds_Calypte_anna	GCF_000699085.1
Birds_Colius_striatus	GCF_000690715.1
Birds_Nestor_notabilis	GCF_000696875.1
Birds_Melopsittacus_undulatus	GCF_000238935.1
Birds_Apaloderma_vittatum	GCF_000703405.1

bioRxiv preprint doi: https://doi.org/10.64898/2026.06.28.735080; this version posted June 29, 2026. The copyright holder for this preprint (which was not certified by peer review) is the author/funder, who has granted bioRxiv a license to display the preprint in perpetuity. It is made available under a CC-BY 4.0 International license.

Birds_Buceros_rhinoceros	GCF_000710305.1
Birds_Cuculus_canorus	GCF_000709325.1
Birds_Mesitornis_unicolor	GCF_000695765.1
Birds_Columba_livia	GCF_000337935.1
Birds_Pterocles_gutturalis	GCF_000699245.1
Birds_Eurypyga_helias	GCF_000690775.1
Birds_Antrostomus_carolinensis	GCF_000700745.1
Birds_Falco_cherrug	GCF_000337975.1
Birds_Falco_peregrinus	GCA_012488915.1
Birds_Leptosomus_discolor	GCF_000691785.1
Birds_Strix_occidentalis_caurina	GCA_002372975.1
Birds_Tyto_alba	GCF_000687205.1
Birds_Cariama_cristata	GCF_000690535.1
Birds_Cathartes_aura	GCA_000699945.1
Birds_Aquila_chrysaetos	GCF_000766835.1
Birds_Haliaeetus_albicilla	GCF_000691405.1
Birds_Haliaeetus_leucocephalus	GCF_000737465.1
Birds_Phaethon_lepturus	GCF_000687285.1
Birds_Chlamydotis_macqueenii	GCF_000695195.1
Birds_Opisthocomus_hoazin	GCF_000692075.1
Birds_Podiceps_cristatus	GCA_000699545.1
Birds_Phoenicopterus_ruber	GCA_000687265.1
Birds_Tauraco_erythrolophus	GCF_000709365.1
Birds_Balearica_regulorum_gibbericeps	GCF_000709895.1
Birds_Charadrius_vociferus	GCF_000708025.1
Birds_Himantopus_himantopus	GCA_003993805.1
Birds_Recurvirostra_avosetta	GCA_004023745.1
Birds_Gavia_stellata	GCF_000690875.1
Birds_Egretta_garzetta	GCF_000687185.1
Birds_Phalacrocorax_pelagicus	GCA_002173435.1
Birds_Phalacrocorax_carbo	GCF_000708925.1
Birds_Phalacrocorax_harrisi	GCA_002173475.1
Birds_Phalacrocorax_auritus	GCA_002173455.1
Birds_Phalacrocorax_brasilianus	GCA_002174335.1
Birds_Pelecanus_crispus	GCF_000687375.1
Birds_Nipponia_nippon	GCF_000708225.1
Birds_Fulmarus_glacialis	GCF_000690835.1
Birds_Aptenodytes_forsteri	GCF_000699145.1
Birds_Pygoscelis_adeliae	GCF_000699105.1
Holostei_Amia_calva	Hughes, 2018, PNAS

bioRxiv preprint doi: https://doi.org/10.64898/2026.06.28.735080; this version posted June 29, 2026. The copyright holder for this preprint (which was not certified by peer review) is the author/funder, who has granted bioRxiv a license to display the preprint in perpetuity. It is made available under a CC-BY 4.0 International license.

Holostei_Atractosteus_tropicus	SRR17183800
Holostei_Atractosteus_spatula	SRR17183802
Holostei_Atractosteus_tistoechus	SRR17183801
Holostei_Lepisosteus_osseus	SRR17183799
Holostei_Lepisosteus_platostomus	SRR17183797
Holostei_Lepisosteus_oculatus	Hughes, 2018, PNAS
Holostei_Lepisosteus_platyrhincus	SRR17183798
Teleostei_Pantodon_buchholzi	Hughes, 2018, PNAS
Arapaima_gigas	GCA_900497675.1
Teleostei_Osteoglossum_bicirrhosum	Hughes, 2018, PNAS
Teleostei_Scleropages_formosus	Hughes, 2018, PNAS
Teleostei_Papyrocranus_afer	Hughes, 2018, PNAS
Teleostei_Mormyrus_tapirus	Hughes, 2018, PNAS
Teleostei_Gnathonemus_petersii	Hughes, 2018, PNAS
Paramormyrops_kingsleyae	GCF_002872115.1
Megalops_atlanticus	GCA_019176425.1
Teleostei_Megalops_cyprinoides	Hughes, 2018, PNAS
Albula_goreensis	GCA_022829145.1
Aldrovandia_affinis	GCA_029706075.1
Synaphobranchus_kaupii	GCA_029718625.1
Teleostei_Anguilla_japonica	Hughes, 2018, PNAS
Teleostei_Anguilla_anguilla	Hughes, 2018, PNAS
Teleostei_Anguilla_rostrata	Hughes, 2018, PNAS
Conger_conger	GCA_963514075.1
Teleostei_Conger_cinereus	Hughes, 2018, PNAS
Teleostei_Kaupichthys_hyporoides	Hughes, 2018, PNAS
Gymnothorax_javanicus	GCA_029692085.1
Teleostei_Gymnothorax_reevesii	Hughes, 2018, PNAS
Teleostei_Engraulis_encrasicolus	Hughes, 2018, PNAS
Teleostei_Coilia_nasus	Hughes, 2018, PNAS
Teleostei_Clupea_harengus	Hughes, 2018, PNAS
Teleostei_Alosa_alosa	Hughes, 2018, PNAS
Teleostei_Amblygaster_clupeoides	Hughes, 2018, PNAS
Teleostei_Chanos_chanos	Hughes, 2018, PNAS
Teleostei_Gyrinocheilus_aymonieri	Hughes, 2018, PNAS
Teleostei_Sinibotia_superciliaris	Hughes, 2018, PNAS
Teleostei_Homatula_potanini	Hughes, 2018, PNAS
Teleostei_Misgurnus_anguillicaudatus	Hughes, 2018, PNAS
Teleostei_Danio_rerio	Hughes, 2018, PNAS
Danionella_dracula	GCA_900490495.1

bioRxiv preprint doi: https://doi.org/10.64898/2026.06.28.735080; this version posted June 29, 2026. The copyright holder for this preprint (which was not certified by peer review) is the author/funder, who has granted bioRxiv a license to display the preprint in perpetuity. It is made available under a CC-BY 4.0 International license.

Teleostei_Cyprinus_carpio	Hughes, 2018, PNAS
Teleostei_Sinocyclocheilus_rhinocerous	Hughes, 2018, PNAS
Teleostei_Sinocyclocheilus_anshuiensis	Hughes, 2018, PNAS
Teleostei_Sinocyclocheilus_grahami	Hughes, 2018, PNAS
Teleostei_Tinca_tinca	Hughes, 2018, PNAS
Teleostei_Leuciscus_waleckii	Hughes, 2018, PNAS
Meda_fulgida	GCA_030578275.1
Tiaroga_cobitis	GCA_030578255.1
Teleostei_Pimephales_promelas	Hughes, 2018, PNAS
Teleostei_Distichodus_sexfasciatus	Hughes, 2018, PNAS
Teleostei_Apteronotus_albifrons	Hughes, 2018, PNAS
Teleostei_Electrophorus_electricus	Hughes, 2018, PNAS
Teleostei_Rhamphichthys_rostratus	Hughes, 2018, PNAS
Teleostei_Erythrinus_erythrinus	Hughes, 2018, PNAS
Teleostei_Pygocentrus_nattereri	Hughes, 2018, PNAS
Teleostei_Hepsetus_odoe	Hughes, 2018, PNAS
Teleostei_Gasteropelecus_sp	Hughes, 2018, PNAS
Teleostei_Hemigrammus_bleheri	Hughes, 2018, PNAS
Teleostei_Astyanax_mexicanus	Hughes, 2018, PNAS
Teleostei_Thayeria_boehlkei	Hughes, 2018, PNAS
Teleostei_Corydoras_julii	Hughes, 2018, PNAS
Teleostei_Pterygoplichthys_pardalis	Hughes, 2018, PNAS
Teleostei_Hemibagrus_guttatus	Hughes, 2018, PNAS
Teleostei_Glyptothorax_sinensis	Hughes, 2018, PNAS
Teleostei_Liobagrus_styani	Hughes, 2018, PNAS
Teleostei_Silurus_asotus	Hughes, 2018, PNAS
Teleostei_Plotosus_lineatus	Hughes, 2018, PNAS
Teleostei_Pangasianodon_hypophthalamus	Hughes, 2018, PNAS
Teleostei_Ictalurus_punctatus	Hughes, 2018, PNAS
Teleostei_Lepidogalaxias_salamandroides	Hughes, 2018, PNAS
Teleostei_Argentina_sp	Hughes, 2018, PNAS
Teleostei_Esox_lucius	Hughes, 2018, PNAS
Teleostei_Umbra_pygmae	Hughes, 2018, PNAS
Teleostei_Thymallus_thymallus	Hughes, 2018, PNAS
Teleostei_Coregonus_clupeaformis	Hughes, 2018, PNAS
Teleostei_Salmo_salar	Hughes, 2018, PNAS
Teleostei_Salvelinus_fontinalis	Hughes, 2018, PNAS
Teleostei_Oncorhynchus_mykiss	Hughes, 2018, PNAS
Teleostei_Borostomias_antarcticus	Hughes, 2018, PNAS
Teleostei_Osmerus_eperlanus	Hughes, 2018, PNAS

bioRxiv preprint doi: https://doi.org/10.64898/2026.06.28.735080; this version posted June 29, 2026. The copyright holder for this preprint (which was not certified by peer review) is the author/funder, who has granted bioRxiv a license to display the preprint in perpetuity. It is made available under a CC-BY 4.0 International license.

Teleostei_Plecoglossus_altivelis	Hughes, 2018, PNAS
Teleostei_Protosalanx_hyalocranius	Hughes, 2018, PNAS
Teleostei_Galaxias_maculatus	Hughes, 2018, PNAS
Teleostei_Galaxiella_nigrostriata	Hughes, 2018, PNAS
Teleostei_Synodus_intermedius	Hughes, 2018, PNAS
Teleostei_Parasudis_fraserbrunneri	Hughes, 2018, PNAS
Teleostei_Chlorophthalmus_agassizi	Hughes, 2018, PNAS
Teleostei_Guentherus_altivela	Hughes, 2018, PNAS
Teleostei_Benthosema_glaciale	Hughes, 2018, PNAS
Teleostei_Typhlichthys_subterraneus	Hughes, 2018, PNAS
Teleostei_Percopsis_transmontana	Hughes, 2018, PNAS
Teleostei_Percopsis_omiscomaycus	Hughes, 2018, PNAS
Teleostei_Polymixia_japonica	Hughes, 2018, PNAS
Teleostei_Regalecus_glesne	Hughes, 2018, PNAS
Teleostei_Lampris_guttatus	Hughes, 2018, PNAS
Lampris_megalopsis	GCA_022114975.2
Teleostei_Monocentris_japonica	Hughes, 2018, PNAS
Teleostei_Beryx_splendens	Hughes, 2018, PNAS
Teleostei_Acanthochaenus_luetkenii	Hughes, 2018, PNAS
Teleostei_Rondeletia_loricata	Hughes, 2018, PNAS
Teleostei_Myripristis_jacobus	Hughes, 2018, PNAS
Teleostei_Myripristis_berndti	Hughes, 2018, PNAS
Teleostei_Sargocentron_rubrum	Hughes, 2018, PNAS
Teleostei_Holocentrus_rufus	Hughes, 2018, PNAS
Teleostei_Neoniphon_sammara	Hughes, 2018, PNAS
Teleostei_Neoniphon_vexillarium	Hughes, 2018, PNAS
Teleostei_Carapus_acus	Hughes, 2018, PNAS
Teleostei_Brotula_barbata	Hughes, 2018, PNAS
Teleostei_Lamprogrammus_exutus	Hughes, 2018, PNAS
Teleostei_Porichthys_notatus	Hughes, 2018, PNAS
Teleostei_Chatrabus_melanurus	Hughes, 2018, PNAS
Teleostei_Batrachomoeus_trispinosus	Hughes, 2018, PNAS
Teleostei_Apogonichthyoides_cathetogramma	Hughes, 2018, PNAS
Teleostei_Phaeoptyx_conklini	Hughes, 2018, PNAS
Teleostei_Periophthalmodon_schlosseri	Hughes, 2018, PNAS
Teleostei_Periophthalmus_magnuspinnatus	Hughes, 2018, PNAS
Teleostei_Boleophthalmus_pectinirostris	Hughes, 2018, PNAS
Teleostei_Scartelaos_histophorus	Hughes, 2018, PNAS
Teleostei_Exyrias_puntang	Hughes, 2018, PNAS

bioRxiv preprint doi: https://doi.org/10.64898/2026.06.28.735080; this version posted June 29, 2026. The copyright holder for this preprint (which was not certified by peer review) is the author/funder, who has granted bioRxiv a license to display the preprint in perpetuity. It is made available under a CC-BY 4.0 International license.

Teleostei_Istigobius_decoratus	Hughes, 2018, PNAS
Teleostei_Glossogobius_aureus	Hughes, 2018, PNAS
Teleostei_Coryphopterus_lipernes	Hughes, 2018, PNAS
Teleostei_Lesueurigobius_cf	Hughes, 2018, PNAS
Teleostei_Parupeneus_indicus	Hughes, 2018, PNAS
Teleostei_Foetorepus_agassizii	Hughes, 2018, PNAS
Phyllopteryx_taeniolatus	GCA_019802545.1
Teleostei_Syngnathoides_biaculeatus	Hughes, 2018, PNAS
Teleostei_Syngnathus_scovelli	Hughes, 2018, PNAS
Teleostei_Hippocampus_erectus	Hughes, 2018, PNAS
Hippocampus_zosterae	GCF_025434085.1
Teleostei_Photopectoralis_bindus	Hughes, 2018, PNAS
Teleostei_Aulostomus_maculatus	Hughes, 2018, PNAS
Teleostei_Aulostomus_maculatus2	Hughes, 2018, PNAS
Teleostei_Scomber_scombrus	Hughes, 2018, PNAS
Teleostei_Pampus_argenteus	Hughes, 2018, PNAS
Teleostei_Nomeus_gronovii	Hughes, 2018, PNAS
Teleostei_Scomberomorus_regalis	Hughes, 2018, PNAS
Teleostei_Thunnus_albacares	Hughes, 2018, PNAS
Teleostei_Thunnus_orientalis	Hughes, 2018, PNAS
Teleostei_Pronotogrammus_martinicensis	Hughes, 2018, PNAS
Teleostei_Lepidonotothen_nudifrons	Hughes, 2018, PNAS
Teleostei_Notothenia_coriiceps	Hughes, 2018, PNAS
Teleostei_Chaenocephalus_aceratus	Hughes, 2018, PNAS
Teleostei_Gymnodraco_acuticeps	Hughes, 2018, PNAS
Teleostei_Perca_fluviatilis	Hughes, 2018, PNAS
Teleostei_Chrionema_squamentum	Hughes, 2018, PNAS
Teleostei_Peristedion_brevirostris	Hughes, 2018, PNAS
Teleostei_Anoplopoma_fimbria	Hughes, 2018, PNAS
Teleostei_Gasterosteus_aculeatus	Hughes, 2018, PNAS
Teleostei_Cyclopterus_lumpus	Hughes, 2018, PNAS
Teleostei_Myoxocephalus_scorpius	Hughes, 2018, PNAS
Teleostei_Cottus_rhenanus	Hughes, 2018, PNAS
Teleostei_Scorpaenopsis_cirrosa	Hughes, 2018, PNAS
Teleostei_Synanceia_verrucosa	Hughes, 2018, PNAS
Teleostei_Dendrochirus_zebra	Hughes, 2018, PNAS
Teleostei_Pontinus_castor	Hughes, 2018, PNAS
Teleostei_Sebastes_norvegicus	Hughes, 2018, PNAS
Teleostei_Sebastes_nigrocinctus	Hughes, 2018, PNAS
Teleostei_Sebastes_rubrivinctus	Hughes, 2018, PNAS

bioRxiv preprint doi: https://doi.org/10.64898/2026.06.28.735080; this version posted June 29, 2026. The copyright holder for this preprint (which was not certified by peer review) is the author/funder, who has granted bioRxiv a license to display the preprint in perpetuity. It is made available under a CC-BY 4.0 International license.

Teleostei_Parapercis_xanthozona	Hughes, 2018, PNAS
Teleostei_Thalassoma_bifasciatum	Hughes, 2018, PNAS
Teleostei_Labrus_bergylta	Hughes, 2018, PNAS
Teleostei_Symphodus_melops	Hughes, 2018, PNAS
Teleostei_Scarus_ghobban	Hughes, 2018, PNAS
Teleostei_Scarus_iseri	Hughes, 2018, PNAS
Teleostei_Eucinostomus_jonesi	Hughes, 2018, PNAS
Teleostei_Gerres_filamentosus	Hughes, 2018, PNAS
Teleostei_Lateolabrax_maculatus	Hughes, 2018, PNAS
Teleostei_Epigonus_sp	Hughes, 2018, PNAS
Teleostei_Terapon_jarbua	Hughes, 2018, PNAS
Teleostei_Oplegnathus_punctatus	Hughes, 2018, PNAS
Teleostei_Amblycirrhitus_pinos	Hughes, 2018, PNAS
Teleostei_Micropterus_floridanus	Hughes, 2018, PNAS
Teleostei_Siniperca_scherzeri	Hughes, 2018, PNAS
Teleostei_Coreoperca_whiteheadi	Hughes, 2018, PNAS
Teleostei_Haemulon_chrysargyreum	Hughes, 2018, PNAS
Teleostei_Haemulon_flavolineatum	Hughes, 2018, PNAS
Teleostei_Siganus_guttatus	Hughes, 2018, PNAS
Teleostei_Dicentrarchus_labrax	Hughes, 2018, PNAS
Teleostei_Morone_saxatilis	Hughes, 2018, PNAS
Teleostei_Drepane_punctata	Hughes, 2018, PNAS
Teleostei_Chaetodipterus_faber	Hughes, 2018, PNAS
Teleostei_Datnioides_microlepis	Hughes, 2018, PNAS
Teleostei_Equetus_punctatus	Hughes, 2018, PNAS
Teleostei_Miichthys_miiuy	Hughes, 2018, PNAS
Teleostei_Larimichthys_crocea	Hughes, 2018, PNAS
Teleostei_Lutjanus_sebae	Hughes, 2018, PNAS
Teleostei_Lutjanus_fulviflamma	Hughes, 2018, PNAS
Teleostei_Chaetodon_auriga	Hughes, 2018, PNAS
Teleostei_Evynnis_cardinalis	Hughes, 2018, PNAS
Teleostei_Spondyliosoma_cantharus	Hughes, 2018, PNAS
Teleostei_Acanthopagrus_schlegelii	Hughes, 2018, PNAS
Teleostei_Acanthopagrus_latus	Hughes, 2018, PNAS
Teleostei_Pomacanthus_paru	Hughes, 2018, PNAS
Teleostei_Acanthurus_tractus	Hughes, 2018, PNAS
Teleostei_Priacanthus_tayenus	Hughes, 2018, PNAS
Teleostei_Antigonia_capros	Hughes, 2018, PNAS
Lophius_piscatorius	GCA_009660295.1
Teleostei_Antennarius_striatus	Hughes, 2018, PNAS

bioRxiv preprint doi: https://doi.org/10.64898/2026.06.28.735080; this version posted June 29, 2026. The copyright holder for this preprint (which was not certified by peer review) is the author/funder, who has granted bioRxiv a license to display the preprint in perpetuity. It is made available under a CC-BY 4.0 International license.

Teleostei_Chaunax_pictus	Hughes, 2018, PNAS
Teleostei_Pseudobalistes_fuscus	Hughes, 2018, PNAS
Teleostei_Mola_mola	Hughes, 2018, PNAS
Teleostei_Lactoria_cornuta	Hughes, 2018, PNAS
Teleostei_Ostracion_rhinorhynchos	Hughes, 2018, PNAS
Teleostei_Diodon_holocanthus	Hughes, 2018, PNAS
Teleostei_Tetraodon_nigroviridis	Hughes, 2018, PNAS
Teleostei_Takifugu_flavidus	Hughes, 2018, PNAS
Teleostei_Takifugu_rubripes	Hughes, 2018, PNAS
Teleostei_Monopterus_albus	Hughes, 2018, PNAS
Teleostei_Mastacembelus_armatus	Hughes, 2018, PNAS
Teleostei_Macrognathus_aculeatus	Hughes, 2018, PNAS
Teleostei_Channa_micropeltes	Hughes, 2018, PNAS
Teleostei_Channa_argus	Hughes, 2018, PNAS
Teleostei_Channa_gachua	Hughes, 2018, PNAS
Teleostei_Helostoma_temminckii	Hughes, 2018, PNAS
Teleostei_Anabas_testudineus	Hughes, 2018, PNAS
Teleostei_Osphronemus_goramy	Hughes, 2018, PNAS
Teleostei_Polynemus_dubius	Hughes, 2018, PNAS
Teleostei_Cynoglossus_semilaevis	Hughes, 2018, PNAS
Teleostei_Solea_ovata	Hughes, 2018, PNAS
Teleostei_Scophthalmus_maximus	Hughes, 2018, PNAS
Teleostei_Paralichthys_olivaceus	Hughes, 2018, PNAS
Teleostei_Pseudopleuronectes_yokohamae	Hughes, 2018, PNAS
Teleostei_Hippoglossus_hippoglossus	Hughes, 2018, PNAS
Teleostei_Toxotes_jaculatrix	Hughes, 2018, PNAS
Teleostei_Lates_calcarifer	Hughes, 2018, PNAS
Teleostei_Centropomus_undecimalis	Hughes, 2018, PNAS
Teleostei_Coryphaena_hippurus	Hughes, 2018, PNAS
Teleostei_Seriola_lalandi	Hughes, 2018, PNAS
Teleostei_Trachinotus_ovatus	Hughes, 2018, PNAS
Teleostei_Selene_dorsalis	Hughes, 2018, PNAS
Teleostei_Caranx_ignobilis	Hughes, 2018, PNAS
Teleostei_Acyrtus_rubiginosus	Hughes, 2018, PNAS
Teleostei_Tomicodon_sp	Hughes, 2018, PNAS
Teleostei_Enneanectes_sp	Hughes, 2018, PNAS
Teleostei_Parablennius_parvicornis	Hughes, 2018, PNAS
Teleostei_Acanthemblemaria_spinosa	Hughes, 2018, PNAS
Teleostei_Liza_haematocheila	Hughes, 2018, PNAS
Teleostei_Mugil_cephalus	Hughes, 2018, PNAS

bioRxiv preprint doi: https://doi.org/10.64898/2026.06.28.735080; this version posted June 29, 2026. The copyright holder for this preprint (which was not certified by peer review) is the author/funder, who has granted bioRxiv a license to display the preprint in perpetuity. It is made available under a CC-BY 4.0 International license.

Teleostei_Pseudochromis_fuscus	Hughes, 2018, PNAS
Teleostei_Lipogramma_evides	Hughes, 2018, PNAS
Teleostei_Opistognathus_aurifrons	Hughes, 2018, PNAS
Teleostei_Stegastes_partitus	Hughes, 2018, PNAS
Teleostei_Amphiprion_melanopus	Hughes, 2018, PNAS
Teleostei_Chromis_chromis	Hughes, 2018, PNAS
Teleostei_Dascyllus_trimaculatus	Hughes, 2018, PNAS
Teleostei_Parambassis_pulcinella	Hughes, 2018, PNAS
Teleostei_Amphilophus_citrinellus	Hughes, 2018, PNAS
Teleostei_Parachromis_managuensis	Hughes, 2018, PNAS
Teleostei_Oreochromis_niloticus	Hughes, 2018, PNAS
Teleostei_Neolamprologus_brichardi	Hughes, 2018, PNAS
Teleostei_Haplochromis_burtoni	Hughes, 2018, PNAS
Teleostei_Pundamilia_nyererei	Hughes, 2018, PNAS
Teleostei_Mchenga_conophoros	Hughes, 2018, PNAS
Teleostei_Melanochromis_auratus	Hughes, 2018, PNAS
Teleostei_Maylandia_zebra	Hughes, 2018, PNAS
Teleostei_Labeotropheus_fuelleborni	Hughes, 2018, PNAS
Teleostei_Rhamphochromis_esox	Hughes, 2018, PNAS
Teleostei_Oryzias_latipes	Hughes, 2018, PNAS
Teleostei_Oryzias_mekongensis	Hughes, 2018, PNAS
Teleostei_Hyporhamphus_intermedius	Hughes, 2018, PNAS
Teleostei_Pseudomugil_paskai	Hughes, 2018, PNAS
Teleostei_Glossolepis_incisus	Hughes, 2018, PNAS
Teleostei_Melanotaenia_praecox	Hughes, 2018, PNAS
Teleostei_Menidia_menidia	Hughes, 2018, PNAS
Teleostei_Odontesthes_bonariensis	Hughes, 2018, PNAS
Teleostei_Basilichthys_microlepidotus	Hughes, 2018, PNAS
Teleostei_Austrofundulus_limnaeus	Hughes, 2018, PNAS
Teleostei_Kryptolebias_marmoratus	Hughes, 2018, PNAS
Teleostei_Nothobranchius_furzeri	Hughes, 2018, PNAS
Teleostei_Aplocheilus_lineatus	Hughes, 2018, PNAS
Teleostei_Pachypanchax_sakaramyi	Hughes, 2018, PNAS
Teleostei_Fundulus_heteroclitus	Hughes, 2018, PNAS
Teleostei_Ameca_splendens	Hughes, 2018, PNAS
Teleostei_Cyprinodon_variegatus	Hughes, 2018, PNAS
Teleostei_nevadensis_pectoralis	Hughes, 2018, PNAS
Teleostei_Poecilia_formosa	Hughes, 2018, PNAS
Teleostei_Poecilia_reticulata	Hughes, 2018, PNAS
Teleostei_Poeciliopsis_prolifica	Hughes, 2018, PNAS

bioRxiv preprint doi: https://doi.org/10.64898/2026.06.28.735080; this version posted June 29, 2026. The copyright holder for this preprint (which was not certified by peer review) is the author/funder, who has granted bioRxiv a license to display the preprint in perpetuity. It is made available under a CC-BY 4.0 International license.

Teleostei_affinis_whole	Hughes, 2018, PNAS
Teleostei_Xiphophorus_hellerii	Hughes, 2018, PNAS
Teleostei_Xiphophorus_couchianus	Hughes, 2018, PNAS
Teleostei_Xiphophorus_maculatus	Hughes, 2018, PNAS
Teleostei_Stylephorus_chordatus	Hughes, 2018, PNAS
Teleostei_Bregmaceros_cantori	Hughes, 2018, PNAS
Teleostei_Macrourus_berglax	Hughes, 2018, PNAS
Teleostei_Malacocephalus_occidentalis	Hughes, 2018, PNAS
Teleostei_Muraenolepis_marmoratus	Hughes, 2018, PNAS
Teleostei_Merluccius_polli	Hughes, 2018, PNAS
Teleostei_Merluccius_capensis	Hughes, 2018, PNAS
Teleostei_Merluccius_merluccius	Hughes, 2018, PNAS
Teleostei_Bathygadus_melanobranchus	Hughes, 2018, PNAS
Teleostei_Melanorus_zugmayeri	Hughes, 2018, PNAS
Teleostei_Laemonema_laureysi	Hughes, 2018, PNAS
Teleostei_Mora_moro	Hughes, 2018, PNAS
Teleostei_Trachyrincus_murrayi	Hughes, 2018, PNAS
Teleostei_Trachyrincus_scabrus	Hughes, 2018, PNAS
Teleostei_Phycis_blennoides	Hughes, 2018, PNAS
Teleostei_Phycis_phycis	Hughes, 2018, PNAS
Teleostei_Lota_lota	Hughes, 2018, PNAS
Teleostei_Brosme_brosme	Hughes, 2018, PNAS
Teleostei_Molva_molva	Hughes, 2018, PNAS
Teleostei_Gadiculus_argenteus	Hughes, 2018, PNAS
Teleostei_Trisopterus_minutus	Hughes, 2018, PNAS
Teleostei_Pollachius_virens	Hughes, 2018, PNAS
Teleostei_Melanogrammus_aeglefinus	Hughes, 2018, PNAS
Teleostei_Merlangius_merlangus	Hughes, 2018, PNAS
Teleostei_Gadus_morhua	Hughes, 2018, PNAS
Teleostei_Theragra_chalcogramma	Hughes, 2018, PNAS
Teleostei_Arctogadus_glacialis	Hughes, 2018, PNAS
Teleostei_Boreogadus_saida	Hughes, 2018, PNAS
Teleostei_Zeus_faber	Hughes, 2018, PNAS
Teleostei_Cyttopsis_rosea2	Hughes, 2018, PNAS
Teleostei_Cyttopsis_rosea	Hughes, 2018, PNAS

Table S2. Comparison of divergence time estimates for key clades estimated from BEAST2 node dating analyses of separate and combined exon sets. Mean and 95% HPDs (Min, Max)

bioRxiv preprint doi: https://doi.org/10.64898/2026.06.28.735080; this version posted June 29, 2026. The copyright holder for this preprint (which was not certified by peer review) is the author/funder, who has granted bioRxiv a license to display the preprint in perpetuity. It is made available under a CC-BY 4.0 International license.

are shown. Note: *Cetoartiodactyla* and *Eulipotypha* may not fully sample MRCA of these clades. ALL, all three sets; E0066, set 1; E0160, set 2; E0026, set 3.

**Clade**	**Tree**	**Median**	**Min**	**Max**	**Relevant Event**
Acropomatiformes	Cal Test	72.1526	44.4932	92.2623	Cretaceous-Paleogene
Afrotheria	Cal Test	67.7928	58.1806	71.9714	Cretaceous-Paleogene
Americhelydia+Testudinoidea	Cal Test	51.1232	37.1834	57.3928	Cretaceous-Paleogene
Anguilliformes	Cal Test	78.7256	47.3165	95.8591	Cretaceous-Paleogene
Aplocheiloidea	Cal Test	58.3115	35.7295	76.7548	Cretaceous-Paleogene
Australidelphia	Cal Test	54.1525	34.8054	58.0829	Cretaceous-Paleogene
Belonioidei	Cal Test	62.3633	46.1636	89.301	Cretaceous-Paleogene
Carangioidei	Cal Test	62.1228	54.67	65.9015	Cretaceous-Paleogene
Cetoartiodactyla	Cal Test	68.5951	50.3068	70.2419	Cretaceous-Paleogene
Cirrhitoidei+Centrarchoidei	Cal Test	57.6711	30.2872	79.8793	Cretaceous-Paleogene
Cottoidea	Cal Test	64.4476	48.8592	75.4429	Cretaceous-Paleogene
Cypriniformes	Cal Test	64.9631	54.2595	62.2284	Cretaceous-Paleogene
Chiroptera	Cal Test	59.3235	47.1193	57.4285	Cretaceous-Paleogene
Euarchonta	Cal Test	70.3377	55.9267	78.5913	Cretaceous-Paleogene
Eulipotyphla	Cal Test	63.0783	50.5857	71.1561	Cretaceous-Paleogene
Ferae	Cal Test	69.432	57.1852	67.8781	Cretaceous-Paleogene
Gadiformes	Cal Test	72.3978	53.0515	83.8532	Cretaceous-Paleogene
Galloanserae	Cal Test	72.9409	59.8735	71.8568	Cretaceous-Paleogene
Glires	Cal Test	59.5624	39.2202	65.0945	Cretaceous-Paleogene
Gobioidei	Cal Test	61.6649	40.4754	89.3349	Cretaceous-Paleogene
Gymnotiformes	Cal Test	56.4226	26.8573	83.9812	Cretaceous-Paleogene
Labriformes	Cal Test	55.6206	49.5349	59.7959	Cretaceous-Paleogene
Lampriformes	Cal Test	51.3942	28.7097	84.0055	Cretaceous-Paleogene
Lophioidei	Cal Test	52.97	48.5352	58.1495	Cretaceous-Paleogene
Neoaves	Cal Test	79.1548	67.9253	77.0644	Cretaceous-Paleogene
Paleognathae	Cal Test	59.5222	47.4657	57.2278	Cretaceous-Paleogene
Perissodactyla	Cal Test	50.239	25.6231	62.6911	Cretaceous-Paleogene
Pleuronectoidei	Cal Test	46.2765	26.3425	62.463	Cretaceous-Paleogene
Pythonomorpha	Cal Test	58.8215	47.0647	58.322	Cretaceous-Paleogene
Scombriformes	Cal Test	60.6781	53.8569	65.5177	Cretaceous-Paleogene
Scorpaenoidea	Cal Test	65.2586	49.4173	77.8218	Cretaceous-Paleogene
Siluroidei	Cal Test	72.8324	52.8862	79.1359	Cretaceous-Paleogene
Xenarthra	Cal Test	59.6641	47.3844	58.4455	Cretaceous-Paleogene
Aves	Cal Test	109.2615	83.2013	129.135	Cretaceous-Terrestrial-Revolution
Boreoeutheria	Cal Test	97.7544	74.9685	111.5105	Cretaceous-Terrestrial-Revolution

bioRxiv preprint doi: https://doi.org/10.64898/2026.06.28.735080; this version posted June 29, 2026. The copyright holder for this preprint (which was not certified by peer review) is the author/funder, who has granted bioRxiv a license to display the preprint in perpetuity. It is made available under a CC-BY 4.0 International license.

Euarchontoglires	Cal Test	78.3303	60.8605	90.2089	Cretaceous-Terrestrial-Revolution
Laurasiatheria	Cal Test	85.3485	67.224	95.4486	Cretaceous-Terrestrial-Revolution
Marsupialia	Cal Test	67.7928	55.5884	66.5969	Cretaceous-Terrestrial-Revolution
Placentalia	Cal Test	113.1743	88.0988	129.5275	Cretaceous-Terrestrial-Revolution
Toxicofera	Cal Test	142.1091	120.6278	148.4431	Cretaceous-Terrestrial-Revolution
Actinopteri	Cal Test	363.5494	324.1502	402.4953	End-Devonian
Chondrichthyes	Cal Test	389.9797	350.2515	411.3358	End-Devonian
Tetrapoda	Cal Test	387.7878	336.3313	415.3943	End-Devonian
Theria	Cal Test	142.7561	115.5171	161.1036	End-Jurassic-Turnover
Sarcopterygii	Cal Test	451.7027	402.6542	480.0094	End-Ordovician
Archosauria	Cal Test	279.8319	237.9508	281.3412	End-Permian
Clupeocephala	Cal Test	265.7206	234.8194	274.3292	End-Permian
Euteleostei	Cal Test	250.609	225.3633	257.383	End-Permian
Holostei	Cal Test	278.5742	247.8359	296.0832	End-Permian
Lepidosauria	Cal Test	269.6823	243.6483	283.0914	End-Permian
Lissamphibia	Cal Test	270.107	232.6776	291.3839	End-Permian
Oseanacephala	Cal Test	228.6386	166.8344	269.575	End-Permian
Neoteleostei	Cal Test	200.9838	173.7832	218.9805	End-Trassic
Squamata	Cal Test	193.671	165.8803	210.4408	End-Triassic
Actinopterygii	Cal Test	395.466	317.2612	433.8007	Mid-Devonian
Acanthomorpha	Cal Test	175.976	154.5899	187.5772	Mid-Jurassic
Mammalia	Cal Test	178.5647	157.5793	186.8972	Mid-Jurassic
Testudines	Cal Test	172.0272	151.3048	179.4185	Mid-Jurassic
Archelosauria	Cal Test	265.198	255.5247	294.2408	End-Permian
Acropomatiformes	E0066	68.6	41.34	89.04	Cretaceous-Paleogene
Acropomatiformes	ALL	67.56	36.45	90.39	Cretaceous-Paleogene
Acropomatiformes	E0160	67.44	25.23	89.9	Cretaceous-Paleogene
Acropomatiformes	E0026	66.62	46.2	83.64	Cretaceous-Paleogene
Afrotheria	E0026	65.5	59.32	72.47	Cretaceous-Paleogene
Afrotheria	E0160	65.45	59.41	71.78	Cretaceous-Paleogene

bioRxiv preprint doi: https://doi.org/10.64898/2026.06.28.735080; this version posted June 29, 2026. The copyright holder for this preprint (which was not certified by peer review) is the author/funder, who has granted bioRxiv a license to display the preprint in perpetuity. It is made available under a CC-BY 4.0 International license.

Afrotheria	ALL	65.37	59.19	72.19	Cretaceous-Paleogene
Afrotheria	E0066	65.19	58.5	71.81	Cretaceous-Paleogene
Americhelydia+Testudinoidea	E0026	47.94	37.57	70.61	Cretaceous-Paleogene
Americhelydia+Testudinoidea	E0160	47.84	36.35	63.62	Cretaceous-Paleogene
Americhelydia+Testudinoidea	ALL	47.17	37.64	67.28	Cretaceous-Paleogene
Americhelydia+Testudinoidea	E0066	46.05	37.92	60	Cretaceous-Paleogene
Anguilliformes	E0026	70.9	45.94	93.4	Cretaceous-Paleogene
Anguilliformes	ALL	69.94	47.06	92.77	Cretaceous-Paleogene
Anguilliformes	E0066	65.56	46.67	88.76	Cretaceous-Paleogene
Anguilliformes	E0160	53.42	51.9	95.58	Cretaceous-Paleogene
Aplocheiloidea	E0026	62.77	49.5	85.95	Cretaceous-Paleogene
Aplocheiloidea	E0160	57.39	42.99	79.13	Cretaceous-Paleogene
Aplocheiloidea	ALL	55.37	39.96	84.27	Cretaceous-Paleogene
Aplocheiloidea	E0066	46.75	36.8	62.86	Cretaceous-Paleogene
Australidelphia	E0026	71.14	52.9	94.35	Cretaceous-Paleogene
Australidelphia	ALL	64.32	41.27	91.01	Cretaceous-Paleogene
Australidelphia	E0066	61.77	41.47	87.51	Cretaceous-Paleogene
Australidelphia	E0160	59.37	37.84	91.08	Cretaceous-Paleogene
Belonioidei	E0160	62.04	38.48	86.04	Cretaceous-Paleogene
Belonioidei	E0026	60.99	50.01	87.43	Cretaceous-Paleogene
Belonioidei	ALL	59.35	28.95	79.91	Cretaceous-Paleogene
Belonioidei	E0066	53.68	28.09	70.5	Cretaceous-Paleogene
Carangioidei	E0026	60.82	55.35	66.52	Cretaceous-Paleogene
Carangioidei	E0066	60.73	55.03	66.97	Cretaceous-Paleogene
Carangioidei	ALL	60.67	55.15	66.8	Cretaceous-Paleogene
Carangioidei	E0160	60.46	55.08	66.77	Cretaceous-Paleogene
Cetoartiodactyla	E0026	62.53	53.97	73.08	Cretaceous-Paleogene
Cetoartiodactyla	ALL	60.35	48.32	72.24	Cretaceous-Paleogene
Cetoartiodactyla	E0066	60.19	50.1	70.54	Cretaceous-Paleogene
Cetoartiodactyla	E0160	58.14	47.1	71.15	Cretaceous-Paleogene
Cirrhitoidei+Centrarchoidei	E0026	68.92	41.87	91.25	Cretaceous-Paleogene
Cirrhitoidei+Centrarchoidei	ALL	62.25	24.91	91.75	Cretaceous-Paleogene
Cirrhitoidei+Centrarchoidei	E0066	60.6	32.31	86.89	Cretaceous-Paleogene
Cirrhitoidei+Centrarchoidei	E0160	57.09	20.58	95.59	Cretaceous-Paleogene
Cottoidea	E0160	66.17	48.28	84.61	Cretaceous-Paleogene
Cottoidea	E0026	64.53	54.36	75.99	Cretaceous-Paleogene
Cottoidea	ALL	63.24	46.12	79.59	Cretaceous-Paleogene
Cottoidea	E0066	57.12	44.02	71.26	Cretaceous-Paleogene
Cypriniformes	E0160	58.09	52.65	63.46	Cretaceous-Paleogene
Cypriniformes	E0026	57.98	52.12	62.62	Cretaceous-Paleogene

bioRxiv preprint doi: https://doi.org/10.64898/2026.06.28.735080; this version posted June 29, 2026. The copyright holder for this preprint (which was not certified by peer review) is the author/funder, who has granted bioRxiv a license to display the preprint in perpetuity. It is made available under a CC-BY 4.0 International license.

Cypriniformes	ALL	57.64	52.73	62.67	Cretaceous-Paleogene
Cypriniformes	E0066	57.13	53	61.66	Cretaceous-Paleogene
Chiroptera	E0026	57.5	52.46	62.38	Cretaceous-Paleogene
Chiroptera	E0160	57.48	52.03	63.05	Cretaceous-Paleogene
Chiroptera	ALL	57.38	52.29	62.62	Cretaceous-Paleogene
Chiroptera	E0066	57.15	52.48	62.46	Cretaceous-Paleogene
Euarchonta	E0066	73.54	67.17	80.24	Cretaceous-Paleogene
Euarchonta	E0160	72.98	65.78	79.96	Cretaceous-Paleogene
Euarchonta	ALL	72.93	66.21	79.57	Cretaceous-Paleogene
Euarchonta	E0026	72.33	66.34	78.34	Cretaceous-Paleogene
Eulipotyphla	E0066	70.7	49.92	88.08	Cretaceous-Paleogene
Eulipotyphla	E0026	58.87	43.36	70.84	Cretaceous-Paleogene
Eulipotyphla	ALL	57.9	20.63	80.71	Cretaceous-Paleogene
Eulipotyphla	E0160	41.59	19.57	60.03	Cretaceous-Paleogene
Ferae	E0160	70.88	64.28	77.37	Cretaceous-Paleogene
Ferae	ALL	69.59	63.22	76.23	Cretaceous-Paleogene
Ferae	E0066	69.23	63.71	76.09	Cretaceous-Paleogene
Ferae	E0026	68.79	62.52	74.88	Cretaceous-Paleogene
Gadiformes	E0026	73.26	61.3	93.77	Cretaceous-Paleogene
Gadiformes	ALL	72.28	57.11	87.16	Cretaceous-Paleogene
Gadiformes	E0160	71.93	57.61	84.88	Cretaceous-Paleogene
Gadiformes	E0066	71.25	58.53	84.03	Cretaceous-Paleogene
Galloanserae	E0026	66.63	61.08	72.48	Cretaceous-Paleogene
Galloanserae	E0066	66.56	59.32	72.64	Cretaceous-Paleogene
Galloanserae	ALL	66.55	60.62	72.75	Cretaceous-Paleogene
Galloanserae	E0160	66.47	61.2	72.78	Cretaceous-Paleogene
Glires	E0160	71.83	63.48	82.68	Cretaceous-Paleogene
Glires	E0026	70.84	62.81	78.98	Cretaceous-Paleogene
Glires	ALL	70.73	62.73	81.05	Cretaceous-Paleogene
Glires	E0066	69.7	61.38	80.29	Cretaceous-Paleogene
Gobioidei	E0026	62.75	44.58	96.38	Cretaceous-Paleogene
Gobioidei	ALL	61.5	40.27	88.77	Cretaceous-Paleogene
Gobioidei	E0160	61.33	41.87	84.76	Cretaceous-Paleogene
Gobioidei	E0066	59.91	39.95	79.9	Cretaceous-Paleogene
Gymnotiformes	E0160	66.25	34.9	100.08	Cretaceous-Paleogene
Gymnotiformes	ALL	54.16	14.84	87.78	Cretaceous-Paleogene
Gymnotiformes	E0026	49.85	25.85	75.25	Cretaceous-Paleogene
Gymnotiformes	E0066	43.38	13.5	81.13	Cretaceous-Paleogene
Labriformes	E0160	54.68	49.34	59.49	Cretaceous-Paleogene
Labriformes	E0066	54.57	49.58	60.85	Cretaceous-Paleogene

bioRxiv preprint doi: https://doi.org/10.64898/2026.06.28.735080; this version posted June 29, 2026. The copyright holder for this preprint (which was not certified by peer review) is the author/funder, who has granted bioRxiv a license to display the preprint in perpetuity. It is made available under a CC-BY 4.0 International license.

Labriformes	ALL	54.53	49.58	60.2	Cretaceous-Paleogene
Labriformes	E0026	54.34	49.75	60.14	Cretaceous-Paleogene
Lampriformes	E0066	52.03	13.69	93.36	Cretaceous-Paleogene
Lampriformes	E0160	50.55	17.98	86.75	Cretaceous-Paleogene
Lampriformes	ALL	42.72	14.46	86.65	Cretaceous-Paleogene
Lampriformes	E0026	37.01	19.35	79.68	Cretaceous-Paleogene
Lophioidei	E0026	53.42	48.28	58.14	Cretaceous-Paleogene
Lophioidei	ALL	53.23	48.41	57.76	Cretaceous-Paleogene
Lophioidei	E0066	53.23	48.85	57.49	Cretaceous-Paleogene
Lophioidei	E0160	53.05	48.1	57.68	Cretaceous-Paleogene
Neoaves	E0160	72.13	67.45	77.63	Cretaceous-Paleogene
Neoaves	E0026	71.61	56.43	70.28	Cretaceous-Paleogene
Neoaves	ALL	71.39	66.45	76.16	Cretaceous-Paleogene
Neoaves	E0066	70.39	66.06	75.23	Cretaceous-Paleogene
Paleognathae	E0160	52.89	47.53	58.08	Cretaceous-Paleogene
Paleognathae	ALL	52.83	47.81	58.22	Cretaceous-Paleogene
Paleognathae	E0066	52.81	47.98	58.13	Cretaceous-Paleogene
Paleognathae	E0026	52.77	48.02	58.45	Cretaceous-Paleogene
Perissodactyla	E0066	42.11	19.25	60.4	Cretaceous-Paleogene
Perissodactyla	ALL	35.86	15.21	60.37	Cretaceous-Paleogene
Perissodactyla	E0160	34.05	14.95	61.9	Cretaceous-Paleogene
Perissodactyla	E0026	33.19	14.35	57.86	Cretaceous-Paleogene
Pleuronectoidei	E0026	66.95	51.68	76.48	Cretaceous-Paleogene
Pleuronectoidei	E0066	66.67	39.64	85.82	Cretaceous-Paleogene
Pleuronectoidei	E0160	65.47	48.85	100.75	Cretaceous-Paleogene
Pleuronectoidei	ALL	59.21	36.63	78.91	Cretaceous-Paleogene
Pythonomorpha	E0160	51.97	47.18	56.85	Cretaceous-Paleogene
Pythonomorpha	E0026	51.89	47.17	55.78	Cretaceous-Paleogene
Pythonomorpha	ALL	51.71	46.8	56.2	Cretaceous-Paleogene
Pythonomorpha	E0066	51.13	46.68	56.12	Cretaceous-Paleogene
Scombriformes	E0026	59.82	53.68	65.12	Cretaceous-Paleogene
Scombriformes	E0066	59.79	54.29	66.34	Cretaceous-Paleogene
Scombriformes	ALL	59.7	54.02	65.69	Cretaceous-Paleogene
Scombriformes	E0160	59.47	53.99	65.4	Cretaceous-Paleogene
Scorpaenoidea	E0160	80.47	36.23	98.06	Cretaceous-Paleogene
Scorpaenoidea	ALL	63.79	37.28	93.97	Cretaceous-Paleogene
Scorpaenoidea	E0026	62.44	49.15	77.84	Cretaceous-Paleogene
Scorpaenoidea	E0066	54.96	38.16	74.94	Cretaceous-Paleogene
Siluroidei	E0160	70.34	54.55	85.22	Cretaceous-Paleogene
Siluroidei	E0026	67.84	59.15	81.34	Cretaceous-Paleogene

bioRxiv preprint doi: https://doi.org/10.64898/2026.06.28.735080; this version posted June 29, 2026. The copyright holder for this preprint (which was not certified by peer review) is the author/funder, who has granted bioRxiv a license to display the preprint in perpetuity. It is made available under a CC-BY 4.0 International license.

Siluroidei	ALL	67.77	52.71	83.63	Cretaceous-Paleogene
Siluroidei	E0066	64.53	48.38	76.75	Cretaceous-Paleogene
Xenarthra	E0066	52.66	47.73	57.32	Cretaceous-Paleogene
Xenarthra	E0160	52.42	47.7	57.81	Cretaceous-Paleogene
Xenarthra	ALL	52.4	47.48	57.43	Cretaceous-Paleogene
Xenarthra	E0026	52.12	47.39	57.54	Cretaceous-Paleogene
Aves	E0026	100.21	85.81	125.8	Cretaceous-Terrestrial-Revolution
Aves	E0160	98.97	83.52	144.81	Cretaceous-Terrestrial-Revolution
Aves	ALL	97.42	83.15	132.22	Cretaceous-Terrestrial-Revolution
Aves	E0066	93.63	82.77	122.91	Cretaceous-Terrestrial-Revolution
Boreoeutheria	E0066	97.57	85.25	114.01	Cretaceous-Terrestrial-Revolution
Boreoeutheria	E0160	93.73	83.5	104.9	Cretaceous-Terrestrial-Revolution
Boreoeutheria	ALL	93.09	78.22	109.45	Cretaceous-Terrestrial-Revolution
Boreoeutheria	E0026	88.57	76.72	101.66	Cretaceous-Terrestrial-Revolution
Euarchontoglires	E0160	82.91	73.36	92.73	Cretaceous-Terrestrial-Revolution
Euarchontoglires	E0066	82.82	74.62	93.76	Cretaceous-Terrestrial-Revolution
Euarchontoglires	ALL	81.54	72.44	93.03	Cretaceous-Terrestrial-Revolution
Euarchontoglires	E0026	78.52	71.15	91.2	Cretaceous-Terrestrial-Revolution
Laurasiatheria	E0066	87.86	76.68	98.39	Cretaceous-Terrestrial-Revolution
Laurasiatheria	ALL	83.53	72.11	95.96	Cretaceous-Terrestrial-Revolution
Laurasiatheria	E0160	83.27	75.43	92.26	Cretaceous-Terrestrial-Revolution
Laurasiatheria	E0026	79.54	70.06	93.73	Cretaceous-Terrestrial-Revolution
Marsupialia	E0160	91.48	82.99	100.88	Cretaceous-Terrestrial-Revolution
Marsupialia	ALL	91.45	82.75	101.97	Cretaceous-Terrestrial-Revolution
Marsupialia	E0026	91.45	83.25	100.92	Cretaceous-Terrestrial-Revolution
Marsupialia	E0066	91.4	82.77	103.7	Cretaceous-Terrestrial-Revolution
Placentalia	E0066	122.51	104.93	140.97	Cretaceous-Terrestrial-Revolution
Placentalia	ALL	111.65	92.7	136.51	Cretaceous-Terrestrial-Revolution
Placentalia	E0160	110.83	96.29	127.58	Cretaceous-Terrestrial-Revolution
Placentalia	E0026	103.42	90.26	118.89	Cretaceous-Terrestrial-Revolution

bioRxiv preprint doi: https://doi.org/10.64898/2026.06.28.735080; this version posted June 29, 2026. The copyright holder for this preprint (which was not certified by peer review) is the author/funder, who has granted bioRxiv a license to display the preprint in perpetuity. It is made available under a CC-BY 4.0 International license.

Toxicofera	E0160	140.6	127.59	154.58	Cretaceous-Terrestrial-Revolution
Toxicofera	ALL	136.64	124.6	153.23	Cretaceous-Terrestrial-Revolution
Toxicofera	E0026	135.85	126.21	148.51	Cretaceous-Terrestrial-Revolution
Toxicofera	E0066	135.03	123.1	153.5	Cretaceous-Terrestrial-Revolution
Actinopteri	E0066	392.52	350.98	424.95	End-Devonian
Actinopteri	ALL	366.45	318.87	417.06	End-Devonian
Actinopteri	E0160	356.22	318.04	394.79	End-Devonian
Actinopteri	E0026	349.63	317	397.12	End-Devonian
Chondrichthyes	E0160	386.3	352.04	419.81	End-Devonian
Chondrichthyes	ALL	383.34	347.56	415.58	End-Devonian
Chondrichthyes	E0066	383.01	347.91	413.12	End-Devonian
Chondrichthyes	E0026	380.71	345.19	412.79	End-Devonian
Tetrapoda	E0066	369.93	334.08	403.79	End-Devonian
Tetrapoda	ALL	358.1	316.05	399.81	End-Devonian
Tetrapoda	E0160	357.66	325.33	400.91	End-Devonian
Tetrapoda	E0026	340.59	311.61	385.54	End-Devonian
Theria	E0066	158.78	138.36	175.33	End-Jurassic-Turnover
Theria	ALL	150.44	126.33	173.85	End-Jurassic-Turnover
Theria	E0160	145.85	126.33	179.01	End-Jurassic-Turnover
Theria	E0026	145.05	126.99	161.19	End-Jurassic-Turnover
Sarcopterygii	E0026	447.88	414.24	469.67	End-Ordovician
Sarcopterygii	E0066	444.25	409.91	468.67	End-Ordovician
Sarcopterygii	ALL	444.02	413.95	467.73	End-Ordovician
Sarcopterygii	E0160	439.1	415.78	463.04	End-Ordovician
Archosauria	E0160	258.88	238.9	278.44	End-Permian
Archosauria	ALL	257.79	236.73	277.83	End-Permian
Archosauria	E0026	257.52	234.32	278.23	End-Permian
Archosauria	E0066	257.05	238.13	276.61	End-Permian
Clupeocephala	E0160	252.36	226.12	291.24	End-Permian
Clupeocephala	ALL	250.7	225.5	301.79	End-Permian
Clupeocephala	E0026	245.74	223.96	267.88	End-Permian
Clupeocephala	E0066	258.18	231.59	306.16	End-Permian
Euteleostei	E0066	243.48	219.27	293.17	End-Permian
Euteleostei	E0160	239.02	212.24	273.36	End-Permian
Euteleostei	ALL	238.41	210.64	278.24	End-Permian
Euteleostei	E0026	230.05	212.23	252.17	End-Permian
Holostei	E0066	274.06	249.44	300.04	End-Permian
Holostei	E0160	273.56	251.99	298	End-Permian

bioRxiv preprint doi: https://doi.org/10.64898/2026.06.28.735080; this version posted June 29, 2026. The copyright holder for this preprint (which was not certified by peer review) is the author/funder, who has granted bioRxiv a license to display the preprint in perpetuity. It is made available under a CC-BY 4.0 International license.

Holostei	ALL	272.05	249.29	297.39	End-Permian
Holostei	E0026	268.88	246.9	293.07	End-Permian
Lepidosauria	E0160	266.9	243.11	288.46	End-Permian
Lepidosauria	ALL	263.27	241.13	285.25	End-Permian
Lepidosauria	E0066	261.33	239.46	284.06	End-Permian
Lepidosauria	E0026	261.12	242.17	279.54	End-Permian
Lissamphibia	E0066	264.68	241.65	289.8	End-Permian
Lissamphibia	E0160	264.16	240.83	289.38	End-Permian
Lissamphibia	ALL	263.19	239.87	290.99	End-Permian
Lissamphibia	E0026	260.51	239.14	294.43	End-Permian
Oseanacephala	E0066	297.04	255.99	332.12	End-Permian
Oseanacephala	E0160	285.64	260.73	318.99	End-Permian
Oseanacephala	ALL	278.56	162.1	331.84	End-Permian
Oseanacephala	E0026	215.43	151.81	253.99	End-Permian
Neoteleostei	E0160	197.33	176.63	221.36	End-Trassic
Neoteleostei	ALL	190.99	168.02	219.51	End-Trassic
Neoteleostei	E0066	187.94	165.67	225.75	End-Trassic
Neoteleostei	E0026	186.41	173.88	199.59	End-Trassic
Squamata	E0026	185.99	170.85	210.8	End-Triassic
Squamata	ALL	182.16	157.12	213.7	End-Triassic
Squamata	E0066	181.84	157.58	222.51	End-Triassic
Squamata	E0160	177.19	154.77	212.12	End-Triassic
Actinopterygii	E0160	408.64	357.58	439.16	Mid-Devonian
Actinopterygii	ALL	401.03	348.26	447.93	Mid-Devonian
Actinopterygii	E0066	400.63	350.41	444.94	Mid-Devonian
Actinopterygii	E0026	387.82	350.85	462.6	Mid-Devonian
Acanthomorpha	E0160	189	174.06	205.74	Mid-Jurassic
Acanthomorpha	ALL	176.88	154.6	203.02	Mid-Jurassic
Acanthomorpha	E0026	169.99	159.86	185.34	Mid-Jurassic
Acanthomorpha	E0066	165.08	151.3	198.48	Mid-Jurassic
Mammalia	E0066	179.42	162.79	195.51	Mid-Jurassic
Mammalia	ALL	176.37	160.8	192.43	Mid-Jurassic
Mammalia	E0026	174.86	163.19	188.28	Mid-Jurassic
Mammalia	E0160	174.81	156.88	188.65	Mid-Jurassic
Testudines	E0026	170.28	156.37	184.13	Mid-Jurassic
Testudines	ALL	168.44	153.92	181.4	Mid-Jurassic
Testudines	E0160	167.9	151.97	181.29	Mid-Jurassic
Testudines	E0066	167.05	155.57	178.3	Mid-Jurassic
Archelosauria	E0160	274.14	256.57	291.24	End-Permian
Archelosauria	ALL	273.27	255.54	290.68	End-Permian

bioRxiv preprint doi: https://doi.org/10.64898/2026.06.28.735080; this version posted June 29, 2026. The copyright holder for this preprint (which was not certified by peer review) is the author/funder, who has granted bioRxiv a license to display the preprint in perpetuity. It is made available under a CC-BY 4.0 International license.

Archelosauria	E0066	272.93	255.55	290.34	End-Permian
Archelosauria	E0026	272.8	255.06	290.7	End-Permian

Table S3. Input Prior Bounds for Full Analysis. Priors on individual nodes in the analysis of the 'ALL' dataset.

**Clade**	**Mean**	**Offset**	**2.5% Q**	**5% Q**	**Median**	**95% Q**	**97.5% Q**	**Distribution Type**
Acipenseriformes	133.5	0.05	121	123	133	145	147	Lognormal, Real Time
Archelosauria	283	0.05	256	260	283	307	312	Lognormal, Real Time
Archosauria	267	0.05	242	246	267	290	294	Lognormal, Real Time
Arctoidea	31	0.05	28.1	28.5	31	33.6	34.1	Lognormal, Real Time
Beryciformes	72.9	0.05	66	67.1	72.8	79.1	80.3	Lognormal, Real Time
Carnivora	41.6	0.05	37.7	38.3	41.5	45.1	45.8	Lognormal, Real Time
Cetacea	40.2	0.05	36.4	37	40.1	43.6	44.3	Lognormal, Real Time
Chiroptera	57.4	0.05	52	52.8	57.3	62.2	63.2	Lognormal, Real Time
Chondrichthyes	397	0.05	359	365	397	430	437	Lognormal, Real Time
Cypriniformes	53.9	0.05	48.8	49.6	53.8	58.4	59.4	Lognormal, Real Time
Dipnoi	154.3	0.05	140	142	154	167	170	Lognormal, Real Time
Elopomorpha	138	0.05	125	127	138	150	152	Lognormal, Real Time
Emydidae	35.3	0.05	32	32.5	35.3	38.3	38.9	Lognormal, Real Time
Ferae	70.7	0.05	64	65	70.6	76.7	77.9	Lognormal, Real Time
Galloanserae	67.5	0.05	61.1	62.1	67.4	73.2	74.4	Lognormal, Real Time
Holostei	276.9	0.05	251	255	277	300	305	Lognormal, Real Time
Labridae-Parapercis	53.6	0.05	48.5	49.3	53.5	58.1	59	Lognormal, Real Time
Lepidosauria	267	0.05	242	246	267	290	294	Lognormal, Real Time
Lepisosteidae	107.3	0.05	97.2	98.7	107	116	118	Lognormal, Real Time
Lissamphibia	262	0.05	237	241	262	284	289	Lognormal, Real Time
Lophioidei	53.6	0.05	48.5	49.3	53.5	58.1	59	Lognormal, Real Time
Mammalia	170.5	0.05	154	157	170	185	188	Lognormal, Real Time

bioRxiv preprint doi: https://doi.org/10.64898/2026.06.28.735080; this version posted June 29, 2026. The copyright holder for this preprint (which was not certified by peer review) is the author/funder, who has granted bioRxiv a license to display the preprint in perpetuity. It is made available under a CC-BY 4.0 International license.

Marsupialia	92.3	0.05	83.6	84.9	92.2	100	102	Lognormal, Real Time
Ostariophysi	134	0.05	121	123	134	145	148	Lognormal, Real Time
Osteichthyes	467	0.05	423	430	466	506	514	Lognormal, Real Time
Osteoglossomorpha	125	0.05	113	115	125	136	138	Lognormal, Real Time
Otocephala	166.8	0.05	151	153	167	181	184	Lognormal, Real Time
Otophysi	123.6	0.05	112	114	123	134	136	Lognormal, Real Time
Pan-Paenungulata	65.4	0.05	59.2	60.2	65.3	70.9	72	Lognormal, Real Time
Pan-Acrodonta	78.4	0.05	71	72.1	78.3	85	86.4	Lognormal, Real Time
Pan-Caimaninae	83.6	0.05	75.7	76.9	83.5	90.7	92.1	Lognormal, Real Time
Pan-Coliiformes	68.7	0.05	62.2	63.2	68.6	74.5	75.7	Lognormal, Real Time
Pan-Galloanserae	75.1	0.05	68	69.1	75	81.4	82.7	Lognormal, Real Time
Pan-Lacertidae	72.9	0.05	66	67.1	72.8	79.1	80.3	Lognormal, Real Time
Pan-Primates	72.3	0.05	65.5	66.5	72.2	78.4	79.6	Lognormal, Real Time
Pan-Pythonidae	51.3	0.05	46.5	47.2	51.2	55.6	56.5	Lognormal, Real Time
Pan-Scincoidea	160	0.05	145	147	160	173	176	Lognormal, Real Time
Pan-Spheniscidae	66.8	0.05	60.5	61.4	66.7	72.4	73.6	Lognormal, Real Time
Pan-Struthio	52.8	0.05	47.8	48.6	52.7	57.3	58.2	Lognormal, Real Time
Pan-Varanidae	133.5	0.05	121	123	133	145	147	Lognormal, Real Time
Pan-Zeiformes	91.7	0.05	83	84.4	91.6	99.4	101	Lognormal, Real Time
Pipoidea-Neobatrachia	163	0.05	148	150	163	177	180	Lognormal, Real Time
Rodentia	61.8	0.05	56	56.8	61.7	67	68.1	Lognormal, Real Time
Pan-Dipnoi	462	0.05	418	425	461	501	509	Lognormal, Real Time
Scombriformes	59.9	0.05	54.2	55.1	59.8	65	66	Lognormal, Real Time
Siluriformes	92.2	0.05	83.5	84.8	92.1	100	102	Lognormal, Real Time
Syngnathiformes	95	0.05	86	87.4	94.9	103	105	Lognormal, Real Time
Testudines	170.5	0.05	154	157	170	185	188	Lognormal, Real Time
Tetraodontoidei	61.8	0.05	56	56.8	61.7	67	68.1	Lognormal, Real Time
Trionychia	145	0.05	131	133	145	157	160	Lognormal, Real Time
Xenarthra	52.2	0.05	47.3	48	52.1	56.6	57.5	Lognormal, Real Time
Pan-Carangidae	61.6	0.05	55.8	56.7	61.5	66.8	67.9	Lognormal, Real Time

bioRxiv preprint doi: https://doi.org/10.64898/2026.06.28.735080; this version posted June 29, 2026. The copyright holder for this preprint (which was not certified by peer review) is the author/funder, who has granted bioRxiv a license to display the preprint in perpetuity. It is made available under a CC-BY 4.0 International license.

Table S4. Comparison of divergence time estimates for vertebrate clades estimated in the taxon exclusion rate test experiments. Mean and 95% HPDs (Min, Max) are shown. Abbreviatons: Subset, No Cal. On LF= subset analysis without node calibrations on living fossil lineages. Subset, LF with Cal. = subset analysis with node calibrations on living fossils. Subset, without LF = subset analysis excluding living fossil lineages. All mean rates (substitutions per site per Ma) are log10 transformed.

Clade	Analysis	Median	Min	Max	Rate_Root	Rate_Min	Rate_Max	Rate_Root_Log	Rate_Min_Log	Rate_Max_Log
Teleostei	Full Tree (Figure 1)	284.12	242.11	326.26	0.002556	0.001046	0.006375	-2.5924392	-2.9804683	-2.1955198
Actinopteri	Full Tree (Figure 1)	366.67	318.87	417.06	0.001167	0.000468	0.00272	-2.9329291	-3.3297541	-2.5654311
Neopterygii	Full Tree (Figure 1)	322.92	279.66	370.27	0.001791	0.000662	0.004479	-2.7469044	-3.179142	-2.3488189
Holostei	Full Tree (Figure 1)	272.79	249.29	297.39	0.000569	0.000216	0.00165	-3.2448877	-3.6655462	-2.7825161
Lepisosteidae	Full Tree (Figure 1)	104.51	95.03	114.56	0.000594	0.000484	0.000719	-3.2262136	-3.3151546	-3.1432711
Acipenseriformes	Full Tree (Figure 1)	131.54	118.87	144.76	0.000442	0.000308	0.00116	-3.3545777	-3.5114493	-2.935542
Actinopterygii	Full Tree (Figure 1)	399.56	348.26	417.06	0.001425	0.000575	0.002946	-2.8461851	-3.2403322	-2.5307673
Dipnoi	Full Tree (Figure 1)	154.07	139.35	168.66	0.0004	0.000323	0.000483	-3.39794	-3.4907975	-3.3160529
Teleostei	Subset, No Cal. On LF	228.34	187.45	439.13	0.001685	0.000313	0.020984	-2.7734001	-3.5044557	-1.6781117
Actinopteri	Subset, No Cal. On LF	327.55	291.68	442.71	0.001814	0.000533	0.064645	-2.7413627	-3.2732728	-1.1894651
Neopterygii	Subset, No Cal. On LF	284.57	258.9	440.96	0.002516	0.000858	0.066502	-2.5992894	-3.0665127	-1.1771653
Holostei	Subset, No Cal. On LF	257.73	116.06	282.36	0.001394	0.000098	0.024633	-2.8557372	-4.0087739	-1.6084827
Lepisosteidae	Subset, No Cal. On LF	9.8	2.16	31.72	0.000392	0.000323	0.000991	-3.4067139	-3.4907975	-3.0039263
Acipenseriformes	Subset, No Cal. On LF	22.92	5.01	52.89	0.000369	0.000237	0.002368	-3.4329736	-3.6252517	-2.6256183
Actinopterygii	Subset, No Cal. On LF	378.68	327.49	443.66	0.001955	0.000013	0.018144	-2.7088532	-4.8860566	-1.741267
Dipnoi	Subset, No Cal. On LF	137.15	35.55	257.7	0.000398	0.000273	0.000708	-3.4001169	-3.5638374	-3.1499667
Teleostei	Subset, LF with Cal.	249.25	197.4	313.5	0.001909	0.00052	0.005589	-2.7191941	-3.2839967	-2.2526659
Actinopteri	Subset, LF with Cal.	321.82	274.68	369.55	0.001509	0.0004	0.005112	-2.8213108	-3.39794	-2.2914092
Neopterygii	Subset, LF with Cal.	292.11	263.66	331.31	0.003521	0.000903	0.013466	-2.453334	-3.0443123	-1.8707614
Holostei	Subset, LF with Cal.	261.69	48.63	287.09	0.001202	0.000211	0.198062	-2.9200955	-3.6757175	-0.7031988
Lepisosteidae	Subset, LF with Cal.	101.91	47.4	112.05	0.000611	0.000407	0.094379	-3.2139588	-3.3904056	-1.0251246
Acipenseriformes	Subset, LF with Cal.	132.33	119.27	145.81	0.000555	0.000344	0.000742	-3.255707	-3.4634416	-3.1295961
Actinopterygii	Subset, LF with Cal.	377.23	321.05	443.73	0.00202	0.000008	0.009097	-2.6946486	-5.09691	-2.0411018
Dipnoi	Subset, LF with Cal.	121.52	21.02	303.91	0.000391	0.000262	0.000964	-3.4078232	-3.5816987	-3.015923

Teleostei	Subset, without LF	220.93	191.9	252.2	0.001819	0.000918	0.00346	-2.7401673	-3.0371573	-2.4609239
Actinopterygii	Subset, without LF	312.28	257.64	376.84	0.001135	0.000514	0.002419	-2.9450041	-3.2890369	-2.6163641
Dipnoi	Subset, without LF	145.66	56.31	227.41	0.000409	0.000293	0.000598	-3.3882767	-3.5331324	-3.2232988

bioRxiv preprint doi: https://doi.org/10.64898/2026.06.28.735080; this version posted June 29, 2026. The copyright holder for this preprint (which was not certified by peer review) is the author/funder, who has granted bioRxiv a license to display the preprint in perpetuity. It is made available under a CC-BY 4.0 International license.

III. References.

A.f B. 2004. THE FIRST DISCOVERY OF AN ANGLERFISH (TELEOSTEI, LOPHIIDAE) IN THE EOCENE OF THE NORTHERN CAUCASUS. Available from: https://repository.geologyscience.ru/handle/123456789/39456

Alfaro ME, Faircloth BC, Harrington RC, Sorenson L, Friedman M, Thacker CE, Oliveros CH, Černý D, Near TJ. 2018. Explosive diversification of marine fishes at the Cretaceous–Palaeogene boundary. *Nat. Ecol. Evol.* 2:688–696.

Álvarez-Carretero S, Tamuri AU, Battini M, Nascimento FF, Carlisle E, Asher RJ, Yang Z, Donoghue PCJ, dos Reis M. 2022. A species-level timeline of mammal evolution integrating phylogenomic data. *Nature* 602:263–267.

Alves YM, Bergqvist LP, Brito PM. 2019. The dorsal and pectoral fin spines of catfishes (Ostariophysi: Siluriformes) from the Bauru Group (Late Cretaceous), Brazil: A comparative and critical analysis. *J. South Am. Earth Sci.* 92:32–40.

Amaral CR, Alvarado-Ortega J, Brito PM. 2013. Sapperichthys gen. nov., a new gonorynchid from the Cenomanian of Chiapas, Mexico. *Mesoz. Fishes* 5:305–323.

and, ,. 2017. Morphological and histological evidence for the oldest known softshell turtles from Japan. *J. Vertebr. Paleontol.* 37:e1278606.

Anderson JS, Reisz RR, Scott D, Fröbisch NB, Sumida SS. 2008. A stem batrachian from the Early Permian of Texas and the origin of frogs and salamanders. *Nature* 453:515–518.

Andrews JV, Schein JP, Friedman M. 2023. An earliest Paleocene squirrelfish (Teleostei: Beryciformes: Holocentroidea) and its bearing on the timescale of holocentroid evolution. *J. Syst. Palaeontol.* 21:2168571.

Anon. 1998. A NEW PALEOCENE ARMADILLO (MAMMALIA, DASYPODOIDEA) FROM THE ITABORAÍ BASIN, BRAZIL. *Publ. Electrónica Asoc. Paleontológica Argent.* [Internet] 5. Available from: https://peapaleontologica.org.ar/index.php/peapa/article/view/174

Anon. 2019. Mystacodon selenensis, the earliest known toothed mysticete (Cetacea, Mammalia) from the late Eocene of Peru: anatomy, phylogeny, and feeding adaptations. *Geodiversitas* 41:401–499.

Anon. Abstract: THE AGE OF DINOSAUR-BEARING STRATA AT PHOEBUS LANDING, CAPE FEAR RIVER, NORTH CAROLINA (Northeastern Section (39th Annual) and Southeastern Section (53rd Annual) Joint Meeting (March 25–27, 2004)). Available from: https://gsa.confex.com/gsa/2004NE/webprogram/Paper69560.html

bioRxiv preprint doi: https://doi.org/10.64898/2026.06.28.735080; this version posted June 29, 2026. The copyright holder for this preprint (which was not certified by peer review) is the author/funder, who has granted bioRxiv a license to display the preprint in perpetuity. It is made available under a CC-BY 4.0 International license.

Arcila D, Ortí G, Vari R, Armbruster JW, Stiassny MLJ, Ko KD, Sabaj MH, Lundberg J, Revell LJ, Betancur-R. R. 2017. Genome-wide interrogation advances resolution of recalcitrant groups in the tree of life. *Nat. Ecol. Evol.* 1:1–10.

Argyriou T, Giles S, Friedman M. A Permian fish reveals widespread distribution of neopterygian-like jaw suspension. *eLife* 11:e58433.

Argyriou T, Giles S, Friedman M, Romano C, Kogan I, Sánchez-Villagra MR. 2018. Internal cranial anatomy of Early Triassic species of †Saurichthys (Actinopterygii: †Saurichthyiformes): implications for the phylogenetic placement of †saurichthyiforms. *BMC Evol. Biol.* 18:161.

Arratia G. 2000. Remarkable teleostean fishes from the Late Jurassic of southern Germany and their phylogenetic relationships. *Foss. Rec.* 3:137–179.

Ascarrunz E, Rage J-C, Legreneur P, Laurin M. 2016. Triadobatrachus massinoti, the earliest known lissamphibian (Vertebrata: Tetrapoda) re-examined by µCT scan, and the evolution of trunk length in batrachians. *Contrib. Zool.* 85:201-223.

Avilla LS, Mothé D. 2021. Out of Africa: A New Afrotheria Lineage Rises From Extinct South American Mammals. *Front. Ecol. Evol.* [Internet] 9. Available from: https://www.frontiersin.org/journals/ecology-and-evolution/articles/10.3389/fevo.2021.654302/full

Báez AM. 2013. Anurans from the Early Cretaceous Lagerstätte of Las Hoyas, Spain: New evidence on the Mesozoic diversification of crown-clade Anura. *Cretac. Res.* 41:90–106.

Bagley JC, Mayden RL, Harris PM. 2018. Phylogeny and divergence times of suckers (Cypriniformes: Catostomidae) inferred from Bayesian total-evidence analyses of molecules, morphology, and fossils. *PeerJ* [Internet] 6. Available from: https://www.ncbi.nlm.nih.gov/pmc/articles/PMC6035723/

Bannikov AF, Carnevale G. 2010. Bellwoodilabrus landinii n. gen., n. sp., a new genus and species of labrid fish (Teleostei, Perciformes) from the Eocene of Monte Bolca. *Geodiversitas* 32:201–220.

Beck RMD, Godthelp H, Weisbecker V, Archer M, Hand SJ. 2008. Australia's Oldest Marsupial Fossils and their Biogeographical Implications. *PLoS ONE* 3:e1858.

Becker RT, Gradstein FM, Hammer O. 2012. Chapter 22 - The Devonian Period. In: Gradstein Felix M., Ogg JG, Schmitz MD, Ogg GM, editors. The Geologic Time Scale. Boston: Elsevier. p. 559–601. Available from: https://www.sciencedirect.com/science/article/pii/B9780444594259000226

bioRxiv preprint doi: https://doi.org/10.64898/2026.06.28.735080; this version posted June 29, 2026. The copyright holder for this preprint (which was not certified by peer review) is the author/funder, who has granted bioRxiv a license to display the preprint in perpetuity. It is made available under a CC-BY 4.0 International license.

Bellwood D. 1990. A new fossil fish Phyllopharyngodon longipinnis gen. et sp. nov. (family Labridae) from the Eocene, Monte Bolca, Italy. *Studi E Ric. Sui Giacimenti Terziari Bolca* 6:149–160.

Benito J, Chen A, Wilson LE, Bhullar B-AS, Burnham D, Field DJ. 2022. Forty new specimens of Ichthyornis provide unprecedented insight into the postcranial morphology of crownward stem group birds. *PeerJ* 10:e13919.

Benito J, Kuo P-C, Widrig KE, Jagt JWM, Field DJ. 2022. Cretaceous ornithurine supports a neognathous crown bird ancestor. *Nature* 612:100–105.

Benton MJ, Donoghue PC, Asher RJ, Friedman M, Near TJ, Vinther J. 2015. Constraints on the timescale of animal evolutionary history. *Palaeontol Electron* 18:1–106.

Benton MJ, Donoghue PCJ. 2007. Paleontological Evidence to Date the Tree of Life. *Mol. Biol. Evol.* 24:26–53.

Bergqvist LP, Pereira PVLG, Machado AS, Castro MCD, Melki LB, Lopes RT. 2019. Osteoderm microstructure of Riostegotherium yanei, the oldest Xenarthra. *An. Acad. Bras. Ciênc.* 91:e20181290.

Bernardi M, Klein H, Petti FM, Ezcurra MD. 2015. The Origin and Early Radiation of Archosauriforms: Integrating the Skeletal and Footprint Record. *PLoS ONE* 10:e0128449.

Berta A. 1991. New <I>Enaliarctos</I>* (Pinnipedimorpha) from the Oligocene and Miocene of Oregon and the Role of “Enaliarctids” in Pinniped Phylogeny. Available from: http://repository.si.edu/xmlui/handle/10088/19145

Berv JS, Field DJ. 2018. Genomic Signature of an Avian Lilliput Effect across the K-Pg Extinction. *Syst. Biol.* 67:1–13.

Berv JS, Singhal S, Field DJ, Walker-Hale N, McHugh SW, Shipley JR, Miller ET, Kimball RT, Braun EL, Dornburg A, et al. 2024. Genome and life-history evolution link bird diversification to the end-Cretaceous mass extinction. *Sci. Adv.* 10:eadp0114.

Betancur-R. R, Arcila D, Vari RP, Hughes LC, Oliveira C, Sabaj MH, Ortí G. 2019. Phylogenomic incongruence, hypothesis testing, and taxonomic sampling: The monophyly of characiform fishes*. *Evolution* 73:329–345.

Betancur-R R, Broughton RE, Wiley EO, Carpenter K, López JA, Li C, Holcroft NI, Arcila D, Sanciangco M, Ii JCC, et al. 2013. The Tree of Life and a New Classification of Bony Fishes. *PLOS Curr. Tree Life* [Internet]. Available from: http://currents.plos.org/treeoflife/article/the-tree-of-life-and-a-new-classification-of-bony-fishes/

bioRxiv preprint doi: https://doi.org/10.64898/2026.06.28.735080; this version posted June 29, 2026. The copyright holder for this preprint (which was not certified by peer review) is the author/funder, who has granted bioRxiv a license to display the preprint in perpetuity. It is made available under a CC-BY 4.0 International license.

Bever GS, Lyson TR, Field DJ, Bhullar B-AS. 2015. Evolutionary origin of the turtle skull. *Nature* 525:239–242.

Bi X, Wang K, Yang L, Pan H, Jiang Haifeng, Wei Q, Fang M, Yu H, Zhu C, Cai Y, et al. 2021a. Tracing the genetic footprints of vertebrate landing in non-teleost ray-finned fishes. *Cell* 184:1377-1391.e14.

Bi X, Wang K, Yang L, Pan H, Jiang Haifeng, Wei Q, Fang M, Yu H, Zhu C, Cai Y, et al. 2021b. Tracing the genetic footprints of vertebrate landing in non-teleost ray-finned fishes. *Cell* 184:1377-1391.e14.

Bian C, Hu Y, Ravi V, Kuznetsova IS, Shen X, Mu X, Sun Y, You X, Li J, Li X, et al. 2016. The Asian arowana (Scleropages formosus) genome provides new insights into the evolution of an early lineage of teleosts. *Sci. Rep.* 6:1–17.

Bittencourt JS, Simões TR, Caldwell MW, Langer MC. 2020. Discovery of the oldest South American fossil lizard illustrates the cosmopolitanism of early South American squamates. *Commun. Biol.* 3:1–11.

Boessenecker RW, Beatty BL, Geisler JH. 2023. New specimens and species of the Oligocene toothed baleen whale Coronodon from South Carolina and the origin of Neoceti. *PeerJ* 11:e14795.

Bona P, Ezcurra MD, Barrios F, Fernandez Blanco MV. 2018. A new Palaeocene crocodylian from southern Argentina sheds light on the early history of caimanines. *Proc. R. Soc. B Biol. Sci.* 285:20180843.

Borsuk-Bialynicka M, Moody SM. 1984. Priscagaminae, a new subfamily of the Agamidae (Sauria) from the Late Cretaceous of the Gobi Desert. *Acta Palaeontol. Pol.* [Internet] 29. Available from: https://agro.icm.edu.pl/agro/element/bwmeta1.element.agro-a23634c5-3ba7-4810-86b8-46315cd4a85a

Bowman J, Enard D, Lynch VJ. 2023. Phylogenomics reveals an almost perfect polytomy among the almost ungulates (Paenungulata). *bioRxiv*:2023.12.07.570590.

Braasch I, Gehrke AR, Smith JJ, Kawasaki K, Manousaki T, Pasquier J, Amores A, Desvignes T, Batzel P, Catchen J, et al. 2016. The spotted gar genome illuminates vertebrate evolution and facilitates human-teleost comparisons. *Nat. Genet.* 48:427–437.

Brazeau MD, Giles S, Dearden RP, Jerve A, Ariunchimeg Y, Zorig E, Sansom R, Guillerme T, Castiello M. 2020. Endochondral bone in an Early Devonian ‘placoderm’ from Mongolia. *Nat. Ecol. Evol.* 4:1477–1484.

Brinkmann H, Venkatesh B, Brenner S, Meyer A. 2004. Nuclear protein-coding genes support lungfish and not the coelacanth as the closest living relatives of land vertebrates. *Proc. Natl. Acad. Sci.* 101:4900–4905.

bioRxiv preprint doi: https://doi.org/10.64898/2026.06.28.735080; this version posted June 29, 2026. The copyright holder for this preprint (which was not certified by peer review) is the author/funder, who has granted bioRxiv a license to display the preprint in perpetuity. It is made available under a CC-BY 4.0 International license.

Brito PM, Alvarado-Ortega J, Meunier FJ. 2017. Earliest known lepisosteoid extends the range of anatomically modern gars to the Late Jurassic. *Sci. Rep.* 7:17830.

Brito PM, Figueiredo FJ, Leal MEC. 2020. A revision of Laeliichthys ancestralis Santos, 1985 (Teleostei: Osteoglossomorpha) from the Lower Cretaceous of Brazil: Phylogenetic relationships and biogeographical implications. *PLOS ONE* 15:e0241009.

Brochu CA. 1997. Morphology, Fossils, Divergence Timing, and the Phylogenetic Relationships of Gavialis. *Syst. Biol.* 46:479–522.

Brownstein CD. 2018. The distinctive theropod assemblage of the Ellisdale site of New Jersey and its implications for North American dinosaur ecology and evolution during the Cretaceous. *J. Paleontol.* 92:1115–1129.

Brownstein CD. 2019. First Record of a Small Juvenile Giant Crocodyliform and its Ontogenetic and Biogeographic Implications. *Bull. Peabody Mus. Nat. Hist.* 60:81–90.

Brownstein Chase Doran. 2023. Palaeospondylus and the early evolution of gnathostomes. *Nature* 620:E20–E22.

Brownstein C D. 2023. Syngnathoid Evolutionary History and the Conundrum of Fossil Misplacement. *Integr. Org. Biol.* 5:obad011.

Brownstein CD, Dornburg A, Near TJ. 2025. Cenozoic evolutionary history obscures the Mesozoic origins of acanthopterygian fishes. *Evolution*:qpaf040.

Brownstein CD, Lyson TR. 2022. Giant gar from directly above the Cretaceous–Palaeogene boundary suggests healthy freshwater ecosystems existed within thousands of years of the asteroid impact. *Biol. Lett.* 18:20220118.

Brownstein CD, MacGuigan DJ, Kim D, Orr O, Yang L, David SR, Kreiser B, Near TJ. 2024. The genomic signatures of evolutionary stasis. *Evolution* 78:821–834.

Brownstein CD, Meyer DL, Fabbri M, Bhullar B-AS, Gauthier JA. 2022. Evolutionary origins of the prolonged extant squamate radiation. *Nat. Commun.* 13:7087.

Brownstein CD, Near TJ. 2024. A giant bowfin from a Paleocene hothouse ecosystem in North America. *Zool. J. Linn. Soc.* 202:zlae042.

Brownstein CD, Near TJ, Dearden RP. 2024. The Palaeozoic assembly of the holocephalan body plan far preceded post-Cretaceous radiations into the ocean depths. *Proc. R. Soc. B Biol. Sci.* 291:20241824.

Brownstein Chase D., Simões TR, Caldwell MW, Lee MSY, Meyer DL, Scarpetta SG. 2023. The affinities of the Late Triassic *Cryptovaranoides* and the age of crown squamates. *R. Soc. Open Sci.* 10:230968.

bioRxiv preprint doi: https://doi.org/10.64898/2026.06.28.735080; this version posted June 29, 2026. The copyright holder for this preprint (which was not certified by peer review) is the author/funder, who has granted bioRxiv a license to display the preprint in perpetuity. It is made available under a CC-BY 4.0 International license.

Brownstein Chase Doran, Yang L, Friedman M, Near TJ. 2023. Phylogenomics of the Ancient and Species-Depauperate Gars Tracks 150 Million Years of Continental Fragmentation in the Northern Hemisphere. *Syst. Biol.* 72:213–227.

Brownstein CD, Zapfe KL, Lott S, Harrington RC, Ghezelayagh A, Dornburg A, Near TJ. 2024. Synergistic innovations enabled the radiation of anglerfishes in the deep open ocean. *Curr. Biol.* 34:2541-2550.e4.

Budd GE, Mann RP. 2023. Two Notorious Nodes: A Critical Examination of Relaxed Molecular Clock Age Estimates of the Bilaterian Animals and Placental Mammals. *Syst. Biol.*:syad057.

Burbrink FT, Grazziotin FG, Pyron RA, Cundall D, Donnellan S, Irish F, Keogh JS, Kraus F, Murphy RW, Noonan B, et al. 2020. Interrogating Genomic-Scale Data for Squamata (Lizards, Snakes, and Amphisbaenians) Shows no Support for Key Traditional Morphological Relationships. *Syst. Biol.* 69:502–520.

Caldwell MW, Nydam RL, Palci A, Apesteguía S. 2015. The oldest known snakes from the Middle Jurassic-Lower Cretaceous provide insights on snake evolution. *Nat. Commun.* 6:5996.

Capobianco A, Friedman M. 2024. Fossils indicate marine dispersal in osteoglossid fishes, a classic example of continental vicariance. *Proc. R. Soc. B Biol. Sci.* 291:20241293.

Capobianco A, Zouhri S, Friedman M. 2025. A long-snouted marine bonytongue (Teleostei: Osteoglossidae) from the early Eocene of Morocco and the phylogenetic affinities of marine osteoglossids. *Zool. J. Linn. Soc.* 203:zlae015.

Carnevale G, Pietsch TW. 2009. An Eocene Frogfish from Monte Bolca, Italy: The Earliest Known Skeletal Record for the Family. *Palaeontology* 52:745–752.

Carnevale G, Pietsch TW. 2010. Eocene handfishes from Monte Bolca, with description of a new genus and species, and a phylogeny of the family Brachionichthyidae (Teleostei: Lophiiformes). *Zool. J. Linn. Soc.* 160:621–647.

Carnevale G, Pietsch TW. 2011. Batfishes from the Eocene of Monte Bolca. *Geol. Mag.* 148:461–472.

Casanovas-Vilar I, Garcia-Porta J, Fortuny J, Sanisidro Ó, Prieto J, Querejeta M, Llácer S, Robles JM, Bernardini F, Alba DM. Oldest skeleton of a fossil flying squirrel casts new light on the phylogeny of the group. *eLife* 7:e39270.

CAVIN L, SUTEETHORN V, BUFFETAUT E, TONG H. 2007. A new Thai Mesozoic lungfish (Sarcopterygii, Dipnoi) with an insight into post-Palaeozoic dipnoan evolution. *Zool. J. Linn. Soc.* 149:141–177.

bioRxiv preprint doi: https://doi.org/10.64898/2026.06.28.735080; this version posted June 29, 2026. The copyright holder for this preprint (which was not certified by peer review) is the author/funder, who has granted bioRxiv a license to display the preprint in perpetuity. It is made available under a CC-BY 4.0 International license.

Celerino de Carvalho J, Santucci RM. 2024. A new fossil Squamata from the Quiricó Formation (Lower Cretaceous), Sanfranciscana Basin, Minas Gerais, Brazil. *Cretac. Res.* 154:105717.

Chakrabarty P, Faircloth BC, Alda F, Ludt WB, Mcmahan CD, Near TJ, Dornburg A, Albert JS, Arroyave J, Stiassny MLJ, et al. 2017. Phylogenomic Systematics of Ostariophysan Fishes: Ultraconserved Elements Support the Surprising Non-Monophyly of Characiformes. *Syst. Biol.* 66:881–895.

Chen M-Y, Liang D, Zhang P. 2015. Selecting Question-Specific Genes to Reduce Incongruence in Phylogenomics: A Case Study of Jawed Vertebrate Backbone Phylogeny. *Syst. Biol.* 64:1104–1120.

Chester SGB, Bloch JI, Boyer DM, Clemens WA. 2015. Oldest known euarchontan tarsals and affinities of Paleocene Purgatorius to Primates. *Proc. Natl. Acad. Sci.* 112:1487–1492.

Chester SGB, Williamson TE, Bloch JI, Silcox MT, Sargis EJ. 2017. Oldest skeleton of a plesiadapiform provides additional evidence for an exclusively arboreal radiation of stem primates in the Palaeocene. *R. Soc. Open Sci.* 4:170329.

Chiari Y, Cahais V, Galtier N, Delsuc F. 2012. Phylogenomic analyses support the position of turtles as the sister group of birds and crocodiles (Archosauria). *BMC Biol.* 10:65.

Choo B, Zhu M, Qu Q, Yu X, Jia L, Zhao W. 2017. A new osteichthyan from the late Silurian of Yunnan, China. *PLOS ONE* 12:e0170929.

Claramunt S, Cracraft J. 2015. A new time tree reveals Earth history's imprint on the evolution of modern birds. *Sci. Adv.* 1:e1501005.

Clarke JA, Norell MA, Dashzeveg D. 2005. New Avian Remains from the Eocene of Mongolia and the Phylogenetic Position of the Eogruidae (Aves, Gruoidea). *Am. Mus. Novit.* 2005:1–17.

Cloutier R, Clement AM, Lee MSY, Noël R, Béchard I, Roy V, Long JA. 2020. Elpistostege and the origin of the vertebrate hand. *Nature* 579:549–554.

Coates MI, Finarelli JA, Sansom IJ, Andreev PS, Criswell KE, Tietjen K, Rivers ML, La Riviere PJ. 2018. An early chondrichthyan and the evolutionary assembly of a shark body plan. *Proc. R. Soc. B Biol. Sci.* 285:20172418.

Cole TL, Zhou C, Fang M, Pan H, Ksepka DT, Fiddaman SR, Emerling CA, Thomas DB, Bi X, Fang Q, et al. 2022. Genomic insights into the secondary aquatic transition of penguins. *Nat. Commun.* 13:3912.

Conrad JL. 2008. Phylogeny And Systematics Of Squamata (Reptilia) Based On Morphology. *Bull. Am. Mus. Nat. Hist.* 2008:1–182.

bioRxiv preprint doi: https://doi.org/10.64898/2026.06.28.735080; this version posted June 29, 2026. The copyright holder for this preprint (which was not certified by peer review) is the author/funder, who has granted bioRxiv a license to display the preprint in perpetuity. It is made available under a CC-BY 4.0 International license.

Cossette AP, Brochu CA. 2020. A systematic review of the giant alligatoroid Deinosuchus from the Campanian of North America and its implications for the relationships at the root of Crocodylia. *J. Vertebr. Paleontol.* 40:e1767638.

Crane A, Benito J, Chen A, Musser G, Torres CR, Clarke JA, Lautenschlager S, Ksepka DT, Field DJ. 2024. Taphonomic damage obfuscates interpretation of the retroarticular region of the *Asteriornis* mandible. *Geobios* [Internet]. Available from: https://www.sciencedirect.com/science/article/pii/S0016699524000536

Crawford NG, Faircloth BC, McCormack JE, Brumfield RT, Winker K, Glenn TC. 2012. More than 1000 ultraconserved elements provide evidence that turtles are the sister group of archosaurs. *Biol. Lett.* 8:783–786.

Croghan JA, Palci A, Onary S, Lee MSY, Caldwell MW. 2024. Morphology and systematics of a new fossil snake from the early Rupelian (Oligocene) White River Formation, Wyoming. *Zool. J. Linn. Soc.*:zlae073.

Cui X, Friedman M, Qiao T, Yu Y, Zhu M. 2022. The rapid evolution of lungfish durophagy. *Nat. Commun.* 13:2390.

Cui X, Friedman M, Yu Y, Zhu Y, Zhu M. 2023. Bony-fish-like scales in a Silurian maxillate placoderm. *Nat. Commun.* 14:7622.

Cui X, Qiao T, Zhu M. 2019. Scale morphology and squamation pattern of Guiyu oneiros provide new insights into early osteichthyan body plan. *Sci. Rep.* 9:4411.

Darlim G, Lee MSY, Walter J, Rabi M. 2022. The impact of molecular data on the phylogenetic position of the putative oldest crown crocodilian and the age of the clade. *Biol. Lett.* 18:20210603.

Davesne D, Carnevale G, Friedman M. 2017. Bajaichthys elegans from the Eocene of Bolca (Italy) and the overlooked morphological diversity of Zeiformes (Teleostei, Acanthomorpha). *Palaeontology* 60:255–268.

Davesne D, Friedman M, Schmitt AD, Fernandez V, Carnevale G, Ahlberg PE, Sanchez S, Benson RBJ. 2021. Fossilized cell structures identify an ancient origin for the teleost whole-genome duplication. *Proc. Natl. Acad. Sci.* 118:e2101780118.

Dawson MR, Beard KC. 1996. New late Paleocene rodents (Mammalia) from big multi quarry, Washakie Basin, Wyoming. *Palaeovertebrata* 25:301–321.

Dawson MR, Janis CM, Gunnell GF, Uhen MD. 2008. Evolution of tertiary mammals of North America.

bioRxiv preprint doi: https://doi.org/10.64898/2026.06.28.735080; this version posted June 29, 2026. The copyright holder for this preprint (which was not certified by peer review) is the author/funder, who has granted bioRxiv a license to display the preprint in perpetuity. It is made available under a CC-BY 4.0 International license.

Denton R. 2022. ALBEMARLE SOUND NC - A MODERN ANALOG FOR THE ELLISDALE FOSSIL SITE (LATE CRETACEOUS, CAMPANIAN, NJ). In: GSA. Available from: https://gsa.confex.com/gsa/2022NC/webprogram/Paper373518.html

Dingus L, Loope D, Dashzeveg D, Swisher C, Minjin C, Novacek M, Norell M. 2008. The Geology of Ukhaa Tolgod (Djadokhta Formation, Upper Cretaceous, Nemegt Basin, Mongolia). *Dep. Earth Atmospheric Sci. Fac. Publ.* [Internet]. Available from: https://digitalcommons.unl.edu/geosciencefacpub/217

Diogo R. 2009. GRANDE, T., F. POYATO-ARIZA & R. DIOGO. (2009). Gonorynchiform interrelationships: historical overview, analysis, and revised systematics of the group. In: Grande, T., F. Poyato-Ariza & R. Diogo (eds.), Gonorynchiformes and ostariophysan relationships – a comprehensive review, Science Publishers and Taylor & Francis (Oxford, UK): 221-231.

Dornburg A, Friedman M, Near TJ. 2015. Phylogenetic analysis of molecular and morphological data highlights uncertainty in the relationships of fossil and living species of Elopomorpha (Actinopterygii: Teleostei). *Mol. Phylogenet. Evol.* 89:205–218.

Dornburg A, Near TJ. 2021. The Emerging Phylogenetic Perspective on the Evolution of Actinopterygian Fishes. *Annu. Rev. Ecol. Evol. Syst.* 52:427–452.

Dornburg A, Townsend JP, Brooks W, Spriggs E, Eytan RI, Moore JA, Wainwright PC, Lemmon A, Lemmon EM, Near TJ. 2017. New insights on the sister lineage of percomorph fishes with an anchored hybrid enrichment dataset. *Mol. Phylogenet. Evol.* 110:27–38.

Du K, Stöck M, Kneitz S, Klopp C, Woltering JM, Adolfi MC, Feron R, Prokopov D, Makunin A, Kichigin I, et al. 2020. The sterlet sturgeon genome sequence and the mechanisms of segmental rediploidization. *Nat. Ecol. Evol.* 4:841–852.

Esselstyn JA, Oliveros CH, Swanson MT, Faircloth BC. 2017. Investigating Difficult Nodes in the Placental Mammal Tree with Expanded Taxon Sampling and Thousands of Ultraconserved Elements. *Genome Biol. Evol.* 9:2308–2321.

Estes R, de Queiroz K, Gauthier J. 1988. Phylogenetic relationships within Squamata. In: Phylogenetic Relationships of the lizard families. Stanford, California: Stanford University Press. p. 119–281. Available from: https://books.google.com/books?hl=en&lr=&id=h5fIP1X7YvoC&oi=fnd&pg=PA119&ots=fW_Kh2cy1A&sig=Ukv57Cm2U52V1_tXWHr5fGB15b4

Evans SE, Borsuk-Bialynicka M. 1998. A stem-group frog from the Early Triassic of Poland. *Acta Palaeontol. Pol.* 43:573–580.

Evans SE, Raia P, Barbera C. 2006. The Lower Cretaceous lizard genus *Chometokadmon* from Italy. *Cretac. Res.* 27:673–683.

bioRxiv preprint doi: https://doi.org/10.64898/2026.06.28.735080; this version posted June 29, 2026. The copyright holder for this preprint (which was not certified by peer review) is the author/funder, who has granted bioRxiv a license to display the preprint in perpetuity. It is made available under a CC-BY 4.0 International license.

Ezcurra MD, Nesbitt SJ, Bronzati M, Dalla Vecchia FM, Agnolin FL, Benson RBJ, Brissón Egli F, Cabreira SF, Evers SW, Gentil AR, et al. 2020. Enigmatic dinosaur precursors bridge the gap to the origin of Pterosauria. *Nature* 588:445–449.

Ezcurra MD, Scheyer TM, Butler RJ. 2014. The Origin and Early Evolution of Sauria: Reassessing the Permian Saurian Fossil Record and the Timing of the Crocodile-Lizard Divergence. *PLOS ONE* 9:e89165.

Field DJ, Benito J, Chen A, Jagt JWM, Ksepka DT. 2020. Late Cretaceous neornithine from Europe illuminates the origins of crown birds. *Nature* 579:397–401.

Field DJ, Bercovici A, Berv JS, Dunn R, Fastovsky DE, Lyson TR, Vajda V, Gauthier JA. 2018. Early Evolution of Modern Birds Structured by Global Forest Collapse at the End-Cretaceous Mass Extinction. *Curr. Biol.* 28:1825-1831.e2.

de Figueiredo FJ, Gallo V, Leal MEC. 2012. Phylogenetic relationships of the elopomorph fish †*Paraelops cearensis* Silva Santos revisited: Evidence from new specimens. *Cretac. Res.* 37:148–154.

Filleul A, Maisey JG. 2004. Redescription of Santanichthys diasii (Otophysi, Characiformes) from the Albian of the Santana Formation and comments on its implications for otophysan relationships. American Museum novitates ; no. 3455. Available from: http://hdl.handle.net/2246/2765

Foley NM, Mason VC, Harris AJ, Bredemeyer KR, Damas J, Lewin HA, Eizirik E, Gatesy J, Karlsson EK, Lindblad-Toh K, et al. 2023. A genomic timescale for placental mammal evolution. *Science* 380:eabl8189.

Ford DP, Evans SE, Choiniere JN, Fernandez V, Benson RBJ. 2021. A reassessment of the enigmatic diapsid *Paliguana whitei* and the early history of Lepidosauromorpha. *Proc. R. Soc. B Biol. Sci.* 288:20211084.

Fordyce RE, Marx FG. 2018. Gigantism Precedes Filter Feeding in Baleen Whale Evolution. *Curr. Biol.* 28:1670-1676.e2.

Fox RC, Youzwyshyn GP. 1994. New primitive carnivorans (Mammalia) from the Paleocene of western Canada, and their bearing on relationships of the order. *J. Vertebr. Paleontol.* 14:382–404.

Freisem LS, Müller J, Sues H-D, Sobral G. 2024. A new sphenodontian (Diapsida: Lepidosauria) from the Upper Triassic (Norian) of Germany and its implications for the mode of sphenodontian evolution. *BMC Ecol. Evol.* 24:35.

Frey L, Coates M, Ginter M, Hairapetian V, Rücklin M, Jerjen I, Klug C. 2019. The early elasmobranch Phoebodus: phylogenetic relationships, ecomorphology and a new time-scale for shark evolution. *Proc. R. Soc. B Biol. Sci.* 286:20191336.

bioRxiv preprint doi: https://doi.org/10.64898/2026.06.28.735080; this version posted June 29, 2026. The copyright holder for this preprint (which was not certified by peer review) is the author/funder, who has granted bioRxiv a license to display the preprint in perpetuity. It is made available under a CC-BY 4.0 International license.

Frey L, Coates MI, Tietjen K, Rücklin M, Klug C. 2020. A symmoriiform from the Late Devonian of Morocco demonstrates a derived jaw function in ancient chondrichthyans. *Commun. Biol.* 3:681.

FRIEDMAN M. 2007. The interrelationships of Devonian lungfishes (Sarcopterygii: Dipnoi) as inferred from neurocranial evidence and new data from the genus Soederberghia Lehman, 1959. *Zool. J. Linn. Soc.* 151:115–171.

Friedman M. 2022. The Macroevolutionary History of Bony Fishes: A Paleontological View. *Annu. Rev. Ecol. Evol. Syst.* 53:353–377.

Friedman M, Carnevale G. 2018. The Bolca Lagerstätten: shallow marine life in the Eocene. *J. Geol. Soc.* 175:569–579.

Friedman M, Feilich KL, Beckett HT, Alfaro ME, Faircloth BC, Černý D, Miya M, Near TJ, Harrington RC. 2019. A phylogenomic framework for pelagiarian fishes (Acanthomorpha: Percomorpha) highlights mosaic radiation in the open ocean. *Proc. R. Soc. B Biol. Sci.* 286:20191502.

de la Fuente MS, Iturralde-Vinent M. 2001. A New Pleurodiran Turtle from the Jagua Formation (Oxfordian) of Western Cuba. *J. Paleontol.* 75:860–869.

Gao K, Norell MA. 2000. Taxonomic composition and systematics of Late Cretaceous lizard assemblages from Ukhaa Tolgod and adjacent localities, Mongolian Gobi Desert. *Bull. Am. Mus. Nat. Hist.* 2000:1–118.

Gauthier JA, Kearney M, Maisano JA, Rieppel O, Behlke ADB. 2012. Assembling the Squamate Tree of Life: Perspectives from the Phenotype and the Fossil Record. *Bull. Peabody Mus. Nat. Hist.* 53:3–308.

Gheerbrant E. 2009. Paleocene emergence of elephant relatives and the rapid radiation of African ungulates. *Proc. Natl. Acad. Sci.* 106:10717–10721.

Gheerbrant E, Schmitt A, Kocsis L. 2018. Early African Fossils Elucidate the Origin of Embrithopod Mammals. *Curr. Biol.* 28:2167-2173.e2.

Ghezelayagh A, Harrington RC, Burress ED, Campbell MA, Buckner JC, Chakrabarty P, Glass JR, McCraney WT, Unmack PJ, Thacker CE, et al. 2022. Prolonged morphological expansion of spiny-rayed fishes following the end-Cretaceous. *Nat. Ecol. Evol.* 6:1211–1220.

Gil-Delgado A, Delclòs X, Sellés A, Galobart À, Oms O. 2023. The Early Cretaceous coastal lake Konservat-Lagerstätte of La Pedrera de Meià (Southern Pyrenees). *Geol. Acta* 21:1–XIII.

Giles S, Darras L, Clément G, Blieck A, Friedman M. 2015. An exceptionally preserved Late Devonian actinopterygian provides a new model for primitive cranial anatomy in ray-finned fishes. *Proc. R. Soc. B Biol. Sci.* 282:20151485.

bioRxiv preprint doi: https://doi.org/10.64898/2026.06.28.735080; this version posted June 29, 2026. The copyright holder for this preprint (which was not certified by peer review) is the author/funder, who has granted bioRxiv a license to display the preprint in perpetuity. It is made available under a CC-BY 4.0 International license.

Giles S, Friedman M, Brazeau MD. 2015. Osteichthyan-like cranial conditions in an Early Devonian stem gnathostome. *Nature* 520:82–85.

Giles S, Xu G-H, Near TJ, Friedman M. 2017. Early members of 'living fossil' lineage imply later origin of modern ray-finned fishes. *Nature* 549:265–268.

Goloboff PA, Catalano SA. 2016. TNT version 1.5, including a full implementation of phylogenetic morphometrics. *Cladistics* 32:221–238.

Gómez RO. 2016. A new pipid frog from the Upper Cretaceous of Patagonia and early evolution of crown-group Pipidae. *Cretac. Res.* 62:52–64.

Gottmann-Quesada A, Sander P. 2009. A redescription of the early archosauromorph *Protorosaurus speneri* MEYER, 1832, and its phylogenetic relationships. *Palaeontogr. A* 287:123–220.

Gradstein FM, Ogg JG, Schmitz M, Ogg G. 2021. The Geologic Time Scale 2020. Amsterdam, The Netherlands: Elsevier Science

Grande L. 2010. An Empirical Synthetic Pattern Study of Gars (lepisosteiformes) and Closely Related Species, Based Mostly on Skeletal Anatomy. the Resurrection of Holostei. *Copeia* 2010:iii–871.

Grande L, Jin F, Yabumoto Y, Bemis WE. 2002. Protopsephurus liui, a well-preserved primitive paddlefish (Acipenseriformes: Polyodontidae) from the Lower Cretaceous of China. *J. Vertebr. Paleontol.* 22:209–237.

GRANDE T, POYATO-ARIZA FJ. 1999. Phylogenetic relationships of fossil and Recent gonorynchiform fishes (Teleostei: Ostariophysi). *Zool. J. Linn. Soc.* 125:197–238.

Grande TC, Borden WC, Wilson MVH, Scarpitta L. 2018. Phylogenetic Relationships among Fishes in the Order Zeiformes Based on Molecular and Morphological Data. *Copeia* 106:20–48.

Griffiths EF, Ford DP, Benson RBJ, Evans SE. 2021. New information on the Jurassic lepidosauromorph *Marmoretta oxoniensis*.Ruta M, editor. *Pap. Palaeontol.* 7:2255–2278.

Hackett SJ, Kimball RT, Reddy S, Bowie RCK, Braun EL, Braun MJ, Chojnowski JL, Cox WA, Han K-L, Harshman J, et al. 2008. A Phylogenomic Study of Birds Reveals Their Evolutionary History. *Science* 320:1763–1768.

Han G, Mao F, Bi S, Wang Y, Meng J. 2017. A Jurassic gliding euharamiyidan mammal with an ear of five auditory bones. *Nature* 551:451–456.

bioRxiv preprint doi: https://doi.org/10.64898/2026.06.28.735080; this version posted June 29, 2026. The copyright holder for this preprint (which was not certified by peer review) is the author/funder, who has granted bioRxiv a license to display the preprint in perpetuity. It is made available under a CC-BY 4.0 International license.

Hand SJ, Maugoust J, Beck RMD, Orliac MJ. 2023. A 50-million-year-old, three-dimensionally preserved bat skull supports an early origin for modern echolocation. *Curr. Biol.* 33:4624-4640.e21.

Hao S, Han K, Meng L, Huang X, Cao W, Shi C, Zhang M, Wang Y, Liu Q, Zhang Y, et al. 2020. African Arowana Genome Provides Insights on Ancient Teleost Evolution. *iScience* 23:101662.

Hedges SB. 2012. Amniote phylogeny and the position of turtles. *BMC Biol.* 10:64.

Henrici AC. 1998. A New Pipoid Anuran from the Late Jurassic Morrison Formation at Dinosaur National Monument, Utah. *J. Vertebr. Paleontol.* 18:321–332.

Hernández-Guerrero C, Cantalice KM, González-Rodríguez KA, Bravo-Cuevas VM. 2021. The first record of a pterothrissin (Albuliformes, Albulidae) from the Muhi Quarry, mid-Cretaceous (Albian-Cenomanian) of Hidalgo, central Mexico. *J. South Am. Earth Sci.* 107:103032.

Hilton EJ, Grande L, Bemis WE. 2011. Skeletal Anatomy of the Shortnose Sturgeon, Acipenser brevirostrum Lesueur, 1818, and the Systematics of Sturgeons (Acipenseriformes, Acipenseridae). *Fieldiana Life Earth Sci.* 2011:1–168.

Hime PM, Lemmon AR, Lemmon ECM, Prendini E, Brown JM, Thomson RC, Kratovil JD, Noonan BP, Pyron RA, Peloso PLV, et al. 2020. Phylogenomics Reveals Ancient Gene Tree Discordance in the Amphibian Tree of Life. *Syst. Biol.* 70:49–66.

Hirayama R, Isaji S, Hibino T. 2013. Kappachelys okurai gen. et sp. nov., a New Stem Soft-Shelled Turtle from the Early Cretaceous of Japan. In: Brinkman DB, Holroyd PA, Gardner JD, editors. Morphology and Evolution of Turtles. Dordrecht: Springer Netherlands. p. 179–185. Available from: https://doi.org/10.1007/978-94-007-4309-0_12

Hoffmann S, Beck RMD, Wible JR, Rougier GW, Krause DW. 2020. Phylogenetic placement of Adalatherium hui (Mammalia, Gondwanatheria) from the Late Cretaceous of Madagascar: implications for allotherian relationships. *J. Vertebr. Paleontol.* 40:213–234.

Houde P, Dickson M, Camarena D. 2023. Basal Anseriformes from the Early Paleogene of North America and Europe. *Diversity* 15:233.

Houssaye A, Rage J-C, Torcida Fernández-Baldor F, Huerta P, Bardet N, Pereda Suberbiola X. 2013. A new varanoid squamate from the Early Cretaceous (Barremian–Aptian) of Burgos, Spain. *Cretac. Res.* 41:127–135.

Hughes LC, Ortí G, Huang Y, Sun Y, Baldwin CC, Thompson AW, Arcila D, Betancur-R R, Li C, Becker L, et al. 2018. Comprehensive phylogeny of ray-finned fishes (Actinopterygii)

bioRxiv preprint doi: https://doi.org/10.64898/2026.06.28.735080; this version posted June 29, 2026. The copyright holder for this preprint (which was not certified by peer review) is the author/funder, who has granted bioRxiv a license to display the preprint in perpetuity. It is made available under a CC-BY 4.0 International license.

based on transcriptomic and genomic data. *Proc. Natl. Acad. Sci. U. S. A.* 115:6249–6254.

Hughes LC, Ortí G, Saad H, Li C, White WT, Baldwin CC, Crandall KA, Arcila D, Betancur-R R. 2021. Exon probe sets and bioinformatics pipelines for all levels of fish phylogenomics. *Mol. Ecol. Resour.* 21:816–833.

Hurley IA, Mueller RL, Dunn KA, Schmidt EJ, Friedman M, Ho RK, Prince VE, Yang Z, Thomas MG, Coates MI. 2006. A new time-scale for ray-finned fish evolution. *Proc. R. Soc. B Biol. Sci.* 274:489–498.

Huttenlocker AK, Grossnickle DM, Kirkland JI, Schultz JA, Luo Z-X. 2018. Late-surviving stem mammal links the lowermost Cretaceous of North America and Gondwana. *Nature* 558:108–112.

Irisarri I, Baurain D, Brinkmann H, Delsuc F, Sire J-Y, Kupfer A, Petersen J, Jarek M, Meyer A, Vences M, et al. 2017. Phylotranscriptomic consolidation of the jawed vertebrate timetree. *Nat. Ecol. Evol.* 1:1370–1378.

Irisarri I, Meyer A. 2016. The Identification of the Closest Living Relative(s) of Tetrapods: Phylogenomic Lessons for Resolving Short Ancient Internodes. *Syst. Biol.* 65:1057–1075.

Iwabe N, Hara Y, Kumazawa Y, Shibamoto K, Saito Y, Miyata T, Katoh K. 2005. Sister Group Relationship of Turtles to the Bird-Crocodilian Clade Revealed by Nuclear DNA–Coded Proteins. *Mol. Biol. Evol.* 22:810–813.

Jäger KRK, Luo Z-X, Martin T. 2020. Postcranial Skeleton of Henkelotherium guimarotae (Cladotheria, Mammalia) and Locomotor Adaptation. *J. Mamm. Evol.* 27:349–372.

Jarvis ED, Mirarab S, Aberer AJ, Li B, Houde P, Li C, Ho SYW, Faircloth BC, Nabholz B, Howard JT, et al. 2014. Whole-genome analyses resolve early branches in the tree of life of modern birds. *Science* 346:1320–1331.

Jones ME, Anderson CL, Hipsley CA, Müller J, Evans SE, Schoch RR. 2013. Integration of molecules and new fossils supports a Triassic origin for Lepidosauria (lizards, snakes, and tuatara). *BMC Evol. Biol.* 13:208.

Jones MEH, Benson RBJ, Skutschas P, Hill L, Panciroli E, Schmitt AD, Walsh SA, Evans SE. 2022. Middle Jurassic fossils document an early stage in salamander evolution. *Proc. Natl. Acad. Sci.* 119:e2114100119.

Jones MF, Beard KC, Simmons NB. 2024. Phylogeny and systematics of early Paleogene bats. *J. Mamm. Evol.* 31:18.

bioRxiv preprint doi: https://doi.org/10.64898/2026.06.28.735080; this version posted June 29, 2026. The copyright holder for this preprint (which was not certified by peer review) is the author/funder, who has granted bioRxiv a license to display the preprint in perpetuity. It is made available under a CC-BY 4.0 International license.

Joyce WG, Parham JF, Lyson TR, Warnock RCM, Donoghue PCJ. 2013. A Divergence Dating Analysis of Turtles Using Fossil Calibrations: An Example of Best Practices. *J. Paleontol.* 87:612–634.

Kemp A, Cavin L, Guinot G. 2017. Evolutionary history of lungfishes with a new phylogeny of post-Devonian genera. *Palaeogeogr. Palaeoclimatol. Palaeoecol.* 471:209–219.

Keqin G, Lianhai H. 1995. Iguanians From the Upper Cretaceous Djadochta Formation, Gobi Desert, China. *J. Vertebr. Paleontol.* 15:57–78.

Kimball RT, Oliveros CH, Wang N, White ND, Barker FK, Field DJ, Ksepka DT, Chesser RT, Moyle RG, Braun MJ, et al. 2019. A Phylogenomic Supertree of Birds. *Diversity* 11:109.

King B, Qiao T, Lee MSY, Zhu M, Long JA. 2017. Bayesian Morphological Clock Methods Resurrect Placoderm Monophyly and Reveal Rapid Early Evolution in Jawed Vertebrates. *Syst. Biol.* 66:499–516.

Kligman BT, Gee BM, Marsh AD, Nesbitt SJ, Smith ME, Parker WG, Stocker MR. 2023. Triassic stem caecilian supports dissorophoid origin of living amphibians. *Nature* 614:102–107.

Klug C, Coates M, Frey L, Greif M, Jobbins M, Pohle A, Lagnaoui A, Haouz WB, Ginter M. 2023. Broad snouted cladoselachian with sensory specialization at the base of modern chondrichthyans. *Swiss J. Palaeontol.* 142:2.

Korth WW. 1994. The Tertiary record of rodents in North America. Springer Science & Business Media Available from: https://books.google.com/books?hl=en&lr=&id=F4yxJ3M06TgC&oi=fnd&pg=PA3&ots=Xb6AGlvLbM&sig=zjbRD4YVyZvV_C9JhP5doS9NtZI

Kowallis BJ, Christiansen EH, Deino AL. 1991. Age of the Brushy Basin Member of the Morrison Formation, Colorado Plateau, western USA. *Cretac. Res.* 12:483–493.

Kramarz A, MacPhee R. 2022. Did some extinct South American native ungulates arise from an afrothere ancestor? A critical appraisal of Avilla and Mothé's (2021) Sudamericungulata – Panameridiungulata hypothesis. *J. Mamm. Evol.* 30:1–11.

Krause DW, Hoffmann S, Hu Y, Wible JR, Rougier GW, Kirk EC, Groenke JR, Rogers RR, Rossie JB, Schultz JA, et al. 2020. Skeleton of a Cretaceous mammal from Madagascar reflects long-term insularity. *Nature* 581:421–427.

Krebs B. 1991. Das Skelett von Henkelotherium guimarotae gen. et sp. nov.(Eupantotheria, Mammalia) aus dem Oberen Jura von Portugal. Selbstverlag Fachbereich Geowissenschaften, FU Berlin Available from: https://e-docs.geo-leo.de/entities/publication/a0f63f76-85c0-4364-a06b-090b3a046f69

bioRxiv preprint doi: https://doi.org/10.64898/2026.06.28.735080; this version posted June 29, 2026. The copyright holder for this preprint (which was not certified by peer review) is the author/funder, who has granted bioRxiv a license to display the preprint in perpetuity. It is made available under a CC-BY 4.0 International license.

Kroth M, Trabucho-Alexandre JP, Pimenta MP, Vis G-J, Boever ED. 2024. Facies characterisation and stratigraphy of the upper Maastrichtian to lower Danian Maastricht Formation, South Limburg, the Netherlands. *Neth. J. Geosci.* 103:e13.

Ksepka DT, Bertelli S, Giannini NP. 2006. The phylogeny of the living and fossil Sphenisciformes (penguins). *Cladistics* 22:412–441.

Ksepka DT, Field DJ, Heath TA, Pett W, Thomas DB, Giovanardi S, Tennyson AJD. 2023. Largest-known fossil penguin provides insight into the early evolution of sphenisciform body size and flipper anatomy. *J. Paleontol.* 97:434–453.

Ksepka DT, Grande L, Mayr G. 2019. Oldest Finch-Beaked Birds Reveal Parallel Ecological Radiations in the Earliest Evolution of Passerines. *Curr. Biol.* 29:657-663.e1.

Ksepka DT, Stidham TA, Williamson TE. 2017. Early Paleocene landbird supports rapid phylogenetic and morphological diversification of crown birds after the K–Pg mass extinction. *Proc. Natl. Acad. Sci.* 114:8047–8052.

Kuhl H, Frankl-Vilches C, Bakker A, Mayr G, Nikolaus G, Boerno ST, Klages S, Timmermann B, Gahr M. 2021. An Unbiased Molecular Approach Using 3′-UTRs Resolves the Avian Family-Level Tree of Life. *Mol. Biol. Evol.* 38:108–127.

Lambert O, Martínez-Cáceres M, Bianucci G, Celma CD, Salas-Gismondi R, Steurbaut E, Urbina M, Muizon C de. 2017. Earliest Mysticete from the Late Eocene of Peru Sheds New Light on the Origin of Baleen Whales. *Curr. Biol.* 27:1535-1541.e2.

Lee MSY. 1993. The Origin of the Turtle Body Plan: Bridging a Famous Morphological Gap. *Science* 261:1716–1720.

Lee MSY, Yates AM. 2018. Tip-dating and homoplasy: reconciling the shallow molecular divergences of modern gharials with their long fossil record. *Proc. R. Soc. B Biol. Sci.* 285:20181071.

Liu G, Pan Q, Dai Y, Wang X, Li M, Zhu P, Zhou X. 2024. Phylogenomics of Afrotherian mammals and improved resolution of extant Paenungulata. *Mol. Phylogenet. Evol.* 195:108047.

Liu J. 2021. Redescription of ‘Amyzon’ brevipinne and remarks on North American Eocene catostomids (Cypriniformes: Catostomidae). *J. Syst. Palaeontol.* 19:677–689.

Longrich NR, Bhullar B-AS, Gauthier JA. 2012. Mass extinction of lizards and snakes at the Cretaceous–Paleogene boundary. *Proc. Natl. Acad. Sci.* 109:21396–21401.

Longrich NR, Vinther J, Pyron RA, Pisani D, Gauthier JA. 2015. Biogeography of worm lizards (Amphisbaenia) driven by end-Cretaceous mass extinction. *Proc. R. Soc. B Biol. Sci.* [Internet]. Available from: https://royalsocietypublishing.org/doi/10.1098/rspb.2014.3034

bioRxiv preprint doi: https://doi.org/10.64898/2026.06.28.735080; this version posted June 29, 2026. The copyright holder for this preprint (which was not certified by peer review) is the author/funder, who has granted bioRxiv a license to display the preprint in perpetuity. It is made available under a CC-BY 4.0 International license.

López-Arbarello A. 2012. Phylogenetic Interrelationships of Ginglymodian Fishes (Actinopterygii: Neopterygii). *PLOS ONE* 7:e39370.

López-Arbarello A, Sferco E. 2018. Neopterygian phylogeny: the merger assay. *R. Soc. Open Sci.* 5:172337.

López-Conde OA, Sterli J, Alvarado-Ortega J, Chavarría-Arellano ML. 2017. A new platychelyid turtle (Pan-Pleurodira) from the Late Jurassic (Kimmeridgian) of Oaxaca, Mexico. *Pap. Palaeontol.* 3:161–174.

L-Recinos M, Cantalice ,Kleyton M., Caballero-Viñas ,Carmen, and Alvarado-Ortega J. 2023. A new Mesozoic teleost of the subfamily Albulinae (Albuliformes: Albulidae) highlights the proto-Gulf of Mexico in the early diversification of extant bonefishes. *J. Syst. Palaeontol.* 21:2223797.

Lu J, Giles S, Friedman M, den Blaauwen JL, Zhu M. 2016. The Oldest Actinopterygian Highlights the Cryptic Early History of the Hyperdiverse Ray-Finned Fishes. *Curr. Biol.* 26:1602–1608.

Lu J, Giles S, Friedman M, Zhu M. 2017. A new stem sarcopterygian illuminates patterns of character evolution in early bony fishes. *Nat. Commun.* 8:1932.

Lu J, Zhu M, Ahlberg PE, Qiao T, Zhu Y, Zhao W, Jia L. 2016. A Devonian predatory fish provides insights into the early evolution of modern sarcopterygians. *Sci. Adv.* 2:e1600154.

Luo Z-X, Gatesy SM, Jenkins FA, Amaral WW, Shubin NH. 2015. Mandibular and dental characteristics of Late Triassic mammaliaform Haramiyavia and their ramifications for basal mammal evolution. *Proc. Natl. Acad. Sci. U. S. A.* 112:E7101–E7109.

Luo Z-X, Martin T. 2023. Mandibular and dental characteristics of the Late Jurassic mammal Henkelotherium guimarotae (Paurodontidae, Dryolestida). *PalZ* 97:569–619.

Luo Z-X, Meng Q-J, Ji Q, Liu D, Zhang Y-G, Neander AI. 2015. Evolutionary development in basal mammaliaforms as revealed by a docodontan. *Science* 347:760–764.

Maidment SCR, Balikova D, Muxworthy AR. 2017. Magnetostratigraphy of the Upper Jurassic Morrison Formation at Dinosaur National Monument, Utah, and Prospects for Using Magnetostratigraphy as a Correlative Tool in the Morrison Formation. In: Zeigler KE, Parker WG, editors. Terrestrial Depositional Systems. Elsevier. p. 279–302. Available from: https://www.sciencedirect.com/science/article/pii/B9780128032435000078

Mallik R, Wcisel DJ, Near TJ, Yoder JA, Dornburg A. 2025. Investigating the Impact of Whole-Genome Duplication on Transposable Element Evolution in Teleost Fishes. *Genome Biol. Evol.* 17:evae272.

bioRxiv preprint doi: https://doi.org/10.64898/2026.06.28.735080; this version posted June 29, 2026. The copyright holder for this preprint (which was not certified by peer review) is the author/funder, who has granted bioRxiv a license to display the preprint in perpetuity. It is made available under a CC-BY 4.0 International license.

Marivaux L, Vianey-Liaud M, Jaeger J-J. 2004. High-level phylogeny of early Tertiary rodents: dental evidence. *Zool. J. Linn. Soc.* 142:105–134.

Martínez RN, Simões TR, Sobral G, Apesteguía S. 2021. A Triassic stem lepidosaur illuminates the origin of lizard-like reptiles. *Nature* 597:235–238.

Marugán-Lobón J, Martín-Abad H, Buscalioni ÁD. 2023. The Las Hoyas Lagerstätte: a palaeontological view of an Early Cretaceous wetland. *J. Geol. Soc.* 180:jgs2022-079.

Mateus O, Puértolas-Pascual E, Callapez PM. 2019. A new eusuchian crocodylomorph from the Cenomanian (Late Cretaceous) of Portugal reveals novel implications on the origin of Crocodylia. *Zool. J. Linn. Soc.* 186:501–528.

Mayr G. 2015. The middle Eocene European "ratite" Palaeotis (Aves, Palaeognathae) restudied once more. *Paläontol. Z.* 89:503–514.

Mayr G, De Pietri VL, Paul Scofield R. 2017. A new fossil from the mid-Paleocene of New Zealand reveals an unexpected diversity of world's oldest penguins. *Sci. Nat.* 104:9.

Mayr G, Pietri VLD, Love L, Mannering AA, Scofield RP. 2017. A well-preserved new mid-paleocene penguin (Aves, Sphenisciformes) from the Waipara Greensand in New Zealand. *J. Vertebr. Paleontol.* [Internet]. Available from: https://www.tandfonline.com/doi/full/10.1080/02724634.2017.1398169

Mayr G, Zelenkov N. 2021. Extinct crane-like birds (Eogruidae and Ergilornithidae) from the Cenozoic of Central Asia are indeed ostrich precursors. *Ornithology* 138:ukab048.

McInerney PL, Blokland JC, Worthy TH. 2024. Skull morphology of the enigmatic Genyornis newtoni Stirling and Zeitz, 1896 (Aves, Dromornithidae), with implications for functional morphology, ecology, and evolution in the context of Galloanserae. *Hist. Biol.* 36:1093–1165.

Melo BF, Sidlauskas BL, Near TJ, Roxo FF, Ghezelayagh A, Ochoa LE, Stiassny MLJ, Arroyave J, Chang J, Faircloth BC, et al. 2022. Accelerated Diversification Explains the Exceptional Species Richness of Tropical Characoid Fishes. *Syst. Biol.* 71:78–92.

Melstrom KM, Irmis RB. 2019. Repeated Evolution of Herbivorous Crocodyliforms during the Age of Dinosaurs. *Curr. Biol.* 29:2389-2395.e3.

Meredith RW, Janečka JE, Gatesy J, Ryder OA, Fisher CA, Teeling EC, Goodbla A, Eizirik E, Simão TLL, Stadler T, et al. 2011. Impacts of the Cretaceous Terrestrial Revolution and KPg extinction on mammal diversification. *Science* 334:521–524.

Mertz DF, Renne PR. 2005. A numerical age for the Messel fossil deposit (UNESCO World Heritage Site derived from^ 4^ 0Ar/^ 3^ 9Ar dating on a basaltic rock fragment. *Cour.-FORSCHUNGSINSTITUT SENCKENBERG* 255:67.

bioRxiv preprint doi: https://doi.org/10.64898/2026.06.28.735080; this version posted June 29, 2026. The copyright holder for this preprint (which was not certified by peer review) is the author/funder, who has granted bioRxiv a license to display the preprint in perpetuity. It is made available under a CC-BY 4.0 International license.

Meyer A, Schloissnig S, Franchini P, Du K, Woltering JM, Irisarri I, Wong WY, Nowoshilow S, Kneitz S, Kawaguchi A, et al. 2021. Giant lungfish genome elucidates the conquest of land by vertebrates. *Nature* 590:284–289.

Meyer D, Brownstein CD, Jenkins KM, Gauthier JA. 2023. A Morrison stem gekkotan reveals gecko evolution and Jurassic biogeography. *Proc. R. Soc. B* [Internet]. Available from: https://royalsocietypublishing.org/doi/10.1098/rspb.2023.2284

Milàn J, Lucas S, Lockley M, Spielmann J, Schwimmer D. 2010. BITE MARKS OF THE GIANT CROCODYLIAN DEINOSUCHUS ON LATE CRETACEOUS (CAMPANIAN) BONES. 51.

Miller EC, Martinez CM, Friedman ST, Wainwright PC, Price SA, Tornabene L. 2022. Alternating regimes of shallow and deep-sea diversification explain a species-richness paradox in marine fishes. *Proc. Natl. Acad. Sci.* 119:e2123544119.

Miller KG, Sugarman PJ, Browning JV, Kominz MA, Olsson RK, Feigenson MD, Hernandez JC. 2004. Upper Cretaceous sequences and sea-level history, New Jersey Coastal Plain. *Geol. Soc. Am. Bull.* 116:368–393.

Mirarab S, Rivas-González I, Feng S, Stiller J, Fang Q, Mai U, Hickey G, Chen G, Brajuka N, Fedrigo O, et al. 2024. A region of suppressed recombination misleads neoavian phylogenomics. *Proc. Natl. Acad. Sci.* 121:e2319506121.

Mohler BF, McDonald AT, Wolfe DG. 2021. First remains of the enormous alligatoroid Deinosuchus from the Upper Cretaceous Menefee Formation, New Mexico. *PeerJ* 9:e11302.

Monsch KA, Bannikov AF. 2011. New taxonomic synopses and revision of the scombroid fishes (Scombroidei, Perciformes), including billfishes, from the Cenozoic of territories of the former USSR. *Earth Environ. Sci. Trans. R. Soc. Edinb.* 102:253–300.

Musilova Z, Cortesi F, Matschiner M, Davies WIL, Patel JS, Stieb SM, de Busserolles F, Malmstrøm M, Tørresen OK, Brown CJ, et al. 2019. Vision using multiple distinct rod opsins in deep-sea fishes. *Science* 364:588–592.

Musser G, Clarke JA. 2024. A new Paleogene fossil and a new dataset for waterfowl (Aves: Anseriformes) clarify phylogeny, ecological evolution, and avian evolution at the K-Pg Boundary. *PLOS ONE* 19:e0278737.

Near TJ, Dornburg A, Friedman M. 2014. Phylogenetic relationships and timing of diversification in gonorynchiform fishes inferred using nuclear gene DNA sequences (Teleostei: Ostariophysi). *Mol. Phylogenet. Evol.* 80:297–307.

Near TJ, Eytan RI, Dornburg A, Kuhn KL, Moore JA, Davis MP, Wainwright PC, Friedman M, Smith WL. 2012. Resolution of ray-finned fish phylogeny and timing of diversification. *Proc. Natl. Acad. Sci.* 109:13698–13703.

bioRxiv preprint doi: https://doi.org/10.64898/2026.06.28.735080; this version posted June 29, 2026. The copyright holder for this preprint (which was not certified by peer review) is the author/funder, who has granted bioRxiv a license to display the preprint in perpetuity. It is made available under a CC-BY 4.0 International license.

Near TJ, Thacker CE. 2024. Phylogenetic classification of living and fossil ray-finned fishes (Actinopterygii). *Bull. Peabody Mus. Nat. Hist.* 65:3–302.

Nesbitt SJ, Butler RJ, Ezcurra MD, Barrett PM, Stocker MR, Angielczyk KD, Smith RMH, Sidor CA, Niedźwiedzki G, Sennikov AG, et al. 2017. The earliest bird-line archosaurs and the assembly of the dinosaur body plan. *Nature* 544:484–487.

Nesbitt SJ, Clarke JA. 2016. The Anatomy and Taxonomy of the Exquisitely Preserved Green River Formation (Early Eocene) Lithornithids (Aves) and the Relationships of Lithornithidae. *Bull. Am. Mus. Nat. Hist.* 2016:1–91.

Nesbitt SJ, Patellos E, Kammerer CF, Ranivoharimanana L, Wyss AR, Flynn JJ. 2023. The earliest-diverging avemetatarsalian: a new osteoderm-bearing taxon from the Triassic (?Earliest Late Triassic) of Madagascar and the composition of avemetatarsalian assemblages prior to the radiation of dinosaurs. *Zool. J. Linn. Soc.* 199:327–353.

Ni X, Gebo DL, Dagosto M, Meng J, Tafforeau P, Flynn JJ, Beard KC. 2013. The oldest known primate skeleton and early haplorhine evolution. *Nature* 498:60–64.

Ogg JG, Ogg GM, Gradstein FM. 2016. 14 - Paleogene. In: Ogg JG, Ogg GM, Gradstein FM, editors. A Concise Geologic Time Scale. Elsevier. p. 187–201. Available from: https://www.sciencedirect.com/science/article/pii/B9780444594679000145

O'Leary MA, Bloch JI, Flynn JJ, Gaudin TJ, Giallombardo A, Giannini NP, Goldberg SL, Kraatz BP, Luo Z-X, Meng J, et al. 2013. The placental mammal ancestor and the post-K-Pg radiation of placentals. *Science* 339:662–667.

Olsen PE. 1984. The skull and pectoral girdle of the parasemionotid fish Watsonulus eugnathoides from the Early Triassic Sakamena Group of Madagascar, with comments on the relationships of the holostean fishes. *J. Vertebr. Paleontol.* 4:481–499.

Onary S, Hsiou AS, Lee MSY, Palci A. 2021. Redescription, taxonomy and phylogenetic relationships of Boavus Marsh, 1871 (Serpentes: Booidea) from the early–middle Eocene of the USA. *J. Syst. Palaeontol.* 19:1601–1622.

Palci A, Onary S, Lee MSY, Smith KT, Wings O, Rabi M, Georgalis GL. 2024. A new booid snake from the Eocene (Lutetian) Konservat-Lagerstätte of Geiseltal, Germany, and a new phylogenetic analysis of Booidea. *Zool. J. Linn. Soc.* 202:zlad179.

Parey E, Louis A, Montfort J, Bouchez O, Roques C, Iampietro C, Lluch J, Castinel A, Donnadieu C, Desvignes T, et al. 2023. Genome structures resolve the early diversification of teleost fishes. *Science* 379:572–575.

Paterson RS, Rybczynski N, Kohno N, Maddin HC. 2020. A Total Evidence Phylogenetic Analysis of Pinniped Phylogeny and the Possibility of Parallel Evolution Within a Monophyletic Framework. *Front. Ecol. Evol.* [Internet] 7. Available from:

bioRxiv preprint doi: https://doi.org/10.64898/2026.06.28.735080; this version posted June 29, 2026. The copyright holder for this preprint (which was not certified by peer review) is the author/funder, who has granted bioRxiv a license to display the preprint in perpetuity. It is made available under a CC-BY 4.0 International license.

https://www.frontiersin.org/journals/ecology-and-evolution/articles/10.3389/fevo.2019.00457/full

Phillips MJ, Fruciano C. 2018. The soft explosive model of placental mammal evolution. *BMC Evol. Biol.* 18:104.

Poust A, Boessenecker R. 2018. Expanding the geographic and geochronologic range of early pinnipeds: new specimens of Enaliarctos from Northern California and Oregon. *Acta Palaeontol. Pol.* [Internet] 63. Available from: http://www.app.pan.pl/article/item/app003992017.html

Poyato-Ariza FJ. 1996a. The phylogenetic relationships of Rubiesichthys gregalis and Gordichthys conquensis (Ostariophysi, Chanidae), from the Early Cretaceous of Spain. *Mesoz. Fishes—Systematics Paleoecol. Verl. Dr Fredrich Pfeil Munich Ger.*:329–348.

Poyato-Ariza FJ. 1996b. A revision of Rubiesichthys gregalis WENZ 1984 (Ostariophysi, Gonorynchiformes), from the Early Cretaceous of Spain. *Syst Paleoecol* 1984:329–348.

Prokofiev AM. 2002. A remarkable new genus of Carangidae fron1 the Upper Paleocene of Turkmenistan (Osteichthyes: Perciformes). Available from: https://www.zin.ru/Journals/zsr/content/2002/zr_2002_11_1_Prokofiev_2.pdf

Prothero D, Bitboul C, Moore G, Niem A, Section AA of PGP, Section GS of AC. 2001. Magnetic stratigraphy of the Pacific Coast Cenozoic : a symposium volume based on Proceedings of the 1997 Pacific Section AAPG-SEPM Meeting, Bakersfield, California, and the 2001 Pacific Section AAPG-SEPM-Cordilleran Section GSA Meeting. Fullerton, CA: Pacific Section SEPM

Prum RO, Berv JS, Dornburg A, Field DJ, Townsend JP, Lemmon EM, Lemmon AR. 2015. A comprehensive phylogeny of birds (Aves) using targeted next-generation DNA sequencing. *Nature* 526:569–573.

Pyron RA, Burbrink FT, Wiens JJ. 2013. A phylogeny and revised classification of Squamata, including 4161 species of lizards and snakes. *BMC Evol. Biol.* 13:93.

Qiao T, King B, Long JA, Ahlberg PE, Zhu M. 2016. Early Gnathostome Phylogeny Revisited: Multiple Method Consensus. *PLoS ONE* 11:e0163157.

Qu Q, Zhu M, Wang W. 2013. Scales and Dermal Skeletal Histology of an Early Bony Fish Psarolepis romeri and Their Bearing on the Evolution of Rhombic Scales and Hard Tissues. *PLOS ONE* 8:e61485.

Reddy S, Kimball RT, Pandey A, Hosner PA, Braun MJ, Hackett SJ, Han K-L, Harshman J, Huddleston CJ, Kingston S, et al. 2017. Why Do Phylogenomic Data Sets Yield Conflicting Trees? Data Type Influences the Avian Tree of Life more than Taxon Sampling. *Syst. Biol.* 66:857–879.

bioRxiv preprint doi: https://doi.org/10.64898/2026.06.28.735080; this version posted June 29, 2026. The copyright holder for this preprint (which was not certified by peer review) is the author/funder, who has granted bioRxiv a license to display the preprint in perpetuity. It is made available under a CC-BY 4.0 International license.

dos Reis M, Donoghue PCJ, Yang Z. 2014. Neither phylogenomic nor palaeontological data support a Palaeogene origin of placental mammals. *Biol. Lett.* 10:20131003.

dos Reis M, Inoue J, Hasegawa M, Asher RJ, Donoghue PCJ, Yang Z. 2012. Phylogenomic datasets provide both precision and accuracy in estimating the timescale of placental mammal phylogeny. *Proc. Biol. Sci.* 279:3491–3500.

Renesto S, Bernardi M. 2014. Redescription and phylogenetic relationships of Megachirella wachtleri Renesto et Posenato, 2003 (Reptilia, Diapsida). *Paläontol. Z.* 88:197–210.

Ribeiro AC, Poyato-Ariza FJ, Bockmann FA, Carvalho MR de. 2018. Phylogenetic relationships of Chanidae (Teleostei: Gonorynchiformes) as impacted by Dastilbe moraesi , from the Sanfranciscana basin, Early Cretaceous of Brazil. *Neotropical Ichthyol.* 16:e180059.

Rietbergen TB, van den Hoek Ostende LW, Aase A, Jones MF, Medeiros ED, Simmons NB. 2023. The oldest known bat skeletons and their implications for Eocene chiropteran diversification. *PLOS ONE* 18:e0283505.

Rio JP, Mannion PD. 2021. Phylogenetic analysis of a new morphological dataset elucidates the evolutionary history of Crocodylia and resolves the long-standing gharial problem. *PeerJ* 9:e12094.

Russell DE. 1987. The Paleogene of Asia: mammals and stratigraphy. *Mém. Muséum Natl. Hist. Nat. Sci. Terre* 52:1–488.

Santaquiteria A, Siqueira AC, Duarte-Ribeiro E, Carnevale G, White WT, Pogonoski JJ, Baldwin CC, Ortí G, Arcila D, Ricardo B-R. 2021. Phylogenomics and Historical Biogeography of Seahorses, Dragonets, Goatfishes, and Allies (Teleostei: Syngnatharia): Assessing Factors Driving Uncertainty in Biogeographic Inferences. *Syst. Biol.* 70:1145–1162.

SANTINI F, TYLER JC. 2003. A phylogeny of the families of fossil and extant tetraodontiform fishes (Acanthomorpha, Tetraodontiformes), Upper Cretaceous to Recent. *Zool. J. Linn. Soc.* 139:565–617.

Scarpetta SG. 2024. A Palaeogene stem crotaphytid (Aciprion formosum) and the phylogenetic affinities of early fossil pleurodontan iguanians. *R. Soc. Open Sci.* [Internet]. Available from: https://royalsocietypublishing.org/doi/10.1098/rsos.221139

Schartl M, Woltering JM, Irisarri I, Du K, Kneitz S, Pippel M, Brown T, Franchini P, Li J, Li M, et al. 2024. The genomes of all lungfish inform on genome expansion and tetrapod evolution. *Nature*:1–8.

Schoch RR, Sues H-D. 2018. A new lepidosauromorph reptile from the Middle Triassic (Ladinian) of Germany and its phylogenetic relationships. *J. Vertebr. Paleontol.* 38:e1444619.

bioRxiv preprint doi: https://doi.org/10.64898/2026.06.28.735080; this version posted June 29, 2026. The copyright holder for this preprint (which was not certified by peer review) is the author/funder, who has granted bioRxiv a license to display the preprint in perpetuity. It is made available under a CC-BY 4.0 International license.

Schoch RR, Werneburg R, Voigt S. 2020. A Triassic stem-salamander from Kyrgyzstan and the origin of salamanders. *Proc. Natl. Acad. Sci.* 117:11584–11588.

Schwimmer DR. 2002. King of the crocodylians: the paleobiology of Deinosuchus. Indiana University Press Available from: https://books.google.com/books?hl=en&lr=&id=0OsPJnC4CCwC&oi=fnd&pg=PR5&dq=King+of+the+Crocodylians:+The+Paleobiology+of+Deinosuchus+(Life+of+the+Past)&ots=8MNNMJwHvk&sig=pvFH3mGhJyykN1opYJWPTcqYfqY

Seiffert ER. 2007. A new estimate of afrotherian phylogeny based on simultaneous analysis of genomic, morphological, and fossil evidence. *BMC Evol. Biol.* 7:224.

Seiffert ER, Heritage S, de Vries D, Sallam HM, Vitek NS, Aoron E, Princehouse P. 2025. Oldest record of a crown anomaluroid rodent from sub-Saharan Africa: a new genus and species from the early Oligocene Topernawi Formation of northern Kenya. *Hist. Biol.* 37:1568–1578.

Shan Y, Gras R. 2011. 43 genes support the lungfish-coelacanth grouping related to the closest living relative of tetrapods with the Bayesian method under the coalescence model. *BMC Res. Notes* 4:49.

Simmons NB, Geisler JH. 1998. Phylogenetic Relationships of Icaronycteris, Archaeonycteris, Hassianycteris, and Palaeochiropteryx to Extant Bat Lineages, with Comments on the Evolution of Echolocation and Foraging Strategies in Microchiroptera. American Museum of Natural History

Simões TR, Caldwell MW, Pierce SE. 2020. Sphenodontian phylogeny and the impact of model choice in Bayesian morphological clock estimates of divergence times and evolutionary rates. *BMC Biol.* 18:191.

Simões TR, Caldwell MW, Tałanda M, Bernardi M, Palci A, Vernygora O, Bernardini F, Mancini L, Nydam RL. 2018. The origin of squamates revealed by a Middle Triassic lizard from the Italian Alps. *Nature* 557:706–709.

Simões TR, Kammerer CF, Caldwell MW, Pierce SE. 2022. Successive climate crises in the deep past drove the early evolution and radiation of reptiles. *Sci. Adv.* 8:eabq1898.

Singhal S, Colston TJ, Grundler MR, Smith SA, Costa GC, Colli GR, Moritz C, Pyron RA, Rabosky DL. 2021. Congruence and Conflict in the Higher-Level Phylogenetics of Squamate Reptiles: An Expanded Phylogenomic Perspective. *Syst. Biol.* 70:542–557.

Siu-Ting K, Torres-Sánchez M, San Mauro D, Wilcockson D, Wilkinson M, Pisani D, O’Connell MJ, Creevey CJ. 2019. Inadvertent Paralog Inclusion Drives Artifactual Topologies and Timetree Estimates in Phylogenomics. *Mol. Biol. Evol.* 36:1344–1356.

bioRxiv preprint doi: https://doi.org/10.64898/2026.06.28.735080; this version posted June 29, 2026. The copyright holder for this preprint (which was not certified by peer review) is the author/funder, who has granted bioRxiv a license to display the preprint in perpetuity. It is made available under a CC-BY 4.0 International license.

Slack KE, Jones CM, Ando T, Harrison GL (Abby), Fordyce RE, Arnason U, Penny D. 2006. Early Penguin Fossils, Plus Mitochondrial Genomes, Calibrate Avian Evolution. *Mol. Biol. Evol.* 23:1144–1155.

Slater GJ. 2015. Iterative adaptive radiations of fossil canids show no evidence for diversity-dependent trait evolution. *Proc. Natl. Acad. Sci.* 112:4897–4902.

Smith ME, Carroll AR, Singer BS. 2008. Synoptic reconstruction of a major ancient lake system: Eocene Green River Formation, western United States. *GSA Bull.* 120:54–84.

Sobral G, Simões TR, Schoch RR. 2020. A tiny new Middle Triassic stem-lepidosauromorph from Germany: implications for the early evolution of lepidosauromorphs and the Vellberg fauna. *Sci. Rep.* 10:2273.

Spiekman SNF, Fraser NC, Scheyer TM. 2021. A new phylogenetic hypothesis of Tanystropheidae (Diapsida, Archosauromorpha) and other "protorosaurs", and its implications for the early evolution of stem archosaurs. *PeerJ* 9:e11143.

Springer MS, Murphy WJ, Eizirik E, O'Brien SJ. 2003. Placental mammal diversification and the Cretaceous–Tertiary boundary. *Proc. Natl. Acad. Sci.* 100:1056–1061.

Stiller J, Feng S, Chowdhury A-A, Rivas-González I, Duchêne DA, Fang Q, Deng Y, Kozlov A, Stamatakis A, Claramunt S, et al. 2024. Complexity of avian evolution revealed by family-level genomes. *Nature*:1–3.

Straube N, Li C, Mertzen M, Yuan H, Moritz T. 2018. A phylogenomic approach to reconstruct interrelationships of main clupeocephalan lineages with a critical discussion of morphological apomorphies. *BMC Evol. Biol.* 18:158.

Streicher JW, Wiens JJ. 2017. Phylogenomic analyses of more than 4000 nuclear loci resolve the origin of snakes among lizard families. *Biol. Lett.* 13:20170393.

Takezaki N. 2018. Global Rate Variation in Bony Vertebrates. *Genome Biol. Evol.* 10:1803–1815.

Takezaki N. 2021. Resolving the Early Divergence Pattern of Teleost Fish Using Genome-Scale Data. *Genome Biol. Evol.* 13:evab052.

Takezaki N, Nishihara H. 2017. Support for Lungfish as the Closest Relative of Tetrapods by Using Slowly Evolving Ray-Finned Fish as the Outgroup. *Genome Biol. Evol.* 9:93–101.

Tałanda M, Fernandez V, Panciroli E, Evans SE, Benson RJ. 2022. Synchrotron tomography of a stem lizard elucidates early squamate anatomy. *Nature* 611:99–104.

Tambussi CP, Degrange FJ, De Mendoza RS, Sferco E, Santillana S. 2019. A stem anseriform from the early Palaeocene of Antarctica provides new key evidence in the early evolution of waterfowl. *Zool. J. Linn. Soc.* 186:673–700.

bioRxiv preprint doi: https://doi.org/10.64898/2026.06.28.735080; this version posted June 29, 2026. The copyright holder for this preprint (which was not certified by peer review) is the author/funder, who has granted bioRxiv a license to display the preprint in perpetuity. It is made available under a CC-BY 4.0 International license.

Tarver JE, dos Reis M, Mirarab S, Moran RJ, Parker S, O'Reilly JE, King BL, O'Connell MJ, Asher RJ, Warnow T, et al. 2016. The Interrelationships of Placental Mammals and the Limits of Phylogenetic Inference. *Genome Biol. Evol.* 8:330–344.

Thomas DB, Tennyson AJD, Marx FG, Ksepka DT. 2023. Pliocene fossils support a New Zealand origin for the smallest extant penguins. *J. Paleontol.* 97:711–721.

Thomas DB, Tennyson AJD, Scofield RP, Heath TA, Pett W, Ksepka DT. 2020. Ancient crested penguin constrains timing of recruitment into seabird hotspot. *Proc. R. Soc. B Biol. Sci.* 287:20201497.

Thompson AW, Hawkins MB, Parey E, Wcisel DJ, Ota T, Kawasaki K, Funk E, Losilla M, Fitch OE, Pan Q, et al. 2021. The bowfin genome illuminates the developmental evolution of ray-finned fishes. *Nat. Genet.* 53:1373–1384.

Title PO, Singhal S, Grundler MC, Costa GC, Pyron RA, Colston TJ, Grundler MR, Prates I, Stepanova N, Jones MEH, et al. 2024. The macroevolutionary singularity of snakes. *Science* 383:918–923.

Tomiya S. 2011. A New Basal Caniform (Mammalia: Carnivora) from the Middle Eocene of North America and Remarks on the Phylogeny of Early Carnivorans. *PLOS ONE* 6:e24146.

Tomiya S, Tseng ZJ. 2016. Whence the beardogs? Reappraisal of the Middle to Late Eocene 'Miacis' from Texas, USA, and the origin of Amphicyonidae (Mammalia, Carnivora). *R. Soc. Open Sci.* 3:160518.

Torres CR, Norell MA, Clarke JA. 2021. Bird neurocranial and body mass evolution across the end-Cretaceous mass extinction: The avian brain shape left other dinosaurs behind. *Sci. Adv.* 7:eabg7099.

Townsend TM, Larson A, Louis E, Macey JR. 2004. Molecular Phylogenetics of Squamata: The Position of Snakes, Amphisbaenians, and Dibamids, and the Root of the Squamate Tree. *Syst. Biol.* 53:735–757.

Trujillo K, Kowallis B. 2015. Recalibrated legacy 40Ar/39Ar ages for the Upper Jurassic Morrison Formation, Western Interior, U.S.A. *Geol. Intermt. West* 2:1–8.

Tyler JC, Santini F. 2005. A phylogeny of the fossil and extant zeiform-like fishes, Upper Cretaceous to Recent, with comments on the putative zeomorph clade (Acanthomorpha). *Zool. Scr.* 34:157–175.

Upham NS, Esselstyn JA, Jetz W. 2021. Molecules and fossils tell distinct yet complementary stories of mammal diversification. *Curr. Biol.* 31:4195-4206.e3.

bioRxiv preprint doi: https://doi.org/10.64898/2026.06.28.735080; this version posted June 29, 2026. The copyright holder for this preprint (which was not certified by peer review) is the author/funder, who has granted bioRxiv a license to display the preprint in perpetuity. It is made available under a CC-BY 4.0 International license.

Velazco PM, Buczek AJ, Hoffman E, Hoffman DK, O'Leary MA, Novacek MJ. 2022. Combined data analysis of fossil and living mammals: a Paleogene sister taxon of Placentalia and the antiquity of Marsupialia. *Cladistics* 38:359–373.

Vianey-Liaud M, Marivaux L. 2021. The beginning of the adaptive radiation of Theridomorpha (Rodentia) in Western Europe: morphological and phylogenetic analyses of early and middle Eocene taxa; implications for systematics. *Palaeovertebrata* 44:2-e2.

Vidal N, Hedges SB. 2005. The phylogeny of squamate reptiles (lizards, snakes, and amphisbaenians) inferred from nine nuclear protein-coding genes. *C. R. Biol.* 328:1000–1008.

Vlachos E. 2018. A Review of the Fossil Record of North American Turtles of the Clade Pan-Testudinoidea. *Bull. Peabody Mus. Nat. Hist.* 59:3–94.

Wang H, Wang Y. 2023. Middle ear innovation in Early Cretaceous eutherian mammals. *Nat. Commun.* 14:6831.

Wang K, Wang J, Zhu C, Yang L, Ren Y, Ruan J, Fan G, Hu J, Xu W, Bi X, et al. 2021. African lungfish genome sheds light on the vertebrate water-to-land transition. *Cell* 184:1362-1376.e18.

Wang X, Tedford R. 1996. Canidae. In: p. 433–452.

Wang Z, Pascual-Anaya J, Zadissa A, Li W, Niimura Y, Huang Z, Li C, White S, Xiong Z, Fang D, et al. 2013. The draft genomes of soft-shell turtle and green sea turtle yield insights into the development and evolution of the turtle-specific body plan. *Nat. Genet.* 45:701–706.

Wescott WA, Diggens JN. 1998. Depositional history and stratigraphical evolution of the Sakamena group (Middle Karoo Supergroup) in the southern Morondava Basin, Madagascar. *J. Afr. Earth Sci.* 27:461–479.

Wesley-Hunt GD, Flynn JJ. 2005. Phylogeny of the carnivora: Basal relationships among the carnivoramorphans, and assessment of the position of 'miacoidea' relative to carnivora. *J. Syst. Palaeontol.* 3:1–28.

Wesley-Hunt GD, Werdelin L. 2005. Basicranial morphology and phylogenetic position of the upper Eocene carnivoramorphan Quercygale. *Acta Palaeontol. Pol.* 50:837.

Wible JR, Rougier GW, Novacek MJ, Asher RJ. 2007. Cretaceous eutherians and Laurasian origin for placental mammals near the K/T boundary. *Nature* 447:1003–1006.

Wilson Mantilla GP, Chester SGB, Clemens WA, Moore JR, Sprain CJ, Hovatter BT, Mitchell WS, Mans WW, Mundil R, Renne PR. 2021. Earliest Palaeocene purgatoriids and the initial radiation of stem primates. *R. Soc. Open Sci.* 8:210050.

bioRxiv preprint doi: https://doi.org/10.64898/2026.06.28.735080; this version posted June 29, 2026. The copyright holder for this preprint (which was not certified by peer review) is the author/funder, who has granted bioRxiv a license to display the preprint in perpetuity. It is made available under a CC-BY 4.0 International license.

Worthy TH, Degrange FJ, Handley WD, Lee MSY. 2017. The evolution of giant flightless birds and novel phylogenetic relationships for extinct fowl (Aves, Galloanseres). *R. Soc. Open Sci.* 4:170975.

Xu G-H. 2019. Osteology and phylogeny of Robustichthys luopingensis, the largest holostean fish in the Middle Triassic. *PeerJ* 7:e7184.

Xu G-H, Zhao L-J, Coates MI. 2014. The oldest ionoscopiform from China sheds new light on the early evolution of halecomorph fishes. *Biol. Lett.* 10:20140204.

Zaher H, Smith KT. 2020. Pythons in the Eocene of Europe reveal a much older divergence of the group in sympatry with boas. *Biol. Lett.* 16:20200735.

Zhang C, Rabiee M, Sayyari E, Mirarab S. 2018. ASTRAL-III: polynomial time species tree reconstruction from partially resolved gene trees. *BMC Bioinformatics* 19:153.

Zhao W, Zhang X, Jia G, Shen Y, Zhu M. 2021. The Silurian-Devonian boundary in East Yunnan (South China) and the minimum constraint for the lungfish-tetrapod split. *Sci. China Life Sci.* 64:1.

Zheng Y, Wiens JJ. 2016. Combining phylogenomic and supermatrix approaches, and a time-calibrated phylogeny for squamate reptiles (lizards and snakes) based on 52 genes and 4162 species. *Mol. Phylogenet. Evol.* 94:537–547.

Zhu M, Yu X, Ahlberg PE. 2001. A primitive sarcopterygian fish with an eyestalk. *Nature* 410:81–84.

Zhu M, Yu X, Ahlberg PE, Choo B, Lu J, Qiao T, Qu Q, Zhao W, Jia L, Blom H, et al. 2013. A Silurian placoderm with osteichthyan-like marginal jaw bones. *Nature* 502:188–193.

Zhu M, Zhao W, Jia L, Lu J, Qiao T, Qu Q. 2009. The oldest articulated osteichthyan reveals mosaic gnathostome characters. *Nature* 458:469–474.

Zhu Y, Giles S, Young GC, Hu Y, Bazzi M, Ahlberg PE, Zhu M, Lu J. 2021. Endocast and Bony Labyrinth of a Devonian "Placoderm" Challenges Stem Gnathostome Phylogeny. *Curr. Biol.* 31:1112-1118.e4.

Figure S1. Comparison of phylogenetic trees. Simplified phylogenies of jawed vertebrates comparing the topologies recovered using ASTRAL-III or maximum likelihood analysis of concatenated sequence data for the whole 1105 exon dataset (top row) and the 75% complete

bioRxiv preprint doi: https://doi.org/10.64898/2026.06.28.735080; this version posted June 29, 2026. The copyright holder for this preprint (which was not certified by peer review) is the author/funder, who has granted bioRxiv a license to display the preprint in perpetuity. It is made available under a CC-BY 4.0 International license.

matrix (bottom row). Clades are color-coded according to their parent lineages: *Chondrichthyes* in black, non-tetrapod sarcopterygians in red, *Amphibia* in yellow, *Euteleostei* in dark blue.

Figure S2. Complete concatenated phylogenies. Phylogeny of 540 jawed vertebrates recovered using maximum likelihood analysis of concatenated sequence data for the whole 1105 exon dataset.

Figure S3. Complete concatenated phylogenies. Phylogeny of 540 jawed vertebrates recovered using maximum likelihood analysis of concatenated sequence data for the 662 (75% complete matrix) exon dataset.

Figure S4. Relationships among gene and site concordance factors, bootstrap support values, and branch lengths. Scatterplots show relationships between support values and branch lengths for the whole 1105 exon dataset (A) and the 75% complete matrix (B).

Figure S5. Summary of nodal support across phylogenies. Violin plots show (A) coalescent support values under 1.0 across different ASTRAL-III species trees, (B) minimum number of anomalous branches for nodes in anomaly zones in the two analyzed ASTRAL-III species trees, and (C) gene and (D) site concordance factors across the phylogenies inferred via maximum likelihood analyses of concatenated exon sequences. 1105 = all exons, 662 = 75% complete matrix.

Figure S6. Main time-calibrated phylogeny of jawed vertebrates, Part I. Phylogeny of jawed vertebrates (*Gnathostomata*) estimated under a Bayesian node-dating approach, showing the relationships of *Chondrichthyes* and *Sarcopterygii*. Bars at nodes represent 95% highest posterior density intervals for divergence times. Abbreviations: O, Ordovician; S, Silurian; D, Devonian; C, Carboniferous; P, Permian; Tr, Triassic; J, Jurassic; K, Cretaceous; Pg, Paleogene; N, Neogene.

Figure S7. Main time-calibrated phylogeny of jawed vertebrates, Part II. Phylogeny of jawed vertebrates (*Gnathostomata*) estimated under a Bayesian node-dating approach, showing

bioRxiv preprint doi: https://doi.org/10.64898/2026.06.28.735080; this version posted June 29, 2026. The copyright holder for this preprint (which was not certified by peer review) is the author/funder, who has granted bioRxiv a license to display the preprint in perpetuity. It is made available under a CC-BY 4.0 International license.

the relationships of *Actinopterygii.* Bars at nodes represent 95% highest posterior density intervals for divergence times. Abbreviations: O, Ordovician; S, Silurian; D, Devonian; C, Carboniferous; P, Permian; Tr, Triassic; J, Jurassic; K, Cretaceous; Pg, Paleogene; N, Neogene.

Figure S8. Comparison of estimated divergence times. Plot shows estimated median ages and 95% HPD intervals for selected clades of jawed vertebrates found in the time tree made by pooling all three sets of 50 exons and from trees built from each exon set. Abbreviations: O, Ordovician; S, Silurian; D, Devonian; C, Carboniferous; P, Permian; Tr, Triassic; J, Jurassic; K, Cretaceous; Pg, Paleogene; N, Neogene.

Figure S9. Signature of the Cretaceous-Paleogene Mass Extinction on Jawed Vertebrate Phylogenetic Resolution. Violin plots show gene and site concordance factors for nodes that appear within 10 million years of the Cretaceous-Paleogene boundary (66.02 Ma)(Gradstein et al. 2021) and grouped according to (A) ecology and (B) clade.

Figure S10. Rates of Jawed Vertebrate Evolution Part I. Hypothesis of how variation in rates of nucleotide substitution (A) can distort the inferred timescale (B) of ray-finned fish evolution. (C) shows the main time-calibrated phylogeny of jawed vertebrates (see Figure 1) and trees built by annotating the target tree using posterior tree sets from analyses of each exon set, with branches colored according to their inferred substitution rate values (substitutions per site million years. Note the very high backbone rates associated with *Actinopterygii*.

Figure S11. Rates of Jawed Vertebrate Evolution Part II. Panel (A) shows the test analysis in which living fossil lineages (*Acipenseriformes*, *Holostei*) were excluded (see Figure 5, Methods), panel (B) shows the full reduced sampling time-calibrated phylogeny of jawed vertebrates (see Figure 5), and panel (C) shows the full reduced sampling time-calibrated phylogeny of jawed vertebrates, but with no fossil calibrations for living fossil clades (see Figure 5).

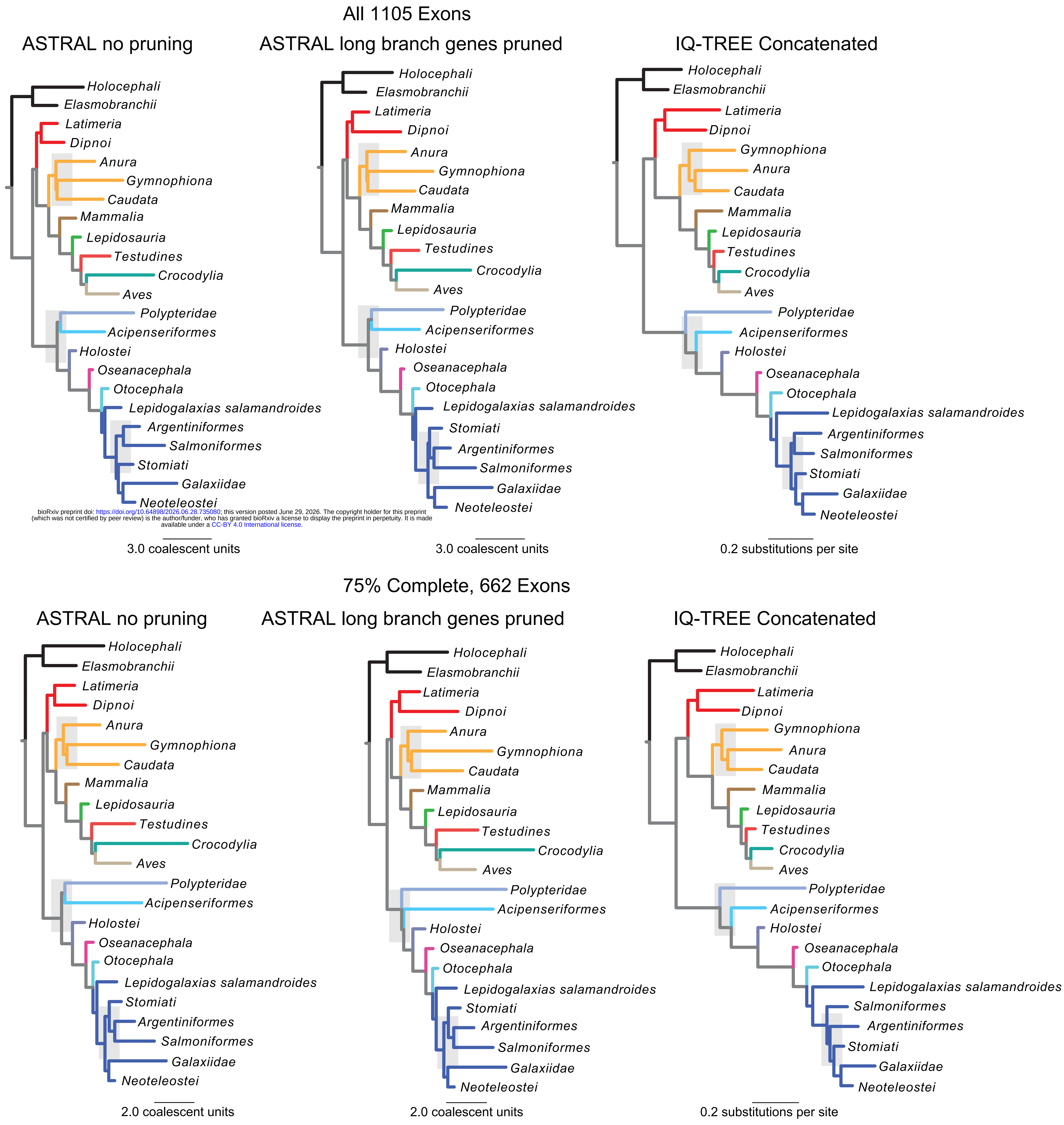

bioRxiv preprint doi: https://doi.org/10.64898/2026.06.28.735080; this version posted June 29, 2026. The copyright holder for this preprint (which was not certified by peer review) is the author/funder, who has granted bioRxiv a license to display the preprint in perpetuity. It is made available under a CC-BY 4.0 International license.

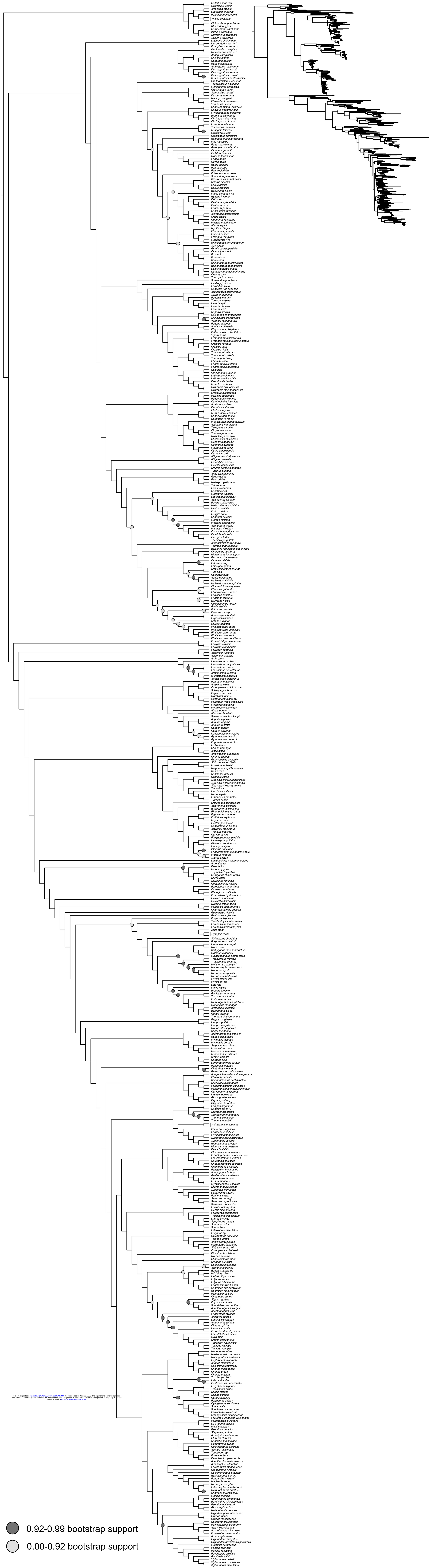

bioRxiv preprint doi: https://doi.org/10.64898/2026.06.28.735080; this version posted June 29, 2026. The copyright holder for this preprint (which was not certified by peer review) is the author/funder, who has granted bioRxiv a license to display the preprint in perpetuity. It is made available under a CC-BY 4.0 International license.

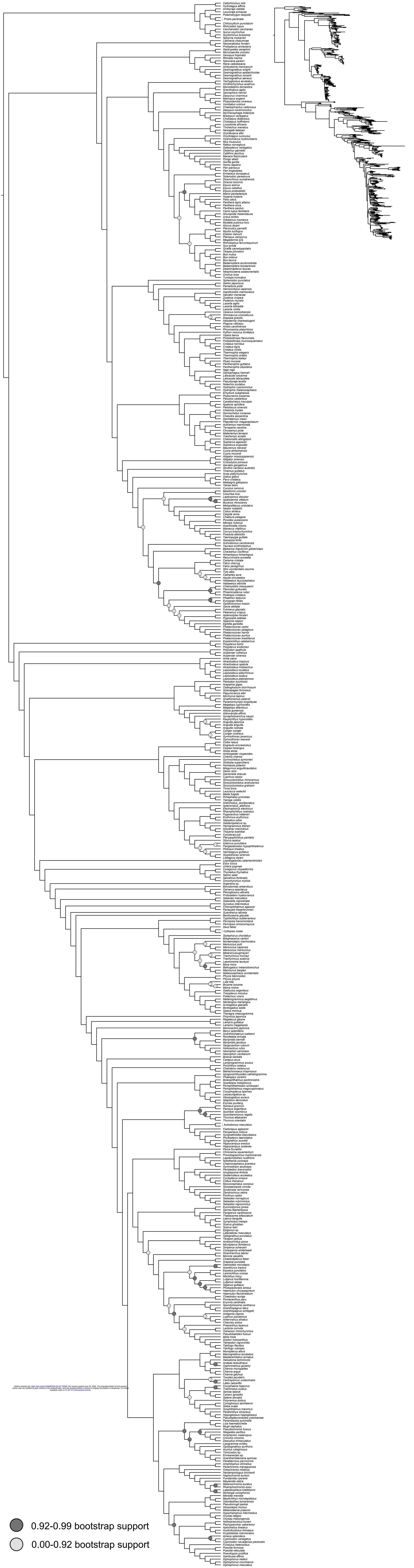

bioRxiv preprint doi: https://doi.org/10.64898/2026.06.28.735080; this version posted June 29, 2026. The copyright holder for this preprint (which was not certified by peer review) is the author/funder, who has granted bioRxiv a license to display the preprint in perpetuity. It is made available under a CC-BY 4.0 International license.

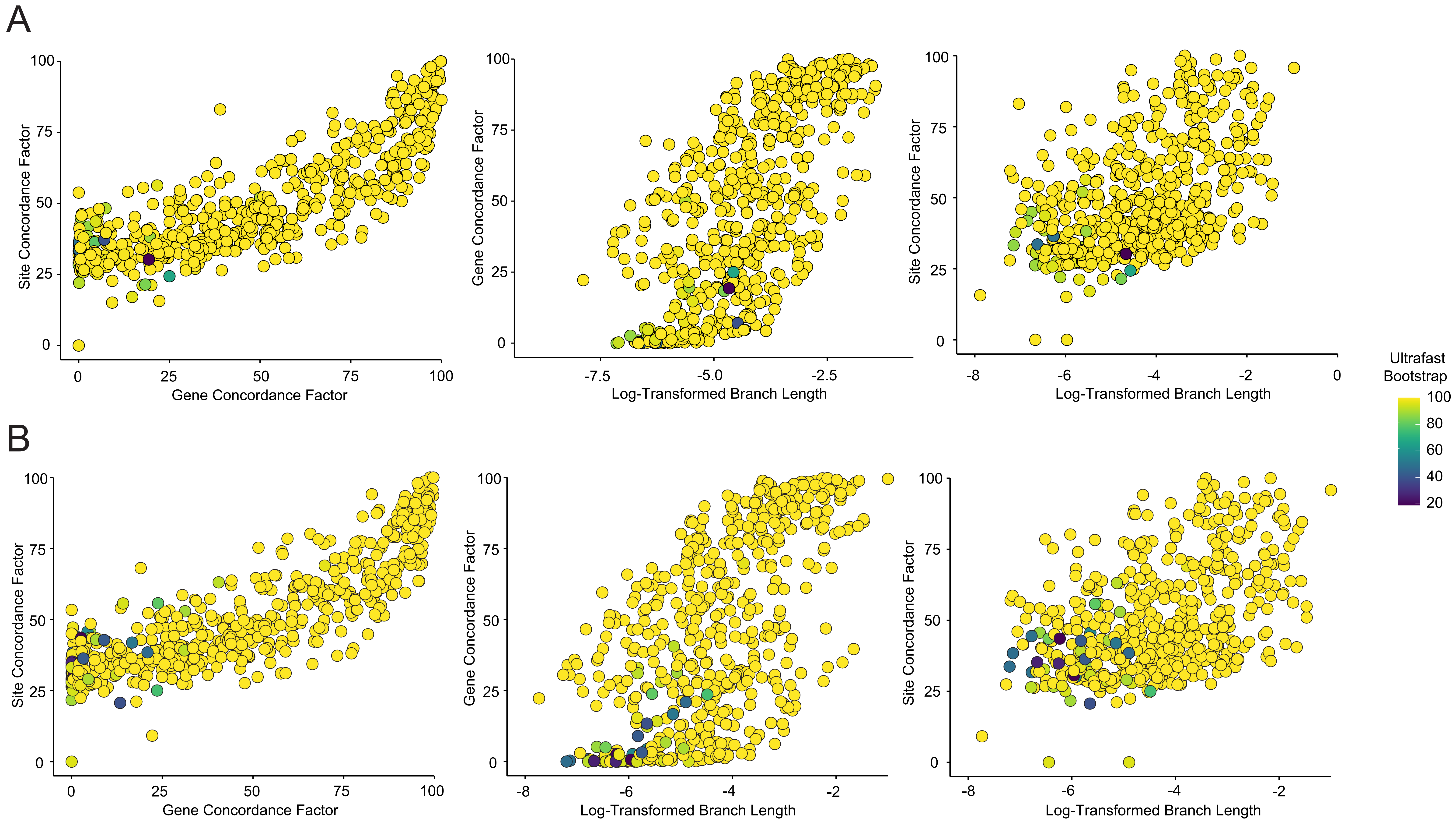
A
B
Site Concordance Factor
Gene Concordance Factor
Log-Transformed Branch Length
Ultrafast Bootstrap
100
80
60
40
20

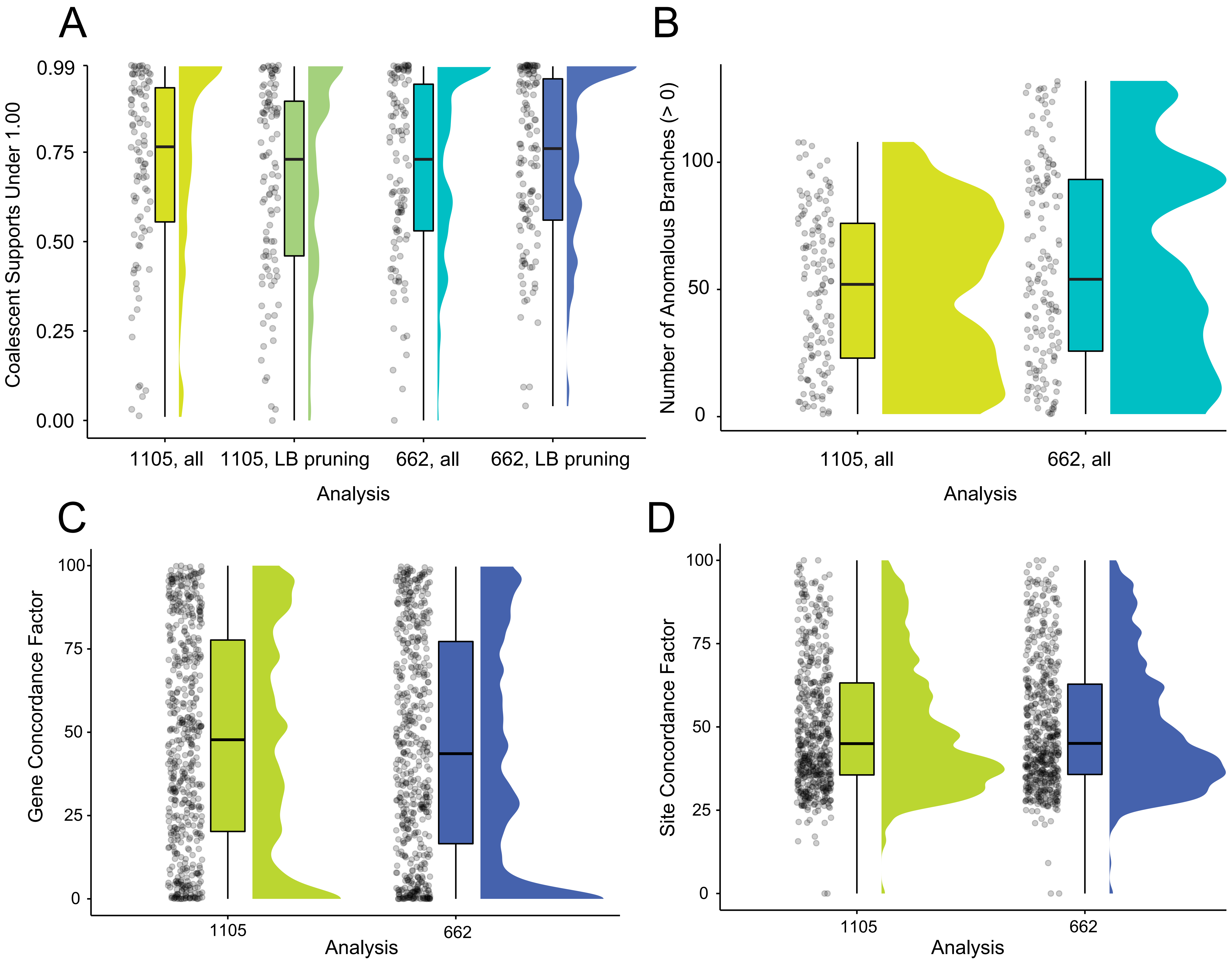

A
Coalescent Supports Under 1.00
0.99
0.75
0.50
0.25
0.00
1105, all
1105, LB pruning
662, all
662, LB pruning
Analysis
B
Number of Anomalous Branches (> 0)
100
50
0
1105, all
662, all
Analysis
C
Gene Concordance Factor
100
75
50
25
0
1105
662
Analysis
D
Site Concordance Factor
100
75
50
25
0
1105
662
Analysis

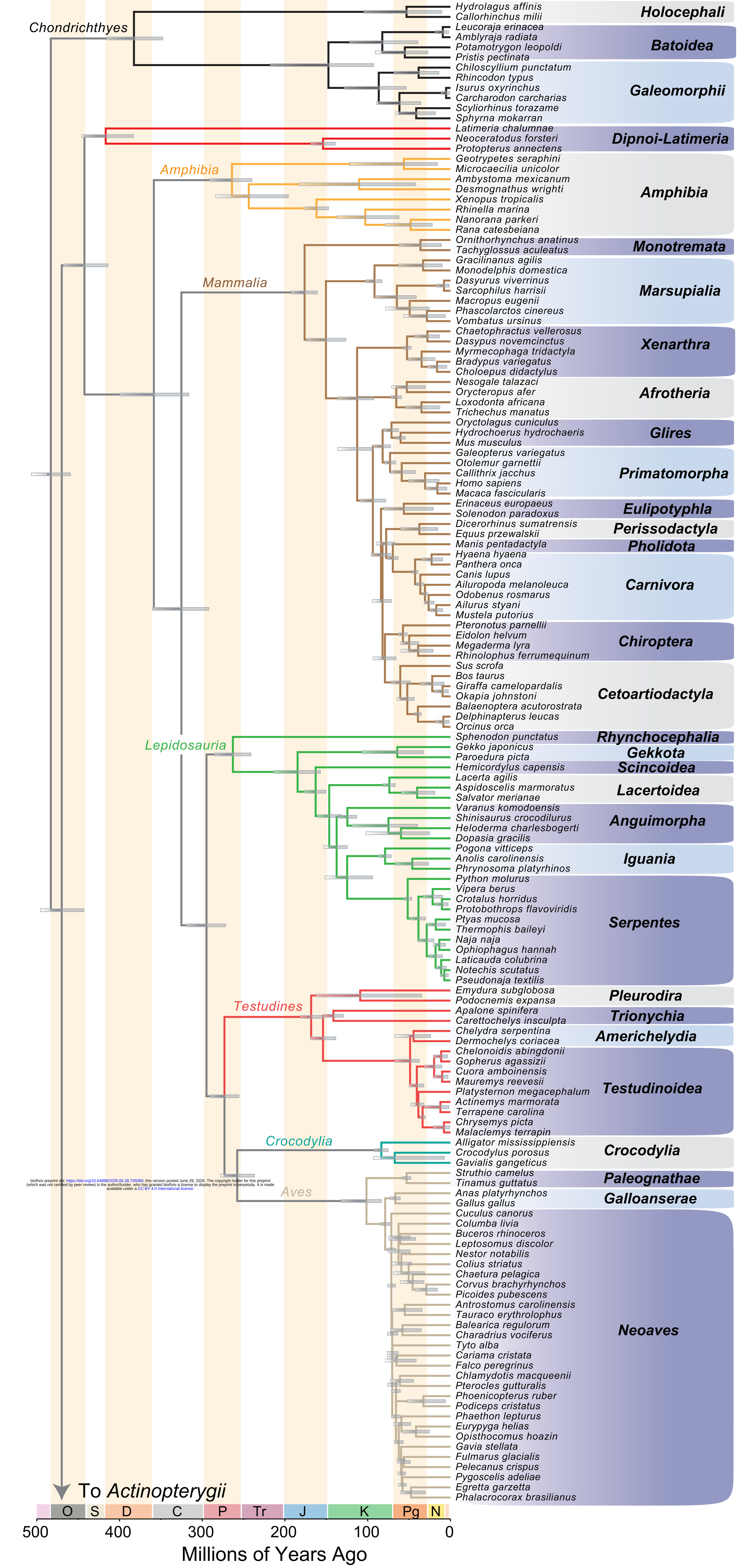

bioRxiv preprint doi: https://doi.org/10.64898/2026.06.28.735080; this version posted June 29, 2026. The copyright holder for this preprint (which was not certified by peer review) is the author/funder, who has granted bioRxiv a license to display the preprint in perpetuity. It is made available under a CC-BY 4.0 International license.

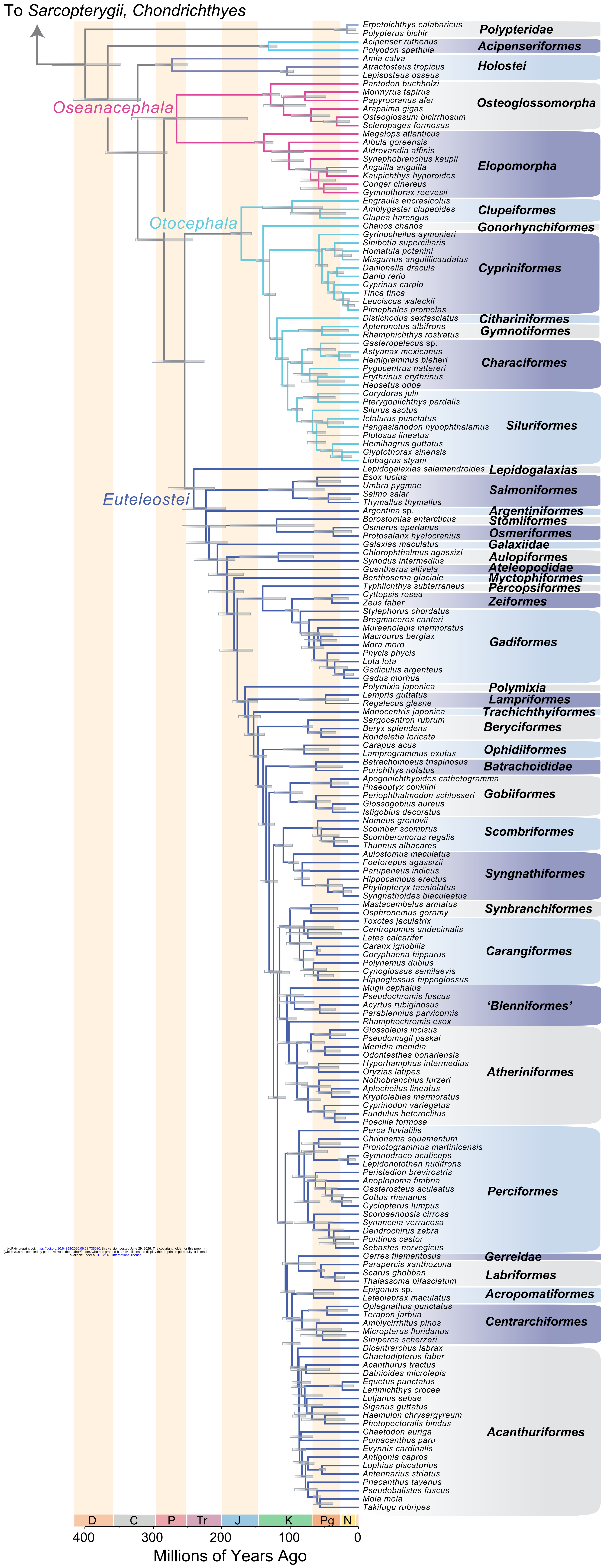

bioRxiv preprint doi: https://doi.org/10.64898/2026.06.28.735080; this version posted June 29, 2026. The copyright holder for this preprint (which was not certified by peer review) is the author/funder, who has granted bioRxiv a license to display the preprint in perpetuity. It is made available under a CC-BY 4.0 International license.

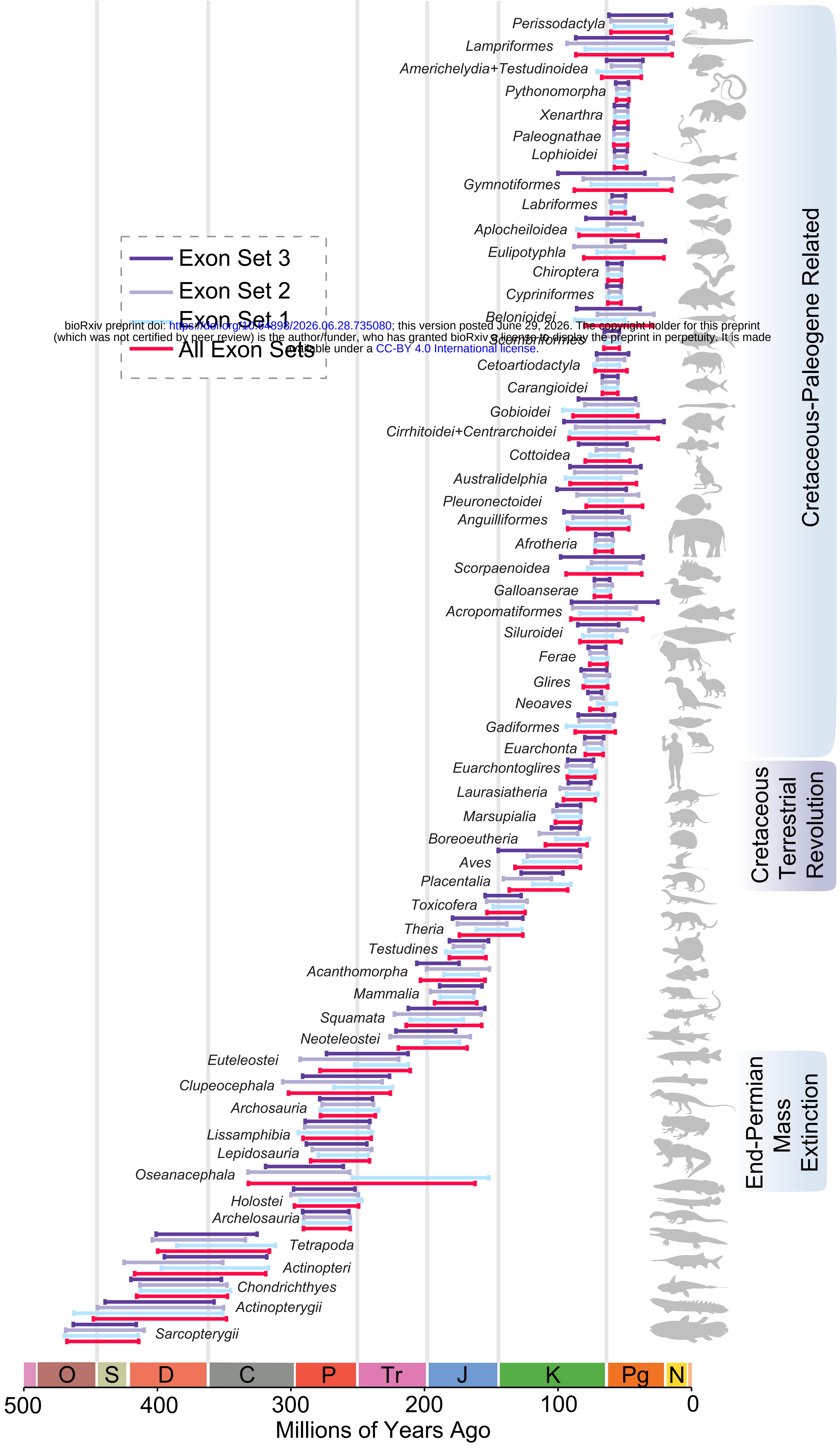

bioRxiv preprint doi: https://doi.org/10.64898/2026.06.28.735080; this version posted June 29, 2026. The copyright holder for this preprint (which was not certified by peer review) is the author/funder, who has granted bioRxiv a license to display the preprint in perpetuity. It is made available under a CC-BY 4.0 International license.

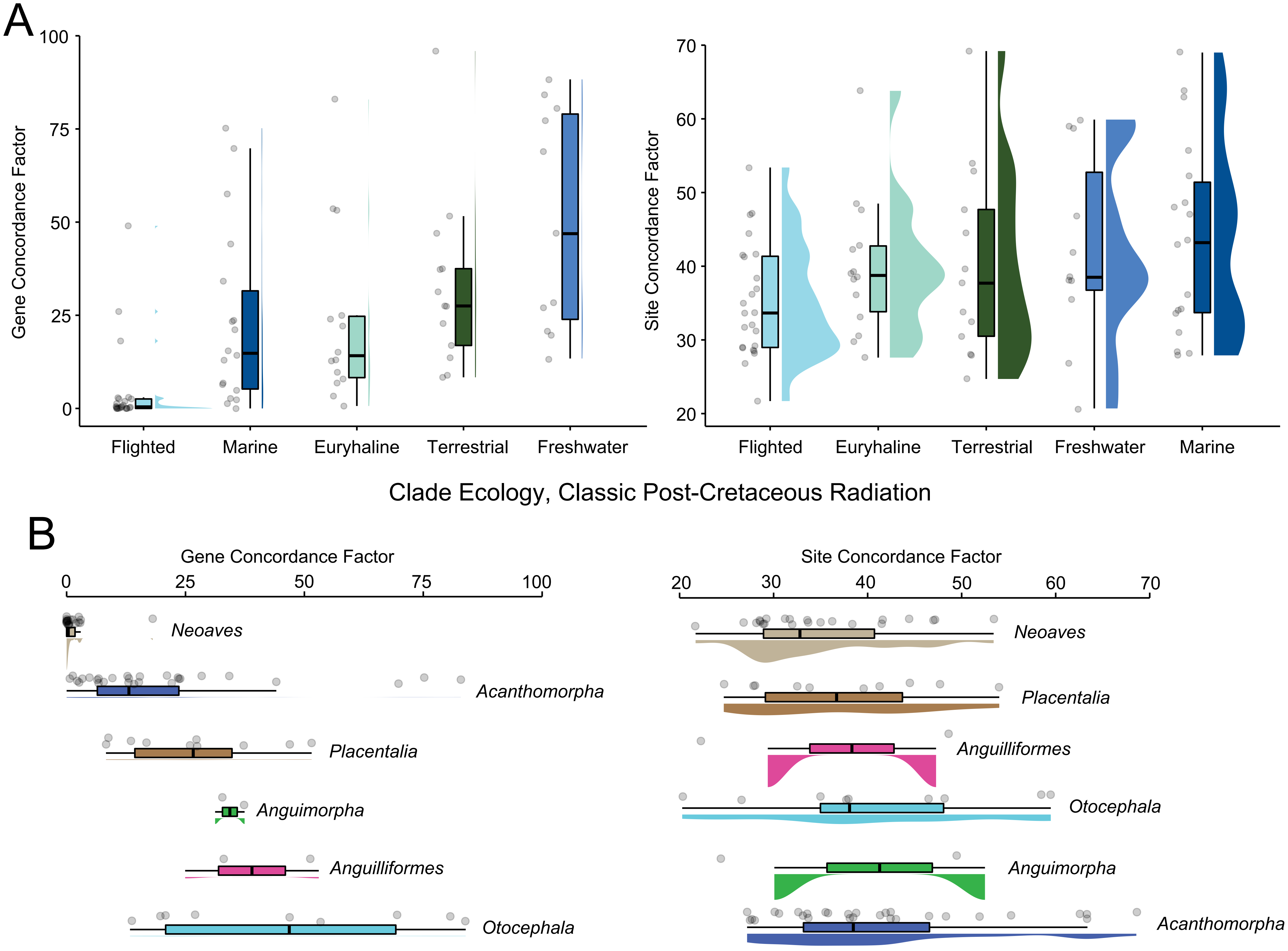

A
Gene Concordance Factor
100
75
50
25
0
Flighted
Marine
Euryhaline
Terrestrial
Freshwater
Site Concordance Factor
70
60
50
40
30
20
Flighted
Euryhaline
Terrestrial
Freshwater
Marine
Clade Ecology, Classic Post-Cretaceous Radiation
B
Gene Concordance Factor
0
25
50
75
100
Neoaves
Acanthomorpha
Placentalia
Anguimorpha
Anguilliformes
Otocephala
Site Concordance Factor
20
30
40
50
60
70
Neoaves
Placentalia
Anguilliformes
Otocephala
Anguimorpha
Acanthomorpha

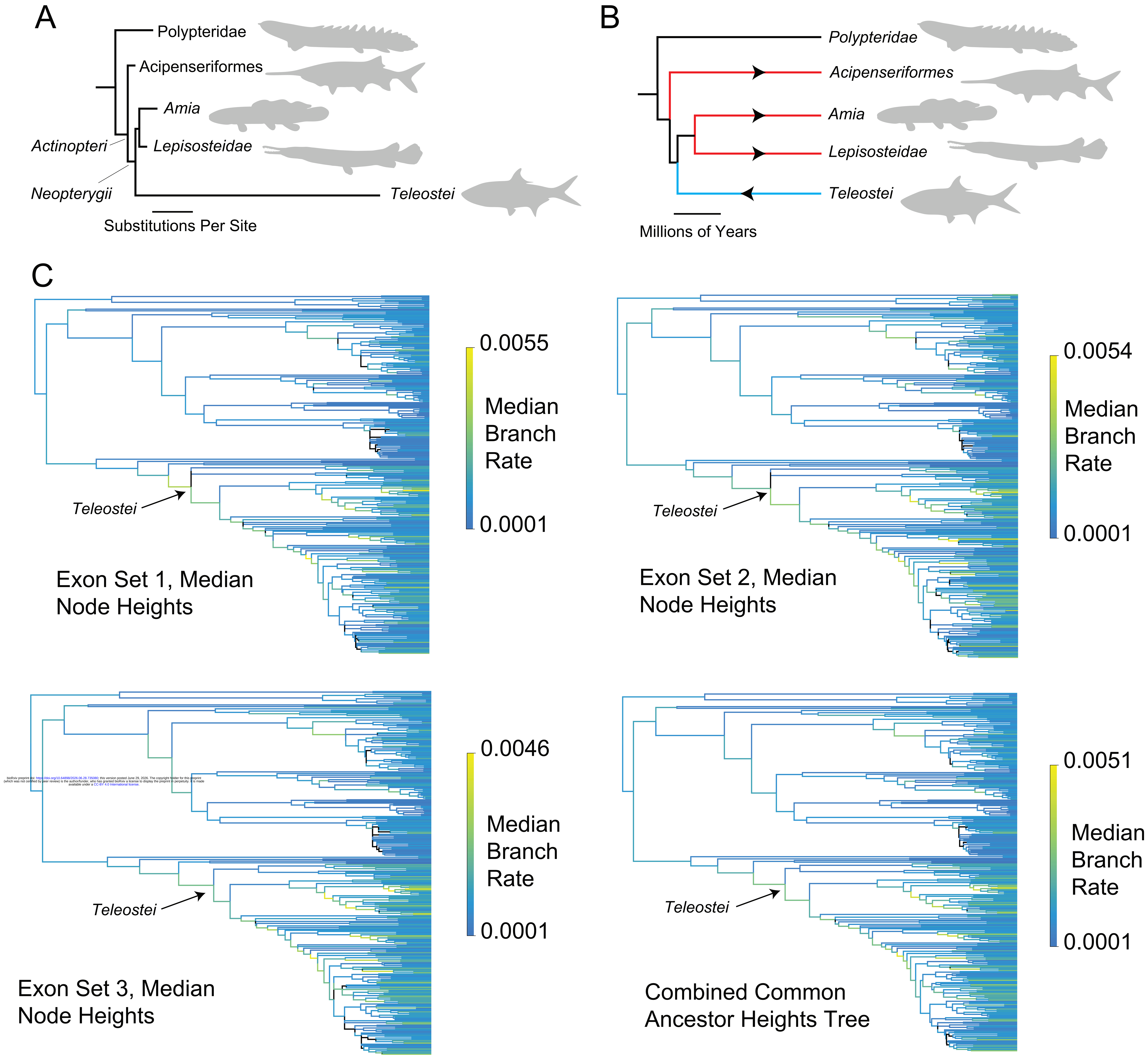

bioRxiv preprint doi: https://doi.org/10.64898/2026.06.28.735080; this version posted June 29, 2026. The copyright holder for this preprint (which was not certified by peer review) is the author/funder, who has granted bioRxiv a license to display the preprint in perpetuity. It is made available under a CC-BY 4.0 International license.

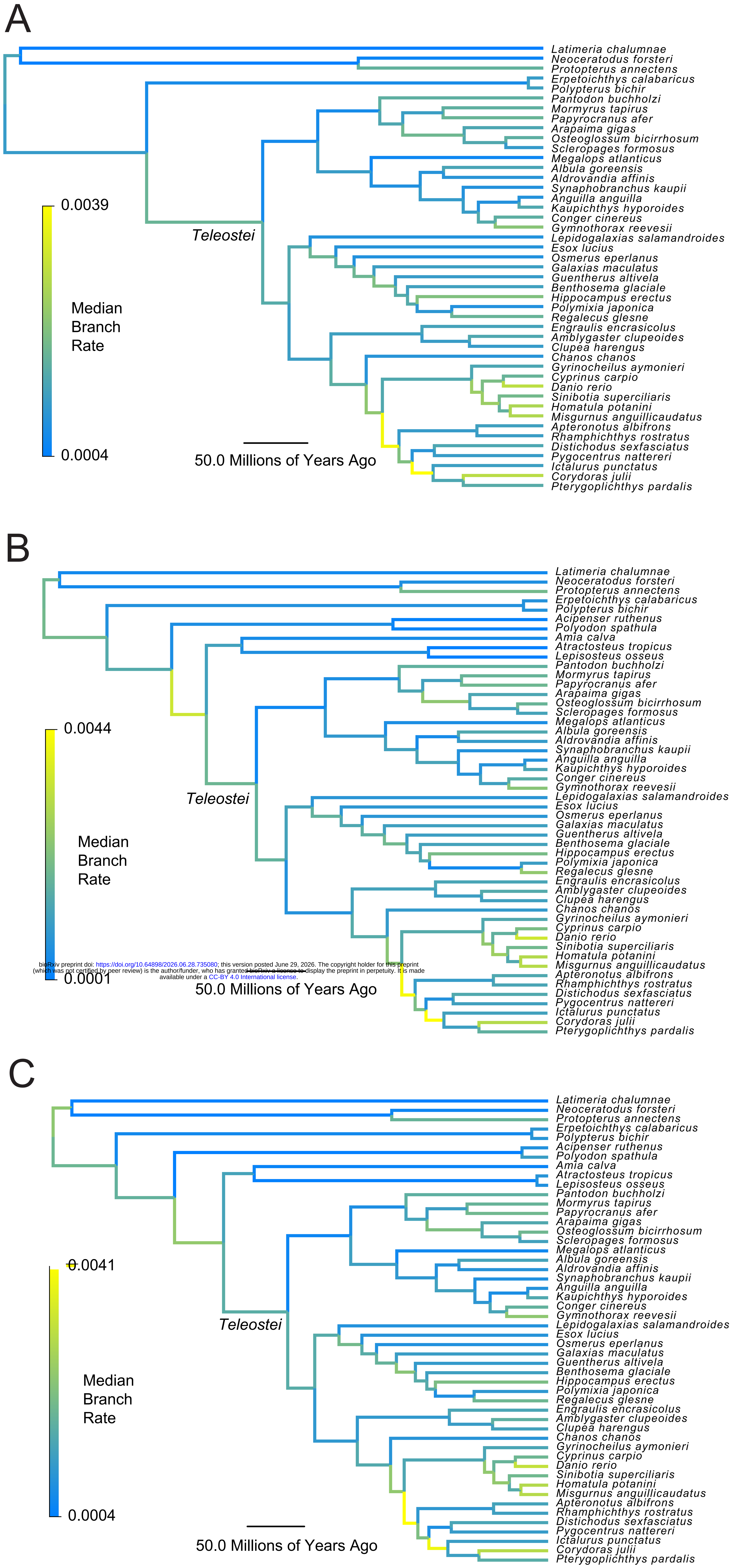

bioRxiv preprint doi: https://doi.org/10.64898/2026.06.28.735080; this version posted June 29, 2026. The copyright holder for this preprint (which was not certified by peer review) is the author/funder, who has granted bioRxiv a license to display the preprint in perpetuity. It is made available under a CC-BY 4.0 International license.
